# Uncovering the Associations of *LILRB4* Genotypes With Parkinson's Disease: From Clinical Traits to Potential Pathologies

**DOI:** 10.1111/cns.70522

**Published:** 2025-07-23

**Authors:** Yuting Zhou, Yaqing Li, Qiqing He, Zhen Kong, Ran Yu, Xin Yu, Anmu Xie

**Affiliations:** ^1^ Department of Neurology Affiliated Hospital of Qingdao University Qingdao China; ^2^ Cerebral Vascular Disease Institute, Affiliated Hospital of Qingdao University Qingdao China; ^3^ NO.971 Hospital of the People's Liberation Army Navy Qingdao Shandong Province China

**Keywords:** DAT‐SPECT, DTI, LILRB4, Parkinson's disease, semantic fluency

## Abstract

**Background:**

Leukocyte immunoglobulin‐like receptor B4 (LILRB4) has been shown to be associated with susceptibility to neurodegenerative diseases. This study was aimed at investigating the relationships between *LILRB4* and the risk of developing PD, as well as its clinical characteristics and pathology.

**Method:**

We analyzed 197 healthy controls and 606 PD patients from the Parkinson's Progression Marker Initiative (PPMI) study. The associations of *LILRB4* loci with image data, CSF biomarkers, and clinical scales at baseline were assessed using multiple linear models.

**Results:**

Dopamine transporter (DAT)‐SPECT results showed that the striatal binding ratios (SBR) in the right caudate (*β* = 0.160, 95% CI = 0.076–0.244, *P*_c_ = 0.002), right putamen (*β* = 0.135, 95% CI = 0.057–0.214, *P*_c_ = 0.009), anterior right putamen (*β* = 0.156, 95% CI = 0.073–0.240, *P*_c_ = 0.003) and left caudate (*β* = 0.121, 95% CI = 0.038–0.2050, *P*_c_ = 0.048) were positively associated with *LILRB4*. Meanwhile, *LILRB4* was associated with reductions in semantic fluency (*β* = −2.135, 95% CI = −3.225 to 1.046, *P*_c_ = 0.001) and impairments in nigrostriatal white matter (WM) microstructure as assessed by diffusion tensor imaging (DTI) (right rostral of substantia nigra (SN), *β* = −0.026, 95% CI = −0.030 to 0.013, *P*_c_ = 0.002; right middle SN, *β* = −0.021, 95% CI = −0.033 to 0.009, *P*_c_ = 0.012). These associations were more prominent in females (DAT in right caudate, *β* = 0.246, 95% CI = 0.099–0.392, *P*_c_ = 0.013; DTI in right middle of SN, *β* = −0.025 95% CI = −0.040 to 0.010, *P*_c_ = 0.021), but less pronounced in males (DAT in right caudate, *p* = 0.036, *P*_c_ = 0.396; DTI in right rostral of SN, *β* = −0.025 95% CI = −0.041 to 0.008, *P*_c_ = 0.021). Interestingly, in females, we also observed associations between *LILRB4* and higher CSF α‐synuclein levels (*β* = 0.177, 95% CI = 0.062–0.292, *p* = 3.280E−03, *P*_c_ = 0.036) and worse cognitive performance (Activity of Daily Living scale, *β* = −2.073, 95% CI = −3.446 to 0.699, *P*_c_ = 0.025; Semantic Fluency test, *β* = −2.508 95% CI = −4.255 to 0.761, *P*_c_ = 0.032). Although our results suggested that dopamine and its metabolites, astrocyte markers, and inflammation‐related molecules were associated with *LILRB4*, these associations disappeared after false discovery rate (FDR) correction (*p* ≤ 0.05, but *P*_c_ > 0.05).

**Conclusion:**

Our study supposes that LILRB4 may play a crucial role in modulating PD clinical characteristics by influencing nigrostriatal dopaminergic neuron function, Alzheimer's disease (AD)‐related pathology, WM microstructural alterations, and astrocyte activation.

## Graphical abstract

This study explored the effects of *LILRB4* on PD by analyzing the association between *LILRB4* and PD clinical characteristics and hallmarks. *LILRB4* might be vital in gatekeeping PD clinical characteristics by tuning nigrostriatal dopaminergic neuron function, AD‐related pathology, WM microstructural alteration, and astrocyte activation.

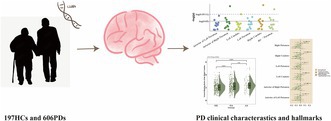

## 1 Introduction

Parkinson's disease (PD) is a common neurodegenerative disorder characterized by the accumulation of α‐synuclein aggregates in neurons and the degeneration of nigrostriatal dopaminergic neurons [1]. In general, PD is well known for its typical motor symptoms (resting tremor, myotonia, bradykinesia), but nonmotor symptoms, especially cognitive deficits, impose a significant burden on patients' quality of life [2]. In the course of PD, mild cognitive impairment (MCI) can emerge early in PD progression, even preceding the onset of motor deficits [3]. In addition, beta amyloid (Aβ), t‐tau, *p*‐tau, as the major sign of cognitive impairment in Alzheimer's disease (AD) pathology, also contribute to PD pathology [4]. Recent studies showed that these proteins would interact with α‐synuclein and promote its aggregation and dissemination [5, 6], and might be potential biomarkers of cognitive decline in PD [4].

Leukocyte immunoglobulin‐like receptor B4 (LILRB4), an inhibitory receptor in the LILR family, is widely expressed on various immune cells [7]. Its engagement with specific ligands triggers multiple signaling cascades, modulating immune responses under both physiological and pathological conditions [8]. Emerging evidence indicates the upregulation of *LILRB4* expression in several neurodegeneration diseases [9, 10, 11]. Specifically, in studies of different transgenic mouse models of amyloidosis, the degree of LILRB4 transcript upregulation correlates with Aβ pathology progression and parallels microglial activation [9, 12]. As LILRB serves as a key surface marker for microglia activation [13], its expression in microglia wrapping around Aβ plaques in AD models has been linked to cognitive improvement [14]. Meanwhile, microglia also played an active role in PD pathology by arousing neuroinflammation [15]. Therefore, these findings underscore the involvement of LILRB4 in neurodegenerative processes. However, its specific role in PD pathology and clinical manifestations remains to be elucidated.

This study aims to determine the association between *LILRB4* polymorphism and PD clinical characteristics (e.g., typical motor and nonmotor functions) and PD pathological biomarkers (e.g., neuroimaging biomarkers and cerebrospinal fluid [CSF] biomarkers), thus clarifying the effect of *LILRB4* on PD. We further hypothesize that these associations would serve as clinical indicators of the current condition and prognosis for individual patients, which helps to deepen understanding of PD pathology and inform targeted therapeutic strategies.

## 2 Materials and Methods

All the data used in this study were obtained on 6 Jun 2023 from Parkinson's Progression Markers Initiative (PPMI) database (https://ida.loni.usc.edu/pages/access/studyData.jsp?project=PPMI). The study was conducted in agreement with the principles of the Declaration of Helsinki. Signed informed consent was obtained from all participants recruited.

### 2.1 Study Participants

All participants of the PD cohort had a clinical diagnosis of PD and a positive dopamine transporter (DAT)‐SPECT. While in healthy control (HC), participants all had no current or active clinically significant neurological disorder, no family history of PD, and normal DAT‐SPECT. Excluding participants younger than 30 years of age, 606 PD patients and 197 healthy controls were ultimately enrolled in our study. The study was approved by the institutional review board at each PPMI site. All patients signed an informed consent form before their participation in the PPMI study.

### 2.2 SNPs Selection

**FIGURE 1:**
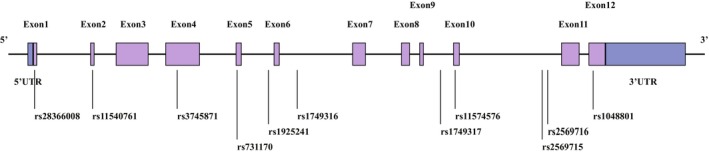
Schematic of SNPs in the *LILRB4* gene. UTR, untranslated region.

Whole‐genome sequencing data was extracted from the PPMI database (https://ida.loni.usc.edu/pages/access/geneticData.jsp). We used PLINK to prune SNPs that were out of criteria. In detail, SNPs that were not in Hardy–Weinberg equilibrium (*p* < 0.05) and whose minor allele frequency was below 20% were excluded. SNP pruning parameters were listed below: window size 10, increment 5, and variance inflation factor 2. The remaining 11 SNPs were included in our analysis (Table 1 and Figure 1).

**TABLE 1:** *LILRB4* SNPs and previous reports related.

SNPs	Alleles	Position (GRCH38)	Annotation	MAF	Previous report
rs28366008	T>A	19:54663046	Exon1	0.250	/
rs11540761	G>T	19:54663551	Exon2	0.193	MDCs and PDCs surface expression of ILT3↓; serum type I IFN activity↑^a^
rs3745871	T>C	19:54664289	Exon4	0.617	/
rs731170	G>A	19:54664811	Exon5	0.319	/
rs1925241	T>C	19:54665084	Intron	0.588	/
rs1749316	C>T	19:54665335	Intron	0.139	/
rs1749317	A>G	19:54666584	Intron	0.268	/
rs11574576	A>G	19:54666711	Exon10	0.237	/
rs2569715	A>G	19:54667469	Intron	0.345	/
rs2569716	A>G	19:54667517	Intron	0.367	/
rs1048801	G>A	19:54667913	Exon12	0.616	serum type I IFN activity↑; serum TNF‐α↑^a^

Abbreviations: MAF, minor allele frequency; MDCs, myeloid dendritic cells; PDCs, plasmacytoid dendritic cells.

^a^ Jensen, M.A., et al. Functional genetic polymorphisms in ILT3 are associated with decreased surface expression on dendritic cells and increased serum cytokines in lupus patients. *Ann Rheum Dis*. **72**, 596–601 (2013).

### 2.3 Brain Function and Structure on Imaging

DAT‐SPECT was an imaging test to visualize the distribution of DAT and to assess the functional status of dopaminergic neurons. DATs were localized on dopaminergic axons in the striatum and regulated extracellular dopamine levels. The decrease of DAT was regarded as important pathology evidence in PD [16]. Left and right caudate, left and right putamen, and the occipital cortex (reference tissue) were selected as regions of interest (ROIs). The striatal binding ratios (SBR) were calculated as (target region densities/reference region densities)‐1 (https://ida.loni.usc.edu/pages/access/studyData.jsp?categoryId=6&subCategoryId=12).

Diffusion tensor imaging (DTI) was used to observe the structural integrity and connectivity of cerebral white matter (WM) tissue to assess the degree and extent of WM microstructural change from various diseases. Commonly used metrics included the mean diffusivity (MD), fractional anisotropy (FA) and relative anisotropy (RA). Progressive neuronal loss in neurodegenerative diseases such as PD was associated with progressive degeneration of associated WM tracts as measured by DTI [17]. Placed 6 ROIs on the left and right substantia nigra (SN), covering the caudal, middle, and rostral aspects of the structure; then selected the left and right cerebral peduncle as reference measures (https://ida.loni.usc.edu/pages/access/studyData.jsp?categoryId=6&subCategoryId=53).

### 2.4 Biospecimen Analysis

The cerebrospinal fluid (CSF) was collected at baseline. Levels of CSF Aβ, Aβ1‐42, total tau (t‐tau), and phosphorylated tau (*p*‐tau) 181 were measured using electrochemiluminescence (ECL) immunoassays. Concentrations of CSF α‐synuclein, glial fibrillary acid protein (GFAP), Interleukin 6 (IL‐6), S‐100 calcium binding protein B (S100B), neurofilament light (NFL), soluble triggering receptor expressed on myeloid cells 2 (sTREM2) and chitinase‐3‐like protein 1 (YKL40) were analyzed using Elecsys Immunoassay. Concentration of Interleukin 1b (IL‐1b) was analyzed using enzyme linked immunosorbent assay (ELISA). Concentrations of catecholamines and related metabolites will be determined by Metabolites Health, Berlin/Germany (see www.metanomics‐health.com). These data were downloaded from the PPMI dataset.

### 2.5 Motor and Nonmotor Function Assessments

Motor and nonmotor function scales were a crucial portion of the diagnosis and evaluation of PD. MDS Unified‐Parkinson Disease Rating Scale (MDS‐UPDRS) was used to assess motor function. The following were used for nonmotor function (especially for cognition) assessments: Modified Schwab & England Activities of Daily Living (ADL), Benton Judgment of Line Orientation (BJLOT), Hopkins Verbal Learning Test (HVLT), Letter‐Number Sequencing (LNS), Lexical Fluency, Modified Boston Naming Test (BNT), Modified Semantic Fluency, Montreal Cognitive Assessment (MoCA). All the data were downloaded from the PPMI dataset.

### 2.6 Statistical Analysis

All statistical analyses were performed by R 4.3.1 and PLINK 1.9. To initially describe demographics and different clinical characteristics, continuous variables were analyzed by using a *t*‐test, while dichotomous variables were assessed using the chi‐square test (Χ^2^ test). Quantile‐quantile (Q‐Q) plots were generated to describe the distribution of all continuous variables included in our study. CSF biomarker data were log‐transformed to approximate normality, as the raw CSF data did not conform to a normal distribution. All Q‐Q plots are presented in Figure S1.

In our regression analysis, logistic regression models were used for dichotomous variables, and multiple linear regression models were used for continuous variables to assess the association between *LILRB4* genotypes and various phenotypes at baseline in the entire cohort. First, in our primary model, we included age and sex as covariates to account for their variability in the distribution of PD patients [18]. Second, recognizing that phenotypes are often influenced by multiple factors, such as educational attainment, family income level, genetic factors, and so on, we sequentially tested these factors using linear correlation and logistic regression and included significant factors as additional covariates. Thus, in Model 2, *GBA* and *LRRK2* were added as covariates. Third, given the known correlation between educational attainment and cognitive function in PD, we included educational attainment as a covariate when analyzing the correlation between *LILRB4* and cognitive function in Model 3. The correlations between PD phenotypes and age, sex, education, family income level, and genetic factors were summarized in Table S1.

Since our regression analysis involves 11 SNPs that are independent of each other, false positive results need to be reduced by correcting for thresholds. We used FDR‐correction for multiple comparisons rather than the stricter Bonferroni method to maintain statistical power and balance sensitivity and specificity, consistent with prior research [19]. Specifically, the calculated *p*‐values were sorted from smallest to largest, and the corrected significance threshold was defined as *p* = 0.05 × (k/11) where k is the rank of the *p*‐value. The largest k‐th *p*‐value satisfying *p* ≤ 0.05 was identified, and all *p*‐values up to this threshold were considered significant, while the rest were deemed nonsignificant [20]. In the *Results* section, we used “*p*” to denote the uncorrected *p*‐value and “*P*_c_” to denote the *p*‐value after FDR‐correction.

Additionally, the variance inflation factor (VIF) was used to assess multicollinearity among the fixed‐effects predictor variables in the linear mixed model.

## 3 Results

### 3.1 Clinical Characteristics at Baseline

Listed below were the major information about subjects in our study (Table 2). As expected, HCs and PD patients had no statistical difference in age (HCs, 60.85 ± 11.30 years; PD patients, 61.73 ± 9.880 years, *p* = 0.296) or gender (OR = 0.835, *p* = 0.278). Besides, PD patients had a significantly decreased level of CSF α‐synuclein (*p* < 0.001), worse scores on MDS‐UPDRS (*p* < 0.001), and worse cognition assessments (*p* < 0.001) from both cognition‐related scales and CSF biomarkers.

**TABLE 2:** The characteristics of the subjects at baseline.

Subjects	HCs	PDs	OR/*t* value	*p* *
Age (years)	60.8 ± 11.3	61.7 ± 9.88	*t* = −0.98	0.33
Gender (female/male)	70/127	242/365	OR = 0.84	0.28
Hoehn‐Yahr (0/I/II/III)	195/2/0/0	2/230/356/17	426/1.98E07/5.68E03	**﹤0.01**
MDS‐UPDRS I (R)	0.548 ± 1.09	1.54 ± 1.97	*t* = −6.74	**﹤0.01**
MDS‐UPDRS I (P)	2.42 ± 2.46	4.89 ± 3.66	*t* = −10.8	**﹤0.01**
MDS‐UPDRS II	0.44 ± 1.00	6.49 ± 4.80	*t* = −19.0	**﹤0.01**
MDS‐UPDRS III	1.21 ± 2.19	20.8 ± 9.84	*t* = −45.7	**﹤0.01**
MDS‐UPDRS IV	0.00 ± 0.00	1.92 ± 3.16	*t* = −14.5	**﹤0.01**
MoCA	28.2 ± 1.11	26.7 ± 2.93	*t* = 10.6	**﹤0.01**
ADL	98.4 ± 5.46	92.3 ± 7.84	*t* = 10.2	**﹤0.01**
SFT	52.6 ± 10.4	50.3 ± 10.7	*t* = 2.61	**0.01**
LNS	11.7 ± 2.73	11.2 ± 2.95	*t* = 2.40	**0.02**
HVLT	49.0 ± 10.2	45.3 ± 11.1	*t* = 4.13	**﹤0.01**
BNT	57.0 ± 6.14	54.6 ± 9.57	*t* = 2.98	**﹤0.01**
BJLOT	26.3 ± 3.96	24.8 ± 4.93	*t* = 4.20	**﹤0.01**
α‐synuclein (pg/ml)	127 ± 53.8	102 ± 48.4	*t* = 4.10	**﹤0.01**
Aβ	988 ± 308	892 ± 330	*t* = 0.76	0.45
Aβ1‐42	1.02E03 ± 499	894 ± 406	*t* = 3.16	**﹤0.01**
*p*‐Tau	16.8 ± 8.42	14.3 ± 5.59	*t* = 3.92	**﹤0.01**
t‐Tau	191 ± 80.0	168 ± 61.6	*t* = 3.62	**﹤0.01**

Abbreviations: Aβ, beta amyloid; ADL, modified Schwab & England activities of daily living; BJLOT, Benton judgment of line orientation; BNT, modified Boston naming test; HCs, healthy controls; HVLT, Hopkins verbal learning test; LNS, letter‐number sequencing; MDS‐UPDRS, MDS Unified‐Parkinson Disease Rating Scale; MoCA, montreal cognitive assessment; OR, odds ratio; PDs, patients of Parkinson's disease; *p*‐Tau, phosphorylated tau; SFT, Semantic fluency test; t‐Tau, total tau.

* *p*‐values for continuous variables were from *t*‐test, *p*‐values for dichotomous variable were from chi‐square test, and *p*‐values for ordered categorical data were from Wilcoxon test.

### 3.2 Robustness Test

Linear regression results were generally consistent across population subgroups and covariates. The regression residuals were independent, and the variance inflation factors (VIF) were all < 10 and close to 1. These indicated the robustness of the regression models used in our study.

### 3.3 LILRB4 Genotypes and PD

**FIGURE 2:**
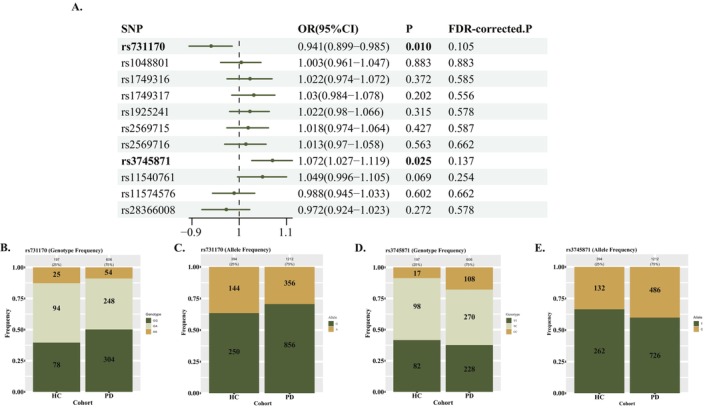
The correlation between *LILRB4* loci and PD risk. (A) The correlation between *LILRB4* loci and PD. (B) Rs731170 genotype frequency. (C) rs731170 allele frequency. (D) Rs3745871 genotype frequency. (E) Rs3745871 allele frequency. FDR, false discovery rate; HC, health control; PD, Parkinson's disease.

First, we analyzed the distribution of genotypes and alleles in HCs and PDs using Χ^2^ test. Three LILRB4 loci had significant differences in genotypes and allele frequencies in HCs and PDs. The rs731170 mutation was negatively associated with PD (genotype GA, OR = 0.677, 95% CI = 0.480–0.955, *p* = 0.026; genotype AA, OR = 0.554, 95% CI = 0.324–0.947, *p* = 0.029; allele A, OR = 0.722, 95% CI = 0.568–0.917, *p* = 0.008), while rs3745871 (genotype CC, OR = 2.285, 95% CI = 1.292–4.041, *p* = 0.004; allele C, OR = 1.329, 95% CI = 1.047–1.687, *p* = 0.019) and rs1749317 (genotype AG, OR = 1.659, 95% CI = 1.168–2.357, *p* = 0.004) were positively associated with PD (Tables S2–S4). Besides, in logistic regression models, similar results were shown (rs731170, OR = 0.941, 95% CI = 0.899–0.985, *p* = 0.010, *P*_c_ = 0.105; rs3745871, OR = 1.072, 95% CI = 1.027–1.119, *p* = 0.025, *P*_c_ = 0.137); the significance of results disappeared after correction (Figure 2, Tables S5–S10).

### 3.4 Brain Images With LILRB4 Genotypes

**FIGURE 3:**
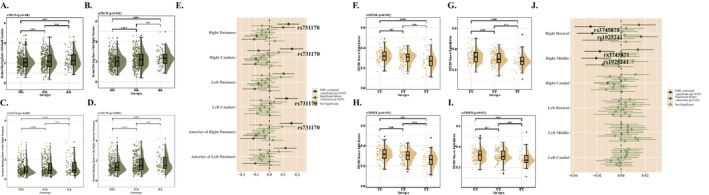
Brain images with *LILRB4* genotypes. (A) Rs731170 was associated with the striatal binding ratios in left caudate. (B) Rs731170 was associated with the striatal binding ratios in right caudate. (C) Rs731170 was associated with the striatal binding ratios in right putamen. (D) Rs731170 was associated with the striatal binding ratios in right anterior putamen. (E) The correlation between *LILRB4* loci and DAT‐SPECT striatal binding ratios. (F) Rs1925241 was associated with FA value in right rostral of SN. (G) Rs1925241 was associated with FA value in right middle of SN. (H) Rs3745871 was associated with FA value in right rostral of SN. (I) Rs3745871 was associated with FA value in right middle of SN. FDR, false discovery rate. (J) The correlation between *LILRB4* loci and DTI FA values.

In Model 1, for SBR from DAT‐SPECT, a significant association between SBR and rs731170 genotypes was detected, especially at right striatal (right caudate, *β* = 0.160, 95% CI = 0.076–0.244, *P*_c_ = 0.002; right putamen, *β* = 0.135, 95% CI = 0.057–0.214, *P*_c_ = 0.009; anterior of right putamen, *β* = 0.156, 95% CI = 0.073–0.240, *P*_c_ = 0.003, and left caudate, *β* = 0.121, 95% CI = 0.038–0.205, *P*_c_ = 0.048) (Figure 3A–F). In the female cohort, we got results similar to the above (right caudate, *β* = 0.246, 95% CI = 0.099–0.392, *P*_c_ = 0.013), while in males, it was not significant enough (right caudate, *p* = 0.036, *P*_c_ = 0.396) (Tables S11–S13). The results in Model 2 were consistent with these (Tables S14–S16).

To illustrate the possible association between WM microstructural change and LILRB4, we integrated DTI as an important factor. In Model 1 we found that a lower FA value would associate with *LILRB4* in the right SN (right rostral of SN, *β* = −0.024, 95% CI = −0.037 to 0.011, *P*_c_ = 0.002; right middle of SN, *β* = −0.021, 95% CI = −0.033 to 0.009, *P*_c_ = 0.012) (Figure 3G–L). And both female (right middle of SN, *β* = −0.025, 95% CI = −0.04 to 0.01, *P*_c_ = 0.021) and male (right rostral of SN, *β* = −0.025, 95% CI = −0.041 to 0.008, *P*_c_ = 0.021) had results as the same (Tables S17–S19). These results were still significant in Model 2 (Tables S20–S22).

### 3.5 CSF Biomarkers With LILRB4 Genotypes

**FIGURE 4:**
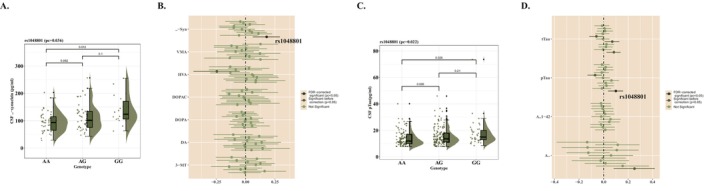
CSF biomarkers with *LILRB4* genotypes. (A) Rs1048801 was associated with the level of CSF α‐synuclein in female. (B) The correlation between *LILRB4* loci and CSF dopaminergic neuron‐related biomarkers in female. (C) rs1048801 was associated with the level of CSF *p*‐Tau in female. (D) The correlation between *LILRB4* loci and CSF Aβ and Tau in female. 3‐MT, 3‐Methoxytyramine; Aβ, beta amyloid; Aβ1‐42, beta amyloid 1–42; DA, dopamine; DOPA, dihydroxyphenylalanine; DOPAC, dihydroxyphenylacetic acid; FDR, false discovery rate; HVA, homovanillic acid; VMA, Vanillymandelic Acid.

At first, we compared the level of CSF α‐synuclein in different *LILRB4* genotypes in multiple linear regression and observed a decrease of CSF α‐synuclein in females (Model 1, *β* = 0.177, 95% CI = 0.062–0.292, *P*_c_ = 0.036; Model 2, *β* = 0.186, 95% CI = 0.071–0.301, *P*_c_ = 0.022) (Figure 4A–C).

Besides, we analyzed the concentration of CSF Aβ, t‐tau, and *p*‐tau. According to our results in Model 1, all the significant associations only appeared in the female cohort that *LILRB4* related to the increase of *p*‐tau (*β* = 0.097, 95% CI = 0.036–0.159, *P*_c_ = 0.022) (Figure 4D–F) and possible increases of CSF Aβ (*β* = 0.250, 95% CI = 0.082–0.417, *p* = 0.007, *P*_c_ = 0.072) and *t*‐tau (*β* = 0.081, 95% CI = 0.024–0.139, *p* = 0.006, *P*_c_ = 0.064). These results still existed in Model 2.

Dopamine (DA) and its metabolites were also involved in our analysis. There was a possible increase of DA (Model 1, *β* = 0.206, 95% CI = 0.032–0.381, *p* = 0.022, *P*_c_ = 0.242) and a decrease of homovanillic acid (HVA) (Model 1, *β* = −0.247, 95% CI = −0.443–0.050, *p* = 0.018, *P*_c_ = 0.196; Model 2, *β* = −0.265, 95% CI = −0.465–0.064, *p* = 0.013, *P*_c_ = 0.143). However, the significance of results disappeared after correction.

Also, S100B displayed possible association with *LILRB4* in female (Model 1, *β* = 0.107, 95% CI = 0.030–0.184, *p* = 0.008, *P*_c_ = 0.085; Model 2, *β* = 0.113, 95% CI = 0.036–0.190, *p* = 0.005, *P*_c_ = 0.057) (Figure S2, Tables S23–S28).

### 3.6 Motor and Nonmotor Function With LILRB4 Genotypes

**FIGURE 5:**
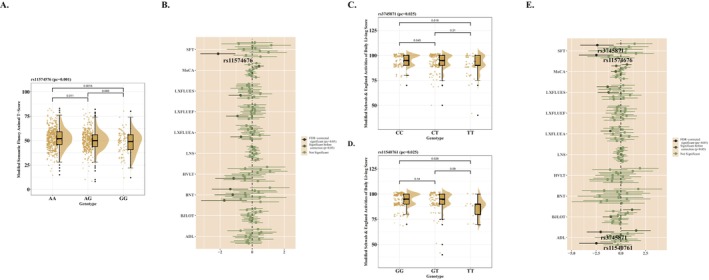
MDS‐UPDRS and nonmotor function scales with LILRB4 genotypes. (A) Rs11574576 was associated with the score of Modifed Semantic Fluncy test. (B) The correlations between *LILRB4* loci and nonmotor function scales. (C) rs3745871 was associated with the score of SFT. (D) rs11540761 was associated with the score of SFT. (E) The correlations between *LILRB4* loci and nonmotor function scales in female. ADL, modified Schwab & England activities of daily living test; BJLOT, Benton judgment of line orientation; BNT, Modified Boston Naming Test; FDR, false discovery rate; HVLT, Hopkins verbal learning test; LNS, letter‐number sequencing test; LXFLUEA, lexical fluency‐A; LXFLUEF, lexical fluency‐F; LXFLUES, lexical fluency‐S; MoCA, Montreal cognitive assessment; SFT, semantic fluency test.

There were no significant associations found among any scales of motor function neither in Model 1 or Model 2 (Figure S3). However, for nonmotor function, we noticed some conspicuous results which majorly concern cognitive impairment, such as the worse semantic fluency (Model 1, *β* = −2.135, 95% CI = −3.225 to 1.046, *P*_c_ = 0.001; Model 2, *β* = −2.107, 95% CI = −3.198 to 1.017, *P*_c_ = 0.002). Besides, in females, the impairment of cognitive function was more prominently displayed not only in semantic fluency (Model 1, *β* = −2.508, 95% CI = −4.255 to 0.761, *P*_c_ = 0.032; Model 2, *β* = −2.619, 95% CI = −4.373 to 0.864, *P*_c_ = 0.04) but also in assessments of daily living functions (Model 1, *β* = −2.073, 95% CI = −3.446 to 0.699, *P*_c_ = 0.025; Model 2, *β* = −2.007, 95% CI = −3.380–0.635, *P*_c_ = 0.025) (Figure 5A–G, Table S29–S34). Interestingly, after excluding the effect of educational attainment on the assessment of cognitive performance in Model 3, all of the significances mentioned above disappeared and were replaced by worse assessments in BJLOT (all, *β* = −2.618, 95% CI = −4.020 to 1.216, *P*_c_ = 0.008; female, β = −3.593, 95% CI = −5.588 to 1.599, *P*_c_ = 0.03) (Table S29–S34).

## 4 Discussion

In our study, we investigated the associations between *LILRB4* and a series of hallmarks and clinical phenotypes in a cohort of patients with PD, including CSF biomarkers and neuroimaging features. Our study innovatively identified the correlation between *LILRB4* loci (rs731170 and rs3745871) and the risk of developing PD. We found that *LILRB4* could be associated with decreased CSF α‐synuclein levels, improved DAT function in the right striatum, and increased DA and its metabolites in CSF. Additionally, *LILRB4* was also linked to WM microstructural alterations in the SN, positively correlating with the classical AD hallmarks (Aβ, t‐tau and *p*‐tau). Consistent with the pathological changes, *LILRB4* appeared to worsen cognitive performance without influencing motor symptoms. Furthermore, in the gender‐specific analysis, we observed that these correlations differed by gender. Specifically, *LILRB4* was associated with better dopaminergic neuron function but poorer cognitive capacity in women, which aligns with clinically observed sex differences for these phenotypes.

Several studies have evidenced that *LILRB4* is upregulated in neurodegenerative diseases [9, 10, 11], but the effects of *LILRB4* loci on neurodegenerative diseases had not been previously investigated. Our study found that *LILRB4* rs731170 and rs3745871 were weakly associated with PD. Interestingly, their effects on PD differed; rs731170 might be a protective factor, while rs3745871 might be a risk factor. Moreover, these two loci are located at exon4 and exon5, respectively (Table 1), suggesting that they may influence PD by altering the functional structure of LILRB4. Specifically, rs731170 was protective for DAT function, whereas rs3745871 was associated with increased WM damage in the brain, reflecting the dual effects of LILRB4 on PD.

Massive α‐syn deposition and consequent damage to nigrostriatal dopaminergic neurons are the predominant pathologic features in PD, playing important roles in motor control functions and motor skill learning [21]. As a potential diagnostic indicator, CSF α‐synuclein has been shown to be reduced in PD [22]. In the present study, we found that *LILRB4* was significantly correlated with a higher level of CSF α‐synuclein and was associated with improved striatal DAT function, particularly in the right caudate and putamen. In addition to indicators related to dopaminergic neuron damage, we also focused on CSF DA and its metabolites. Our analyses across different cohorts consistently indicated that *LILRB4* was slightly associated with increased CSF DA and its metabolite HVA. Previous studies have demonstrated that DA and its metabolites are often reduced in PD [23]. Recent studies have shown that CSF HVA levels in PD patients increase with the severity of motor dysfunction [24]. Consistently, in ALS (a neurodegenerative disease primarily characterized by motor dysfunction), upregulation of *LILRB4* expression was observed during disease recovery, suggesting a potential neuroprotective role of *LILRB4* [10]. Thus, *LILRB4* might exert a beneficial effect on the PD pathology, but further research is needed to elucidate the exact mechanism.

Previous studies have investigated that WM microstructural alteration in the SN can be observed in PD [25, 26]. Meanwhile, existing evidence indicates that WM damage is macroscopically and microscopically related to cognitive decline [27], executive dysfunction, and language impairment in PD [28]. Our analyses revealed that *LILRB4* was linked to WM microstructural alterations, especially in the right SN. This finding highlighted the possible role of *LILRB4* in WM microstructural changes and cognitive impairment in PD. Similarly, elevated LILRB4 expression has been detected in cerebral WM demyelinating lesions in patients with multiple sclerosis (MS) [11]. Therefore, further studies are warranted to validate and explore the mechanisms by which *LILRB4* may contribute to cognitive impairment in PD.

As is well known, the level of CSF Aβ and tau is an important neuropathological hallmark of AD [29, 30]. These hallmarks have also been extensively studied in PD, where their increase would lead to worse cognitive performance [31, 32]. Substantial evidence suggests that CSF Aβ and tau accumulation may accelerate α‐synuclein aggregation and propagation in PD [5, 33]. In our study, we found that *LILRB4* was associated with increased CSF Aβ and tau levels, consistent with previous research findings. Recent studies have demonstrated that *LILRB4* is expressed in microglia surrounding Aβ plaques in AD models, and that reducing *LILRB4* expression through anti‐human LILRB4 monoclonal antibody (mAb) treatment decreases Aβ load and ameliorates some Aβ‐related behavioral abnormalities [14]. Thus, we hypothesize that *LILRB4* might facilitate PD cognitive impairment in PD by promoting Aβ accumulation. Our findings in females further confirmed this hypothesis, showing that *LILRB4* exacerbates PD‐related cognitive impairment through accumulation of both Aβ and tau.

CSF GFAP and S100B, as the markers of astrocyte activation, are significantly elevated in neurodegenerative diseases and contribute to neuroinflammation [34, 35]. Considerable evidence indicates that astrocyte activation plays a crucial role in PD progression [36]. Activated astrocytes promote PD pathogenesis through neurotoxic effects [37], and pathological α‐synuclein in turn stimulates astrocyte activation [38]. Besides, CSF GFAP and S100B levels could serve as predictors of cognitive impairment [39] and would decline with cognitive ability in PD patients [40]. Our study systematically analyzed the relationship between *LILRB4* and astrocytic‐associated biomarkers in CSF, clearly demonstrating that *LILRB4* is positively associated with higher CSF S100B levels. These findings suggest that *LILRB4* may worsen cognitive dysfunction in PD, at least partially, by modulating astrocyte function and activity.

We observed that some associations lost significance after FDR‐correction, particularly when *GBA* and *LRRK2* were included as covariates. Mutations in *GBA* and *LRRK2* are well‐established risk factors for PD and are associated with significant pathological changes [41]. Studies have shown more significant dopaminergic neuronal damage and lower striatal DA and its metabolite levels in a PD mouse model with heterozygous knock‐in *GBA* L444P mutations compared to wild‐type PD mice [42]. Furthermore, research has identified upregulation of CSF DOPA decarboxylase in *GBA* or *LRRK2* mutation carriers [43]. These findings suggest that *GBA* and *LRRK2* mutations might confound the observed associations between *LILRB4* and DA and its metabolites.

In terms of potential molecular mechanisms, *LILRB4* is an inhibitory receptor in the *LILR* family. Previous studies have confirmed that *LILRB4* transmits signals by activating pathways such as the TGFβ1 signaling pathway [44]. For example, a human clinical trial demonstrated that TGFβ1 restores mitochondrial function in dopaminergic neurons by improving immunoregulation and autophagic homeostasis in microglia [45]. Additionally, TGFβ1, a key regulatory molecule of astrocyte function and differentiation, is involved in synaptic development and remodeling by astrocytes, which might elucidate its potential role in human cognitive function [46]. Moreover, AD models have shown that in aging organisms, upregulation of TGFβ1 is not exclusively neuroprotective but rather tends to promote inflammation and impair Aβ clearance, thereby affecting cognitive capacity [47, 48]. The association of TGFβ1 dysregulation with pathologic tau proteins further supports the impact of TGFβ1 on intellectual function. Therefore, we hypothesize that LILRB4 might protect dopaminergic neurons and influence cognitive function by activating TGFβ1.

Additionally, LILRB4 is also involved in other inflammation‐related pathways through activation of STAT3 or inhibition of NF‐κB [49]. Different activation states of microglia are associated with neuroinflammation and neurodegenerative diseases [50]. NF‐κB promotes microglia differentiation toward the M1 phenotype, while STAT3 enhances the expression of M2 markers [51]. Animal experiments have further validated that STAT3 and NF‐κB pathways regulate neuroinflammation and dopaminergic neuronal degeneration [52]. Besides, STAT3 would promote neurite outgrowth and synapse formation in neurons after injury [53]. However, the role of NF‐κB in cognition remains unclear. On the one hand, the NF‐κB signaling pathway has been reported to maintain synaptic plasticity and support cognitive function [54]; on the other hand, evidence suggests that NF‐κB may induce Aβ plaque accumulation and tau hyperphosphorylation [55]. In summary, we propose that STAT3 and NF‐κB are potential mechanisms through which LILRB4 regulates dopaminergic neuron function and influences cognitive performance.

Given the sex differences in PD risk, our sex‐stratified analysis revealed that the *LILRB4* gene exerts female‐specific effects on DAT functional improvement and cognitive decline, with no significant association observed in males. Notably, prior studies have not established a link between *LILRB4* and PD pathology in females. First, we considered sex‐biased *LILRB4* variant distribution as an explanation, but genetic analyses showed no sex‐specific differences (Table S38), indicating that sex‐related pathophysiological mechanisms predominate rather than sex‐biased *LILRB4* expression variations. Second, existing evidence highlights that sex disparities in PD DAT functionality correlate with heterogeneity in the nigrostriatal circuit: female patients exhibit significantly higher DAT binding affinity and density than males [56, 57]. Combined with estrogen's neuroprotective roles in inhibiting pathological α‐syn aggregation [58] and enhancing dopaminergic neuron survival [59], *LILRB4*'s sex‐specific effects likely arise from inherent dopaminergic system differences synergized with estrogen regulation. Last, further analyses demonstrated the *LILRB4*‐cognitive impairment link in females may involve compounded WMH pathology and sex‐specific glial activation patterns. For example, brain MRI revealed age‐dependent severity of WMH in cognitively impaired females [60]. Meanwhile, Females exhibit heightened susceptibility in neuroimmune‐synaptic regulation, manifested by elevated baseline microglial immune priming, amplified neuroinflammatory responses, and concomitant hyperactivation [61, 62]. In summary, this study uncovers *LILRB4*'s dual role in female PD patients: its variants may delay motor symptom progression by preserving DAT function but exacerbate cognitive deficits, underscoring the complexity of sex‐specific pathological mechanisms.

## 5 Conclusion

Our study supposed that *LILRB4* may play a crucial role in modulating PD clinical characteristics by regulating nigrostriatal dopaminergic neuron function, AD‐related pathology, WM microstructural alterations, and astrocyte activation. On the one hand, *LILRB4* appears to exert beneficial effects on the nigrostriatal dopamine system, including reduced α‐synuclein deposition, improved DAT function, and enhanced DA and its metabolite, which could delay the onset of motor dysfunction in PD. On the other hand, it might also contribute to cognitive deterioration in PD through WM microstructural changes in the SN, abnormal accumulation of Aβ and tau, and astrocyte over‐activation. Notably, these effects demonstrate stronger associations in females. These findings further support the hypothesis that *LILRB4* genetic variation could serve as potential biomarkers for PD. Larger clinical studies involving diverse ethnic populations would provide more robust evidence to clarify the relevance of *LILRB4* to PD and its phenotypes. Moreover, investigations of relevant molecule pathways in animal models would also help elucidate the underlying mechanisms in greater depth.

## Author Contributions

**Yuting Zhou:** conceptualization, methodology, data curation, formal analysis, validation, writing‐original draft, writing‐review and editing, visualization. **Yaqing Li:** conceptualization, methodology, validation, writing – review and editing, funding acquisition. **Qiqing He:** data curation, validation, writing – review and editing. **Zhen Kong:** data curation, writing – review and editing. **Ran Yu:** data curation. **Xin Yu:** supervision, project administration. **Anmu Xie:** supervision, project administration, funding acquisition.

## Conflicts of Interest

The authors declare no conflicts of interest.

## Supporting information


**Figure S1.** Test for normality of continuous variables. (A) DAT‐SPECT striatal binding ratios; (B) DTI FA value; (C) CSF biomarkers. 3‐MT, 3‐Methoxytyrosine; DOPA, 3,4‐Dihydroxymandelic acid; DOPAC, 3,4‐Dihydroxyphenylacetic acid; GFAP, glial fibrillary acid protein; HVA, homovanillic acid; IL‐1b, Interleukin 1b; IL‐6, Interleukin 6; NFL, neurofilament light; S100, S‐100 calcium binding protein B; sTREM2, soluble triggering receptor expressed on myeloid cells 2; VMA, Vanillymandelic Acid; YKL40, chitinase‐3‐like protein 1.

**Figure S2.** The significances of correlation between *LILRB4* loci and CSF biomarkers. 3‐MT, 3‐Methoxytyrosine; DA, Dopamine; DOPA, 3,4‐Dihydroxymandelic acid; DOPAC, 3,4‐Dihydroxyphenylacetic acid; GFAP, glial fibrillary acid protein; HVA, homovanillic acid; IL‐1b, Interleukin 1b; IL‐6, Interleukin 6; NFL, neurofilament light; S100B, S‐100 calcium binding protein B; sTREM2, soluble triggering receptor expressed on myeloid cells 2; VMA, Vanillymandelic Acid; YKL40, chitinase‐3‐like protein 1.

**Figure S3.** The significances of correlations between LILRB4 loci and MDS‐UPDRS. MDS‐UPDRS, Movement disorder society‐sponsored revision of the Unified Parkinson’s Disease Rating Scale; P, patient questionnaire; R, rater completed.

Table S1.

## Data Availability

The data that support the findings of this study are available from the corresponding author upon reasonable request.
