# Supplementary figures and images for: Uncovering the Associations of LILRB4 Genotypes With Parkinson's Disease: From Clinical Traits to Potential Pathologies
Source: CNS Neurosci Ther. 2025 Jul 23;31(7):e70522. doi: 10.1111/cns.70522 (PMC12287542; doi:10.1111/cns.70522)

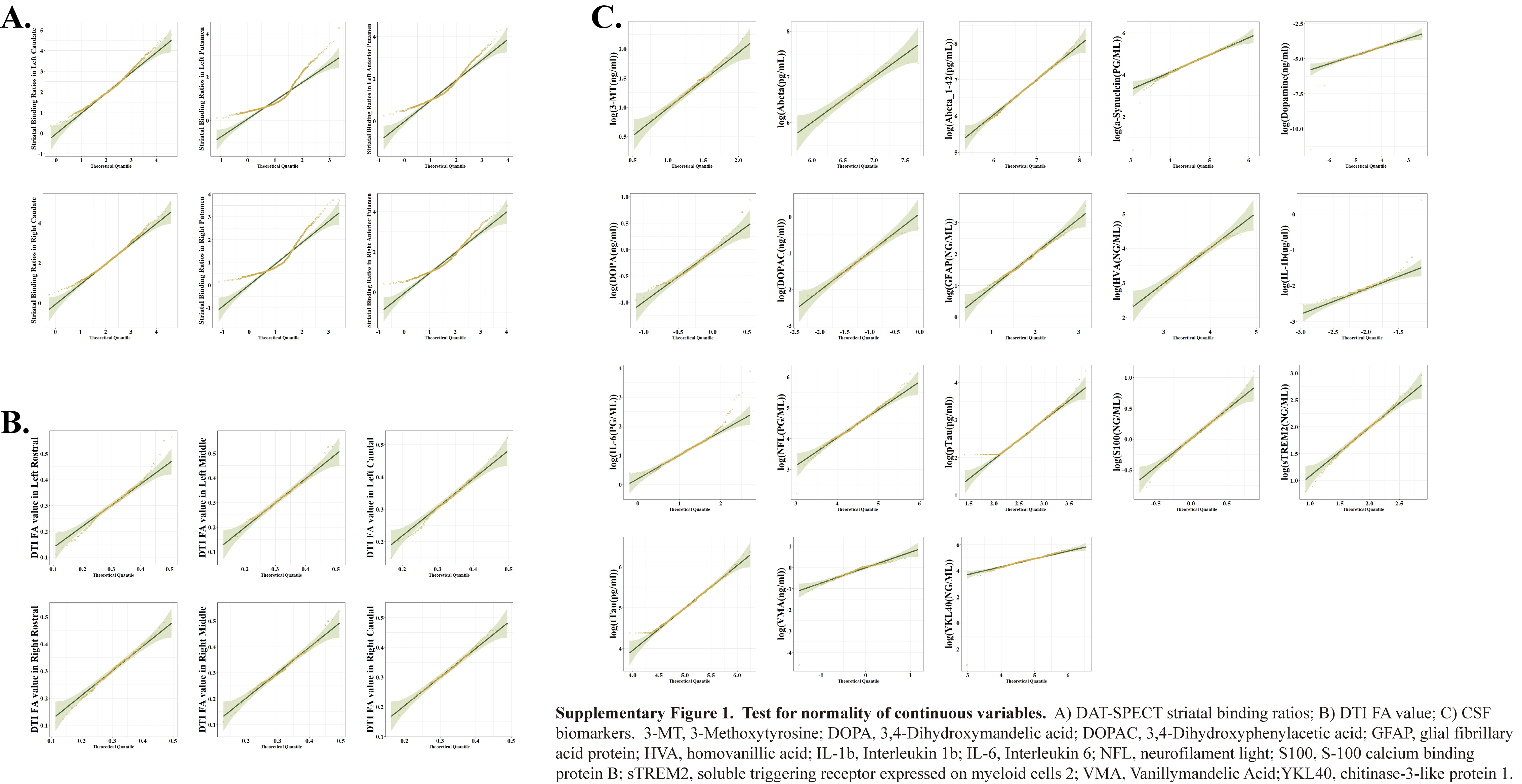

Supplement: Supplementary file 1 — Figure S1. Test for normality of continuous variables. (A) DAT‐SPECT striatal binding ratios; (B) DTI FA value; (C) CSF biomarkers. 3‐MT, 3‐Methoxytyrosine; DOPA, 3,4‐Dihydroxymandelic acid; DOPAC, 3,4‐Dihydroxyphenylacetic acid; GFAP, glial fibrillary acid protein; HVA, homovanillic acid; IL‐1b, Interleukin 1b; IL‐6, Interleukin 6; NFL, neurofilament light; S100, S‐100 calcium binding protein B; sTREM2, soluble triggering receptor expressed on myeloid cells 2; VMA, Vanillymandelic Acid; YKL40, chitinase‐3‐like protein 1. [file CNS-31-e70522-s004.tif]

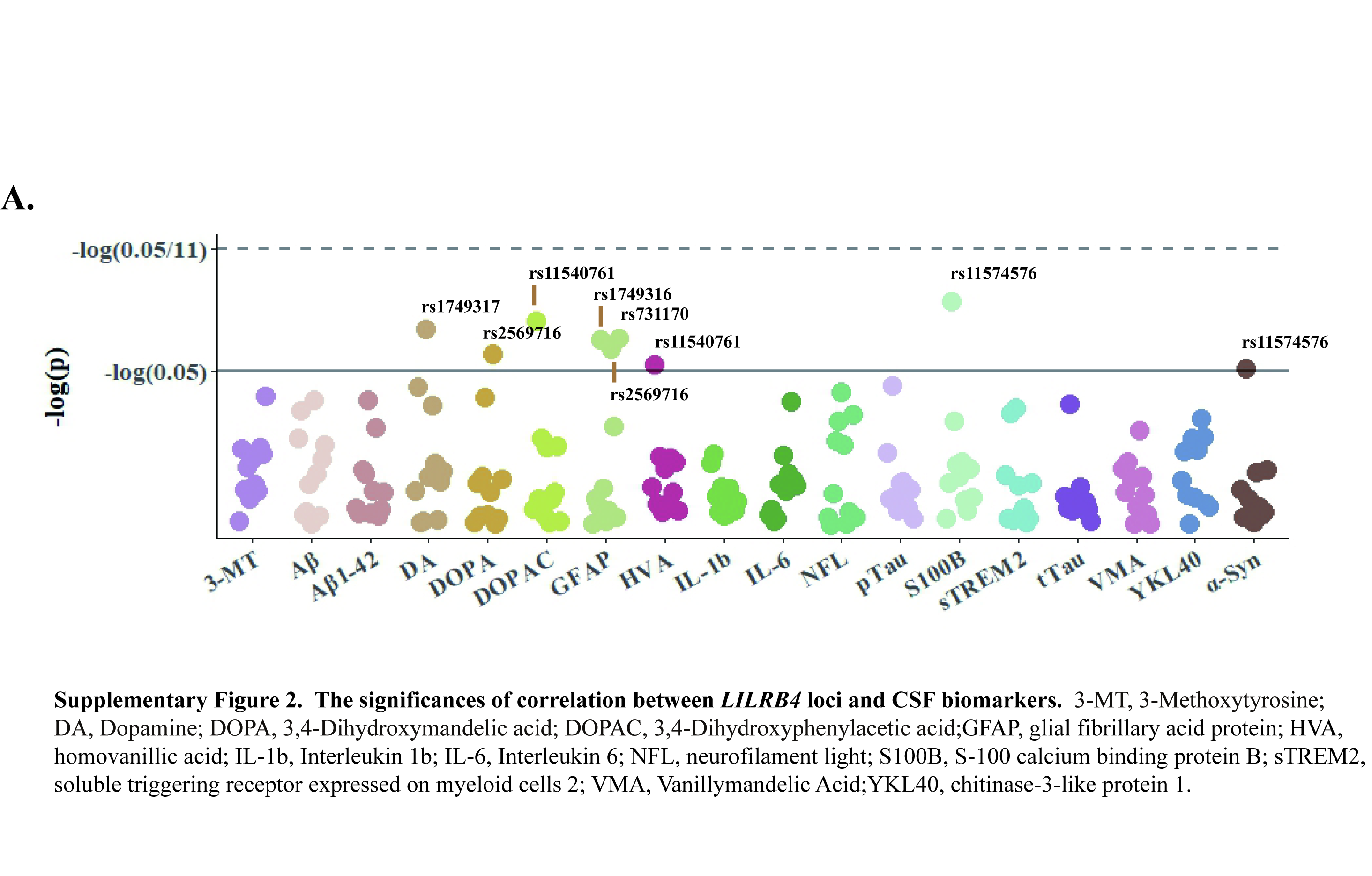

Supplement: Supplementary file 2 — Figure S2. The significances of correlation between LILRB4 loci and CSF biomarkers. 3‐MT, 3‐Methoxytyrosine; DA, Dopamine; DOPA, 3,4‐Dihydroxymandelic acid; DOPAC, 3,4‐Dihydroxyphenylacetic acid; GFAP, glial fibrillary acid protein; HVA, homovanillic acid; IL‐1b, Interleukin 1b; IL‐6, Interleukin 6; NFL, neurofilament light; S100B, S‐100 calcium binding protein B; sTREM2, soluble triggering receptor expressed on myeloid cells 2; VMA, Vanillymandelic Acid; YKL40, chitinase‐3‐like protein 1. [file CNS-31-e70522-s003.tif]

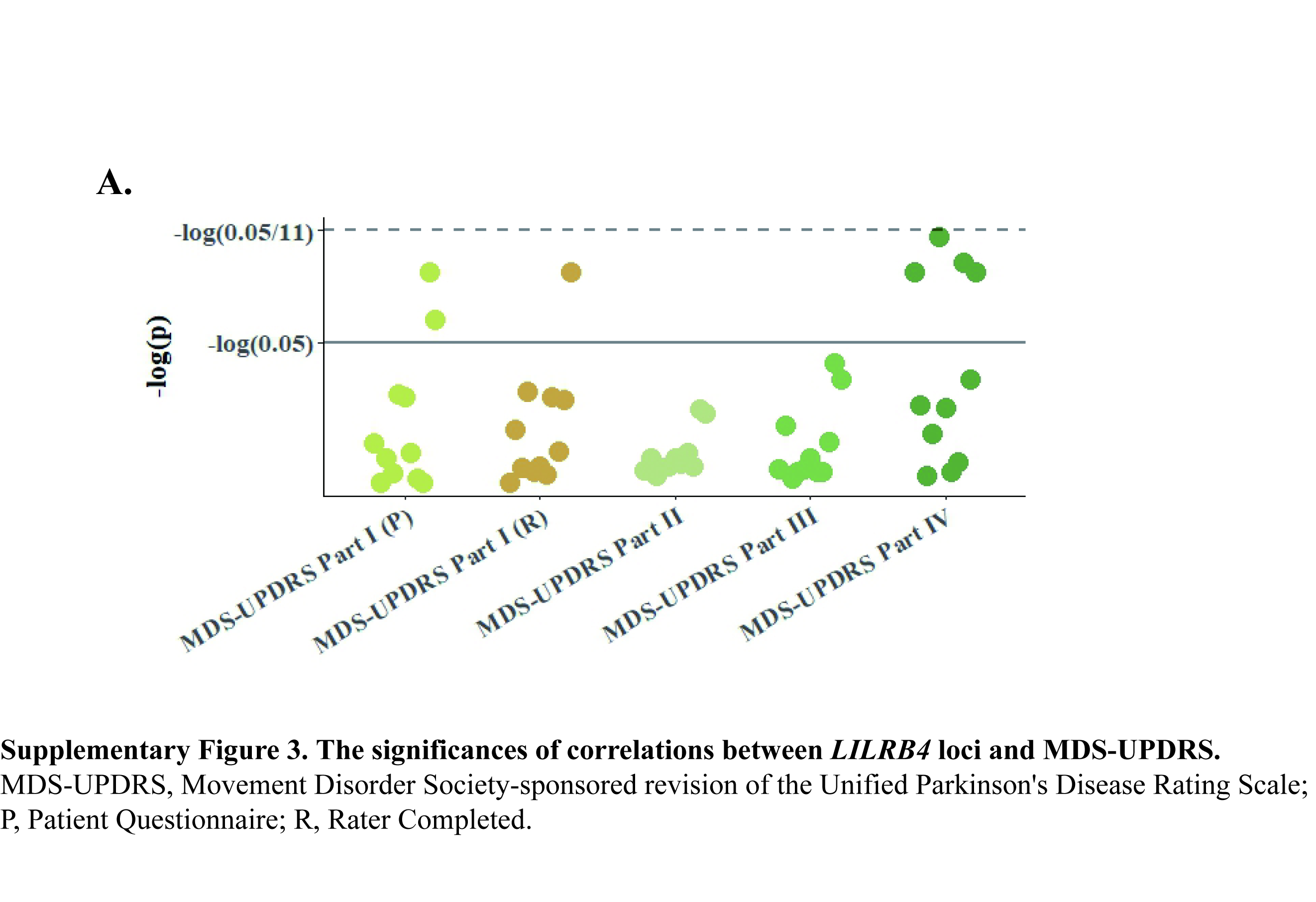

Supplement: Supplementary file 3 — Figure S3. The significances of correlations between LILRB4 loci and MDS‐UPDRS. MDS‐UPDRS, Movement disorder society‐sponsored revision of the Unified Parkinson’s Disease Rating Scale; P, patient questionnaire; R, rater completed. [file CNS-31-e70522-s001.tif]
