# Supplementary material for: Uncovering the Associations of LILRB4 Genotypes With Parkinson's Disease: From Clinical Traits to Potential Pathologies
Source: CNS Neurosci Ther. 2025 Jul 23;31(7):e70522. doi: 10.1111/cns.70522 (PMC12287542; doi:10.1111/cns.70522)
Supplement: Supplementary file 4 — Table S1. [file CNS-31-e70522-s002.zip › cns70522-sup-0004-TableS1@Supplementary Table 1 The correlations between PD phenotypes and potential factors.docx]

**Supplementary Table 1. The correlations between PD phenotypes and potential factors.**

| Factors | Phenotypes | | β(95%CI) | P value |
| --- | --- | --- | --- | --- |
| AGE | COHORT | | 0.883(-0.771-2.537) | 0.296 |
|  | CSF | 3-MT(ng/ml) | 0.005(0.000-0.01) | 0.051 |
|  |  | 4-Hydroxy-3-methoxymandelic acid (VMA)(ng/ml) | 0.007(-0.001-0.015) | 0.091 |
|  |  | α-Synuclein(PG/ML) | 0.008(0.002-0.014) | **0.007** |
|  |  | Aβ(pg/mL) | 0.000(-0.01-0.009) | 0.930 |
|  |  | Aβ_1-42(pg/mL) | 0.001(-0.003-0.004) | 0.680 |
|  |  | DOPA(ng/ml) | -0.002(-0.007-0.003) | 0.473 |
|  |  | DOPAC(ng/ml) | 0.000(-0.007-0.008) | 0.899 |
|  |  | Dopamine(ng/ml) | 0.021(0.008-0.033) | **0.001** |
|  |  | GFAP(NG/ML) | 0.018(0.013-0.022) | **＜0.001** |
|  |  | Homovanillic acid (HVA)(ng/ml) | 0.005(-0.003-0.012) | 0.258 |
|  |  | IL-1b(ug/ul) | 0.004(-0.003-0.01) | 0.308 |
|  |  | IL-6(PG/ML) | 0.004(-0.002-0.009) | 0.193 |
|  |  | NFL(PG/ML) | 0.03(0.026-0.034) | **＜0.001** |
|  |  | pTau (pg/ml) | 0.011(0.008-0.014) | **＜0.001** |
|  |  | S100B(NG/ML) | 0.000(-0.003-0.003) | 0.979 |
|  |  | sTREM2(NG/ML) | 0.011(0.008-0.014) | **＜0.001** |
|  |  | tTau(pg/ml) | 0.01(0.008-0.013) | **＜0.001** |
|  |  | YKL40(NG/ML) | 0.019(0.013-0.025) | **＜0.001** |
|  | DAT | Left Caudate | -0.011(-0.017--0.006) | **＜0.001** |
|  |  | Right Caudate | -0.009(-0.015--0.004) | **0.001** |
|  |  | Left Putamen | -0.007(-0.012--0.002) | **0.006** |
|  |  | Left Anterior of Putamen | -0.007(-0.013--0.002) | **0.008** |
|  |  | Right Putamen | -0.007(-0.013--0.002) | **0.004** |
|  |  | Right Anterior of Putamen | -0.007(-0.013--0.002) | **0.011** |
|  | DTI | Left Rostral | 0.000(-0.001-0.001) | 0.589 |
|  |  | Left Middle | 0.000(-0.001-0.001) | 0.924 |
|  |  | Left Caudal | 0.000(-0.001-0.001) | 0.743 |
|  |  | Right Rostral | 0.000(-0.001-0.001) | 0.905 |
|  |  | Right Middle | -0.001(-0.002-0.000) | 0.085 |
|  |  | Right Caudal | -0.001(-0.002-0.000) | **0.009** |
|  | SCALEs | Benton Judgement of Line Orientation | -0.055(-0.087--0.023) | **0.001** |
|  |  | Letter-Number Sequencing Test | -0.009(-0.029-0.011) | 0.374 |
|  |  | Hopkins Verbal Learning Test | 0.086(0.011-0.160) | **0.025** |
|  |  | Lexical Fluency-A | -0.068(-0.112--0.025) | **0.002** |
|  |  | Lexical Fluency-F | -0.081(-0.128--0.034) | **0.001** |
|  |  | Lexical Fluency-S | -0.070(-0.119--0.021) | **0.005** |
|  |  | MDS-UPDRS Part I (Rater Completed) | -0.008(-0.020-0.005) | 0.229 |
|  |  | MDS-UPDRS Part I (Patient Questionnaire) | 0.033(0.009-0.057) | **0.007** |
|  |  | MDS-UPDRS Part II | 0.027(-0.007-0.060) | 0.121 |
|  |  | MDS-UPDRS Part III | 0.155(0.074-0.235) | **＜0.001** |
|  |  | MDS-UPDRS Part IV | -0.050(-0.075--0.025) | **＜0.001** |
|  |  | Modified Boston Naming Test | 0.038(-0.047-0.124) | 0.380 |
|  |  | Modified Schwab & England Activities of Daily Living | -0.022(-0.079-0.034) | 0.439 |
|  |  | Modified Semantic Fluency | 0.122(0.050-0.194) | **0.001** |
|  |  | Montreal Cognitive Assessment | -0.038(-0.057--0.020) | **＜0.001** |
| SEX | COHORT | | -0.042(-0.121-0.036) | 0.290 |
|  | CSF | 3-MT(ng/ml) | -0.068(-0.173-0.036) | 0.203 |
|  |  | 4-Hydroxy-3-methoxymandelic acid (VMA)(ng/ml) | -0.027(-0.197-0.143) | 0.753 |
|  |  | α-Synuclein(PG/ML) | -0.019(-0.141-0.103) | 0.763 |
|  |  | Aβ(pg/mL) | -0.167(-0.338-0.004) | 0.059 |
|  |  | Aβ_1-42(pg/mL) | -0.032(-0.101-0.036) | 0.355 |
|  |  | DOPA(ng/ml) | 0.104(-0.001-0.209) | 0.053 |
|  |  | DOPAC(ng/ml) | -0.136(-0.286-0.013) | 0.076 |
|  |  | Dopamine(ng/ml) | -0.118(-0.389-0.152) | 0.393 |
|  |  | GFAP(NG/ML) | 0.110(0.000-0.219) | 0.050 |
|  |  | Homovanillic acid (HVA)(ng/ml) | -0.215(-0.378--0.053) | **0.011** |
|  |  | IL-1b(ug/ul) | -0.087(-0.236-0.062) | 0.255 |
|  |  | IL-6(PG/ML) | 0.085(-0.031-0.200) | 0.152 |
|  |  | NFL(PG/ML) | 0.200(0.086-0.315) | **0.001** |
|  |  | pTau (pg/ml) | -0.048(-0.104-0.007) | 0.088 |
|  |  | S100B(NG/ML) | 0.019(-0.045-0.083) | 0.560 |
|  |  | sTREM2(NG/ML) | 0.038(-0.040-0.115) | 0.342 |
|  |  | tTau(pg/ml) | -0.062(-0.115--0.009) | **0.021** |
|  |  | YKL40(NG/ML) | 0.135(-0.005-0.275) | 0.060 |
|  | DAT | Left Caudate | -0.069(-0.184-0.046) | 0.238 |
|  |  | Right Caudate | -0.027(-0.142-0.088) | 0.644 |
|  |  | Left Putamen | -0.003(-0.11-0.104) | 0.957 |
|  |  | Left Anterior of Putamen | -0.024(-0.137-0.089) | 0.674 |
|  |  | Right Putamen | 0.020(-0.088-0.128) | 0.712 |
|  |  | Right Anterior of Putamen | 0.014(-0.100-0.129) | 0.804 |
|  | DTI | Left Rostral | -0.001(-0.021-0.018) | 0.881 |
|  |  | Left Middle | 0.007(-0.012-0.025) | 0.488 |
|  |  | Left Caudal | 0.019(0.003-0.035) | 0.019 |
|  |  | Right Rostral | -0.010(-0.029-0.008) | 0.274 |
|  |  | Right Middle | 0.001(-0.017-0.019) | 0.915 |
|  |  | Right Caudal | 0.005(-0.011-0.021) | 0.566 |
|  | SCALEs | Benton Judgement of Line Orientation | 2.818(2.167-3.468) | **＜0.001** |
|  |  | Letter-Number Sequencing Test | 0.284(-0.131-0.698) | 0.180 |
|  |  | Hopkins Verbal Learning Test | 0.080(-0.817-0.977) | 0.862 |
|  |  | Lexical Fluency-A | 0.150(-0.824-1.123) | 0.764 |
|  |  | Lexical Fluency-F | 0.320(-0.684-1.325) | 0.532 |
|  |  | Lexical Fluency-S | -3.046(-4.597--1.495) | **＜0.001** |
|  |  | MDS-UPDRS Part I (Rater Completed) | -0.461(-0.721--0.201) | **0.001** |
|  |  | MDS-UPDRS Part I (Patient Questionnaire) | -0.935(-1.438--0.433) | **＜0.001** |
|  |  | MDS-UPDRS Part II | -0.232(-0.936-0.473) | 0.519 |
|  |  | MDS-UPDRS Part III | 0.488(-1.229-2.204) | 0.578 |
|  |  | MDS-UPDRS Part IV | 0.147(-0.368-0.662) | 0.576 |
|  |  | Modified Boston Naming Test | 2.336(0.661-4.011) | **0.007** |
|  |  | Modified Schwab & England Activities of Daily Living | 0.385(-0.785-1.555) | 0.519 |
|  |  | Modified Semantic Fluency | -1.226(-2.745-0.293) | 0.114 |
|  |  | Montreal Cognitive Assessment | -0.081(-0.463-0.301) | 0.679 |
| EDUCATION | COHORT | | -1.075(-2.971-0.820) | 0.272 |
|  | CSF | 3-MT(ng/ml) | -0.008(-0.082-0.067) | 0.845 |
|  |  | 4-Hydroxy-3-methoxymandelic acid (VMA)(ng/ml) | 0.023(-0.215-0.262) | 0.852 |
|  |  | α-Synuclein(PG/ML) | -0.001(-0.059-0.057) | 0.964 |
|  |  | Aβ(pg/mL) | 0.112(-0.055-0.279) | 0.319 |
|  |  | Aβ_1-42(pg/mL) | 0.019(-0.019-0.057) | 0.337 |
|  |  | DOPA(ng/ml) | -0.019(-0.071-0.032) | 0.470 |
|  |  | DOPAC(ng/ml) | -0.036(-0.127-0.056) | 0.458 |
|  |  | Dopamine(ng/ml) | -0.080(-0.153--0.008) | **0.045** |
|  |  | GFAP(NG/ML) | -0.014(-0.062-0.034) | 0.576 |
|  |  | Homovanillic acid (HVA)(ng/ml) | -0.016(-0.113-0.081) | 0.752 |
|  |  | IL-1b(ug/ul) | 0.001(-0.044-0.046) | 0.955 |
|  |  | IL-6(PG/ML) | 0.020(-0.043-0.082) | 0.540 |
|  |  | NFL(PG/ML) | 0.015(-0.058-0.089) | 0.687 |
|  |  | pTau (pg/ml) | 0.017(-0.014-0.049) | 0.281 |
|  |  | S100B(NG/ML) | -0.010(-0.040-0.020) | 0.528 |
|  |  | sTREM2(NG/ML) | -0.016(-0.063-0.030) | 0.499 |
|  |  | tTau(pg/ml) | 0.018(-0.014-0.049) | 0.275 |
|  |  | YKL40(NG/ML) | -0.017(-0.08-0.045) | 0.596 |
|  | DAT | Left Caudate | 0.046(-0.026-0.118) | 0.218 |
|  |  | Right Caudate | 0.047(-0.024-0.118) | 0.202 |
|  |  | Left Putamen | 0.056(-0.025-0.136) | 0.185 |
|  |  | Left Anterior of Putamen | 0.052(-0.027-0.132) | 0.203 |
|  |  | Right Putamen | 0.061(-0.009-0.131) | 0.097 |
|  |  | Right Anterior of Putamen | 0.064(-0.008-0.136) | 0.090 |
|  | DTI | Left Rostral | -0.009(-0.021-0.004) | 0.196 |
|  |  | Left Middle | -0.003(-0.014-0.009) | 0.669 |
|  |  | Left Caudal | -0.004(-0.016-0.007) | 0.462 |
|  |  | Right Rostral | 0.006(-0.010-0.023) | 0.483 |
|  |  | Right Middle | 0.003(-0.015-0.022) | 0.736 |
|  |  | Right Caudal | -0.009(-0.026-0.008) | 0.318 |
|  | SCALEs | Benton Judgement of Line Orientation | 0.308(-0.052-0.668) | 0.100 |
|  |  | Letter-Number Sequencing Test | 0.218(-0.014-0.45) | 0.072 |
|  |  | Hopkins Verbal Learning Test | 0.717(0.239-1.195) | **0.005** |
|  |  | Lexical Fluency-A | 0.510(0.121-0.898) | **0.014** |
|  |  | Lexical Fluency-F | 0.273(-0.208-0.754) | 0.272 |
|  |  | Lexical Fluency-S | 1.199(0.504-1.895) | **0.002** |
|  |  | MDS-UPDRS Part I (Rater Completed) | 0.057(-0.089-0.203) | 0.448 |
|  |  | MDS-UPDRS Part I (Patient Questionnaire) | -0.049(-0.31-0.212) | 0.714 |
|  |  | MDS-UPDRS Part II | -0.014(-0.390-0.363) | 0.944 |
|  |  | MDS-UPDRS Part III | -0.248(-1.090-0.594) | 0.567 |
|  |  | MDS-UPDRS Part IV | 0.047(-0.275-0.369) | 0.777 |
|  |  | Modified Boston Naming Test | 0.709(-0.435-1.853) | 0.232 |
|  |  | Modified Schwab & England Activities of Daily Living | -0.116(-0.634-0.402) | 0.663 |
|  |  | Modified Semantic Fluency | 0.589(-0.432-1.611) | 0.264 |
|  |  | Montreal Cognitive Assessment | 0.107(-0.07-0.284) | 0.244 |
| INCOME | COHORT | | -0.416(-1.132-0.301) | 0.261 |
|  | CSF | 3-MT(ng/ml) | 0.021(-0.09-0.133) | 0.711 |
|  |  | 4-Hydroxy-3-methoxymandelic acid (VMA)(ng/ml) | -0.038(-0.141-0.066) | 0.485 |
|  |  | α-Synuclein(PG/ML) | 0.289(-0.021-0.599) | 0.084 |
|  |  | Aβ(pg/mL) | -0.085(-0.262-0.092) | 0.391 |
|  |  | Aβ_1-42(pg/mL) | -0.014(-0.093-0.065) | 0.726 |
|  |  | DOPA(ng/ml) | 0.036(-0.036-0.108) | 0.337 |
|  |  | DOPAC(ng/ml) | 0.054(-0.087-0.196) | 0.462 |
|  |  | Dopamine(ng/ml) | -0.016(-0.526-0.495) | 0.953 |
|  |  | GFAP(NG/ML) | -0.008(-0.101-0.084) | 0.863 |
|  |  | Homovanillic acid (HVA)(ng/ml) | 0.053(-0.088-0.194) | 0.471 |
|  |  | IL-1b(ug/ul) | -0.057(-0.113--0.001) | 0.066 |
|  |  | IL-6(PG/ML) | 0.012(-0.110-0.135) | 0.845 |
|  |  | NFL(PG/ML) | 0.004(-0.144-0.151) | 0.963 |
|  |  | pTau (pg/ml) | -0.003(-0.07-0.065) | 0.938 |
|  |  | S100B(NG/ML) | -0.055(-0.120-0.010) | 0.106 |
|  |  | sTREM2(NG/ML) | -0.037(-0.135-0.060) | 0.458 |
|  |  | tTau(pg/ml) | -0.005(-0.073-0.063) | 0.887 |
|  |  | YKL40(NG/ML) | -0.032(-0.151-0.088) | 0.607 |
|  | DAT | Left Caudate | 0.059(-0.103-0.222) | 0.476 |
|  |  | Right Caudate | 0.047(-0.113-0.206) | 0.569 |
|  |  | Left Putamen | 0.012(-0.173-0.197) | 0.896 |
|  |  | Left Anterior of Putamen | 0.049(-0.131-0.228) | 0.598 |
|  |  | Right Putamen | 0.039(-0.126-0.203) | 0.649 |
|  |  | Right Anterior of Putamen | 0.056(-0.110-0.223) | 0.510 |
|  | DTI | Left Rostral | 0.002(-0.064-0.068) | 0.954 |
|  |  | Left Middle | 0.017(-0.039-0.073) | 0.569 |
|  |  | Left Caudal | -0.014(-0.082-0.054) | 0.697 |
|  |  | Right Rostral | 0.022(-0.07-0.113) | 0.649 |
|  |  | Right Middle | -0.001(-0.098-0.096) | 0.983 |
|  |  | Right Caudal | -0.024(-0.113-0.064) | 0.600 |
|  | SCALEs | Benton Judgement of Line Orientation | 0.290(-0.508-1.087) | 0.480 |
|  |  | Letter-Number Sequencing Test | 0.141(-0.391-0.673) | 0.605 |
|  |  | Hopkins Verbal Learning Test | 1.325(0.187-2.463) | 0.027 |
|  |  | Lexical Fluency-A | 0.957(0.035-1.879) | **0.047** |
|  |  | Lexical Fluency-F | 0.641(-0.472-1.754) | 0.265 |
|  |  | Lexical Fluency-S | 1.265(-0.412-2.942) | 0.145 |
|  |  | MDS-UPDRS Part I (Rater Completed) | -0.106(-0.422-0.211) | 0.515 |
|  |  | MDS-UPDRS Part I (Patient Questionnaire) | -0.484(-1.086-0.118) | 0.121 |
|  |  | MDS-UPDRS Part II | 0.118(-0.752-0.988) | 0.791 |
|  |  | MDS-UPDRS Part III | -0.743(-2.692-1.205) | 0.458 |
|  |  | MDS-UPDRS Part IV | 0.394(-0.261-1.049) | 0.246 |
|  |  | Modified Boston Naming Test | 0.881(-1.56-3.323) | 0.483 |
|  |  | Modified Schwab & England Activities of Daily Living | -0.408(-1.564-0.749) | 0.493 |
|  |  | Modified Semantic Fluency | 1.794(-0.323-3.911) | 0.103 |
|  |  | Montreal Cognitive Assessment | 0.296(-0.096-0.688) | 0.145 |
| APOE | COHORT | | -0.022(-0.1-0.056) | 0.583 |
|  | CSF | 3-MT(ng/ml) | -0.010(-0.125-0.104) | 0.860 |
|  |  | 4-Hydroxy-3-methoxymandelic acid (VMA)(ng/ml) | -0.062(-0.153-0.030) | 0.188 |
|  |  | α-Synuclein(PG/ML) | 0.016(-0.133-0.165) | 0.833 |
|  |  | Aβ(pg/mL) | -0.143(-0.337-0.051) | 0.153 |
|  |  | Aβ_1-42(pg/mL) | -0.223(-0.287--0.158) | **＜0.001** |
|  |  | DOPA(ng/ml) | 0.008(-0.085-0.100) | 0.870 |
|  |  | DOPAC(ng/ml) | 0.011(-0.121-0.143) | 0.871 |
|  |  | Dopamine(ng/ml) | -0.223(-0.457-0.011) | 0.064 |
|  |  | GFAP(NG/ML) | -0.007(-0.110-0.096) | 0.897 |
|  |  | Homovanillic acid (HVA)(ng/ml) | -0.006(-0.152-0.139) | 0.933 |
|  |  | IL-1b(ug/ul) | -0.019(-0.149-0.111) | 0.778 |
|  |  | IL-6(PG/ML) | -0.043(-0.152-0.065) | 0.437 |
|  |  | NFL(PG/ML) | 0.000(-0.110-0.109) | 0.996 |
|  |  | pTau (pg/ml) | 0.010(-0.045-0.065) | 0.719 |
|  |  | S100B(NG/ML) | 0.058(-0.001-0.118) | 0.057 |
|  |  | sTREM2(NG/ML) | -0.075(-0.147--0.003) | 0.043 |
|  |  | tTau(pg/ml) | -0.002(-0.055-0.051) | 0.942 |
|  |  | YKL40(NG/ML) | -0.065(-0.197-0.067) | 0.337 |
|  | DAT | Left Caudate | 0.032(-0.081-0.144) | 0.578 |
|  |  | Right Caudate | 0.028(-0.085-0.141) | 0.623 |
|  |  | Left Putamen | 0.026(-0.078-0.131) | 0.621 |
|  |  | Left Anterior of Putamen | 0.029(-0.082-0.140) | 0.606 |
|  |  | Right Putamen | 0.008(-0.098-0.114) | 0.876 |
|  |  | Right Anterior of Putamen | 0.004(-0.109-0.116) | 0.951 |
|  | DTI | Left Rostral | -0.008(-0.025-0.010) | 0.375 |
|  |  | Left Middle | 0.002(-0.015-0.018) | 0.844 |
|  |  | Left Caudal | 0.005(-0.010-0.019) | 0.531 |
|  |  | Right Rostral | -0.016(-0.033-0.000) | 0.056 |
|  |  | Right Middle | -0.002(-0.018-0.015) | 0.854 |
|  |  | Right Caudal | -0.003(-0.017-0.012) | 0.730 |
|  | SCALEs | Benton Judgement of Line Orientation | -0.101(-0.781-0.578) | 0.770 |
|  |  | Letter-Number Sequencing Test | 0.162(-0.254-0.579) | 0.445 |
|  |  | Hopkins Verbal Learning Test | 0.442(-0.485-1.369) | 0.350 |
|  |  | Lexical Fluency-A | 0.101(-0.906-1.109) | 0.844 |
|  |  | Lexical Fluency-F | 0.427(-0.611-1.464) | 0.421 |
|  |  | Lexical Fluency-S | -1.233(-2.799-0.333) | 0.123 |
|  |  | MDS-UPDRS Part I (Rater Completed) | -0.030(-0.293-0.232) | 0.822 |
|  |  | MDS-UPDRS Part I (Patient Questionnaire) | -0.112(-0.62-0.396) | 0.666 |
|  |  | MDS-UPDRS Part II | 0.043(-0.662-0.749) | 0.904 |
|  |  | MDS-UPDRS Part III | 0.081(-1.641-1.803) | 0.926 |
|  |  | MDS-UPDRS Part IV | -0.267(-0.791-0.257) | 0.319 |
|  |  | Modified Boston Naming Test | 0.733(-1.000-2.465) | 0.408 |
|  |  | Modified Schwab & England Activities of Daily Living | -0.372(-1.541-0.796) | 0.533 |
|  |  | Modified Semantic Fluency | 1.053(-0.471-2.577) | 0.176 |
|  |  | Montreal Cognitive Assessment | 0.072(-0.311-0.455) | 0.713 |
| GBA | COHORT | | 0.097(0.053-0.141) | **＜0.001** |
|  | CSF | 3-MT(ng/ml) | 0.086(-0.305-0.478) | 0.665 |
|  |  | 4-Hydroxy-3-methoxymandelic acid (VMA)(ng/ml) | 0.216(-0.133-0.564) | 0.228 |
|  |  | α-Synuclein(PG/ML) | 0.075(-0.491-0.64) | 0.796 |
|  |  | Aβ(pg/mL) | -0.177(-0.355-0.001) | 0.055 |
|  |  | Aβ_1-42(pg/mL) | -0.146(-0.312-0.021) | 0.087 |
|  |  | DOPA(ng/ml) | 0.415(0.070-0.760) | **0.020** |
|  |  | DOPAC(ng/ml) | -0.366(-0.853-0.122) | 0.144 |
|  |  | Dopamine(ng/ml) | 0.037(-0.864-0.938) | 0.935 |
|  |  | GFAP(NG/ML) | 0.110(-0.243-0.462) | 0.542 |
|  |  | Homovanillic acid (HVA)(ng/ml) | -0.198(-0.737-0.341) | 0.473 |
|  |  | IL-1b(ug/ul) | -0.245(-0.746-0.256) | 0.340 |
|  |  | IL-6(PG/ML) | 0.269(-0.101-0.638) | 0.155 |
|  |  | NFL(PG/ML) | -0.043(-0.417-0.33) | 0.821 |
|  |  | pTau (pg/ml) | -0.059(-0.163-0.045) | 0.264 |
|  |  | S100B(NG/ML) | 0.067(-0.137-0.271) | 0.521 |
|  |  | sTREM2(NG/ML) | 0.074(-0.175-0.322) | 0.562 |
|  |  | tTau(pg/ml) | -0.043(-0.143-0.056) | 0.394 |
|  |  | YKL40(NG/ML) | -0.066(-0.517-0.386) | 0.775 |
|  | DAT | Left Caudate | -0.216(-0.423--0.008) | **0.042** |
|  |  | Right Caudate | -0.323(-0.531--0.116) | **0.002** |
|  |  | Left Putamen | -0.284(-0.477--0.092) | **0.004** |
|  |  | Left Anterior of Putamen | -0.302(-0.506--0.097) | **0.004** |
|  |  | Right Putamen | -0.341(-0.536--0.147) | **0.001** |
|  |  | Right Anterior of Putamen | -0.347(-0.553--0.141) | **0.001** |
|  | DTI | Left Rostral | -0.045(-0.102-0.011) | 0.118 |
|  |  | Left Middle | -0.022(-0.077-0.032) | 0.425 |
|  |  | Left Caudal | 0.000(-0.047-0.047) | 0.991 |
|  |  | Right Rostral | -0.011(-0.066-0.044) | 0.691 |
|  |  | Right Middle | -0.025(-0.078-0.027) | 0.351 |
|  |  | Right Caudal | -0.007(-0.054-0.040) | 0.766 |
|  | SCALEs | Benton Judgement of Line Orientation | -0.634(-1.838-0.570) | 0.303 |
|  |  | Letter-Number Sequencing Test | 0.119(-0.619-0.857) | 0.753 |
|  |  | Hopkins Verbal Learning Test | 0.114(-1.487-1.715) | 0.889 |
|  |  | Lexical Fluency-A | 0.515(-1.229-2.258) | 0.563 |
|  |  | Lexical Fluency-F | 0.247(-1.545-2.038) | 0.787 |
|  |  | Lexical Fluency-S | -0.033(-2.818-2.751) | 0.981 |
|  |  | MDS-UPDRS Part I (Rater Completed) | 0.497(0.039-0.956) | **0.034** |
|  |  | MDS-UPDRS Part I (Patient Questionnaire) | 1.975(1.096-2.855) | **＜0.001** |
|  |  | MDS-UPDRS Part II | 2.386(1.152-3.619) | **＜0.001** |
|  |  | MDS-UPDRS Part III | 5.910(2.923-8.898) | **＜0.001** |
|  |  | MDS-UPDRS Part IV | -0.167(-0.991-0.657) | 0.691 |
|  |  | Modified Boston Naming Test | -2.316(-5.265-0.633) | 0.124 |
|  |  | Modified Schwab & England Activities of Daily Living | -1.323(-3.315-0.670) | 0.194 |
|  |  | Modified Semantic Fluency | 1.410(-1.297-4.117) | 0.308 |
|  |  | Montreal Cognitive Assessment | -0.874(-1.545--0.204) | **0.011** |
| LRRK2 | COHORT | | 0.000(-0.057-0.057) | **＜0.001** |
|  | CSF | 3-MT(ng/ml) | 0.120(-0.302-0.541) | 0.578 |
|  |  | 4-Hydroxy-3-methoxymandelic acid (VMA)(ng/ml) | -0.434(-1.031-0.163) | 0.156 |
|  |  | α-Synuclein(PG/ML) | 0.230(-0.739-1.199) | 0.643 |
|  |  | Aβ(pg/mL) | 0.061(-0.115-0.236) | 0.499 |
|  |  | Aβ_1-42(pg/mL) | -0.081(-0.184-0.021) | 0.120 |
|  |  | DOPA(ng/ml) | -0.026(-0.630-0.578) | 0.932 |
|  |  | DOPAC(ng/ml) | 0.432(-0.428-1.292) | 0.326 |
|  |  | Dopamine(ng/ml) | 0.508(-1.036-2.051) | 0.520 |
|  |  | GFAP(NG/ML) | -0.123(-0.503-0.256) | 0.525 |
|  |  | Homovanillic acid (HVA)(ng/ml) | -0.045(-0.995-0.904) | 0.926 |
|  |  | IL-1b(ug/ul) | / | / |
|  |  | IL-6(PG/ML) | -0.133(-0.532-0.266) | 0.514 |
|  |  | NFL(PG/ML) | -0.099(-0.501-0.303) | 0.629 |
|  |  | pTau (pg/ml) | -0.035(-0.109-0.040) | 0.364 |
|  |  | S100B(NG/ML) | 0.091(-0.130-0.311) | 0.421 |
|  |  | sTREM2(NG/ML) | 0.083(-0.184-0.351) | 0.542 |
|  |  | tTau(pg/ml) | -0.031(-0.102-0.041) | 0.398 |
|  |  | YKL40(NG/ML) | 0.087(-0.399-0.574) | 0.725 |
|  | DAT | Left Caudate | -0.371(-0.521--0.221) | **＜0.001** |
|  |  | Right Caudate | -0.372(-0.523--0.222) | **＜0.001** |
|  |  | Left Putamen | -0.383(-0.522--0.244) | **＜0.001** |
|  |  | Left Anterior of Putamen | -0.438(-0.585--0.290) | **＜0.001** |
|  |  | Right Putamen | -0.436(-0.576--0.295) | **＜0.001** |
|  |  | Right Anterior of Putamen | -0.455(-0.604--0.307) | **＜0.001** |
|  | DTI | Left Rostral | 0.063(-0.075-0.201) | 0.374 |
|  |  | Left Middle | 0.061(-0.072-0.194) | 0.368 |
|  |  | Left Caudal | 0.026(-0.088-0.140) | 0.651 |
|  |  | Right Rostral | -0.033(-0.165-0.100) | 0.630 |
|  |  | Right Middle | 0.048(-0.079-0.175) | 0.462 |
|  |  | Right Caudal | 0.078(-0.035-0.191) | 0.179 |
|  | SCALEs | Benton Judgement of Line Orientation | -1.941(-2.835--1.046) | **＜0.001** |
|  |  | Letter-Number Sequencing Test | -0.629(-1.178--0.081) | **0.025** |
|  |  | Hopkins Verbal Learning Test | 1.143(-0.026-2.311) | 0.056 |
|  |  | Lexical Fluency-A | 0.089(-1.189-1.366) | 0.892 |
|  |  | Lexical Fluency-F | -0.301(-1.62-1.018) | 0.655 |
|  |  | Lexical Fluency-S | -0.221(-2.296-1.855) | 0.835 |
|  |  | MDS-UPDRS Part I (Rater Completed) | 0.885(0.545-1.226) | **＜0.001** |
|  |  | MDS-UPDRS Part I (Patient Questionnaire) | 1.849(1.192-2.506) | **＜0.001** |
|  |  | MDS-UPDRS Part II | 2.609(1.688-3.529) | **＜0.001** |
|  |  | MDS-UPDRS Part III | 3.783(1.531-6.035) | **0.001** |
|  |  | MDS-UPDRS Part IV | -0.534(-1.146-0.077) | 0.087 |
|  |  | Modified Boston Naming Test | -2.423(-4.572--0.274) | **0.028** |
|  |  | Modified Schwab & England Activities of Daily Living | -2.149(-3.628--0.669) | **0.005** |
|  |  | Modified Semantic Fluency | -0.889(-2.908-1.129) | 0.388 |
|  |  | Montreal Cognitive Assessment | -1.331(-1.829--0.833) | **＜0.001** |

3-MT, 3-Methoxytyrosine; DOPA, 3,4-Dihydroxymandelic acid; DOPAC, 3,4-Dihydroxyphenylacetic acid; GFAP, glial fibrillary acid protein; IL-1b, Interleukin 1b; IL-6, Interleukin 6; NFL, neurofilament light; S100B, S-100 calcium binding protein B; sTREM2, soluble triggering receptor expressed on myeloid cells 2; VMA, Vanillymandelic Acid; YKL40, chitinase-3-like protein 1.
