# Supplementary material for: Uncovering the Associations of LILRB4 Genotypes With Parkinson's Disease: From Clinical Traits to Potential Pathologies
Source: CNS Neurosci Ther. 2025 Jul 23;31(7):e70522. doi: 10.1111/cns.70522 (PMC12287542; doi:10.1111/cns.70522)
Supplement: Supplementary file 4 — Table S1. [file CNS-31-e70522-s002.zip › cns70522-sup-0005-TableS2-S4@Supplementary Table 2-4 Model 1_LILRB4 genotypes with PD.docx]

**Supplementary Table 2** *LILRB4* genotypes with PD.

| SNPs | | HCs | PDs | P | OR | 95%CI |
| --- | --- | --- | --- | --- | --- | --- |
| rs731170 | **Genotype** |  |  |  |  |  |
|  | GG | 78(9.7%) | 304(37.9%) |  | 1 |  |
|  | GA | 94(11.7%) | 248(30.9%) | **0.026** | 0.677 | 0.480-0.955 |
|  | AA | 25(3.1%) | 54(6.7%) | **0.029** | 0.554 | 0.324-0.947 |
|  | **Allele** |  |  |  |  |  |
|  | G | 250(15.6%) | 856(53.3%) |  | 1 |  |
|  | A | 144(8.9%) | 356(22.2%) | **0.008** | 0.722 | 0.568-0.917 |
| rs1048801 | **Genotype** |  |  |  |  |  |
|  | GG | 83(10.3%) | 242(30.1%) |  | 1 |  |
|  | GA | 82(10.2%) | 273(34.0%) | 0.458 | 1.142 | 0.804-1.622 |
|  | AA | 32(4.0%) | 91(11.3%) | 0.918 | 1.021 | 0.807-1.292 |
|  | **Allele** |  |  |  |  |  |
|  | G | 248(15.4%) | 757(47.1%) |  | 1 |  |
|  | A | 146(9.1%) | 455(28.3%) | 0.863 | 1.217 | 0.723-2.049 |
| rs1749316 | **Genotype** |  |  |  |  |  |
|  | CC | 109(13.6%) | 310(38.6%) |  | 1 |  |
|  | CT | 74(9.2%) | 251(31.3%) | 0.308 | 1.193 | 0.850-1.674 |
|  | TT | 14(1.7%) | 45(5.6%) | 0.707 | 1.130 | 0.597-2.140 |
|  | **Allele** |  |  |  |  |  |
|  | C | 292(18.2%) | 871(54.2%) |  | 1 |  |
|  | T | 102(6.4%) | 341(21.2%) | 0.386 | 1.121 | 0.866-1.450 |
| rs1749317 | **Genotype** |  |  |  |  |  |
|  | AA | 112(13.9%) | 285(35.5%) |  | 1 |  |
|  | AG | 78(16.2%) | 266(33.1%) | **0.004** | 1.659 | 1.168-2.357 |
|  | GG | 22(2.7%) | 55(6.8%) | 0.949 | 0.982 | 0.572-1.687 |
|  | **Allele** |  |  |  |  |  |
|  | A | 287(17.9%) | 836(52.1%) |  | 1 |  |
|  | G | 107(6.7%) | 376(23.4%) | 0.146 | 1.206 | 0.937-1.554 |
| rs1925241 | **Genotype** |  |  |  |  |  |
|  | TT | 66(8.2%) | 208(25.9%) |  | 1 |  |
|  | TC | 104(13.0%) | 269(33.5%) | 0.279 | 0.821 | 0.574-1.174 |
|  | CC | 27(3.4%) | 129(16.1%) | 0.101 | 1.516 | 0.921-2.496 |
|  | **Allele** |  |  |  |  |  |
|  | T | 236(14.7%) | 685(42.7%) |  | 1 |  |
|  | C | 158(9.8%) | 527(32.8%) | 0.239 | 1.149 | 0.912-1.448 |
| rs2569715 | **Genotype** |  |  |  |  |  |
|  | AA | 85(10.6%) | 250(31.1%) |  | 1 |  |
|  | AG | 89(11.1%) | 272(33.9%) | 0.827 | 1.039 | 0.737-1.465 |
|  | GG | 23(2.9%) | 84(10.5%) | 0.416 | 1.242 | 0.736-2.094 |
|  | **Allele** |  |  |  |  |  |
|  | A | 259(16.1%) | 772(48.1%) |  | 1 |  |
|  | G | 135(8.4%) | 440(27.4%) | 0.463 | 1.093 | 0.861-1.388 |
| rs2569716 | **Genotype** |  |  |  |  |  |
|  | AA | 86(10.7%) | 237(29.5%) |  | 1 |  |
|  | AG | 80(10.0%) | 290(36.1%) | 0.124 | 1.315 | 0.927-1.866 |
|  | GG | 31(3.9%) | 79(9.8%) | 0.751 | 0.925 | 0.570-1.499 |
|  | **Allele** |  |  |  |  |  |
|  | A | 252(15.7%) | 764(47.6%) |  | 1 |  |
|  | G | 142(8.8%) | 448(27.9%) | 0.741 | 1.041 | 0.822-1.318 |
| rs3745871 | **Genotype** |  |  |  |  |  |
|  | TT | 82(9.8%) | 228(28.4%) |  |  |  |
|  | TC | 98(12.2%) | 270(33.6%) | 0.958 | 0.991 | 0.704-1.395 |
|  | CC | 17(2.1%) | 108(13.4%) | **0.004** | 2.285 | 1.292-4.041 |
|  | **Allele** |  |  |  |  |  |
|  | T | 262(16.3%) | 726(45.2%) |  | 1 |  |
|  | C | 132(8.2%) | 486(30.3%) | **0.019** | 1.329 | 1.047-1.687 |
| rs11540761 | **Genotype** |  |  |  |  |  |
|  | GG | 136(16.9%) | 385(47.9%) |  | 1 |  |
|  | GT | 56(7.0%) | 185(23.0%) | 0.397 | 1.167 | 0.816-1.668 |
|  | TT | 5(0.6%) | 33(4.1%) | 0.076 | 2.331 | 0.892-6.093 |
|  | **Allele** |  |  |  |  |  |
|  | G | 328(20.4%) | 955(59.5%) |  | 1 |  |
|  | T | 66(4.2%) | 251(15.6%) | 0.079 | 1.306 | 0.969-1.761 |
| rs11574576 | **Genotype** |  |  |  |  |  |
|  | AA | 89(11.1%) | 315(39.2%) |  | 1 |  |
|  | AG | 91(11.3%) | 223(27.8%) | 0.033 | 0.692 | 0.493-0.972 |
|  | GG | 16(2.0%) | 68(8.5%) | 0.545 | 1.201 | 0.663-2.173 |
|  | **Allele** |  |  |  |  |  |
|  | A | 269(16.7%) | 853(53.1%) |  | 1 |  |
|  | G | 123(7.7%) | 359(22.4%) | 0.510 | 0.920 | 0.719-1.178 |
| rs28366008 | **Genotype** |  |  |  |  |  |
|  | TT | 111(13.8%) | 360(44.8%) |  | 1 |  |
|  | TA | 72(9.0%) | 21927.3%) | 0.712 | 0.938 | 0.667-1.319 |
|  | AA | 14(1.7%) | 27(3.4%) | 0.130 | 0.595 | 0.301-1.173 |
|  | **Allele** |  |  |  |  |  |
|  | T | 294(18.3%) | 939(58.5%) |  | 1 |  |
|  | A | 100(6.2%) | 273(17.0%) | 0.244 | 0.855 | 0.656-1.113 |

CI, Confidence internal; HC, healthy controls; PD, Parkinson’s Disease patients; OR, Odds ratio.

**Supplementary Table 3** *LILRB4* genotypes with PD in male.

| SNPs | | HCs | PDs | P | OR | 95%CI |
| --- | --- | --- | --- | --- | --- | --- |
| rs731170 | **Genotype** |  |  |  |  |  |
|  | GG | 52(10.6%) | 180(36.6%) |  | 1 |  |
|  | GA | 59(12.0%) | 151(30.7%) | 0.169 | 0.739 | 0.480-0.138 |
|  | AA | 16(3.3%) | 34(6.9%) | 0.151 | 0.614 | 0.314-1.199 |
|  | **Allele** |  |  |  |  |  |
|  | G | 161(16.4%) | 511(51.9%) |  | 1 |  |
|  | A | 91(9.2%) | 219(22.3%) | 0.072 | 0.758 | 0.561-1.025 |
| rs1048801 | **Genotype** |  |  |  |  |  |
|  | GG | 58(11.8%) | 149(30.3%) |  | 1 |  |
|  | GA | 49(10.0%) | 160(32.5%) | 0.286 | 1.271 | 0.818-1.975 |
|  | AA | 20(4.1%) | 56(11.4%) | 0.776 | 1.090 | 0.602-1.974 |
|  | **Allele** |  |  |  |  |  |
|  | G | 165(16.8%) | 458(46.5%) |  | 1 |  |
|  | A | 89(9.0%) | 272(27.6%) | 0.527 | 1.101 | 0.817-1.484 |
| rs1749316 | **Genotype** |  |  |  |  |  |
|  | CC | 74(15.0%) | 185(37.6%) |  | 1 |  |
|  | CT | 45(9.1%) | 155(31.5%) | 0.141 | 1.378 | 0.898-2.113 |
|  | TT | 8(1.6%) | 25(5.1%) | 0.602 | 1.250 | 0.539-2.897 |
|  | **Allele** |  |  |  |  |  |
|  | C | 193(19.6%) | 525(53.4%) |  | 1 |  |
|  | T | 61(6.2%) | 205(20.8%) | 0.209 | 1.235 | 0.888-1.719 |
| rs1749317 | **Genotype** |  |  |  |  |  |
|  | AA | 70(14.2%) | 179(36.4%) |  | 1 |  |
|  | AG | 47(9.6%) | 152(30.9%) | 0.282 | 1.265 | 0.824-1.941 |
|  | GG | 10(2.0%) | 34(6.9%) | 0.460 | 1.330 | 0.624-2.835 |
|  | **Allele** |  |  |  |  |  |
|  | A | 187(19.0%) | 510(51.8%) |  | 1 |  |
|  | G | 67(6.8%) | 220(22.4%) | 0.256 | 1.204 | 0.874-1.659 |
| rs1925241 | **Genotype** |  |  |  |  |  |
|  | TT | 42(8.5%) | 129(26.2%) |  | 1 |  |
|  | TC | 63(12.8%) | 165(33.5%) | 0.491 | 0.853 | 0.542-1.342 |
|  | CC | 22(4.5%) | 71(14.4%) | 0.870 | 1.051 | 0.582-1.899 |
|  | **Allele** |  |  |  |  |  |
|  | T | 147(14.9%) | 423(43.0%) |  | 1 |  |
|  | C | 107(10.9%) | 307(31.2%) | 0.984 | 0.997 | 0.747-1.331 |
| rs2569715 | **Genotype** |  |  |  |  |  |
|  | AA | 50(10.8%) | 150(30.5%) |  | 1 |  |
|  | AG | 59(12.0%) | 173(35.2%) | 0.918 | 0.977 | 0.632-1.511 |
|  | GG | 18(3.7%) | 42(8.5%) | 0.440 | 0.778 | 0.411-1.472 |
|  | **Allele** |  |  |  |  |  |
|  | A | 159(16.2%) | 473(48.1%) |  | 1 |  |
|  | G | 95(9.7%) | 257(26.1%) | 0.529 | 0.909 | 0.676-1.223 |
| rs2569716 | **Genotype** |  |  |  |  |  |
|  | AA | 53(10.8%) | 147(29.9%) |  | 1 |  |
|  | AG | 50(10.2%) | 176(35.8%) | 0.292 | 1.269 | 0.814-1.979 |
|  | GG | 24(4.9%) | 45(9.1%) | 0.189 | 0.676 | 0.376-1.215 |
|  | **Allele** |  |  |  |  |  |
|  | A | 156(15.8%) | 470(47.8%) |  | 1 |  |
|  | G | 98(10.0%) | 266(27.0%) | 0.973 | 1.004 | 0.778-1.296 |
| rs3745871 | **Genotype** |  |  |  |  |  |
|  | TT | 50(10.2%) | 141(28.6%) |  |  |  |
|  | TC | 63(12.8%) | 161(32.7%) | 0.657 | 0.906 | 0.587-1.400 |
|  | CC | 14(2.8%) | 63(12.8%) | 0.165 | 1.596 | 0.822-3.096 |
|  | **Allele** |  |  |  |  |  |
|  | T | 163(16.6%) | 443(45.0%) |  | 1 |  |
|  | C | 91(9.2%) | 287(29.2%) | 0.325 | 1.160 | 0.863-1.561 |
| rs11540761 | **Genotype** |  |  |  |  |  |
|  | GG | 84(17.1%) | 239(48.6%) |  | 1 |  |
|  | GT | 38(7.7%) | 105(21.3%) | 0.898 | 0.971 | 0.621-1.518 |
|  | TT | 5(0.1%) | 21(4.3%) | 0.446 | 1.476 | 0.540-4.039 |
|  | **Allele** |  |  |  |  |  |
|  | G | 206(20.9%) | 583(59.2%) |  | 1 |  |
|  | T | 48(4.9%) | 147(14.9%) | 0.670 | 1.082 | 0.753-1.555 |
| rs11574576 | **Genotype** |  |  |  |  |  |
|  | AA | 64(13.0%) | 194(39.4%) |  | 1 |  |
|  | AG | 52(10.6%) | 132(26.8%) | 0.416 | 0.837 | 0.546-1.284 |
|  | GG | 10(2.0%) | 39(7.9%) | 0.509 | 1.287 | 0.608-2.724 |
|  | **Allele** |  |  |  |  |  |
|  | A | 180(18.3%) | 520(52.3%) |  | 1 |  |
|  | G | 72(7.3%) | 210(21.3%) | 0.953 | 1.010 | 0.735-1.386 |
| rs28366008 | **Genotype** |  |  |  |  |  |
|  | TT | 73(14.8%) | 227(46.1%) |  | 1 |  |
|  | TA | 46(9.3%) | 121(24.6%) | 0.445 | 0.846 | 0.550-1.300 |
|  | AA | 8(1.6%) | 17(3.5%) | 0.395 | 0.683 | 0.283-1.649 |
|  | **Allele** |  |  |  |  |  |
|  | T | 192(19.5%) | 575(58.4%) |  | 1 |  |
|  | A | 62(6.3%) | 155(15.8%) | 0.293 | 0.835 | 0.596-1.169 |

HC, healthy controls; PD, Parkinson’s Disease patients; OR, Odds ratio; CI, Confidence internal.

**Supplementary Table 4** *LILRB4* genotypes with PD in female.

| SNPs | | HCs | PDs | P | OR | 95%CI |
| --- | --- | --- | --- | --- | --- | --- |
| rs731170 | **Genotype** |  |  |  |  |  |
|  | GG | 26(8.4%) | 124(39.9%) |  | 1 |  |
|  | GA | 35(11.3%) | 97(31.2%) | 0.062 | 0.581 | 0.328-1.030 |
|  | AA | 9(2.9%) | 20(6.4%) | 0.089 | 0.466 | 0.191-1.138 |
|  | **Allele** |  |  |  |  |  |
|  | G | 87(14.0%) | 345(55.5%) |  | 1 |  |
|  | A | 53(8.5%) | 137(22.0%) | **0.033** | 0.652 | 0.439-0.967 |
| rs1048801 | **Genotype** |  |  |  |  |  |
|  | GG | 25(8.0%) | 93(30.0%) |  | 1 |  |
|  | GA | 33(10.6%) | 113(36.3%) | 0.782 | 0.920 | 0.511-1.657 |
|  | AA | 12(3.9%) | 35(11.3%) | 0.546 | 0.784 | 0.356-1.728 |
|  | **Allele** |  |  |  |  |  |
|  | G | 83(13.3%) | 299(48.1%) |  | 1 |  |
|  | A | 57(9.2%) | 183(29.4%) | 0.557 | 0.891 | 0.607-1.309 |
| rs1749316 | **Genotype** |  |  |  |  |  |
|  | CC | 35(11.3%) | 125(40.2%) |  | 1 |  |
|  | CT | 29(9.3%) | 96(30.9%) | 0.790 | 0.927 | 0.530-1.622 |
|  | TT | 6(1.9%) | 20(6.4%) | 0.891 | 0.933 | 0.348-2.503 |
|  | **Allele** |  |  |  |  |  |
|  | C | 99(15.9%) | 346(55.6%) |  | 1 |  |
|  | T | 41(6.6%) | 136(21.9%) | 0.805 | 0.949 | 0.627-1.437 |
| rs1749317 | **Genotype** |  |  |  |  |  |
|  | AA | 42(13.5%) | 106(34.1%) |  | 1 |  |
|  | AG | 16(5.1%) | 114(36.7%) | **0.001** | 2.823 | 1.498-5.320 |
|  | GG | 12(3.9%) | 21(6.8%) | 0.365 | 0.693 | 0.313-1.534 |
|  | **Allele** |  |  |  |  |  |
|  | A | 100(16.1%) | 326(52.4%) |  | 1 |  |
|  | G | 40(6.4%) | 156(25.1%) | 0.094 | 1.284 | 0.958-1.720 |
| rs1925241 | **Genotype** |  |  |  |  |  |
|  | TT | 24(7.7%) | 79(25.4%) |  | 1 |  |
|  | TC | 41(13.2%) | 104(33.4%) | 0.279 | 0.821 | 0.574-1.174 |
|  | CC | 5(1.6%) | 58(18.6%) | **0.029** | 2.940 | 1.077-8.023 |
|  | **Allele** |  |  |  |  |  |
|  | T | 89(14.3%) | 262(42.1%) |  | 1 |  |
|  | C | 51(8.2%) | 220(35.4%) | 0.053 | 1.465 | 0.994-2.160 |
| rs2569715 | **Genotype** |  |  |  |  |  |
|  | AA | 35(11.3%) | 100(32.2%) |  | 1 |  |
|  | AG | 30(9.6%) | 99(31.8%) | 0.615 | 1.155 | 0.659-2.025 |
|  | GG | 5(1.6%) | 42(13.5%) | 0.416 | 1.242 | 0.736-2.094 |
|  | **Allele** |  |  |  |  |  |
|  | A | 100(16.1%) | 299(48.1%) |  | 1 |  |
|  | G | 40(6.4%) | 181(29.1%) | 0.463 | 1.093 | 0.861-1.388 |
| rs2569716 | **Genotype** |  |  |  |  |  |
|  | AA | 33(10.6%) | 91(29.3%) |  | 1 |  |
|  | AG | 30(9.6%) | 115(37.0%) | 0.300 | 1.342 | 0.769-2.340 |
|  | GG | 7(2.3%) | 35(11.3%) | 0.218 | 1.750 | 0.713-4.296 |
|  | **Allele** |  |  |  |  |  |
|  | A | 96(15.7%) | 297(47.7%) |  | 1 |  |
|  | G | 42(6.8%) | 185(29.7%) | 0.088 | 1.424 | 0.948-2.138 |
| rs3745871 | **Genotype** |  |  |  |  |  |
|  | TT | 32(10.3%) | 87(28.0%) |  |  |  |
|  | TC | 35(11.3%) | 109(35.0%) | 0.632 | 1.145 | 0.657-1.997 |
|  | CC | 3(0.1%) | 45(14.5%) | **0.003** | 5.517 | 1.601-19.007 |
|  | **Allele** |  |  |  |  |  |
|  | T | 99(15.9%) | 283(45.5%) |  | 1 |  |
|  | C | 41(6.6%) | 199(32.0%) | **0.010** | 1.698 | 1.131-2.550 |
| rs11540761 | **Genotype** |  |  |  |  |  |
|  | GG | 52(16.7%) | 146(46.9%) |  | 1 |  |
|  | GT | 18(5.8%) | 83(26.7%) | 0.103 | 1.642 | 0.901-2.992 |
|  | TT | 0(0.0%) | 12(3.9%) | **0.041** | 0.737 | 0.679-0.801 |
|  | **Allele** |  |  |  |  |  |
|  | G | 122(19.6%) | 375(50.3%) |  | 1 |  |
|  | T | 18(2.9%) | 107(17.2%) | **0.015** | 1.934 | 1.128-3.317 |
| rs11574576 | **Genotype** |  |  |  |  |  |
|  | AA | 25(8.0%) | 121(38.9%) |  | 1 |  |
|  | AG | 39(12.5%) | 91(29.3%) | **0.011** | 0.482 | 0.272-0.853 |
|  | GG | 6(1.9%) | 29(9.3%) | 0.998 | 0.999 | 0.375-2.658 |
|  | **Allele** |  |  |  |  |  |
|  | A | 89(14.3%) | 333(53.5%) |  | 1 |  |
|  | G | 51(8.2%) | 149(24.0%) | 0.219 | 0.781 | 0.526-1.159 |
| rs28366008 | **Genotype** |  |  |  |  |  |
|  | TT | 38(12.2%) | 133(428%) |  | 1 |  |
|  | TA | 26(8.4%) | 98(31.5%) | 0.796 | 1.077 | 0.613-1.891 |
|  | AA | 6(1.9%) | 10(3.2%) | 0.168 | 0.476 | 0.163-1.394 |
|  | **Allele** |  |  |  |  |  |
|  | T | 102(16.4%) | 364(58.5%) |  | 1 |  |
|  | A | 38(6.1%) | 118(19.0%) | 0.522 | 0.870 | 0.568-1.333 |

HC, healthy controls; PD, Parkinson’s Disease patients; OR, Odds ratio; CI, Confidence internal.
