# Supplementary material for: Uncovering the Associations of LILRB4 Genotypes With Parkinson's Disease: From Clinical Traits to Potential Pathologies
Source: CNS Neurosci Ther. 2025 Jul 23;31(7):e70522. doi: 10.1111/cns.70522 (PMC12287542; doi:10.1111/cns.70522)
Supplement: Supplementary file 4 — Table S1. [file CNS-31-e70522-s002.zip › cns70522-sup-0006-TableS5-S7@Supplementary Table 5-7 Model 1_The correlation between LILRB4 loci and PD.docx]

**Supplementary Table 5.** Model 1: The correlation between *LILRB4* loci and PD.

| SNP | OR (95%CI) | P | FDR-corrected. P |
| --- | --- | --- | --- |
| rs731170 | 0.941(0.899-0.985) | **0.010** | 0.105 |
| rs1048801 | 1.003(0.961-1.047) | 0.883 | 0.883 |
| rs1749316 | 1.022(0.974-1.072) | 0.372 | 0.585 |
| rs1749317 | 1.03(0.984-1.078) | 0.202 | 0.556 |
| rs1925241 | 1.022(0.98-1.066) | 0.315 | 0.578 |
| rs2569715 | 1.018(0.974-1.064) | 0.427 | 0.587 |
| rs2569716 | 1.013(0.97-1.058) | 0.563 | 0.662 |
| rs3745871 | 1.072(1.027-1.119) | **0.025** | 0.137 |
| rs11540761 | 1.049(0.996-1.105) | 0.069 | 0.254 |
| rs11574576 | 0.988(0.945-1.033) | 0.602 | 0.662 |
| rs28366008 | 0.972(0.924-1.023) | 0.272 | 0.578 |

CI, Confidence internal; FDR, false discovery rate; OR, odds ratio.

**Supplementary Table 6.** Model 1: The correlation between *LILRB4* loci and PD in male.

| SNP | OR (95%CI) | P | FDR-corrected. P |
| --- | --- | --- | --- |
| rs731170 | 0.947(0.893-1.005) | 0.075 | 0.724 |
| rs1048801 | 1.018(0.964-1.076) | 0.522 | 0.732 |
| rs1749316 | 1.045(0.981-1.113) | 0.170 | 0.724 |
| rs1749317 | 1.034(0.974-1.098) | 0.271 | 0.724 |
| rs1925241 | 0.998(0.944-1.054) | 0.938 | 0.938 |
| rs2569715 | 0.982(0.926-1.041) | 0.543 | 0.732 |
| rs2569716 | 0.985(0.93-1.043) | 0.599 | 0.732 |
| rs3745871 | 1.028(0.972-1.088) | 0.329 | 0.724 |
| rs11540761 | 1.019(0.954-1.09) | 0.571 | 0.732 |
| rs11574576 | 1.006(0.948-1.067) | 0.845 | 0.930 |
| rs28366008 | 0.967(0.905-1.033) | 0.318 | 0.724 |

CI, Confidence internal; FDR, false discovery rate; OR, odds ratio.

**Supplementary Table 7.** Model 1: The correlation between *LILRB4* loci and PD in female.

| SNP | OR (95%CI) | P | FDR-corrected. P |
| --- | --- | --- | --- |
| rs731170 | 0.932(0.868-1.001) | 0.055 | 0.152 |
| rs1048801 | 0.977(0.913-1.045) | 0.503 | 0.691 |
| rs1749316 | 0.989(0.92-1.064) | 0.768 | 0.768 |
| rs1749317 | 1.02(0.951-1.094) | 0.584 | 0.691 |
| rs1925241 | 1.059(0.993-1.13) | 0.084 | 0.170 |
| rs2569715 | 1.073(1.005-1.146) | **0.036** | 0.130 |
| rs2569716 | 1.06(0.991-1.135) | 0.093 | 0.170 |
| rs3745871 | 1.086(1.016-1.161) | **0.016** | 0.110 |
| rs11540761 | 1.104(1.016-1.199) | **0.020** | 0.110 |
| rs11574576 | 0.957(0.894-1.026) | 0.218 | 0.343 |
| rs28366008 | 0.981(0.907-1.061) | 0.628 | 0.691 |

CI, Confidence internal; FDR, false discovery rate; OR, odds ratio.
