# Supplementary material for: Uncovering the Associations of LILRB4 Genotypes With Parkinson's Disease: From Clinical Traits to Potential Pathologies
Source: CNS Neurosci Ther. 2025 Jul 23;31(7):e70522. doi: 10.1111/cns.70522 (PMC12287542; doi:10.1111/cns.70522)
Supplement: Supplementary file 4 — Table S1. [file CNS-31-e70522-s002.zip › cns70522-sup-0007-TableS8-S10@Supplementary Table 8-10 Model 2_The correlation between LILRB4 loci and PD.docx]

**Supplementary Table 8.** Model 2: The correlation between *LILRB4* loci and PD.

| SNP | OR (95%CI) | P value | FDR-corrected. P |
| --- | --- | --- | --- |
| rs731170 | 0.954(0.913-0.997) | **0.037** | 0.176 |
| rs1048801 | 0.996(0.957-1.038) | 0.865 | 0.865 |
| rs1749316 | 1.012(0.967-1.06) | 0.6 | 0.733 |
| rs1749317 | 1.019(0.975-1.065) | 0.407 | 0.687 |
| rs1925241 | 1.012(0.972-1.054) | 0.555 | 0.733 |
| rs2569715 | 1.017(0.975-1.061) | 0.431 | 0.687 |
| rs2569716 | 1.022(0.98-1.066) | 0.304 | 0.687 |
| rs3745871 | 1.042(1-1.086) | **0.048** | 0.176 |
| rs11540761 | 1.053(1.002-1.107) | **0.041** | 0.176 |
| rs11574576 | 0.994(0.952-1.038) | 0.784 | 0.862 |
| rs28366008 | 0.981(0.934-1.03) | 0.437 | 0.687 |

CI, Confidence internal; FDR, false discovery rate; OR, odds ratio.

**Supplementary Table 9.** Model 2: The correlation between *LILRB4* loci and PD in male.

| SNP | OR (95%CI) | P value | FDR-corrected. P |
| --- | --- | --- | --- |
| rs731170 | 0.961(0.907-1.018) | 0.174 | 0.949 |
| rs1048801 | 1.01(0.957-1.065) | 0.726 | 0.949 |
| rs1749316 | 1.029(0.968-1.094) | 0.365 | 0.949 |
| rs1749317 | 1.022(0.964-1.083) | 0.472 | 0.949 |
| rs1925241 | 0.993(0.941-1.047) | 0.791 | 0.949 |
| rs2569715 | 0.981(0.926-1.038) | 0.501 | 0.949 |
| rs2569716 | 0.99(0.937-1.046) | 0.713 | 0.871 |
| rs3745871 | 1.026(0.972-1.083) | 0.353 | 0.949 |
| rs11540761 | 1.026(0.962-1.094) | 0.432 | 0.871 |
| rs11574576 | 1.008(0.952-1.066) | 0.796 | 0.949 |
| rs28366008 | 0.981(0.919-1.046) | 0.557 | 0.949 |

CI, Confidence internal; FDR, false discovery rate; OR, odds ratio.

**Supplementary Table 10.** Model 2: The correlation between *LILRB4* loci and PD in female.

| SNP | OR (95%CI) | P value | FDR-corrected. P |
| --- | --- | --- | --- |
| rs731170 | 0.945(0.883-1.011) | 0.103 | 0.228 |
| rs1048801 | 0.973(0.913-1.037) | 0.403 | 0.555 |
| rs1749316 | 0.988(0.922-1.058) | 0.721 | 0.732 |
| rs1749317 | 1.012(0.946-1.082) | 0.732 | 0.732 |
| rs1925241 | 1.043(0.981-1.11) | 0.179 | 0.329 |
| rs2569715 | 1.071(1.007-1.14) | **0.031** | 0.101 |
| rs2569716 | 1.076(1.009-1.148) | **0.027** | 0.101 |
| rs3745871 | 1.07(1.004-1.141) | **0.037** | 0.101 |
| rs11540761 | 1.103(1.02-1.193) | **0.015** | 0.101 |
| rs11574576 | 0.97(0.908-1.036) | 0.361 | 0.555 |
| rs28366008 | 0.983(0.913-1.059) | 0.658 | 0.732 |

CI, Confidence internal; FDR, false discovery rate; OR, odds ratio.
