# Supplementary material for: Uncovering the Associations of LILRB4 Genotypes With Parkinson's Disease: From Clinical Traits to Potential Pathologies
Source: CNS Neurosci Ther. 2025 Jul 23;31(7):e70522. doi: 10.1111/cns.70522 (PMC12287542; doi:10.1111/cns.70522)
Supplement: Supplementary file 4 — Table S1. [file CNS-31-e70522-s002.zip › cns70522-sup-0008-TableS9-S11@Supplementary Table 11-13 Model 1_The correlation between LILRB4 loci and DAT-SPECT striatal binding ratios.docx]

**Supplementary Table 11**. Model 1: The correlation between *LILRB4* loci and DAT-SPECT striatal binding ratios.

| Items | SNP | β(95%CI) | P | FDR-corrected. P |
| --- | --- | --- | --- | --- |
| Left Caudate | rs731170 | 0.121(0.038-0.205) | **0.004** | **0.048** |
|  | rs1048801 | 0.003(-0.076-0.082) | 0.934 | 0.934 |
|  | rs1749316 | -0.065(-0.154-0.024) | 0.153 | 0.288 |
|  | rs1749317 | -0.071(-0.156-0.013) | 0.097 | 0.288 |
|  | rs1925241 | -0.037(-0.113-0.040) | 0.350 | 0.550 |
|  | rs2569715 | -0.058(-0.139-0.022) | 0.157 | 0.288 |
|  | rs2569716 | 0.008(-0.072-0.089) | 0.839 | 0.923 |
|  | rs3745871 | -0.063(-0.142-0.015) | 0.112 | 0.288 |
|  | rs11540761 | -0.088(-0.183-0.006) | 0.067 | 0.288 |
|  | rs11574576 | -0.014(-0.096-0.068) | 0.743 | 0.908 |
|  | rs28366008 | 0.032(-0.062-0.125) | 0.505 | 0.694 |
| Right Caudate | rs731170 | 0.160(0.076-0.244) | **<0.001** | **0.002** |
|  | rs1048801 | 0.013(-0.066-0.093) | 0.744 | 0.779 |
|  | rs1749316 | -0.084(-0.173-0.005) | 0.066 | 0.146 |
|  | rs1749317 | -0.099(-0.184--0.014) | **0.023** | 0.124 |
|  | rs1925241 | -0.062(-0.139-0.015) | 0.116 | 0.213 |
|  | rs2569715 | -0.043(-0.123-0.038) | 0.308 | 0.423 |
|  | rs2569716 | -0.012(-0.093-0.070) | 0.779 | 0.779 |
|  | rs3745871 | -0.080(-0.159--0.001) | **0.047** | 0.128 |
|  | rs11540761 | -1.020(-1.116--0.925) | **0.036** | 0.128 |
|  | rs11574576 | -0.018(-0.101-0.065) | 0.671 | 0.779 |
|  | rs28366008 | 0.066(-0.028-0.160) | 0.170 | 0.267 |
| Left Putamen | rs731170 | 0.101(0.023-0.179) | **0.012** | 0.094 |
|  | rs1048801 | 0.003(-0.071-0.078) | 0.927 | 0.927 |
|  | rs1749316 | -0.061(-0.144-0.022) | 0.151 | 0.243 |
|  | rs1749317 | -0.081(-0.160--0.002) | **0.045** | 0.165 |
|  | rs1925241 | -0.032(-0.103-0.040) | 0.387 | 0.532 |
|  | rs2569715 | -0.055(-0.131-0.021) | 0.155 | 0.243 |
|  | rs2569716 | -0.017(-0.092-0.059) | 0.665 | 0.813 |
|  | rs3745871 | -0.056(-0.129-0.018) | 0.137 | 0.243 |
|  | rs11540761 | -0.108(-0.196--0.019) | **0.017** | 0.094 |
|  | rs11574576 | -0.008(-0.085-0.069) | 0.839 | 0.923 |
|  | rs28366008 | 0.081(-0.007-0.168) | 0.070 | 0.193 |
| Left Anterior of Putamen | rs731170 | 0.113(0.031-0.196) | **0.007** | 0.082 |
|  | rs1048801 | 0.004(-0.075-0.083) | 0.923 | 0.938 |
|  | rs1749316 | -0.057(-0.145-0.031) | 0.207 | 0.325 |
|  | rs1749317 | -0.072(-0.156-0.012) | 0.092 | 0.253 |
|  | rs1925241 | -0.043(-0.119-0.033) | 0.266 | 0.366 |
|  | rs2569715 | -0.063(-0.144-0.018) | 0.126 | 0.277 |
|  | rs2569716 | -0.011(-0.072-0.050) | 0.788 | 0.938 |
|  | rs3745871 | -0.069(-0.147-0.009) | 0.084 | 0.253 |
|  | rs11540761 | -0.103(-0.197--0.009) | **0.032** | 0.175 |
|  | rs11574576 | -0.003(-0.085-0.079) | 0.938 | 0.938 |
|  | rs28366008 | 0.065(-0.028-0.158) | 0.170 | 0.312 |
| Right Putamen | rs731170 | 0.135(0.057-0.214) | **0.001** | **0.009** |
|  | rs1048801 | 0.037(-0.038-0.112) | 0.333 | 0.458 |
|  | rs1749316 | -0.093(-0.177--0.009) | **0.030** | 0.088 |
|  | rs1749317 | -0.057(-0.137-0.024) | 0.167 | 0.262 |
|  | rs1925241 | -0.030(-0.102-0.043) | 0.423 | 0.518 |
|  | rs2569715 | -0.055(-0.131-0.022) | 0.164 | 0.262 |
|  | rs2569716 | -0.012(-0.089-0.065) | 0.757 | 0.757 |
|  | rs3745871 | -0.055(-0.129-0.020) | 0.150 | 0.262 |
|  | rs11540761 | -0.111(-0.201--0.022) | **0.015** | 0.083 |
|  | rs11574576 | -0.023(-0.101-0.055) | 0.565 | 0.622 |
|  | rs28366008 | 0.097(0.008-0.185) | **0.032** | 0.088 |
| Right Anterior of Putamen | rs731170 | 0.156(0.073-0.240) | **<0.001** | **0.003** |
|  | rs1048801 | 0.024(-0.056-0.104) | 0.557 | 0.613 |
|  | rs1749316 | -0.099(-0.188--0.009) | **0.031** | 0.112 |
|  | rs1749317 | -0.080(-0.165-0.005) | 0.065 | 0.143 |
|  | rs1925241 | -0.046(-0.123-0.031) | 0.245 | 0.385 |
|  | rs2569715 | -0.042(-0.123-0.040) | 0.317 | 0.436 |
|  | rs2569716 | -0.007(-0.089-0.074) | 0.858 | 0.858 |
|  | rs3745871 | -0.065(-0.144-0.014) | 0.106 | 0.194 |
|  | rs11540761 | -0.117(-0.212--0.022) | **0.016** | 0.088 |
|  | rs11574576 | -0.035(-0.118-0.048) | 0.404 | 0.493 |
|  | rs28366008 | 0.089(-0.005-0.183) | 0.064 | 0.143 |

CI, Confidence internal; DAT, dopamine transporter; FDR, false discovery rate

**Supplementary Table 9**. Model 1: The correlation between *LILRB4* loci and DAT-SPECT striatal binding ratios in male.

| Items | SNP | β(95%CI) | P | FDR-corrected. P |
| --- | --- | --- | --- | --- |
| Left Caudate | rs731170 | 0.118(0.019-0.216) | **0.020** | 0.221 |
|  | rs1048801 | -0.001(-0.095-0.093) | 0.984 | 0.984 |
|  | rs1749316 | -0.061(-0.168-0.046) | 0.267 | 0.545 |
|  | rs1749317 | -0.062(-0.162-0.039) | 0.230 | 0.545 |
|  | rs1925241 | -0.042(-0.133-0.049) | 0.367 | 0.545 |
|  | rs2569715 | -0.043(-0.142-0.056) | 0.396 | 0.545 |
|  | rs2569716 | 0.031(-0.066-0.128) | 0.533 | 0.586 |
|  | rs3745871 | -0.060(-0.153-0.033) | 0.206 | 0.545 |
|  | rs11540761 | -0.057(-0.167-0.054) | 0.317 | 0.545 |
|  | rs11574576 | -0.050(-0.149-0.049) | 0.324 | 0.545 |
|  | rs28366008 | 0.042(-0.071-0.154) | 0.467 | 0.571 |
| Right Caudate | rs731170 | 0.108(0.007-0.209) | **0.036** | 0.396 |
|  | rs1048801 | 0.024(-0.071-0.120) | 0.618 | 0.755 |
|  | rs1749316 | -0.086(-0.195-0.023) | 0.123 | 0.594 |
|  | rs1749317 | -0.063(-0.165-0.040) | 0.232 | 0.594 |
|  | rs1925241 | -0.019(-0.112-0.074) | 0.692 | 0.761 |
|  | rs2569715 | -0.044(-0.145-0.057) | 0.392 | 0.682 |
|  | rs2569716 | -0.003(-0.102-0.095) | 0.947 | 0.947 |
|  | rs3745871 | -0.027(-0.122-0.068) | 0.578 | 0.755 |
|  | rs11540761 | -0.045(-0.158-0.068) | 0.434 | 0.682 |
|  | rs11574576 | -0.063(-0.164-0.038) | 0.224 | 0.594 |
|  | rs28366008 | 0.064(-0.050-0.179) | 0.270 | 0.594 |
| Left Putamen | rs731170 | 0.090(-0.005-0.184) | 0.063 | 0.334 |
|  | rs1048801 | -0.033(-0.122-0.057) | 0.471 | 0.715 |
|  | rs1749316 | -0.079(-0.181-0.022) | 0.128 | 0.334 |
|  | rs1749317 | -0.089(-0.184-0.007) | 0.070 | 0.334 |
|  | rs1925241 | -0.014(-0.100-0.073) | 0.755 | 0.755 |
|  | rs2569715 | -0.017(-0.111-0.077) | 0.721 | 0.755 |
|  | rs2569716 | 0.022(-0.070-0.114) | 0.636 | 0.755 |
|  | rs3745871 | -0.029(-0.118-0.060) | 0.520 | 0.715 |
|  | rs11540761 | -0.083(-0.188-0.022) | 0.122 | 0.334 |
|  | rs11574576 | -0.062(-0.157-0.032) | 0.196 | 0.359 |
|  | rs28366008 | 0.008(-0.099-0.114) | 0.152 | 0.334 |
| Left Anterior of Putamen | rs731170 | 0.100(0.001-0.200) | **0.049** | 0.475 |
|  | rs1048801 | -0.019(-0.113-0.075) | 0.694 | 0.763 |
|  | rs1749316 | -0.066(-0.173-0.042) | 0.233 | 0.475 |
|  | rs1749317 | -0.070(-0.171-0.031) | 0.177 | 0.475 |
|  | rs1925241 | -0.026(-0.117-0.066) | 0.580 | 0.709 |
|  | rs2569715 | -0.045(-0.144-0.054) | 0.376 | 0.517 |
|  | rs2569716 | 0.015(-0.082-0.112) | 0.765 | 0.765 |
|  | rs3745871 | -0.046(-0.140-0.047) | 0.333 | 0.517 |
|  | rs11540761 | -0.073(-0.184-0.038) | 0.199 | 0.475 |
|  | rs11574576 | -0.058(-0.157-0.042) | 0.259 | 0.475 |
|  | rs28366008 | 0.071(-0.042-0.184) | 0.217 | 0.475 |
| Right Putamen | rs731170 | 0.120(0.024-0.216) | **0.015** | 0.161 |
|  | rs1048801 | 0.016(-0.076-0.107) | 0.737 | 0.811 |
|  | rs1749316 | -0.114(-0.218--0.010) | **0.031** | 0.173 |
|  | rs1749317 | -0.058(-0.156-0.040) | 0.243 | 0.446 |
|  | rs1925241 | -0.005(-0.093-0.084) | 0.913 | 0.913 |
|  | rs2569715 | -0.022(-0.118-0.074) | 0.656 | 0.811 |
|  | rs2569716 | 0.021(-0.073-0.115) | 0.664 | 0.811 |
|  | rs3745871 | -0.021(-0.112-0.069) | 0.644 | 0.811 |
|  | rs11540761 | -0.069(-0.176-0.038) | 0.221 | 0.446 |
|  | rs11574576 | -0.072(-0.168-0.024) | 0.143 | 0.393 |
|  | rs28366008 | 0.086(-0.023-0.195) | 0.122 | 0.393 |
| Right Anterior of Putamen | rs731170 | 0.124(0.022-0.226) | **0.017** | 0.190 |
|  | rs1048801 | 0.017(-0.080-0.114) | 0.727 | 0.889 |
|  | rs1749316 | -0.110(-0.220-0.000) | **0.050** | 0.274 |
|  | rs1749317 | -0.065(-0.168-0.039) | 0.222 | 0.513 |
|  | rs1925241 | -0.010(-0.104-0.084) | 0.836 | 0.916 |
|  | rs2569715 | -0.005(-0.107-0.096) | 0.916 | 0.916 |
|  | rs2569716 | 0.025(-0.075-0.124) | 0.628 | 0.884 |
|  | rs3745871 | -0.023(-0.119-0.073) | 0.643 | 0.884 |
|  | rs11540761 | -0.061(-0.175-0.053) | 0.295 | 0.541 |
|  | rs11574576 | -0.093(-0.195-0.010) | 0.077 | 0.282 |
|  | rs28366008 | 0.070(-0.045-0.186) | 0.233 | 0.513 |

CI, Confidence internal; DAT, dopamine transporter; FDR, false discovery rate

**Supplementary Table 10**. Model 1: The correlation between *LILRB4* loci and DAT-SPECT striatal binding ratios in female.

| Items | SNP | β(95%CI) | P | FDR-corrected. P |
| --- | --- | --- | --- | --- |
| Left Caudate | rs731170 | 0.123(-0.026-0.272) | 0.108 | 0.592 |
|  | rs1048801 | 0.017(-0.125-0.158) | 0.820 | 0.889 |
|  | rs1749316 | -0.068(-0.223-0.087) | 0.394 | 0.722 |
|  | rs1749317 | -0.074(-0.225-0.077) | 0.338 | 0.722 |
|  | rs1925241 | -0.026(-0.162-0.111) | 0.713 | 0.889 |
|  | rs2569715 | -0.087(-0.226-0.052) | 0.222 | 0.722 |
|  | rs2569716 | -0.024(-0.166-0.119) | 0.745 | 0.889 |
|  | rs3745871 | -0.067(-0.207-0.073) | 0.352 | 0.722 |
|  | rs11540761 | -0.147(-0.321-0.027) | 0.098 | 0.592 |
|  | rs11574576 | 0.051(-0.092-0.195) | 0.483 | 0.759 |
|  | rs28366008 | 0.012(-0.153-0.177) | 0.889 | 0.889 |
| Right Caudate | rs731170 | 0.246(0.099-0.392) | **0.001** | **0.013** |
|  | rs1048801 | -0.001(-0.143-0.140) | 0.984 | 0.984 |
|  | rs1749316 | -0.078(-0.233-0.076) | 0.321 | 0.589 |
|  | rs1749317 | -0.151(-0.301--0.001) | **0.049** | 0.117 |
|  | rs1925241 | -0.134(-0.269-0.001) | 0.053 | 0.117 |
|  | rs2569715 | -0.044(-0.183-0.095) | 0.535 | 0.654 |
|  | rs2569716 | -0.022(-0.164-0.120) | 0.764 | 0.840 |
|  | rs3745871 | -0.169(-0.308--0.031) | **0.017** | 0.067 |
|  | rs11540761 | -0.209(-0.381--0.037) | **0.018** | 0.067 |
|  | rs11574576 | 0.059(-0.084-0.202) | 0.422 | 0.592 |
|  | rs28366008 | 0.066(-0.098-0.230) | 0.431 | 0.592 |
| Left Putamen | rs731170 | 0.116(-0.021-0.253) | 0.099 | 0.359 |
|  | rs1048801 | 0.070(-0.060-0.200) | 0.290 | 0.399 |
|  | rs1749316 | -0.029(-0.172-0.113) | 0.687 | 0.687 |
|  | rs1749317 | -0.057(-0.196-0.081) | 0.418 | 0.460 |
|  | rs1925241 | -0.061(-0.186-0.065) | 0.343 | 0.419 |
|  | rs2569715 | -0.116(-0.243-0.011) | 0.075 | 0.359 |
|  | rs2569716 | -0.077(-0.208-0.054) | 0.250 | 0.399 |
|  | rs3745871 | -0.099(-0.228-0.029) | 0.131 | 0.359 |
|  | rs11540761 | -0.154(-0.313-0.006) | 0.060 | 0.359 |
|  | rs11574576 | 0.084(-0.048-0.216) | 0.214 | 0.399 |
|  | rs28366008 | 0.083(-0.069-0.234) | 0.285 | 0.399 |
| Left Anterior of Putamen | rs731170 | 0.131(-0.016-0.277) | 0.082 | 0.453 |
|  | rs1048801 | 0.049(-0.090-0.188) | 0.490 | 0.587 |
|  | rs1749316 | -0.040(-0.192-0.113) | 0.610 | 0.610 |
|  | rs1749317 | -0.062(-0.210-0.087) | 0.415 | 0.587 |
|  | rs1925241 | -0.071(-0.205-0.063) | 0.300 | 0.550 |
|  | rs2569715 | -0.095(-0.232-0.041) | 0.172 | 0.457 |
|  | rs2569716 | -0.049(-0.189-0.092) | 0.498 | 0.587 |
|  | rs3745871 | -0.104(-0.242-0.033) | 0.137 | 0.457 |
|  | rs11540761 | -0.013(-0.030-0.004) | 0.069 | 0.453 |
|  | rs11574576 | 0.091(-0.050-0.232) | 0.208 | 0.457 |
|  | rs28366008 | 0.052(-0.111-0.214) | 0.534 | 0.587 |
| Right Putamen | rs731170 | 0.159(0.022-0.296) | **0.024** | 0.129 |
|  | rs1048801 | 0.077(-0.053-0.207) | 0.248 | 0.425 |
|  | rs1749316 | -0.059(-0.202-0.084) | 0.421 | 0.463 |
|  | rs1749317 | -0.046(-0.186-0.093) | 0.516 | 0.516 |
|  | rs1925241 | -0.071(-0.196-0.055) | 0.271 | 0.425 |
|  | rs2569715 | -0.107(-0.234-0.021) | 0.104 | 0.285 |
|  | rs2569716 | -0.064(-0.195-0.068) | 0.343 | 0.463 |
|  | rs3745871 | -0.110(-0.239-0.019) | 0.095 | 0.285 |
|  | rs11540761 | -0.189(-0.349--0.030) | **0.021** | 0.129 |
|  | rs11574576 | 0.059(-0.073-0.191) | 0.384 | 0.463 |
|  | rs28366008 | 0.113(-0.038-0.265) | 0.145 | 0.319 |
| Right Anterior of Putamen | rs731170 | 0.208(0.063-0.353) | **0.005** | 0.057 |
|  | rs1048801 | 0.039(-0.100-0.178) | 0.585 | 0.585 |
|  | rs1749316 | -0.078(-0.230-0.074) | 0.315 | 0.433 |
|  | rs1749317 | -0.098(-0.246-0.049) | 0.193 | 0.304 |
|  | rs1925241 | -0.106(-0.239-0.028) | 0.122 | 0.284 |
|  | rs2569715 | -0.099(-0.235-0.037) | 0.155 | 0.284 |
|  | rs2569716 | -0.057(-0.197-0.082) | 0.423 | 0.465 |
|  | rs3745871 | -0.136(-0.273-0.000) | 0.051 | 0.188 |
|  | rs11540761 | -0.222(-0.391--0.053) | **0.011** | 0.058 |
|  | rs11574576 | 0.059(-0.082-0.200) | 0.411 | 0.465 |
|  | rs28366008 | 0.117(-0.044-0.279) | 0.155 | 0.284 |

CI, Confidence internal; DAT, dopamine transporter; FDR, false discovery rate
