# Supplementary material for: Uncovering the Associations of LILRB4 Genotypes With Parkinson's Disease: From Clinical Traits to Potential Pathologies
Source: CNS Neurosci Ther. 2025 Jul 23;31(7):e70522. doi: 10.1111/cns.70522 (PMC12287542; doi:10.1111/cns.70522)
Supplement: Supplementary file 4 — Table S1. [file CNS-31-e70522-s002.zip › cns70522-sup-0009-TableS14-S16@Supplementary Table 14-16 Model 2_The correlation between LILRB4 loci and DAT-SPECT striatal binding ratios.docx]

**Supplementary Table 14**. Model 2: The correlation between *LILRB4* loci and DAT-SPECT striatal binding ratios.

| Items | SNP | β(95%CI) | P value | FDR-corrected. P |
| --- | --- | --- | --- | --- |
| Left Caudate | rs731170 | 0.107(0.025-0.190) | **0.011** | 0.117 |
|  | rs1048801 | 0.011(-0.067-0.089) | 0.780 | 0.858 |
|  | rs1749316 | -0.052(-0.14-0.035) | 0.240 | 0.440 |
|  | rs1749317 | -0.061(-0.144-0.022) | 0.151 | 0.393 |
|  | rs1925241 | -0.03(-0.105-0.045) | 0.436 | 0.685 |
|  | rs2569715 | -0.055(-0.134-0.025) | 0.179 | 0.393 |
|  | rs2569716 | 0.002(-0.078-0.081) | 0.966 | 0.966 |
|  | rs3745871 | -0.055(-0.132-0.022) | 0.159 | 0.393 |
|  | rs11540761 | -0.092(-0.184-0.001) | 0.053 | 0.290 |
|  | rs11574576 | -0.022(-0.102-0.059) | 0.600 | 0.742 |
|  | rs28366008 | 0.024(-0.068-0.117) | 0.607 | 0.742 |
| Right Caudate | rs731170 | 0.142(0.060-0.225) | **0.001** | **0.008** |
|  | rs1048801 | 0.022(-0.056-0.100) | 0.579 | 0.597 |
|  | rs1749316 | -0.069(-0.157-0.019) | 0.125 | 0.274 |
|  | rs1749317 | -0.087(-0.17--0.003) | **0.042** | 0.155 |
|  | rs1925241 | -0.054(-0.129-0.021) | 0.160 | 0.294 |
|  | rs2569715 | -0.041(-0.121-0.039) | 0.318 | 0.437 |
|  | rs2569716 | -0.022(-0.101-0.058) | 0.597 | 0.597 |
|  | rs3745871 | -0.071(-0.148-0.006) | 0.071 | 0.197 |
|  | rs11540761 | -0.105(-0.198--0.012) | **0.028** | 0.152 |
|  | rs11574576 | -0.027(-0.109-0.054) | 0.512 | 0.597 |
|  | rs28366008 | 0.056(-0.037-0.149) | 0.239 | 0.375 |
| Left Putamen | rs731170 | 0.083(0.007-0.160) | **0.033** | 0.184 |
|  | rs1048801 | 0.012(-0.060-0.084) | 0.748 | 0.748 |
|  | rs1749316 | -0.046(-0.127-0.035) | 0.269 | 0.422 |
|  | rs1749317 | -0.070(-0.148-0.007) | 0.076 | 0.279 |
|  | rs1925241 | -0.024(-0.094-0.046) | 0.498 | 0.608 |
|  | rs2569715 | -0.053(-0.127-0.021) | 0.163 | 0.358 |
|  | rs2569716 | -0.026(-0.100-0.048) | 0.493 | 0.608 |
|  | rs3745871 | -0.047(-0.118-0.025) | 0.200 | 0.366 |
|  | rs11540761 | -0.110(-0.197--0.024) | **0.012** | 0.134 |
|  | rs11574576 | -0.016(-0.092-0.059) | 0.668 | 0.735 |
|  | rs28366008 | 0.070(-0.016-0.156) | 0.110 | 0.302 |
| Left Anterior of  Putamen | rs731170 | 0.094(0.013-0.175) | **0.023** | 0.127 |
|  | rs1048801 | 0.013(-0.063-0.090) | 0.734 | 0.757 |
|  | rs1749316 | -0.040(-0.126-0.046) | 0.361 | 0.497 |
|  | rs1749317 | -0.060(-0.142-0.022) | 0.149 | 0.328 |
|  | rs1925241 | -0.035(-0.109-0.039) | 0.355 | 0.497 |
|  | rs2569715 | -0.060(-0.138-0.019) | 0.135 | 0.328 |
|  | rs2569716 | -0.021(-0.099-0.058) | 0.602 | 0.736 |
|  | rs3745871 | -0.059(-0.134-0.017) | 0.129 | 0.328 |
|  | rs11540761 | -0.106(-0.197--0.015) | **0.023** | 0.127 |
|  | rs11574576 | -0.013(-0.092-0.067) | 0.757 | 0.757 |
|  | rs28366008 | 0.053(-0.038-0.144) | 0.255 | 0.468 |
| Right Putamen | rs731170 | 0.115(0.038-0.192) | **0.003** | **0.037** |
|  | rs1048801 | 0.047(-0.026-0.119) | 0.209 | 0.354 |
|  | rs1749316 | -0.076(-0.158-0.006) | 0.068 | 0.188 |
|  | rs1749317 | -0.044(-0.122-0.034) | 0.266 | 0.365 |
|  | rs1925241 | -0.021(-0.091-0.049) | 0.555 | 0.555 |
|  | rs2569715 | -0.052(-0.127-0.022) | 0.169 | 0.354 |
|  | rs2569716 | -0.023(-0.097-0.052) | 0.547 | 0.555 |
|  | rs3745871 | -0.045(-0.117-0.027) | 0.225 | 0.354 |
|  | rs11540761 | -0.114(-0.2--0.027) | **0.010** | 0.056 |
|  | rs11574576 | -0.032(-0.108-0.043) | 0.401 | 0.490 |
|  | rs28366008 | 0.084(-0.002-0.170) | 0.057 | 0.188 |
| Right Anterior of  Putamen | rs731170 | 0.136(0.054-0.217) | **0.001** | **0.013** |
|  | rs1048801 | 0.034(-0.043-0.111) | 0.391 | 0.430 |
|  | rs1749316 | -0.081(-0.168-0.006) | 0.069 | 0.246 |
|  | rs1749317 | -0.067(-0.150-0.016) | 0.112 | 0.246 |
|  | rs1925241 | -0.037(-0.112-0.038) | 0.333 | 0.410 |
|  | rs2569715 | -0.039(-0.118-0.040) | 0.335 | 0.410 |
|  | rs2569716 | -0.018(-0.097-0.061) | 0.650 | 0.650 |
|  | rs3745871 | -0.055(-0.131-0.022) | 0.161 | 0.296 |
|  | rs11540761 | -0.120(-0.212--0.028) | **0.011** | 0.059 |
|  | rs11574576 | -0.045(-0.126-0.035) | 0.269 | 0.410 |
|  | rs28366008 | 0.076(-0.016-0.168) | 0.105 | 0.246 |

CI, Confidence internal; DAT, dopamine transporter; FDR, false discovery rate

**Supplementary Table 15**. Model 2: The correlation between *LILRB4* loci and DAT-SPECT striatal binding ratios in male.

| Items | SNP | β(95%CI) | P value | FDR-corrected. P |
| --- | --- | --- | --- | --- |
| Left Caudate | rs731170 | 0.104(0.006-0.202) | **0.038** | 0.423 |
|  | rs1048801 | 0.014(-0.079-0.106) | 0.774 | 0.774 |
|  | rs1749316 | -0.043(-0.149-0.063) | 0.425 | 0.584 |
|  | rs1749317 | -0.047(-0.147-0.053) | 0.358 | 0.584 |
|  | rs1925241 | -0.038(-0.127-0.052) | 0.407 | 0.584 |
|  | rs2569715 | -0.045(-0.142-0.053) | 0.370 | 0.584 |
|  | rs2569716 | 0.026(-0.069-0.122) | 0.591 | 0.650 |
|  | rs3745871 | -0.056(-0.148-0.035) | 0.228 | 0.584 |
|  | rs11540761 | -0.063(-0.172-0.046) | 0.257 | 0.584 |
|  | rs11574576 | -0.050(-0.147-0.048) | 0.318 | 0.584 |
|  | rs28366008 | 0.035(-0.077-0.146) | 0.545 | 0.650 |
| Right Caudate | rs731170 | 0.092(-0.008-0.191) | 0.071 | 0.540 |
|  | rs1048801 | 0.041(-0.053-0.135) | 0.393 | 0.540 |
|  | rs1749316 | -0.065(-0.173-0.042) | 0.234 | 0.540 |
|  | rs1749317 | -0.047(-0.148-0.054) | 0.363 | 0.540 |
|  | rs1925241 | -0.014(-0.105-0.077) | 0.758 | 0.834 |
|  | rs2569715 | -0.046(-0.145-0.053) | 0.361 | 0.540 |
|  | rs2569716 | -0.009(-0.106-0.088) | 0.855 | 0.855 |
|  | rs3745871 | -0.023(-0.116-0.070) | 0.632 | 0.772 |
|  | rs11540761 | -0.052(-0.162-0.058) | 0.355 | 0.540 |
|  | rs11574576 | -0.062(-0.162-0.037) | 0.219 | 0.540 |
|  | rs28366008 | 0.055(-0.058-0.168) | 0.344 | 0.540 |
| Left Putamen | rs731170 | 0.073(-0.019-0.166) | 0.122 | 0.447 |
|  | rs1048801 | -0.018(-0.105-0.070) | 0.694 | 0.790 |
|  | rs1749316 | -0.059(-0.159-0.041) | 0.249 | 0.457 |
|  | rs1749317 | -0.075(-0.170-0.019) | 0.119 | 0.447 |
|  | rs1925241 | -0.010(-0.094-0.075) | 0.825 | 0.825 |
|  | rs2569715 | -0.020(-0.112-0.073) | 0.678 | 0.790 |
|  | rs2569716 | 0.017(-0.074-0.107) | 0.718 | 0.790 |
|  | rs3745871 | -0.025(-0.112-0.061) | 0.567 | 0.790 |
|  | rs11540761 | -0.089(-0.192-0.014) | 0.090 | 0.447 |
|  | rs11574576 | -0.061(-0.154-0.031) | 0.195 | 0.457 |
|  | rs28366008 | 0.067(-0.038-0.173) | 0.213 | 0.457 |
| Left Anterior of  Putamen | rs731170 | 0.082(-0.015-0.180) | 0.100 | 0.579 |
|  | rs1048801 | -0.002(-0.094-0.091) | 0.972 | 0.972 |
|  | rs1749316 | -0.042(-0.148-0.063) | 0.431 | 0.592 |
|  | rs1749317 | -0.055(-0.154-0.045) | 0.282 | 0.579 |
|  | rs1925241 | -0.021(-0.110-0.068) | 0.642 | 0.784 |
|  | rs2569715 | -0.047(-0.144-0.049) | 0.338 | 0.579 |
|  | rs2569716 | 0.009(-0.086-0.103) | 0.861 | 0.947 |
|  | rs3745871 | -0.042(-0.133-0.049) | 0.368 | 0.579 |
|  | rs11540761 | -0.080(-0.188-0.028) | 0.149 | 0.579 |
|  | rs11574576 | -0.056(-0.153-0.041) | 0.258 | 0.579 |
|  | rs28366008 | 0.059(-0.053-0.170) | 0.302 | 0.579 |
| Right Putamen | rs731170 | 0.101(0.007-0.195) | **0.035** | 0.390 |
|  | rs1048801 | 0.034(-0.055-0.123) | 0.458 | 0.720 |
|  | rs1749316 | -0.090(-0.192-0.011) | 0.081 | 0.414 |
|  | rs1749317 | -0.043(-0.139-0.052) | 0.376 | 0.689 |
|  | rs1925241 | 0.000(-0.086-0.086) | 0.997 | 0.997 |
|  | rs2569715 | -0.025(-0.118-0.069) | 0.606 | 0.833 |
|  | rs2569716 | 0.014(-0.077-0.105) | 0.762 | 0.838 |
|  | rs3745871 | -0.017(-0.105-0.071) | 0.705 | 0.838 |
|  | rs11540761 | -0.076(-0.18-0.028) | 0.152 | 0.414 |
|  | rs11574576 | -0.070(-0.164-0.023) | 0.141 | 0.414 |
|  | rs28366008 | 0.072(-0.035-0.179) | 0.188 | 0.414 |
| Right Anterior of  Putamen | rs731170 | 0.104(0.005-0.204) | **0.040** | 0.400 |
|  | rs1048801 | 0.036(-0.058-0.130) | 0.452 | 0.710 |
|  | rs1749316 | -0.086(-0.193-0.022) | 0.119 | 0.436 |
|  | rs1749317 | -0.049(-0.150-0.053) | 0.347 | 0.637 |
|  | rs1925241 | -0.005(-0.096-0.086) | 0.915 | 0.915 |
|  | rs2569715 | -0.008(-0.107-0.091) | 0.878 | 0.915 |
|  | rs2569716 | 0.018(-0.079-0.115) | 0.719 | 0.879 |
|  | rs3745871 | -0.018(-0.111-0.075) | 0.705 | 0.879 |
|  | rs11540761 | -0.069(-0.179-0.042) | 0.224 | 0.615 |
|  | rs11574576 | -0.091(-0.190-0.008) | 0.073 | 0.400 |
|  | rs28366008 | 0.057(-0.057-0.170) | 0.329 | 0.637 |

CI, Confidence internal; DAT, dopamine transporter; FDR, false discovery rate

**Supplementary Table 16**. Model 2: The correlation between *LILRB4* loci and DAT-SPECT striatal binding ratios in female.

| Items | SNP | β(95%CI) | P value | FDR-corrected. P |
| --- | --- | --- | --- | --- |
| Left Caudate | rs731170 | 0.108(-0.039-0.255) | 0.152 | 0.819 |
|  | rs1048801 | 0.018(-0.122-0.157) | 0.805 | 0.915 |
|  | rs1749316 | -0.059(-0.211-0.093) | 0.447 | 0.819 |
|  | rs1749317 | -0.071(-0.219-0.077) | 0.348 | 0.819 |
|  | rs1925241 | -0.015(-0.149-0.120) | 0.832 | 0.915 |
|  | rs2569715 | -0.073(-0.210-0.064) | 0.296 | 0.819 |
|  | rs2569716 | -0.029(-0.170-0.112) | 0.686 | 0.915 |
|  | rs3745871 | -0.054(-0.192-0.084) | 0.444 | 0.819 |
|  | rs11540761 | -0.146(-0.316-0.025) | 0.095 | 0.819 |
|  | rs11574576 | 0.037(-0.105-0.179) | 0.614 | 0.915 |
|  | rs28366008 | -0.001(-0.163-0.161) | 0.991 | 0.991 |
| Right Caudate | rs731170 | 0.226(0.081-0.371) | **0.002** | **0.027** |
|  | rs1048801 | -0.004(-0.143-0.134) | 0.951 | 0.951 |
|  | rs1749316 | -0.071(-0.222-0.081) | 0.361 | 0.662 |
|  | rs1749317 | -0.144(-0.291-0.003) | 0.055 | 0.152 |
|  | rs1925241 | -0.121(-0.254-0.011) | 0.074 | 0.164 |
|  | rs2569715 | -0.037(-0.174-0.100) | 0.595 | 0.683 |
|  | rs2569716 | -0.039(-0.179-0.102) | 0.590 | 0.683 |
|  | rs3745871 | -0.154(-0.290--0.018) | **0.028** | 0.101 |
|  | rs11540761 | -0.205(-0.374--0.036) | **0.018** | 0.101 |
|  | rs11574576 | 0.036(-0.106-0.177) | 0.621 | 0.683 |
|  | rs28366008 | 0.054(-0.107-0.216) | 0.509 | 0.683 |
| Left Putamen | rs731170 | 0.096(-0.038-0.231) | 0.162 | 0.422 |
|  | rs1048801 | 0.069(-0.057-0.196) | 0.285 | 0.488 |
|  | rs1749316 | -0.021(-0.160-0.118) | 0.770 | 0.770 |
|  | rs1749317 | -0.052(-0.187-0.083) | 0.453 | 0.498 |
|  | rs1925241 | -0.048(-0.170-0.074) | 0.443 | 0.498 |
|  | rs2569715 | -0.106(-0.230-0.019) | 0.097 | 0.422 |
|  | rs2569716 | -0.091(-0.219-0.038) | 0.167 | 0.422 |
|  | rs3745871 | -0.084(-0.209-0.042) | 0.192 | 0.422 |
|  | rs11540761 | -0.151(-0.306-0.005) | 0.059 | 0.422 |
|  | rs11574576 | 0.063(-0.066-0.193) | 0.338 | 0.488 |
|  | rs28366008 | 0.07(-0.078-0.217) | 0.355 | 0.488 |
| Left Anterior of  Putamen | rs731170 | 0.109(-0.034-0.252) | 0.137 | 0.593 |
|  | rs1048801 | 0.048(-0.087-0.184) | 0.485 | 0.593 |
|  | rs1749316 | -0.030(-0.178-0.118) | 0.691 | 0.691 |
|  | rs1749317 | -0.056(-0.201-0.088) | 0.445 | 0.593 |
|  | rs1925241 | -0.057(-0.187-0.073) | 0.393 | 0.593 |
|  | rs2569715 | -0.083(-0.216-0.050) | 0.223 | 0.593 |
|  | rs2569716 | -0.062(-0.199-0.075) | 0.378 | 0.593 |
|  | rs3745871 | -0.087(-0.221-0.046) | 0.201 | 0.593 |
|  | rs11540761 | -0.156(-0.322-0.010) | 0.066 | 0.593 |
|  | rs11574576 | 0.069(-0.069-0.207) | 0.329 | 0.593 |
|  | rs28366008 | 0.037(-0.121-0.194) | 0.648 | 0.691 |
| Right Putamen | rs731170 | 0.137(0.004-0.271) | **0.045** | 0.247 |
|  | rs1048801 | 0.075(-0.051-0.202) | 0.244 | 0.383 |
|  | rs1749316 | -0.050(-0.188-0.089) | 0.483 | 0.591 |
|  | rs1749317 | -0.040(-0.175-0.095) | 0.565 | 0.596 |
|  | rs1925241 | -0.057(-0.178-0.065) | 0.363 | 0.500 |
|  | rs2569715 | -0.096(-0.221-0.028) | 0.130 | 0.383 |
|  | rs2569716 | -0.080(-0.208-0.048) | 0.222 | 0.383 |
|  | rs3745871 | -0.093(-0.218-0.032) | 0.147 | 0.383 |
|  | rs11540761 | -0.186(-0.340--0.031) | **0.019** | 0.211 |
|  | rs11574576 | 0.035(-0.094-0.164) | 0.596 | 0.596 |
|  | rs28366008 | 0.099(-0.048-0.246) | 0.187 | 0.383 |
| Right Anterior of  Putamen | rs731170 | 0.186(0.045-0.328) | **0.011** | 0.058 |
|  | rs1048801 | 0.037(-0.098-0.172) | 0.595 | 0.624 |
|  | rs1749316 | -0.069(-0.216-0.079) | 0.362 | 0.443 |
|  | rs1749317 | -0.092(-0.235-0.052) | 0.213 | 0.334 |
|  | rs1925241 | -0.091(-0.221-0.039) | 0.169 | 0.334 |
|  | rs2569715 | -0.089(-0.222-0.044) | 0.190 | 0.334 |
|  | rs2569716 | -0.074(-0.211-0.063) | 0.290 | 0.398 |
|  | rs3745871 | -0.119(-0.252-0.014) | 0.082 | 0.300 |
|  | rs11540761 | -0.218(-0.382--0.054) | **0.010** | 0.058 |
|  | rs11574576 | 0.035(-0.103-0.172) | 0.624 | 0.624 |
|  | rs28366008 | 0.103(-0.053-0.260) | 0.198 | 0.334 |

CI, Confidence internal; DAT, dopamine transporter; FDR, false discovery rate
