# Supplementary material for: Uncovering the Associations of LILRB4 Genotypes With Parkinson's Disease: From Clinical Traits to Potential Pathologies
Source: CNS Neurosci Ther. 2025 Jul 23;31(7):e70522. doi: 10.1111/cns.70522 (PMC12287542; doi:10.1111/cns.70522)
Supplement: Supplementary file 4 — Table S1. [file CNS-31-e70522-s002.zip › cns70522-sup-0010-TableS17-S19@Supplementary Table 17-19 Model 1_The correlation between LILRB4 loci and DTI FA values.docx]

**Supplementary Table 17**. Model 1: The correlation between *LILRB4* loci and DTI FA values.

| Items | SNP | β(95%CI) | P | FDR-corrected. P |
| --- | --- | --- | --- | --- |
| Left Caudal | rs731170 | 0.006(-0.006-0.018) | 0.332 | 0.788 |
|  | rs1048801 | -0.002(-0.013-0.009) | 0.774 | 0.788 |
|  | rs1749316 | -0.009(-0.021-0.003) | 0.148 | 0.788 |
|  | rs1749317 | -0.003(-0.014-0.009) | 0.658 | 0.788 |
|  | rs1925241 | 0.003(-0.008-0.014) | 0.604 | 0.788 |
|  | rs2569715 | 0.004(-0.007-0.016) | 0.459 | 0.788 |
|  | rs2569716 | 0.004(-0.007-0.016) | 0.446 | 0.788 |
|  | rs3745871 | 0.003(-0.008-0.015) | 0.551 | 0.788 |
|  | rs11540761 | -0.004(-0.017-0.009) | 0.549 | 0.788 |
|  | rs11574576 | -0.002(-0.013-0.010) | 0.788 | 0.788 |
|  | rs28366008 | 0.005(-0.008-0.018) | 0.453 | 0.788 |
| Left Middle | rs731170 | 0.000(-0.014-0.014) | 0.986 | 0.986 |
|  | rs1048801 | 0.003(-0.010-0.016) | 0.658 | 0.804 |
|  | rs1749316 | -0.005(-0.019-0.009) | 0.465 | 0.775 |
|  | rs1749317 | 0.000(-0.014-0.014) | 0.968 | 0.986 |
|  | rs1925241 | 0.005(-0.008-0.019) | 0.418 | 0.775 |
|  | rs2569715 | 0.005(-0.008-0.019) | 0.441 | 0.775 |
|  | rs2569716 | 0.008(-0.005-0.022) | 0.220 | 0.775 |
|  | rs3745871 | 0.005(-0.008-0.019) | 0.431 | 0.775 |
|  | rs11540761 | 0.014(-0.002-0.029) | 0.640 | 0.804 |
|  | rs11574576 | 0.012(-0.002-0.025) | 0.101 | 0.775 |
|  | rs28366008 | 0.005(-0.010-0.021) | 0.493 | 0.775 |
| Left Rostral | rs731170 | 0.007(-0.008-0.022) | 0.348 | 0.820 |
|  | rs1048801 | 0.007(-0.007-0.020) | 0.337 | 0.820 |
|  | rs1749316 | -0.005(-0.019-0.01) | 0.546 | 0.820 |
|  | rs1749317 | 0.002(-0.013-0.016) | 0.820 | 0.820 |
|  | rs1925241 | -0.003(-0.017-0.011) | 0.702 | 0.820 |
|  | rs2569715 | 0.004(-0.01-0.018) | 0.604 | 0.820 |
|  | rs2569716 | 0.010(-0.004-0.024) | 0.153 | 0.820 |
|  | rs3745871 | -0.002(-0.016-0.012) | 0.814 | 0.820 |
|  | rs11540761 | -0.007(-0.023-0.009) | 0.404 | 0.820 |
|  | rs11574576 | 0.003(-0.011-0.018) | 0.655 | 0.820 |
|  | rs28366008 | 0.003(-0.013-0.019) | 0.672 | 0.820 |
| Right Caudal | rs731170 | 0.012(0.000-0.024) | 0.051 | 0.202 |
|  | rs1048801 | 0.001(-0.010-0.012) | 0.331 | 0.520 |
|  | rs1749316 | 0.002(-0.010-0.014) | 0.775 | 0.889 |
|  | rs1749317 | -0.006(-0.018-0.006) | 0.311 | 0.520 |
|  | rs1925241 | -0.011(-0.022-0.000) | **0.046** | 0.202 |
|  | rs2569715 | 0.000(-0.011-0.011) | 0.993 | 0.993 |
|  | rs2569716 | 0.003(-0.008-0.015) | 0.545 | 0.749 |
|  | rs3745871 | -0.010(-0.021-0.002) | 0.098 | 0.270 |
|  | rs11540761 | -0.013(-0.025-0.000) | 0.055 | 0.202 |
|  | rs11574576 | -0.007(-0.018-0.005) | 0.256 | 0.520 |
|  | rs28366008 | -0.002(-0.014-0.011) | 0.808 | 0.889 |
| Right Middle | rs731170 | 0.014(0.000-0.027) | **0.045** | 0.122 |
|  | rs1048801 | -0.002(-0.014-0.011) | 0.761 | 0.975 |
|  | rs1749316 | 0.010(-0.004-0.023) | 0.166 | 0.365 |
|  | rs1749317 | -0.001(-0.015-0.012) | 0.829 | 0.975 |
|  | rs1925241 | -0.021(-0.033--0.009) | **0.001** | **0.012** |
|  | rs2569715 | 0.002(-0.01-0.015) | 0.707 | 0.975 |
|  | rs2569716 | 0.000(-0.013-0.013) | 0.970 | 0.975 |
|  | rs3745871 | -0.018(-0.03--0.005) | **0.006** | 0.032 |
|  | rs11540761 | -0.015(-0.03--0.001) | 0.037 | 0.122 |
|  | rs11574576 | -0.003(-0.016-0.011) | 0.711 | 0.975 |
|  | rs28366008 | 0.000(-0.014-0.015) | 0.975 | 0.975 |
| Right Rostral | rs731170 | 0.015(0.001-0.029) | **0.039** | 0.087 |
|  | rs1048801 | 0.004(-0.009-0.017) | 0.578 | 0.837 |
|  | rs1749316 | 0.016(0.002-0.030) | **0.022** | 0.077 |
|  | rs1749317 | 0.003(-0.011-0.017) | 0.690 | 0.837 |
|  | rs1925241 | -0.024(-0.037--0.011) | **<0.001** | **0.002** |
|  | rs2569715 | 0.001(-0.013-0.014) | 0.911 | 0.911 |
|  | rs2569716 | -0.002(-0.015-0.011) | 0.760 | 0.837 |
|  | rs3745871 | -0.026(-0.039--0.013) | **<0.001** | **0.002** |
|  | rs11540761 | -0.017(-0.032--0.002) | **0.028** | 0.077 |
|  | rs11574576 | -0.002(-0.016-0.012) | 0.761 | 0.837 |
|  | rs28366008 | 0.004(-0.011-0.019) | 0.600 | 0.837 |

CI, confidence internal; DTI, diffusion tensor imaging; FDR, false discovery rate

**Supplementary Table 18**. Model 1: The correlation between *LILRB4* loci and DTI FA values in male.

| Items | SNP | β(95%CI) | P | FDR-corrected. P |
| --- | --- | --- | --- | --- |
| Left Caudal | rs731170 | 0.009(-0.007-0.025) | 0.253 | 0.814 |
|  | rs1048801 | -0.005(-0.02-0.009) | 0.471 | 0.814 |
|  | rs1749316 | -0.006(-0.023-0.011) | 0.501 | 0.814 |
|  | rs1749317 | -0.006(-0.022-0.009) | 0.434 | 0.814 |
|  | rs1925241 | 0.002(-0.013-0.017) | 0.800 | 0.814 |
|  | rs2569715 | 0.010(-0.005-0.026) | 0.173 | 0.814 |
|  | rs2569716 | 0.003(-0.013-0.018) | 0.739 | 0.814 |
|  | rs3745871 | -0.002(-0.017-0.013) | 0.814 | 0.814 |
|  | rs11540761 | -0.007(-0.024-0.010) | 0.412 | 0.814 |
|  | rs11574576 | -0.004(-0.02-0.011) | 0.600 | 0.814 |
|  | rs28366008 | 0.003(-0.014-0.020) | 0.736 | 0.814 |
| Left Middle | rs731170 | 0.008(-0.011-0.026) | 0.415 | 0.994 |
|  | rs1048801 | 0.002(-0.015-0.019) | 0.833 | 0.994 |
|  | rs1749316 | -0.003(-0.022-0.016) | 0.766 | 0.994 |
|  | rs1749317 | 0.000(-0.018-0.018) | 0.972 | 0.994 |
|  | rs1925241 | 0.000(-0.017-0.017) | 0.994 | 0.994 |
|  | rs2569715 | 0.010(-0.007-0.027) | 0.261 | 0.994 |
|  | rs2569716 | 0.003(-0.015-0.021) | 0.768 | 0.994 |
|  | rs3745871 | -0.002(-0.020-0.015) | 0.785 | 0.994 |
|  | rs11540761 | -0.005(-0.024-0.014) | 0.608 | 0.994 |
|  | rs11574576 | 0.006(-0.012-0.023) | 0.546 | 0.994 |
|  | rs28366008 | 0.002(-0.018-0.021) | 0.878 | 0.994 |
| Left Rostral | rs731170 | 0.012(-0.008-0.031) | 0.249 | 0.859 |
|  | rs1048801 | 0.003(-0.015-0.021) | 0.712 | 0.859 |
|  | rs1749316 | -0.001(-0.022-0.020) | 0.919 | 0.919 |
|  | rs1749317 | 0.003(-0.017-0.022) | 0.781 | 0.859 |
|  | rs1925241 | -0.007(-0.025-0.012) | 0.476 | 0.859 |
|  | rs2569715 | 0.007(-0.011-0.026) | 0.439 | 0.859 |
|  | rs2569716 | 0.004(-0.016-0.023) | 0.714 | 0.859 |
|  | rs3745871 | -0.004(-0.023-0.014) | 0.635 | 0.859 |
|  | rs11540761 | -0.013(-0.033-0.007) | 0.211 | 0.859 |
|  | rs11574576 | -0.006(-0.025-0.013) | 0.540 | 0.859 |
|  | rs28366008 | 0.010(-0.01-0.031) | 0.317 | 0.859 |
| Right Caudal | rs731170 | 0.009(-0.006-0.024) | 0.254 | 0.626 |
|  | rs1048801 | 0.004(-0.010-0.018) | 0.580 | 0.798 |
|  | rs1749316 | 0.002(-0.014-0.019) | 0.765 | 0.935 |
|  | rs1749317 | -0.011(-0.026-0.004) | 0.148 | 0.626 |
|  | rs1925241 | -0.009(-0.023-0.006) | 0.238 | 0.626 |
|  | rs2569715 | 0.000(-0.015-0.014) | 0.972 | 0.982 |
|  | rs2569716 | 0.007(-0.008-0.022) | 0.374 | 0.626 |
|  | rs3745871 | -0.006(-0.021-0.008) | 0.399 | 0.626 |
|  | rs11540761 | -0.019(-0.034--0.003) | **0.022** | 0.238 |
|  | rs11574576 | -0.007(-0.022-0.008) | 0.355 | 0.626 |
|  | rs28366008 | 0.000(-0.016-0.016) | 0.982 | 0.982 |
| Right Middle | rs731170 | 0.011(-0.007-0.030) | 0.238 | 0.528 |
|  | rs1048801 | -0.004(-0.021-0.013) | 0.667 | 0.815 |
|  | rs1749316 | 0.012(-0.008-0.031) | 0.240 | 0.528 |
|  | rs1749317 | -0.004(-0.023-0.014) | 0.649 | 0.815 |
|  | rs1925241 | -0.019(-0.036--0.001) | **0.036** | 0.266 |
|  | rs2569715 | 0.005(-0.013-0.023) | 0.576 | 0.815 |
|  | rs2569716 | 0.008(-0.010-0.026) | 0.392 | 0.719 |
|  | rs3745871 | -0.014(-0.032-0.003) | 0.108 | 0.396 |
|  | rs11540761 | -0.019(-0.038-0.000) | **0.048** | 0.266 |
|  | rs11574576 | 0.002(-0.016-0.020) | 0.819 | 0.834 |
|  | rs28366008 | 0.002(-0.017-0.021) | 0.834 | 0.834 |
| Right Rostral | rs731170 | 0.015(-0.003-0.033) | 0.108 | 0.238 |
|  | rs1048801 | 0.007(-0.009-0.024) | 0.389 | 0.611 |
|  | rs1749316 | 0.018(-0.001-0.037) | 0.060 | 0.166 |
|  | rs1749317 | 0.001(-0.017-0.019) | 0.896 | 0.986 |
|  | rs1925241 | -0.025(-0.041--0.008) | **0.004** | **0.021** |
|  | rs2569715 | 0.000(-0.017-0.017) | 0.986 | 0.986 |
|  | rs2569716 | 0.004(-0.014-0.021) | 0.671 | 0.820 |
|  | rs3745871 | -0.024(-0.041--0.008) | **0.005** | **0.021** |
|  | rs11540761 | -0.026(-0.045--0.008) | **0.006** | **0.021** |
|  | rs11574576 | 0.007(-0.011-0.024) | 0.461 | 0.634 |
|  | rs28366008 | 0.009(-0.01-0.028) | 0.349 | 0.611 |

CI, Confidence internal; DTI, diffusion tensor imaging; FDR, false discovery rate

**Supplementary Table 19**. Model 1: The correlation between *LILRB4* loci and DTI FA values in female.

| Items | SNP | β(95%CI) | P | FDR-corrected. P |
| --- | --- | --- | --- | --- |
| Left Caudal | rs731170 | 0.000(-0.017-0.017) | 0.994 | 0.994 |
|  | rs1048801 | 0.006(-0.011-0.023) | 0.494 | 0.857 |
|  | rs1749316 | -0.014(-0.030-0.003) | 0.103 | 0.605 |
|  | rs1749317 | 0.004(-0.013-0.021) | 0.656 | 0.857 |
|  | rs1925241 | 0.005(-0.011-0.021) | 0.563 | 0.857 |
|  | rs2569715 | -0.007(-0.023-0.009) | 0.409 | 0.857 |
|  | rs2569716 | 0.007(-0.009-0.023) | 0.367 | 0.857 |
|  | rs3745871 | 0.014(-0.003-0.031) | 0.110 | 0.605 |
|  | rs11540761 | 0.003(-0.018-0.023) | 0.779 | 0.857 |
|  | rs11574576 | 0.003(-0.014-0.021) | 0.718 | 0.857 |
|  | rs28366008 | 0.010(-0.011-0.030) | 0.349 | 0.857 |
| Left Middle | rs731170 | -0.014(-0.036-0.007) | 0.200 | 0.440 |
|  | rs1048801 | 0.005(-0.017-0.026) | 0.674 | 0.891 |
|  | rs1749316 | -0.007(-0.028-0.014) | 0.510 | 0.801 |
|  | rs1749317 | 0.000(-0.022-0.021) | 0.966 | 0.989 |
|  | rs1925241 | 0.015(-0.005-0.036) | 0.139 | 0.382 |
|  | rs2569715 | -0.004(-0.025-0.017) | 0.729 | 0.891 |
|  | rs2569716 | 0.016(-0.003-0.036) | 0.110 | 0.382 |
|  | rs3745871 | 0.02(-0.001-0.041) | 0.061 | 0.337 |
|  | rs11540761 | 0.000(-0.026-0.026) | 0.989 | 0.989 |
|  | rs11574576 | 0.021(0.000-0.043) | 0.057 | 0.337 |
|  | rs28366008 | 0.013(-0.013-0.038) | 0.331 | 0.607 |
| Left Rostral | rs731170 | -0.001(-0.022-0.020) | 0.924 | 0.958 |
|  | rs1048801 | 0.013(-0.007-0.034) | 0.210 | 0.770 |
|  | rs1749316 | -0.009(-0.030-0.011) | 0.364 | 0.801 |
|  | rs1749317 | -0.001(-0.022-0.021) | 0.958 | 0.958 |
|  | rs1925241 | 0.005(-0.016-0.025) | 0.662 | 0.946 |
|  | rs2569715 | -0.003(-0.023-0.017) | 0.774 | 0.946 |
|  | rs2569716 | 0.021(0.002-0.040) | **0.037** | 0.343 |
|  | rs3745871 | 0.004(-0.017-0.025) | 0.731 | 0.946 |
|  | rs11540761 | 0.008(-0.018-0.033) | 0.554 | 0.946 |
|  | rs11574576 | 0.020(-0.001-0.042) | 0.062 | 0.343 |
|  | rs28366008 | -0.013(-0.038-0.012) | 0.325 | 0.801 |
| Right Caudal | rs731170 | 0.018(0.000-0.036) | 0.059 | 0.334 |
|  | rs1048801 | 0.009(-0.009-0.027) | 0.320 | 0.880 |
|  | rs1749316 | -0.001(-0.019-0.017) | 0.932 | 0.955 |
|  | rs1749317 | 0.005(-0.014-0.023) | 0.629 | 0.955 |
|  | rs1925241 | -0.016(-0.033-0.001) | 0.067 | 0.334 |
|  | rs2569715 | 0.001(-0.017-0.019) | 0.938 | 0.955 |
|  | rs2569716 | -0.001(-0.018-0.017) | 0.941 | 0.955 |
|  | rs3745871 | -0.016(-0.034-0.002) | 0.091 | 0.334 |
|  | rs11540761 | 0.001(-0.021-0.023) | 0.955 | 0.955 |
|  | rs11574576 | -0.005(-0.024-0.014) | 0.605 | 0.955 |
|  | rs28366008 | -0.005(-0.026-0.017) | 0.685 | 0.955 |
| Right Middle | rs731170 | 0.019(0.002-0.036) | **0.031** | 0.114 |
|  | rs1048801 | 0.002(-0.015-0.019) | 0.810 | 0.819 |
|  | rs1749316 | 0.006(-0.011-0.022) | 0.516 | 0.816 |
|  | rs1749317 | 0.005(-0.013-0.022) | 0.593 | 0.816 |
|  | rs1925241 | -0.025(-0.040--0.010) | **0.002** | **0.021** |
|  | rs2569715 | -0.001(-0.018-0.015) | 0.819 | 0.819 |
|  | rs2569716 | -0.011(-0.027-0.005) | 0.167 | 0.460 |
|  | rs3745871 | -0.025(-0.041--0.009) | **0.004** | **0.021** |
|  | rs11540761 | -0.007(-0.027-0.014) | 0.519 | 0.816 |
|  | rs11574576 | -0.010(-0.028-0.007) | 0.247 | 0.543 |
|  | rs28366008 | -0.003(-0.024-0.017) | 0.766 | 0.819 |
| Right Rostral | rs731170 | 0.017(-0.005-0.038) | 0.132 | 0.437 |
|  | rs1048801 | -0.002(-0.023-0.019) | 0.845 | 0.845 |
|  | rs1749316 | 0.011(-0.010-0.031) | 0.299 | 0.632 |
|  | rs1749317 | 0.009(-0.012-0.031) | 0.402 | 0.632 |
|  | rs1925241 | -0.024(-0.043--0.004) | **0.022** | 0.119 |
|  | rs2569715 | 0.003(-0.018-0.023) | 0.796 | 0.845 |
|  | rs2569716 | -0.009(-0.029-0.011) | 0.368 | 0.632 |
|  | rs3745871 | -0.028(-0.048--0.007) | **0.010** | 0.111 |
|  | rs11540761 | 0.003(-0.022-0.029) | 0.803 | 0.845 |
|  | rs11574576 | -0.016(-0.037-0.006) | 0.159 | 0.437 |
|  | rs28366008 | -0.005(-0.007--0.002) | 0.720 | 0.845 |

CI, Confidence internal; DTI, diffusion tensor imaging;FDR, false discovery rate
