# Supplementary material for: Uncovering the Associations of LILRB4 Genotypes With Parkinson's Disease: From Clinical Traits to Potential Pathologies
Source: CNS Neurosci Ther. 2025 Jul 23;31(7):e70522. doi: 10.1111/cns.70522 (PMC12287542; doi:10.1111/cns.70522)
Supplement: Supplementary file 4 — Table S1. [file CNS-31-e70522-s002.zip › cns70522-sup-0011-TableS20-S22@Supplementary Table 20-22 Model 2_The correlation between LILRB4 loci and DTI FA values.docx]

**Supplementary Table 20**. Model 2: The correlation between *LILRB4* loci and DTI FA values.

| Items | SNP | β(95%CI) | P value | FDR-corrected. P |
| --- | --- | --- | --- | --- |
| Left Rostral | rs731170 | 0.005(-0.009-0.02) | 0.466 | 0.997 |
|  | rs1048801 | 0.007(-0.006-0.021) | 0.305 | 0.997 |
|  | rs1749316 | -0.004(-0.019-0.011) | 0.574 | 0.997 |
|  | rs1749317 | 0(-0.014-0.015) | 0.976 | 0.997 |
|  | rs1925241 | -0.002(-0.015-0.012) | 0.813 | 0.997 |
|  | rs2569715 | 0.003(-0.011-0.017) | 0.645 | 0.997 |
|  | rs2569716 | 0.01(-0.004-0.024) | 0.163 | 0.997 |
|  | rs3745871 | -0.001(-0.015-0.013) | 0.942 | 0.997 |
|  | rs11540761 | -0.005(-0.021-0.011) | 0.546 | 0.997 |
|  | rs11574576 | 0.004(-0.011-0.018) | 0.622 | 0.997 |
|  | rs28366008 | 0(-0.016-0.016) | 0.997 | 0.997 |
| Left Middle | rs731170 | -0.001(-0.015-0.014) | 0.926 | 0.926 |
|  | rs1048801 | 0.003(-0.01-0.016) | 0.619 | 0.926 |
|  | rs1749316 | -0.006(-0.02-0.009) | 0.450 | 0.876 |
|  | rs1749317 | -0.001(-0.015-0.013) | 0.869 | 0.926 |
|  | rs1925241 | 0.006(-0.007-0.02) | 0.353 | 0.876 |
|  | rs2569715 | 0.005(-0.008-0.018) | 0.478 | 0.876 |
|  | rs2569716 | 0.008(-0.005-0.022) | 0.220 | 0.876 |
|  | rs3745871 | 0.006(-0.007-0.02) | 0.363 | 0.876 |
|  | rs11540761 | -0.002(-0.018-0.013) | 0.773 | 0.926 |
|  | rs11574576 | 0.012(-0.002-0.026) | 0.100 | 0.876 |
|  | rs28366008 | 0.003(-0.012-0.019) | 0.685 | 0.926 |
| Left Caudal | rs731170 | 0.006(-0.006-0.018) | 0.336 | 0.756 |
|  | rs1048801 | -0.002(-0.013-0.01) | 0.792 | 0.792 |
|  | rs1749316 | -0.009(-0.021-0.003) | 0.137 | 0.756 |
|  | rs1749317 | -0.004(-0.016-0.008) | 0.515 | 0.756 |
|  | rs1925241 | 0.003(-0.008-0.015) | 0.576 | 0.756 |
|  | rs2569715 | 0.004(-0.007-0.015) | 0.495 | 0.756 |
|  | rs2569716 | 0.005(-0.007-0.016) | 0.437 | 0.756 |
|  | rs3745871 | 0.004(-0.008-0.015) | 0.530 | 0.756 |
|  | rs11540761 | -0.003(-0.016-0.01) | 0.618 | 0.756 |
|  | rs11574576 | -0.002(-0.014-0.01) | 0.769 | 0.792 |
|  | rs28366008 | 0.004(-0.009-0.017) | 0.565 | 0.756 |
| Right Rostral | rs731170 | 0.015(0.001-0.029) | **0.041** | 0.091 |
|  | rs1048801 | 0.004(-0.009-0.017) | 0.582 | 0.800 |
|  | rs1749316 | 0.017(0.003-0.031) | **0.019** | 0.064 |
|  | rs1749317 | 0.005(-0.009-0.019) | 0.517 | 0.800 |
|  | rs1925241 | -0.024(-0.037--0.011) | **0.000** | **0.002** |
|  | rs2569715 | 0.001(-0.012-0.015) | 0.862 | 0.862 |
|  | rs2569716 | -0.002(-0.016-0.011) | 0.741 | 0.849 |
|  | rs3745871 | -0.026(-0.039--0.013) | **0.000** | **0.002** |
|  | rs11540761 | -0.018(-0.033--0.003) | **0.023** | 0.064 |
|  | rs11574576 | -0.002(-0.016-0.012) | 0.772 | 0.849 |
|  | rs28366008 | 0.006(-0.01-0.021) | 0.487 | 0.800 |
| Right Middle | rs731170 | 0.014(0-0.027) | **0.049** | 0.135 |
|  | rs1048801 | -0.001(-0.014-0.011) | 0.835 | 0.975 |
|  | rs1749316 | 0.009(-0.005-0.023) | 0.198 | 0.436 |
|  | rs1749317 | 0(-0.014-0.013) | 0.975 | 0.975 |
|  | rs1925241 | -0.02(-0.033--0.008) | **0.002** | **0.020** |
|  | rs2569715 | 0.003(-0.01-0.015) | 0.691 | 0.975 |
|  | rs2569716 | 0(-0.012-0.013) | 0.941 | 0.975 |
|  | rs3745871 | -0.017(-0.03--0.004) | **0.010** | 0.053 |
|  | rs11540761 | -0.015(-0.03--0.001) | **0.043** | 0.135 |
|  | rs11574576 | -0.004(-0.017-0.01) | 0.608 | 0.975 |
|  | rs28366008 | 0.001(-0.014-0.016) | 0.889 | 0.975 |
| Right Caudal | rs731170 | 0.012(0.001-0.024) | **0.041** | 0.241 |
|  | rs1048801 | 0.006(-0.005-0.017) | 0.265 | 0.485 |
|  | rs1749316 | 0(-0.012-0.012) | 0.970 | 0.995 |
|  | rs1749317 | -0.005(-0.017-0.007) | 0.377 | 0.593 |
|  | rs1925241 | -0.011(-0.022-0.001) | 0.066 | 0.241 |
|  | rs2569715 | 0(-0.011-0.011) | 0.995 | 0.995 |
|  | rs2569716 | 0.004(-0.007-0.016) | 0.466 | 0.640 |
|  | rs3745871 | -0.009(-0.02-0.003) | 0.137 | 0.342 |
|  | rs11540761 | -0.012(-0.025-0) | 0.060 | 0.241 |
|  | rs11574576 | -0.009(-0.02-0.003) | 0.155 | 0.342 |
|  | rs28366008 | 0(-0.013-0.013) | 0.988 | 0.995 |

CI, confidence internal; DTI, diffusion tensor imaging; FDR, false discovery rate

**Supplementary Table 21**. Model 2: The correlation between *LILRB4* loci and DTI FA values in male.

| Items | SNP | β(95%CI) | P value | FDR-corrected. P |
| --- | --- | --- | --- | --- |
| Left Rostral | rs731170 | 0.010(-0.006-0.027) | 0.220 | 0.968 |
|  | rs1048801 | -0.006(-0.021-0.008) | 0.400 | 0.968 |
|  | rs1749316 | -0.006(-0.023-0.011) | 0.488 | 0.968 |
|  | rs1749317 | -0.010(-0.026-0.007) | 0.251 | 0.997 |
|  | rs1925241 | 0.002(-0.013-0.017) | 0.834 | 0.968 |
|  | rs2569715 | 0.011(-0.004-0.026) | 0.158 | 0.968 |
|  | rs2569716 | 0.003(-0.013-0.018) | 0.733 | 0.968 |
|  | rs3745871 | -0.002(-0.017-0.013) | 0.762 | 0.968 |
|  | rs11540761 | -0.006(-0.023-0.010) | 0.450 | 0.968 |
|  | rs11574576 | -0.004(-0.020-0.012) | 0.637 | 0.968 |
|  | rs28366008 | 0.003(-0.017-0.023) | 0.778 | 0.968 |
| Left Middle | rs731170 | 0.007(-0.012-0.026) | 0.466 | 0.957 |
|  | rs1048801 | 0.002(-0.015-0.018) | 0.860 | 0.957 |
|  | rs1749316 | -0.002(-0.021-0.018) | 0.870 | 0.957 |
|  | rs1749317 | -0.003(-0.022-0.015) | 0.736 | 0.957 |
|  | rs1925241 | 0.000(-0.017-0.017) | 0.984 | 0.984 |
|  | rs2569715 | 0.009(-0.008-0.027) | 0.288 | 0.957 |
|  | rs2569716 | 0.002(-0.016-0.02) | 0.820 | 0.957 |
|  | rs3745871 | -0.003(-0.02-0.014) | 0.753 | 0.957 |
|  | rs11540761 | -0.004(-0.023-0.015) | 0.682 | 0.957 |
|  | rs11574576 | 0.007(-0.011-0.025) | 0.463 | 0.957 |
|  | rs28366008 | 0.005(-0.016-0.026) | 0.624 | 0.957 |
| Left Caudal | rs731170 | 0.010(-0.010-0.030) | 0.338 | 0.834 |
|  | rs1048801 | 0.004(-0.014-0.021) | 0.694 | 0.834 |
|  | rs1749316 | 0.002(-0.019-0.022) | 0.880 | 0.834 |
|  | rs1749317 | 0.000(-0.020-0.020) | 0.997 | 0.834 |
|  | rs1925241 | -0.007(-0.025-0.011) | 0.461 | 0.834 |
|  | rs2569715 | 0.006(-0.013-0.024) | 0.530 | 0.834 |
|  | rs2569716 | 0.002(-0.017-0.021) | 0.808 | 0.834 |
|  | rs3745871 | -0.005(-0.023-0.014) | 0.614 | 0.834 |
|  | rs11540761 | -0.012(-0.032-0.009) | 0.260 | 0.834 |
|  | rs11574576 | -0.004(-0.023-0.015) | 0.661 | 0.834 |
|  | rs28366008 | -0.002(-0.022-0.018) | 0.832 | 0.834 |
| Right Rostral | rs731170 | 0.009(-0.007-0.025) | 0.255 | 0.202 |
|  | rs1048801 | 0.005(-0.010-0.019) | 0.527 | 0.667 |
|  | rs1749316 | 0.002(-0.014-0.019) | 0.778 | 0.191 |
|  | rs1749317 | -0.010(-0.025-0.006) | 0.223 | 0.869 |
|  | rs1925241 | -0.008(-0.023-0.006) | 0.252 | **0.019** |
|  | rs2569715 | 0.000(-0.015-0.014) | 0.964 | 0.955 |
|  | rs2569716 | 0.007(-0.008-0.022) | 0.369 | 0.869 |
|  | rs3745871 | -0.006(-0.020-0.009) | 0.427 | **0.019** |
|  | rs11540761 | -0.019(-0.035--0.003) | **0.018** | **0.019** |
|  | rs11574576 | -0.008(-0.023-0.007) | 0.326 | 0.805 |
|  | rs28366008 | 0.012(-0.007-0.032) | 0.224 | 0.939 |
| Right Middle | rs731170 | 0.011(-0.008-0.030) | 0.253 | 0.557 |
|  | rs1048801 | -0.003(-0.020-0.014) | 0.721 | 0.840 |
|  | rs1749316 | 0.012(-0.008-0.032) | 0.228 | 0.557 |
|  | rs1749317 | -0.003(-0.022-0.016) | 0.792 | 0.840 |
|  | rs1925241 | -0.018(-0.036--0.001) | **0.039** | 0.248 |
|  | rs2569715 | 0.005(-0.013-0.023) | 0.598 | 0.840 |
|  | rs2569716 | 0.008(-0.010-0.026) | 0.399 | 0.732 |
|  | rs3745871 | -0.014(-0.031-0.003) | 0.119 | 0.436 |
|  | rs11540761 | -0.02(-0.039--0.001) | **0.045** | 0.248 |
|  | rs11574576 | 0.002(-0.016-0.020) | 0.840 | 0.840 |
|  | rs28366008 | 0.002(-0.015-0.019) | 0.837 | 0.840 |
| Right Caudal | rs731170 | 0.016(-0.002-0.034) | 0.092 | 0.561 |
|  | rs1048801 | 0.008(-0.009-0.024) | 0.364 | 0.644 |
|  | rs1749316 | 0.018(-0.001-0.037) | 0.070 | 0.856 |
|  | rs1749317 | 0.003(-0.015-0.022) | 0.711 | 0.561 |
|  | rs1925241 | -0.024(-0.041--0.008) | **0.005** | 0.561 |
|  | rs2569715 | 0.000(-0.017-0.018) | 0.955 | 0.964 |
|  | rs2569716 | 0.004(-0.013-0.022) | 0.641 | 0.580 |
|  | rs3745871 | -0.024(-0.041--0.007) | **0.005** | 0.587 |
|  | rs11540761 | -0.027(-0.046--0.009) | **0.004** | 0.201 |
|  | rs11574576 | 0.006(-0.012-0.024) | 0.512 | 0.580 |
|  | rs28366008 | 0.002(-0.015-0.018) | 0.854 | 0.561 |

CI, confidence internal; DTI, diffusion tensor imaging; FDR, false discovery rate

**Supplementary Table 22**. Model 2: The correlation between *LILRB4* loci and DTI FA values in female.

| Items | SNP | β(95%CI) | P value | FDR-corrected. P |
| --- | --- | --- | --- | --- |
| Left Rostral | rs731170 | 0.000(-0.017-0.018) | 0.974 | 0.974 |
|  | rs1048801 | 0.005(-0.012-0.022) | 0.257 | 0.578 |
|  | rs1749316 | -0.019(-0.036--0.002) | 0.116 | 0.426 |
|  | rs1749317 | 0.004(-0.014-0.021) | 0.938 | 0.974 |
|  | rs1925241 | 0.008(-0.009-0.024) | 0.368 | 0.578 |
|  | rs2569715 | -0.004(-0.021-0.013) | 0.803 | 0.974 |
|  | rs2569716 | 0.009(-0.007-0.025) | 0.018 | 0.201 |
|  | rs3745871 | 0.018(0.001-0.036) | 0.367 | 0.578 |
|  | rs11540761 | 0.006(-0.015-0.027) | 0.324 | 0.578 |
|  | rs11574576 | 0.002(-0.016-0.020) | 0.092 | 0.426 |
|  | rs28366008 | 0.014(-0.007-0.034) | 0.555 | 0.763 |
| Left Middle | rs731170 | -0.013(-0.035-0.008) | 0.226 | 0.379 |
|  | rs1048801 | 0.004(-0.017-0.025) | 0.713 | 0.942 |
|  | rs1749316 | -0.013(-0.034-0.009) | 0.241 | 0.379 |
|  | rs1749317 | -0.001(-0.023-0.021) | 0.933 | 0.985 |
|  | rs1925241 | 0.020(-0.001-0.040) | 0.062 | 0.242 |
|  | rs2569715 | 0.000(-0.022-0.022) | 0.985 | 0.985 |
|  | rs2569716 | 0.019(-0.001-0.039) | 0.070 | 0.242 |
|  | rs3745871 | 0.026(0.005-0.048) | 0.018 | 0.201 |
|  | rs11540761 | 0.004(-0.022-0.030) | 0.771 | 0.942 |
|  | rs11574576 | 0.020(-0.003-0.042) | 0.088 | 0.242 |
|  | rs28366008 | 0.017(-0.008-0.043) | 0.189 | 0.379 |
| Left Caudal | rs731170 | 0.000(-0.022-0.021) | 0.964 | 0.964 |
|  | rs1048801 | 0.012(-0.009-0.033) | 0.563 | 0.816 |
|  | rs1749316 | -0.017(-0.037-0.004) | 0.029 | 0.204 |
|  | rs1749317 | -0.001(-0.022-0.020) | 0.668 | 0.816 |
|  | rs1925241 | 0.009(-0.011-0.030) | 0.350 | 0.770 |
|  | rs2569715 | 0.003(-0.018-0.024) | 0.652 | 0.816 |
|  | rs2569716 | 0.023(0.004-0.043) | 0.281 | 0.770 |
|  | rs3745871 | 0.010(-0.011-0.031) | 0.037 | 0.204 |
|  | rs11540761 | 0.013(-0.012-0.038) | 0.568 | 0.816 |
|  | rs11574576 | 0.019(-0.003-0.040) | 0.865 | 0.952 |
|  | rs28366008 | -0.008(-0.033-0.018) | 0.197 | 0.722 |
| Right Rostral | rs731170 | 0.019(0.001-0.038) | 0.155 | 0.531 |
|  | rs1048801 | 0.011(-0.008-0.029) | 0.680 | 0.796 |
|  | rs1749316 | -0.005(-0.024-0.013) | 0.295 | 0.579 |
|  | rs1749317 | 0.004(-0.015-0.022) | 0.369 | 0.579 |
|  | rs1925241 | -0.014(-0.032-0.003) | 0.024 | 0.134 |
|  | rs2569715 | 0.002(-0.017-0.020) | 0.552 | 0.759 |
|  | rs2569716 | 0.001(-0.016-0.019) | 0.348 | 0.579 |
|  | rs3745871 | -0.014(-0.032-0.005) | 0.012 | 0.134 |
|  | rs11540761 | 0.003(-0.019-0.025) | 0.735 | 0.796 |
|  | rs11574576 | -0.009(-0.028-0.010) | 0.193 | 0.531 |
|  | rs28366008 | -0.002(-0.024-0.020) | 0.796 | 0.796 |
| Right Middle | rs731170 | 0.020(0.003-0.036) | 0.023 | 0.084 |
|  | rs1048801 | 0.001(-0.016-0.018) | 0.900 | 0.937 |
|  | rs1749316 | 0.001(-0.016-0.018) | 0.896 | 0.937 |
|  | rs1749317 | 0.005(-0.013-0.022) | 0.610 | 0.937 |
|  | rs1925241 | -0.023(-0.038--0.007) | 0.006 | 0.068 |
|  | rs2569715 | 0.002(-0.015-0.019) | 0.818 | 0.937 |
|  | rs2569716 | -0.010(-0.026-0.006) | 0.229 | 0.504 |
|  | rs3745871 | -0.022(-0.039--0.005) | 0.012 | 0.068 |
|  | rs11540761 | -0.003(-0.024-0.017) | 0.746 | 0.937 |
|  | rs11574576 | -0.013(-0.031-0.004) | 0.140 | 0.384 |
|  | rs28366008 | 0.001(-0.020-0.021) | 0.937 | 0.937 |
| Right Caudal | rs731170 | 0.016(-0.006-0.037) | 0.038 | 0.416 |
|  | rs1048801 | -0.005(-0.026-0.017) | 0.256 | 0.703 |
|  | rs1749316 | 0.012(-0.01-0.033) | 0.569 | 0.880 |
|  | rs1749317 | 0.010(-0.012-0.032) | 0.696 | 0.880 |
|  | rs1925241 | -0.024(-0.044--0.003) | 0.112 | 0.556 |
|  | rs2569715 | 0.007(-0.015-0.028) | 0.863 | 0.880 |
|  | rs2569716 | -0.010(-0.030-0.010) | 0.880 | 0.880 |
|  | rs3745871 | -0.028(-0.049--0.007) | 0.152 | 0.556 |
|  | rs11540761 | 0.005(-0.022-0.031) | 0.781 | 0.880 |
|  | rs11574576 | -0.015(-0.037-0.007) | 0.352 | 0.773 |
|  | rs28366008 | -0.003(-0.030-0.023) | 0.864 | 0.880 |

CI, confidence internal; DTI, diffusion tensor imaging; FDR, false discovery rate
