# Supplementary material for: Uncovering the Associations of LILRB4 Genotypes With Parkinson's Disease: From Clinical Traits to Potential Pathologies
Source: CNS Neurosci Ther. 2025 Jul 23;31(7):e70522. doi: 10.1111/cns.70522 (PMC12287542; doi:10.1111/cns.70522)
Supplement: Supplementary file 4 — Table S1. [file CNS-31-e70522-s002.zip › cns70522-sup-0012-TableS23-S25@Supplementary Table 23-25 Model 1_The correlation between LILRB4 loci and CSF biomarkers.docx]

**Supplementary Table 23**. Model 1: The correlation between *LILRB4* loci and CSF biomarkers.

| Items | SNP | β(95%CI) | P | FDR-corrected. P |
| --- | --- | --- | --- | --- |
| 3-MT | rs731170 | -0.064(-0.135-0.008) | 0.083 | 0.601 |
|  | rs1048801 | 0.003(-0.068-0.074) | 0.935 | 0.935 |
|  | rs1749316 | 0.038(-0.038-0.114) | 0.328 | 0.601 |
|  | rs1749317 | -0.019(-0.089-0.052) | 0.603 | 0.663 |
|  | rs1925241 | 0.039(-0.032-0.111) | 0.282 | 0.601 |
|  | rs2569715 | 0.027(-0.043-0.098) | 0.451 | 0.640 |
|  | rs2569716 | -0.023(-0.095-0.048) | 0.524 | 0.640 |
|  | rs3745871 | 0.041(-0.029-0.112) | 0.254 | 0.601 |
|  | rs11540761 | 0.059(-0.036-0.155) | 0.226 | 0.601 |
|  | rs11574576 | -0.026(-0.101-0.049) | 0.502 | 0.640 |
|  | rs28366008 | -0.053(-0.139-0.032) | 0.222 | 0.601 |
| Aβ | rs731170 | -0.102(-0.260-0.056) | 0.209 | 0.576 |
|  | rs1048801 | 0.077(-0.036-0.190) | 0.187 | 0.576 |
|  | rs1749316 | 0.017(-0.106-0.140) | 0.790 | 0.928 |
|  | rs1749317 | -0.044(-0.160-0.072) | 0.460 | 0.723 |
|  | rs1925241 | 0.001(-0.117-0.120) | 0.985 | 0.985 |
|  | rs2569715 | -0.009(-0.126-0.108) | 0.088 | 0.576 |
|  | rs2569716 | 0.059(-0.07-0.189) | 0.370 | 0.679 |
|  | rs3745871 | 0.067(-0.054-0.188) | 0.281 | 0.618 |
|  | rs11540761 | 0.115(-0.023-0.254) | 0.107 | 0.576 |
|  | rs11574576 | -0.013(-0.128-0.102) | 0.823 | 0.928 |
|  | rs28366008 | -0.015(-0.157-0.128) | 0.844 | 0.928 |
| Aβ1-42 | rs731170 | -0.016(-0.067-0.034) | 0.529 | 0.844 |
|  | rs1048801 | -0.009(-0.057-0.040) | 0.730 | 0.844 |
|  | rs1749316 | 0.025(-0.030-0.079) | 0.372 | 0.844 |
|  | rs1749317 | 0.045(-0.007-0.097) | 0.089 | 0.831 |
|  | rs1925241 | 0.006(-0.041-0.053) | 0.802 | 0.844 |
|  | rs2569715 | -0.017(-0.066-0.033) | 0.515 | 0.844 |
|  | rs2569716 | -0.036(-0.084-0.013) | 0.151 | 0.831 |
|  | rs3745871 | -0.008(-0.057-0.040) | 0.739 | 0.844 |
|  | rs11540761 | 0.007(-0.051-0.065) | 0.807 | 0.844 |
|  | rs11574576 | 0.025(-0.027-0.076) | 0.346 | 0.844 |
|  | rs28366008 | -0.014(-0.157-0.128) | 0.844 | 0.844 |
| α-Syn | rs731170 | -0.042(-0.130-0.045) | 0.346 | 0.961 |
|  | rs1048801 | 0.029(-0.056-0.114) | 0.509 | 0.961 |
|  | rs1749316 | 0.025(-0.068-0.117) | 0.603 | 0.961 |
|  | rs1749317 | 0.013(-0.074-0.099) | 0.773 | 0.961 |
|  | rs1925241 | -0.001(-0.082-0.079) | 0.971 | 0.971 |
|  | rs2569715 | -0.040(-0.126-0.046) | 0.366 | 0.961 |
|  | rs2569716 | -0.016(-0.102-0.069) | 0.706 | 0.961 |
|  | rs3745871 | -0.012(-0.095-0.071) | 0.778 | 0.961 |
|  | rs11540761 | -0.008(-0.108-0.092) | 0.874 | 0.961 |
|  | rs11574576 | 0.084(0.001-0.166) | **0.048** | 0.531 |
|  | rs28366008 | -0.011(-0.105-0.084) | 0.826 | 0.961 |
| DOPA | rs731170 | -0.031(-0.103-0.042) | 0.411 | 0.964 |
|  | rs1048801 | 0.002(-0.070-0.074) | 0.951 | 0.992 |
|  | rs1749316 | 0.034(-0.043-0.110) | 0.392 | 0.964 |
|  | rs1749317 | 0.063(-0.008-0.133) | 0.083 | 0.458 |
|  | rs1925241 | 0.008(-0.064-0.080) | 0.825 | 0.992 |
|  | rs2569715 | 0.023(-0.048-0.094) | 0.526 | 0.964 |
|  | rs2569716 | -0.077(-0.148--0.006) | **0.036** | 0.395 |
|  | rs3745871 | 0.005(-0.066-0.077) | 0.881 | 0.992 |
|  | rs11540761 | 0.038(-0.059-0.135) | 0.447 | 0.964 |
|  | rs11574576 | -0.008(-0.083-0.068) | 0.845 | 0.992 |
|  | rs28366008 | 0.000(-0.087-0.086) | 0.992 | 0.992 |
| DOPAC | rs731170 | -0.004(-0.109-0.100) | 0.936 | 0.949 |
|  | rs1048801 | -0.017(-0.120-0.086) | 0.744 | 0.949 |
|  | rs1749316 | -0.074(-0.183-0.035) | 0.187 | 0.618 |
|  | rs1749317 | 0.012(-0.090-0.114) | 0.814 | 0.949 |
|  | rs1925241 | 0.064(-0.039-0.167) | 0.225 | 0.618 |
|  | rs2569715 | -0.003(-0.105-0.099) | 0.949 | 0.949 |
|  | rs2569716 | -0.028(-0.131-0.076) | 0.598 | 0.941 |
|  | rs3745871 | 0.065(-0.037-0.167) | 0.214 | 0.618 |
|  | rs11540761 | 0.165(0.029-0.302) | **0.019** | 0.207 |
|  | rs11574576 | -0.029(-0.137-0.079) | 0.599 | 0.941 |
|  | rs28366008 | 0.040(-0.085-0.164) | 0.533 | 0.941 |
| DA | rs731170 | -0.087(-0.268-0.095) | 0.350 | 0.687 |
|  | rs1048801 | 0.060(-0.120-0.240) | 0.514 | 0.706 |
|  | rs1749316 | 0.009(-0.183-0.201) | 0.926 | 0.957 |
|  | rs1749317 | 0.206(0.032-0.381) | **0.022** | 0.242 |
|  | rs1925241 | 0.080(-0.101-0.260) | 0.388 | 0.687 |
|  | rs2569715 | 0.150(-0.026-0.326) | 0.097 | 0.355 |
|  | rs2569716 | 0.095(-0.085-0.275) | 0.301 | 0.687 |
|  | rs3745871 | 0.071(-0.108-0.250) | 0.437 | 0.687 |
|  | rs11540761 | 0.225(-0.015-0.465) | 0.068 | 0.355 |
|  | rs11574576 | -0.005(-0.194-0.184) | 0.957 | 0.957 |
|  | rs28366008 | -0.013(-0.230-0.203) | 0.904 | 0.957 |
| GFAP | rs731170 | -0.083(-0.156--0.010) | **0.026** | 0.117 |
|  | rs1048801 | -0.001(-0.073-0.070) | 0.974 | 0.974 |
|  | rs1749316 | 0.087(0.010-0.164) | **0.027** | 0.117 |
|  | rs1749317 | 0.026(-0.047-0.098) | 0.487 | 0.974 |
|  | rs1925241 | -0.002(-0.070-0.065) | 0.947 | 0.974 |
|  | rs2569715 | -0.011(-0.083-0.062) | 0.769 | 0.974 |
|  | rs2569716 | -0.078(-0.149--0.007) | **0.032** | 0.117 |
|  | rs3745871 | -0.006(-0.075-0.064) | 0.876 | 0.974 |
|  | rs11540761 | -0.022(-0.106-0.062) | 0.610 | 0.974 |
|  | rs11574576 | 0.014(-0.055-0.084) | 0.685 | 0.974 |
|  | rs28366008 | -0.059(-0.138-0.020) | 0.146 | 0.401 |
| HVA | rs731170 | -0.017(-0.130-0.096) | 0.769 | 0.788 |
|  | rs1048801 | 0.040(-0.071-0.152) | 0.481 | 0.788 |
|  | rs1749316 | -0.068(-0.187-0.051) | 0.263 | 0.733 |
|  | rs1749317 | 0.015(-0.095-0.125) | 0.788 | 0.788 |
|  | rs1925241 | 0.055(-0.056-0.167) | 0.333 | 0.733 |
|  | rs2569715 | -0.024(-0.134-0.087) | 0.674 | 0.788 |
|  | rs2569716 | -0.063(-0.175-0.049) | 0.271 | 0.733 |
|  | rs3745871 | 0.059(-0.052-0.170) | 0.298 | 0.733 |
|  | rs11540761 | 0.154(0.005-0.302) | **0.044** | 0.487 |
|  | rs11574576 | 0.025(-0.092-0.142) | 0.677 | 0.788 |
|  | rs28366008 | -0.043(-0.177-0.092) | 0.535 | 0.788 |
| IL-1b | rs731170 | -0.025(-0.133-0.082) | 0.645 | 0.853 |
|  | rs1048801 | 0.055(-0.049-0.159) | 0.301 | 0.853 |
|  | rs1749316 | 0.016(-0.093-0.126) | 0.783 | 0.853 |
|  | rs1749317 | 0.039(-0.069-0.147) | 0.482 | 0.853 |
|  | rs1925241 | -0.010(-0.118-0.098) | 0.853 | 0.853 |
|  | rs2569715 | -0.012(-0.117-0.094) | 0.828 | 0.853 |
|  | rs2569716 | 0.037(-0.070-0.144) | 0.498 | 0.853 |
|  | rs3745871 | 0.016(-0.092-0.124) | 0.769 | 0.853 |
|  | rs11540761 | -0.086(-0.234-0.061) | 0.255 | 0.853 |
|  | rs11574576 | 0.031(-0.079-0.142) | 0.579 | 0.853 |
|  | rs28366008 | -0.025(-0.150-0.100) | 0.694 | 0.853 |
| IL-6 | rs731170 | -0.031(-0.115-0.052) | 0.464 | 0.819 |
|  | rs1048801 | -0.010(-0.091-0.072) | 0.813 | 0.947 |
|  | rs1749316 | 0.006(-0.082-0.095) | 0.887 | 0.947 |
|  | rs1749317 | -0.031(-0.114-0.051) | 0.456 | 0.819 |
|  | rs1925241 | 0.044(-0.032-0.121) | 0.258 | 0.819 |
|  | rs2569715 | -0.027(-0.109-0.055) | 0.521 | 0.819 |
|  | rs2569716 | -0.037(-0.118-0.044) | 0.374 | 0.819 |
|  | rs3745871 | 0.032(-0.047-0.111) | 0.425 | 0.819 |
|  | rs11540761 | -0.017(-0.112-0.079) | 0.734 | 0.947 |
|  | rs11574576 | 0.003(-0.077-0.082) | 0.947 | 0.947 |
|  | rs28366008 | -0.077(-0.167-0.012) | 0.091 | 0.819 |
| NFL | rs731170 | -0.005(-0.071-0.060) | 0.876 | 0.999 |
|  | rs1048801 | 0.005(-0.058-0.068) | 0.875 | 0.999 |
|  | rs1749316 | 0.046(-0.024-0.115) | 0.197 | 0.463 |
|  | rs1749317 | 0.050(-0.015-0.114) | 0.132 | 0.463 |
|  | rs1925241 | -0.054(-0.114-0.005) | 0.075 | 0.463 |
|  | rs2569715 | -0.041(-0.106-0.023) | 0.211 | 0.463 |
|  | rs2569716 | 0.001(-0.063-0.065) | 0.983 | 0.999 |
|  | rs3745871 | -0.050(-0.111-0.012) | 0.117 | 0.463 |
|  | rs11540761 | 0.000(-0.075-0.075) | 0.999 | 0.999 |
|  | rs11574576 | 0.019(-0.043-0.082) | 0.546 | 0.999 |
|  | rs28366008 | -0.010(-0.080-0.061) | 0.791 | 0.999 |
| pTau | rs731170 | -0.003(-0.042-0.037) | 0.900 | 0.900 |
|  | rs1048801 | 0.022(-0.015-0.059) | 0.246 | 0.831 |
|  | rs1749316 | -0.012(-0.053-0.029) | 0.575 | 0.831 |
|  | rs1749317 | 0.006(-0.033-0.046) | 0.756 | 0.831 |
|  | rs1925241 | 0.014(-0.022-0.050) | 0.440 | 0.831 |
|  | rs2569715 | -0.015(-0.053-0.023) | 0.432 | 0.831 |
|  | rs2569716 | 0.007(-0.030-0.045) | 0.701 | 0.831 |
|  | rs3745871 | 0.013(-0.024-0.050) | 0.500 | 0.831 |
|  | rs11540761 | 0.012(-0.033-0.057) | 0.600 | 0.831 |
|  | rs11574576 | 0.036(-0.002-0.074) | 0.067 | 0.734 |
|  | rs28366008 | -0.008(-0.051-0.035) | 0.725 | 0.831 |
| S100B | rs731170 | -0.013(-0.059-0.034) | 0.595 | 0.765 |
|  | rs1048801 | -0.003(-0.048-0.042) | 0.899 | 0.899 |
|  | rs1749316 | 0.038(-0.011-0.086) | 0.132 | 0.690 |
|  | rs1749317 | 0.024(-0.022-0.070) | 0.308 | 0.690 |
|  | rs1925241 | -0.019(-0.061-0.024) | 0.390 | 0.690 |
|  | rs2569715 | 0.024(-0.021-0.070) | 0.294 | 0.690 |
|  | rs2569716 | 0.011(-0.053-0.075) | 0.626 | 0.765 |
|  | rs3745871 | -0.022(-0.065-0.022) | 0.332 | 0.690 |
|  | rs11540761 | -0.021(-0.074-0.032) | 0.439 | 0.690 |
|  | rs11574576 | 0.056(-0.008-0.119) | **0.013** | 0.142 |
|  | rs28366008 | 0.008(-0.042-0.057) | 0.764 | 0.840 |
| sTREM2 | rs731170 | -0.020(-0.073-0.032) | 0.449 | 0.950 |
|  | rs1048801 | 0.023(-0.029-0.075) | 0.382 | 0.950 |
|  | rs1749316 | 0.003(-0.053-0.059) | 0.914 | 0.950 |
|  | rs1749317 | 0.043(-0.009-0.096) | 0.103 | 0.633 |
|  | rs1925241 | 0.018(-0.03-0.067) | 0.463 | 0.950 |
|  | rs2569715 | -0.006(-0.059-0.046) | 0.810 | 0.950 |
|  | rs2569716 | 0.001(-0.051-0.052) | 0.725 | 0.950 |
|  | rs3745871 | 0.057(0.007-0.107) | 0.882 | 0.950 |
|  | rs11540761 | 0.005(-0.056-0.065) | 0.879 | 0.950 |
|  | rs11574576 | 0.040(-0.010-0.091) | 0.115 | 0.633 |
|  | rs28366008 | 0.002(-0.055-0.059) | 0.950 | 0.950 |
| tTau | rs731170 | 0.001(-0.036-0.039) | 0.948 | 0.948 |
|  | rs1048801 | 0.010(-0.025-0.046) | 0.572 | 0.831 |
|  | rs1749316 | -0.006(-0.046-0.033) | 0.756 | 0.831 |
|  | rs1749317 | 0.007(-0.031-0.045) | 0.725 | 0.831 |
|  | rs1925241 | 0.010(-0.024-0.045) | 0.556 | 0.831 |
|  | rs2569715 | -0.013(-0.049-0.023) | 0.482 | 0.831 |
|  | rs2569716 | 0.062(0.026-0.098) | 0.736 | 0.831 |
|  | rs3745871 | 0.006(-0.029-0.042) | 0.734 | 0.831 |
|  | rs11540761 | 0.008(-0.035-0.050) | 0.725 | 0.831 |
|  | rs11574576 | 0.031(-0.005-0.068) | 0.094 | 0.831 |
|  | rs28366008 | -0.010(-0.051-0.031) | 0.632 | 0.831 |
| VMA | rs731170 | 0.000(-0.118-0.117) | 0.995 | 0.995 |
|  | rs1048801 | -0.057(-0.173-0.058) | 0.333 | 0.883 |
|  | rs1749316 | -0.052(-0.176-0.071) | 0.406 | 0.883 |
|  | rs1749317 | 0.000(-0.115-0.114) | 0.994 | 0.995 |
|  | rs1925241 | 0.022(-0.094-0.138) | 0.713 | 0.980 |
|  | rs2569715 | 0.083(-0.031-0.196) | 0.158 | 0.883 |
|  | rs2569716 | 0.051(-0.065-0.167) | 0.390 | 0.883 |
|  | rs3745871 | 0.013(-0.102-0.128) | 0.817 | 0.995 |
|  | rs11540761 | 0.049(-0.107-0.206) | 0.536 | 0.883 |
|  | rs11574576 | 0.065(-0.057-0.186) | 0.298 | 0.883 |
|  | rs28366008 | -0.041(-0.181-0.098) | 0.562 | 0.883 |
| YKL40 | rs731170 | 0.018(-0.078-0.115) | 0.713 | 0.784 |
|  | rs1048801 | -0.038(-0.132-0.056) | 0.425 | 0.779 |
|  | rs1749316 | 0.000(-0.102-0.101) | 0.995 | 0.995 |
|  | rs1749317 | -0.064(-0.159-0.031) | 0.185 | 0.523 |
|  | rs1925241 | -0.024(-0.113-0.064) | 0.589 | 0.784 |
|  | rs2569715 | -0.057(-0.152-0.037) | 0.237 | 0.523 |
|  | rs2569716 | 0.073(-0.020-0.167) | 0.127 | 0.523 |
|  | rs3745871 | 0.019(-0.072-0.110) | 0.678 | 0.784 |
|  | rs11540761 | -0.066(-0.177-0.044) | 0.238 | 0.523 |
|  | rs11574576 | -0.028(-0.119-0.064) | 0.555 | 0.784 |
|  | rs28366008 | 0.070(-0.033-0.174) | 0.183 | 0.523 |

CI, confidence internal; FDR, false discovery rate; 3-MT, 3-Methoxytyramine; Aβ, beta amyloid; Aβ1-42, beta amyloid 1-42; DA, dopamine; DOPA, dihydroxyphenylalanine; DOPAC, dihydroxyphenylacetic acid; FDR, false discovery rate; GFAP, glial fibrillary acid protein; HVA, homovanillic acid; IL-1b, Interleukin 1b; IL-6, Interleukin 6; NFL, neurofilament light; S100B, S-100 calcium binding protein B; sTREM2, soluble triggering receptor expressed on myeloid cells 2; VMA, Vanillymandelic Acid; YKL40, chitinase-3-like protein 1

**Supplementary Table 24**. Model 1: The correlation between *LILRB4* loci and CSF biomarkers in male.

| Items | SNP | β(95%CI) | P | FDR-corrected. P |
| --- | --- | --- | --- | --- |
| 3-MT | rs731170 | -0.039(-0.123-0.046) | 0.374 | 0.806 |
|  | rs1048801 | 0.009(-0.075-0.092) | 0.841 | 0.841 |
|  | rs1749316 | 0.059(-0.03-0.148) | 0.199 | 0.806 |
|  | rs1749317 | 0.014(-0.065-0.092) | 0.733 | 0.806 |
|  | rs1925241 | 0.018(-0.066-0.102) | 0.670 | 0.806 |
|  | rs2569715 | -0.020(-0.104-0.064) | 0.646 | 0.806 |
|  | rs2569716 | -0.032(-0.112-0.048) | 0.432 | 0.806 |
|  | rs3745871 | 0.017(-0.068-0.101) | 0.696 | 0.806 |
|  | rs11540761 | 0.023(-0.088-0.135) | 0.681 | 0.806 |
|  | rs11574576 | -0.023(-0.111-0.066) | 0.617 | 0.806 |
|  | rs28366008 | -0.044(-0.143-0.055) | 0.387 | 0.806 |
| Aβ | rs731170 | -0.089(-0.295-0.117) | 0.401 | 0.842 |
|  | rs1048801 | -0.017(-0.162-0.127) | 0.818 | 0.869 |
|  | rs1749316 | 0.065(-0.082-0.212) | 0.390 | 0.842 |
|  | rs1749317 | -0.066(-0.212-0.081) | 0.384 | 0.842 |
|  | rs1925241 | -0.060(-0.216-0.097) | 0.459 | 0.842 |
|  | rs2569715 | 0.089(-0.062-0.240) | 0.254 | 0.842 |
|  | rs2569716 | 0.034(-0.156-0.224) | 0.727 | 0.869 |
|  | rs3745871 | 0.014(-0.150-0.178) | 0.869 | 0.869 |
|  | rs11540761 | 0.059(-0.127-0.246) | 0.536 | 0.842 |
|  | rs11574576 | -0.035(-0.194-0.125) | 0.674 | 0.869 |
|  | rs28366008 | 0.083(-0.110-0.275) | 0.405 | 0.842 |
| Aβ1-42 | rs731170 | -0.012(-0.077-0.052) | 0.706 | 0.811 |
|  | rs1048801 | -0.021(-0.082-0.040) | 0.494 | 0.776 |
|  | rs1749316 | 0.012(-0.058-0.082) | 0.737 | 0.811 |
|  | rs1749317 | 0.045(-0.022-0.111) | 0.189 | 0.693 |
|  | rs1925241 | 0.023(-0.037-0.083) | 0.451 | 0.776 |
|  | rs2569715 | -0.037(-0.101-0.028) | 0.269 | 0.722 |
|  | rs2569716 | -0.058(-0.120-0.003) | 0.064 | 0.519 |
|  | rs3745871 | -0.002(-0.063-0.059) | 0.946 | 0.946 |
|  | rs11540761 | 0.016(-0.056-0.088) | 0.655 | 0.811 |
|  | rs11574576 | 0.055(-0.009-0.120) | 0.094 | 0.519 |
|  | rs28366008 | -0.035(-0.106-0.035) | 0.328 | 0.722 |
| α-Syn | rs731170 | -0.040(-0.155-0.076) | 0.502 | 0.926 |
|  | rs1048801 | -0.050(-0.164-0.064) | 0.392 | 0.926 |
|  | rs1749316 | 0.010(-0.114-0.134) | 0.875 | 0.926 |
|  | rs1749317 | 0.005(-0.108-0.118) | 0.926 | 0.926 |
|  | rs1925241 | 0.016(-0.092-0.123) | 0.777 | 0.926 |
|  | rs2569715 | -0.006(-0.122-0.110) | 0.914 | 0.926 |
|  | rs2569716 | 0.020(-0.090-0.131) | 0.717 | 0.926 |
|  | rs3745871 | -0.007(-0.119-0.105) | 0.900 | 0.926 |
|  | rs11540761 | 0.010(-0.117-0.137) | 0.877 | 0.926 |
|  | rs11574576 | 0.073(-0.037-0.184) | 0.193 | 0.926 |
|  | rs28366008 | -0.034(-0.161-0.092) | 0.595 | 0.926 |
| DOPA | rs731170 | -0.030(-0.123-0.062) | 0.522 | 0.860 |
|  | rs1048801 | 0.011(-0.080-0.102) | 0.811 | 0.860 |
|  | rs1749316 | 0.030(-0.068-0.129) | 0.544 | 0.860 |
|  | rs1749317 | 0.077(-0.008-0.161) | 0.079 | 0.622 |
|  | rs1925241 | 0.010(-0.081-0.102) | 0.827 | 0.860 |
|  | rs2569715 | 0.014(-0.077-0.106) | 0.759 | 0.860 |
|  | rs2569716 | -0.071(-0.157-0.016) | 0.113 | 0.622 |
|  | rs3745871 | 0.015(-0.077-0.107) | 0.747 | 0.860 |
|  | rs11540761 | 0.039(-0.082-0.160) | 0.529 | 0.860 |
|  | rs11574576 | -0.009(-0.105-0.087) | 0.858 | 0.860 |
|  | rs28366008 | 0.010(-0.099-0.118) | 0.860 | 0.860 |
| DOPAC | rs731170 | -0.013(-0.141-0.116) | 0.847 | 0.901 |
|  | rs1048801 | 0.008(-0.118-0.133) | 0.901 | 0.901 |
|  | rs1749316 | -0.119(-0.252-0.015) | 0.085 | 0.312 |
|  | rs1749317 | 0.011(-0.108-0.130) | 0.856 | 0.901 |
|  | rs1925241 | 0.098(-0.027-0.223) | 0.127 | 0.349 |
|  | rs2569715 | 0.026(-0.101-0.152) | 0.694 | 0.901 |
|  | rs2569716 | 0.014(-0.107-0.135) | 0.824 | 0.901 |
|  | rs3745871 | 0.115(-0.010-0.241) | 0.075 | 0.312 |
|  | rs11540761 | 0.224(0.062-0.386) | **0.008** | 0.087 |
|  | rs11574576 | -0.063(-0.196-0.069) | 0.350 | 0.770 |
|  | rs28366008 | 0.033(-0.116-0.183) | 0.663 | 0.901 |
| DA | rs731170 | -0.160(-0.561-0.241) | 0.435 | 0.798 |
|  | rs1048801 | -0.019(-0.413-0.374) | 0.923 | 0.966 |
|  | rs1749316 | 0.102(-0.323-0.527) | 0.639 | 0.966 |
|  | rs1749317 | 0.419(0.057-0.781) | **0.026** | 0.282 |
|  | rs1925241 | 0.062(-0.334-0.459) | 0.758 | 0.966 |
|  | rs2569715 | 0.321(-0.072-0.714) | 0.112 | 0.466 |
|  | rs2569716 | 0.189(-0.189-0.566) | 0.330 | 0.798 |
|  | rs3745871 | 0.033(-0.366-0.433) | 0.870 | 0.966 |
|  | rs11540761 | 0.408(-0.112-0.928) | 0.127 | 0.466 |
|  | rs11574576 | -0.172(-0.587-0.244) | 0.420 | 0.798 |
|  | rs28366008 | 0.010(-0.459-0.480) | 0.966 | 0.966 |
| GFAP | rs731170 | -0.106(-0.200--0.013) | **0.027** | 0.150 |
|  | rs1048801 | -0.071(-0.164-0.022) | 0.130 | 0.286 |
|  | rs1749316 | 0.136(0.037-0.236) | **0.008** | 0.088 |
|  | rs1749317 | 0.020(-0.073-0.112) | 0.678 | 0.746 |
|  | rs1925241 | -0.008(-0.096-0.080) | 0.864 | 0.864 |
|  | rs2569715 | 0.054(-0.041-0.149) | 0.265 | 0.486 |
|  | rs2569716 | -0.072(-0.162-0.018) | 0.119 | 0.286 |
|  | rs3745871 | -0.002(-0.094-0.089) | 0.594 | 0.746 |
|  | rs11540761 | -0.035(-0.138-0.069) | 0.515 | 0.746 |
|  | rs11574576 | -0.021(-0.112-0.069) | 0.642 | 0.746 |
|  | rs28366008 | -0.085(-0.187-0.018) | 0.108 | 0.286 |
| HVA | rs731170 | 0.034(-0.108-0.177) | 0.638 | 0.936 |
|  | rs1048801 | 0.023(-0.117-0.163) | 0.746 | 0.936 |
|  | rs1749316 | -0.142(-0.291-0.006) | 0.064 | 0.350 |
|  | rs1749317 | -0.004(-0.136-0.128) | 0.948 | 0.967 |
|  | rs1925241 | 0.055(-0.086-0.196) | 0.445 | 0.936 |
|  | rs2569715 | 0.031(-0.111-0.172) | 0.670 | 0.936 |
|  | rs2569716 | -0.003(-0.138-0.132) | 0.967 | 0.967 |
|  | rs3745871 | 0.077(-0.064-0.218) | 0.289 | 0.936 |
|  | rs11540761 | 0.187(0.004-0.370) | **0.048** | 0.350 |
|  | rs11574576 | 0.028(-0.120-0.175) | 0.716 | 0.936 |
|  | rs28366008 | -0.025(-0.192-0.141) | 0.766 | 0.936 |
| IL-1b | rs731170 | 0.037(-0.118-0.191) | 0.645 | 0.996 |
|  | rs1048801 | 0.061(-0.088-0.209) | 0.427 | 0.939 |
|  | rs1749316 | 0.000(-0.164-0.165) | 0.996 | 0.996 |
|  | rs1749317 | 0.062(-0.086-0.209) | 0.415 | 0.939 |
|  | rs1925241 | -0.025(-0.179-0.130) | 0.753 | 0.996 |
|  | rs2569715 | -0.023(-0.173-0.127) | 0.765 | 0.996 |
|  | rs2569716 | 0.065(-0.078-0.208) | 0.380 | 0.939 |
|  | rs3745871 | -0.006(-0.165-0.152) | 0.937 | 0.996 |
|  | rs11540761 | -0.133(-0.346-0.081) | 0.229 | 0.939 |
|  | rs11574576 | 0.067(0.052-0.083) | 0.400 | 0.939 |
|  | rs28366008 | -0.017(-0.194-0.159) | 0.847 | 0.996 |
| IL-6 | rs731170 | -0.052(-0.150-0.046) | 0.301 | 0.961 |
|  | rs1048801 | 0.006(-0.090-0.103) | 0.898 | 0.961 |
|  | rs1749316 | 0.067(-0.038-0.172) | 0.210 | 0.961 |
|  | rs1749317 | -0.024(-0.120-0.071) | 0.619 | 0.961 |
|  | rs1925241 | -0.003(-0.094-0.089) | 0.957 | 0.961 |
|  | rs2569715 | -0.045(-0.143-0.053) | 0.370 | 0.961 |
|  | rs2569716 | -0.015(-0.109-0.079) | 0.751 | 0.961 |
|  | rs3745871 | -0.005(-0.100-0.090) | 0.916 | 0.961 |
|  | rs11540761 | -0.003(-0.110-0.105) | 0.961 | 0.961 |
|  | rs11574576 | 0.014(-0.080-0.108) | 0.776 | 0.961 |
|  | rs28366008 | -0.094(-0.201-0.012) | 0.084 | 0.920 |
| NFL | rs731170 | -0.008(-0.084-0.068) | 0.839 | 0.940 |
|  | rs1048801 | -0.007(-0.082-0.068) | 0.864 | 0.940 |
|  | rs1749316 | 0.055(-0.027-0.136) | 0.189 | 0.839 |
|  | rs1749317 | 0.016(-0.058-0.090) | 0.672 | 0.940 |
|  | rs1925241 | -0.041(-0.112-0.029) | 0.253 | 0.839 |
|  | rs2569715 | -0.061(-0.137-0.015) | 0.118 | 0.839 |
|  | rs2569716 | -0.003(-0.076-0.07) | 0.940 | 0.940 |
|  | rs3745871 | -0.039(-0.112-0.035) | 0.305 | 0.839 |
|  | rs11540761 | -0.007(-0.09-0.077) | 0.876 | 0.940 |
|  | rs11574576 | 0.019(-0.054-0.091) | 0.614 | 0.940 |
|  | rs28366008 | -0.015(-0.098-0.068) | 0.720 | 0.940 |
| pTau | rs731170 | 0.006(-0.043-0.055) | 0.820 | 0.820 |
|  | rs1048801 | -0.022(-0.068-0.024) | 0.359 | 0.494 |
|  | rs1749316 | -0.029(-0.081-0.023) | 0.278 | 0.494 |
|  | rs1749317 | -0.030(-0.080-0.020) | 0.239 | 0.494 |
|  | rs1925241 | 0.028(-0.018-0.073) | 0.233 | 0.494 |
|  | rs2569715 | 0.025(-0.024-0.073) | 0.321 | 0.494 |
|  | rs2569716 | 0.023(-0.024-0.070) | 0.338 | 0.494 |
|  | rs3745871 | 0.012(-0.035-0.058) | 0.626 | 0.689 |
|  | rs11540761 | 0.027(-0.028-0.082) | 0.336 | 0.494 |
|  | rs11574576 | 0.044(-0.005-0.093) | 0.078 | 0.494 |
|  | rs28366008 | -0.018(-0.073-0.036) | 0.514 | 0.628 |
| S100B | rs731170 | -0.029(-0.087-0.029) | 0.322 | 0.501 |
|  | rs1048801 | -0.048(-0.104-0.009) | 0.102 | 0.251 |
|  | rs1749316 | 0.054(-0.008-0.116) | 0.089 | 0.251 |
|  | rs1749317 | -0.006(-0.052-0.04) | 0.836 | 0.836 |
|  | rs1925241 | -0.024(-0.078-0.029) | 0.375 | 0.501 |
|  | rs2569715 | 0.081(0.024-0.139) | **0.006** | 0.063 |
|  | rs2569716 | 0.045(-0.011-0.100) | 0.114 | 0.251 |
|  | rs3745871 | -0.030(-0.086-0.026) | 0.302 | 0.501 |
|  | rs11540761 | -0.027(-0.09-0.037) | 0.410 | 0.501 |
|  | rs11574576 | 0.048(-0.007-0.103) | 0.091 | 0.251 |
|  | rs28366008 | -0.012(-0.076-0.051) | 0.703 | 0.773 |
| sTREM2 | rs731170 | -0.042(-0.105-0.022) | 0.201 | 0.851 |
|  | rs1048801 | -0.013(-0.076-0.049) | 0.676 | 0.998 |
|  | rs1749316 | 0.030(-0.038-0.099) | 0.387 | 0.851 |
|  | rs1749317 | 0.021(-0.036-0.077) | 0.515 | 0.944 |
|  | rs1925241 | 0.029(-0.031-0.088) | 0.346 | 0.851 |
|  | rs2569715 | 0.005(-0.059-0.069) | 0.889 | 0.998 |
|  | rs2569716 | 0.032(-0.029-0.093) | 0.305 | 0.851 |
|  | rs3745871 | -0.008(-0.07-0.054) | 0.793 | 0.998 |
|  | rs11540761 | 0.002(-0.068-0.072) | 0.948 | 0.998 |
|  | rs11574576 | 0.038(-0.023-0.099) | 0.225 | 0.851 |
|  | rs28366008 | 0.000(-0.070-0.070) | 0.998 | 0.998 |
| tTau | rs731170 | 0.011(-0.037-0.059) | 0.654 | 0.719 |
|  | rs1048801 | -0.031(-0.075-0.014) | 0.182 | 0.630 |
|  | rs1749316 | -0.024(-0.074-0.027) | 0.361 | 0.630 |
|  | rs1749317 | -0.033(-0.081-0.016) | 0.189 | 0.630 |
|  | rs1925241 | 0.021(-0.023-0.066) | 0.347 | 0.630 |
|  | rs2569715 | 0.021(-0.027-0.068) | 0.398 | 0.630 |
|  | rs2569716 | 0.017(-0.029-0.063) | 0.480 | 0.660 |
|  | rs3745871 | 0.006(-0.039-0.051) | 0.791 | 0.791 |
|  | rs11540761 | 0.016(-0.038-0.069) | 0.570 | 0.697 |
|  | rs11574576 | 0.040(-0.008-0.088) | 0.100 | 0.630 |
|  | rs28366008 | -0.023(-0.076-0.030) | 0.401 | 0.630 |
| VMA | rs731170 | -0.104(-0.441-0.233) | 0.547 | 0.638 |
|  | rs1048801 | -0.316(-0.640-0.008) | 0.059 | 0.635 |
|  | rs1749316 | -0.149(-0.505-0.207) | 0.414 | 0.635 |
|  | rs1749317 | -0.088(-0.400-0.223) | 0.580 | 0.638 |
|  | rs1925241 | 0.171(-0.161-0.502) | 0.316 | 0.635 |
|  | rs2569715 | 0.244(-0.087-0.574) | 0.152 | 0.635 |
|  | rs2569716 | 0.045(-0.273-0.363) | 0.783 | 0.783 |
|  | rs3745871 | 0.178(-0.156-0.512) | 0.298 | 0.635 |
|  | rs11540761 | 0.109(-0.332-0.549) | 0.355 | 0.635 |
|  | rs11574576 | 0.192(-0.156-0.540) | 0.281 | 0.635 |
|  | rs28366008 | -0.148(-0.542-0.245) | 0.462 | 0.635 |
| YKL40 | rs731170 | -0.026(-0.095-0.043) | 0.460 | 0.807 |
|  | rs1048801 | -0.030(-0.098-0.037) | 0.382 | 0.807 |
|  | rs1749316 | 0.045(-0.028-0.119) | 0.229 | 0.807 |
|  | rs1749317 | -0.012(-0.079-0.056) | 0.736 | 0.847 |
|  | rs1925241 | -0.018(-0.082-0.046) | 0.587 | 0.807 |
|  | rs2569715 | -0.010(-0.079-0.059) | 0.785 | 0.847 |
|  | rs2569716 | 0.040(-0.026-0.106) | 0.235 | 0.807 |
|  | rs3745871 | -0.029(-0.095-0.038) | 0.397 | 0.807 |
|  | rs11540761 | -0.007(-0.083-0.068) | 0.847 | 0.847 |
|  | rs11574576 | 0.019(-0.047-0.085) | 0.577 | 0.807 |
|  | rs28366008 | 0.026(-0.049-0.101) | 0.493 | 0.807 |

CI, confidence internal; FDR, false discovery rate; 3-MT, 3-Methoxytyramine; Aβ, beta amyloid; Aβ1-42, beta amyloid 1-42; DA, dopamine; DOPA, dihydroxyphenylalanine; DOPAC, dihydroxyphenylacetic acid; FDR, false discovery rate; GFAP, glial fibrillary acid protein; HVA, homovanillic acid; IL-1b, Interleukin 1b; IL-6, Interleukin 6; NFL, neurofilament light; S100B, S-100 calcium binding protein B; sTREM2, soluble triggering receptor expressed on myeloid cells 2; VMA, Vanillymandelic Acid; YKL40, chitinase-3-like protein 1

**Supplementary Table 25**. Model 1: The correlation between *LILRB4* loci and CSF biomarkers in female.

| Items | SNP | β(95%CI) | P | FDR-corrected. P |
| --- | --- | --- | --- | --- |
| 3-MT | rs731170 | -0.121(-0.253-0.011) | 0.080 | 0.274 |
|  | rs1048801 | -0.008(-0.147-0.131) | 0.910 | 0.944 |
|  | rs1749316 | -0.005(-0.149-0.138) | 0.944 | 0.944 |
|  | rs1749317 | -0.142(-0.294-0.010) | 0.073 | 0.274 |
|  | rs1925241 | 0.085(-0.051-0.220) | 0.226 | 0.414 |
|  | rs2569715 | 0.124(-0.001-0.249) | 0.059 | 0.274 |
|  | rs2569716 | 0.016(-0.142-0.173) | 0.847 | 0.944 |
|  | rs3745871 | 0.094(-0.035-0.223) | 0.160 | 0.352 |
|  | rs11540761 | 0.159(-0.026-0.345) | 0.100 | 0.274 |
|  | rs11574576 | -0.044(-0.189-0.100) | 0.550 | 0.756 |
|  | rs28366008 | -0.078(-0.247-0.092) | 0.374 | 0.587 |
| Aβ | rs731170 | -0.136(-0.382-0.110) | 0.286 | 0.449 |
|  | rs1048801 | 0.250(0.082-0.417) | **0.007** | 0.072 |
|  | rs1749316 | -0.026(-0.260-0.207) | 0.828 | 0.925 |
|  | rs1749317 | -0.009(-0.198-0.180) | 0.925 | 0.925 |
|  | rs1925241 | 0.058(-0.124-0.240) | 0.536 | 0.737 |
|  | rs2569715 | -0.127(-0.302-0.048) | 0.166 | 0.449 |
|  | rs2569716 | 0.105(-0.075-0.285) | 0.262 | 0.449 |
|  | rs3745871 | 0.113(-0.067-0.292) | 0.229 | 0.449 |
|  | rs11540761 | 0.153(-0.063-0.369) | 0.175 | 0.449 |
|  | rs11574576 | 0.013(-0.155-0.180) | 0.882 | 0.925 |
|  | rs28366008 | -0.133(-0.341-0.075) | 0.219 | 0.449 |
| Aβ1-42 | rs731170 | -0.023(-0.106-0.059) | 0.580 | 0.944 |
|  | rs1048801 | 0.011(-0.069-0.092) | 0.724 | 0.944 |
|  | rs1749316 | 0.044(-0.044-0.131) | 0.328 | 0.944 |
|  | rs1749317 | 0.054(-0.029-0.138) | 0.203 | 0.944 |
|  | rs1925241 | -0.022(-0.099-0.056) | 0.583 | 0.944 |
|  | rs2569715 | 0.010(-0.068-0.088) | 0.806 | 0.944 |
|  | rs2569716 | 0.000(-0.079-0.079) | 0.994 | 0.994 |
|  | rs3745871 | -0.018(-0.098-0.062) | 0.658 | 0.944 |
|  | rs11540761 | -0.009(-0.108-0.090) | 0.858 | 0.944 |
|  | rs11574576 | -0.023(-0.107-0.062) | 0.596 | 0.944 |
|  | rs28366008 | -0.010(-0.101-0.080) | 0.822 | 0.944 |
| α-Synuclein | rs731170 | -0.047(-0.173-0.079) | 0.468 | 0.693 |
|  | rs1048801 | 0.177(0.062-0.292) | **0.003** | **0.036** |
|  | rs1749316 | 0.051(-0.077-0.180) | 0.435 | 0.693 |
|  | rs1749317 | 0.032(-0.099-0.164) | 0.630 | 0.693 |
|  | rs1925241 | -0.033(-0.146-0.079) | 0.561 | 0.693 |
|  | rs2569715 | -0.103(-0.222-0.016) | 0.093 | 0.330 |
|  | rs2569716 | -0.101(-0.228-0.025) | 0.120 | 0.330 |
|  | rs3745871 | -0.021(-0.135-0.093) | 0.721 | 0.721 |
|  | rs11540761 | -0.058(-0.218-0.102) | 0.478 | 0.693 |
|  | rs11574576 | 0.107(-0.011-0.225) | 0.078 | 0.330 |
|  | rs28366008 | 0.034(-0.098-0.165) | 0.618 | 0.693 |
| DOPA | rs731170 | -0.036(-0.145-0.073) | 0.525 | 0.982 |
|  | rs1048801 | -0.015(-0.125-0.096) | 0.799 | 0.982 |
|  | rs1749316 | 0.043(-0.071-0.157) | 0.459 | 0.982 |
|  | rs1749317 | -0.008(-0.134-0.118) | 0.899 | 0.982 |
|  | rs1925241 | -0.001(-0.111-0.109) | 0.982 | 0.982 |
|  | rs2569715 | 0.044(-0.060-0.147) | 0.412 | 0.982 |
|  | rs2569716 | -0.079(-0.203-0.045) | 0.219 | 0.982 |
|  | rs3745871 | -0.010(-0.115-0.096) | 0.858 | 0.982 |
|  | rs11540761 | 0.053(-0.099-0.205) | 0.499 | 0.982 |
|  | rs11574576 | -0.026(-0.142-0.090) | 0.662 | 0.982 |
|  | rs28366008 | -0.026(-0.163-0.110) | 0.705 | 0.982 |
| DOPAC | rs731170 | 0.009(-0.170-0.188) | 0.923 | 0.923 |
|  | rs1048801 | -0.072(-0.252-0.108) | 0.437 | 0.923 |
|  | rs1749316 | 0.025(-0.162-0.213) | 0.791 | 0.923 |
|  | rs1749317 | -0.010(-0.216-0.195) | 0.922 | 0.923 |
|  | rs1925241 | -0.019(-0.198-0.161) | 0.840 | 0.923 |
|  | rs2569715 | -0.058(-0.228-0.111) | 0.502 | 0.923 |
|  | rs2569716 | -0.142(-0.163--0.122) | 0.173 | 0.923 |
|  | rs3745871 | -0.033(-0.205-0.139) | 0.708 | 0.923 |
|  | rs11540761 | 0.040(-0.210-0.290) | 0.755 | 0.923 |
|  | rs11574576 | 0.026(-0.163-0.215) | 0.790 | 0.923 |
|  | rs28366008 | 0.054(-0.168-0.277) | 0.635 | 0.923 |
| DA | rs731170 | -0.107(-0.274-0.06) | 0.215 | 0.655 |
|  | rs1048801 | 0.048(-0.124-0.220) | 0.587 | 0.922 |
|  | rs1749316 | -0.012(-0.19-0.166) | 0.898 | 0.954 |
|  | rs1749317 | 0.085(-0.109-0.278) | 0.397 | 0.728 |
|  | rs1925241 | 0.134(-0.032-0.301) | 0.121 | 0.655 |
|  | rs2569715 | 0.011(-0.151-0.173) | 0.893 | 0.954 |
|  | rs2569716 | -0.095(-0.267-0.077) | 0.342 | 0.728 |
|  | rs3745871 | 0.114(-0.047-0.274) | 0.171 | 0.655 |
|  | rs11540761 | 0.143(-0.091-0.377) | 0.238 | 0.655 |
|  | rs11574576 | -0.005(-0.186-0.175) | 0.954 | 0.954 |
|  | rs28366008 | 0.026(-0.186-0.238) | 0.809 | 0.954 |
| GFAP | rs731170 | -0.034(-0.149-0.081) | 0.566 | 0.981 |
|  | rs1048801 | 0.131(0.024-0.237) | **0.018** | 0.097 |
|  | rs1749316 | -0.001(-0.119-0.116) | 0.981 | 0.981 |
|  | rs1749317 | 0.053(-0.066-0.172) | 0.384 | 0.844 |
|  | rs1925241 | 0.007(-0.095-0.109) | 0.894 | 0.981 |
|  | rs2569715 | -0.137(-0.243--0.03) | **0.013** | 0.097 |
|  | rs2569716 | -0.098(-0.213-0.016) | 0.096 | 0.264 |
|  | rs3745871 | 0.025(-0.079-0.129) | 0.640 | 0.981 |
|  | rs11540761 | 0.008(-0.138-0.153) | 0.917 | 0.981 |
|  | rs11574576 | 0.094(-0.013-0.201) | 0.089 | 0.264 |
|  | rs28366008 | -0.009(-0.129-0.111) | 0.880 | 0.981 |
| HVA | rs731170 | -0.133(-0.311-0.045) | 0.151 | 0.554 |
|  | rs1048801 | 0.083(-0.100-0.266) | 0.378 | 0.697 |
|  | rs1749316 | 0.093(-0.096-0.282) | 0.339 | 0.697 |
|  | rs1749317 | 0.065(-0.144-0.273) | 0.546 | 0.697 |
|  | rs1925241 | 0.053(-0.129-0.236) | 0.570 | 0.697 |
|  | rs2569715 | -0.132(-0.301-0.037) | 0.133 | 0.554 |
|  | rs2569716 | -0.247(-0.443--0.050) | **0.018** | 0.196 |
|  | rs3745871 | 0.026(-0.149-0.201) | 0.770 | 0.847 |
|  | rs11540761 | 0.085(-0.169-0.339) | 0.514 | 0.697 |
|  | rs11574576 | 0.005(-0.188-0.199) | 0.956 | 0.956 |
|  | rs28366008 | -0.086(-0.312-0.140) | 0.459 | 0.697 |
| IL-1b | rs731170 | -0.139(-0.255--0.022) | **0.026** | 0.286 |
|  | rs1048801 | 0.043(-0.080-0.166) | 0.476 | 0.927 |
|  | rs1749316 | 0.047(-0.090-0.184) | 0.504 | 0.927 |
|  | rs1749317 | -0.018(-0.161-0.124) | 0.803 | 0.927 |
|  | rs1925241 | 0.017(-0.110-0.144) | 0.794 | 0.927 |
|  | rs2569715 | 0.011(-0.117-0.139) | 0.870 | 0.927 |
|  | rs2569716 | -0.040(-0.184-0.105) | 0.595 | 0.927 |
|  | rs3745871 | 0.053(-0.067-0.173) | 0.393 | 0.927 |
|  | rs11540761 | -0.008(-0.177-0.161) | 0.927 | 0.927 |
|  | rs11574576 | -0.045(-0.182-0.093) | 0.530 | 0.927 |
|  | rs28366008 | -0.041(-0.193-0.110) | 0.597 | 0.927 |
| IL-6 | rs731170 | 0.005(-0.152-0.162) | **0.014** | 0.150 |
|  | rs1048801 | -0.038(-0.186-0.111) | 0.621 | 0.748 |
|  | rs1749316 | -0.108(-0.266-0.051) | 0.187 | 0.513 |
|  | rs1749317 | -0.068(-0.231-0.094) | 0.411 | 0.748 |
|  | rs1925241 | 0.133(-0.004-0.270) | 0.061 | 0.334 |
|  | rs2569715 | 0.016(-0.133-0.166) | 0.831 | 0.831 |
|  | rs2569716 | -0.078(-0.236-0.080) | 0.336 | 0.738 |
|  | rs3745871 | 0.105(-0.035-0.245) | 0.146 | 0.513 |
|  | rs11540761 | -0.048(-0.246-0.151) | 0.639 | 0.748 |
|  | rs11574576 | -0.031(-0.180-0.117) | 0.680 | 0.748 |
|  | rs28366008 | -0.048(-0.211-0.115) | 0.566 | 0.748 |
| NFL | rs731170 | -0.007(-0.132-0.118) | 0.915 | 0.976 |
|  | rs1048801 | 0.031(-0.088-0.149) | 0.612 | 0.976 |
|  | rs1749316 | 0.026(-0.101-0.153) | 0.689 | 0.976 |
|  | rs1749317 | 0.106(-0.022-0.235) | 0.107 | 0.946 |
|  | rs1925241 | -0.077(-0.188-0.033) | 0.172 | 0.946 |
|  | rs2569715 | 0.007(-0.112-0.126) | 0.910 | 0.976 |
|  | rs2569716 | 0.020(-0.107-0.146) | 0.761 | 0.976 |
|  | rs3745871 | -0.061(-0.174-0.051) | 0.287 | 0.976 |
|  | rs11540761 | 0.026(-0.132-0.184) | 0.746 | 0.976 |
|  | rs11574576 | 0.005(-0.113-0.123) | 0.935 | 0.976 |
|  | rs28366008 | -0.002(-0.132-0.128) | 0.976 | 0.976 |
| pTau | rs731170 | -0.016(-0.081-0.049) | 0.633 | 0.801 |
|  | rs1048801 | 0.097(0.036-0.159) | **0.002** | **0.022** |
|  | rs1749316 | 0.015(-0.052-0.082) | 0.653 | 0.801 |
|  | rs1749317 | 0.062(-0.002-0.126) | 0.060 | 0.221 |
|  | rs1925241 | -0.008(-0.068-0.051) | 0.788 | 0.801 |
|  | rs2569715 | -0.072(-0.131--0.013) | **0.018** | 0.101 |
|  | rs2569716 | -0.018(-0.079-0.043) | 0.568 | 0.801 |
|  | rs3745871 | 0.015(-0.047-0.076) | 0.643 | 0.801 |
|  | rs11540761 | -0.016(-0.091-0.060) | 0.684 | 0.801 |
|  | rs11574576 | 0.022(-0.041-0.084) | 0.498 | 0.801 |
|  | rs28366008 | 0.009(-0.062-0.080) | 0.801 | 0.801 |
| S100B | rs731170 | 0.025(-0.051-0.102) | 0.519 | 0.816 |
|  | rs1048801 | 0.080(0.008-0.151) | **0.031** | 0.085 |
|  | rs1749316 | 0.009(-0.069-0.088) | 0.817 | 0.841 |
|  | rs1749317 | 0.107(0.030-0.184) | **0.008** | 0.085 |
|  | rs1925241 | -0.009(-0.077-0.060) | 0.802 | 0.841 |
|  | rs2569715 | -0.087(-0.159--0.016) | **0.018** | 0.085 |
|  | rs2569716 | -0.071(-0.147-0.006) | 0.073 | 0.161 |
|  | rs3745871 | -0.001(-0.071-0.068) | 0.730 | 0.841 |
|  | rs11540761 | -0.010(-0.107-0.087) | 0.841 | 0.841 |
|  | rs11574576 | 0.081(0.010-0.152) | **0.027** | 0.085 |
|  | rs28366008 | 0.046(-0.033-0.126) | 0.257 | 0.471 |
| sTREM2 | rs731170 | 0.019(-0.076-0.115) | 0.693 | 0.995 |
|  | rs1048801 | 0.094(0.005-0.182) | **0.041** | 0.453 |
|  | rs1749316 | -0.048(-0.145-0.049) | 0.332 | 0.994 |
|  | rs1749317 | 0.087(-0.011-0.185) | 0.084 | 0.461 |
|  | rs1925241 | 0.000(-0.085-0.085) | 0.995 | 0.995 |
|  | rs2569715 | -0.021(-0.112-0.070) | 0.658 | 0.995 |
|  | rs2569716 | -0.037(-0.133-0.059) | 0.452 | 0.994 |
|  | rs3745871 | 0.008(-0.078-0.094) | 0.856 | 0.995 |
|  | rs11540761 | 0.015(-0.105-0.136) | 0.805 | 0.995 |
|  | rs11574576 | 0.039(-0.050-0.129) | 0.392 | 0.994 |
|  | rs28366008 | 0.004(-0.095-0.103) | 0.938 | 0.995 |
| tTau | rs731170 | -0.015(-0.075-0.046) | 0.635 | 0.851 |
|  | rs1048801 | 0.081(0.024-0.139) | **0.006** | 0.064 |
|  | rs1749316 | 0.021(-0.041-0.084) | 0.502 | 0.851 |
|  | rs1749317 | 0.068(0.009-0.128) | **0.026** | 0.120 |
|  | rs1925241 | -0.008(-0.064-0.048) | 0.782 | 0.851 |
|  | rs2569715 | -0.061(-0.116--0.005) | **0.033** | 0.120 |
|  | rs2569716 | -0.011(-0.068-0.047) | 0.719 | 0.851 |
|  | rs3745871 | 0.006(-0.051-0.063) | 0.835 | 0.851 |
|  | rs11540761 | -0.007(-0.078-0.064) | 0.851 | 0.851 |
|  | rs11574576 | 0.017(-0.041-0.075) | 0.569 | 0.851 |
|  | rs28366008 | 0.010(-0.056-0.077) | 0.759 | 0.851 |
| VMA | rs731170 | 0.125(-0.027-0.277) | 0.115 | 0.449 |
|  | rs1048801 | 0.124(-0.030-0.278) | 0.122 | 0.449 |
|  | rs1749316 | 0.002(-0.161-0.166) | 0.977 | 0.981 |
|  | rs1749317 | 0.12(-0.056-0.296) | 0.189 | 0.514 |
|  | rs1925241 | -0.087(-0.242-0.069) | 0.280 | 0.514 |
|  | rs2569715 | -0.034(-0.183-0.115) | 0.656 | 0.884 |
|  | rs2569716 | 0.002(-0.178-0.182) | 0.981 | 0.981 |
|  | rs3745871 | -0.122(-0.268-0.024) | 0.108 | 0.449 |
|  | rs11540761 | -0.137(-0.352-0.079) | 0.255 | 0.514 |
|  | rs11574576 | 0.030(-0.136-0.196) | 0.723 | 0.884 |
|  | rs28366008 | -0.076(-0.270-0.118) | 0.445 | 0.699 |
| YKL40 | rs731170 | 0.098(-0.156-0.353) | 0.451 | 0.621 |
|  | rs1048801 | -0.005(-0.247-0.237) | 0.695 | 0.764 |
|  | rs1749316 | -0.088(-0.114--0.062) | 0.508 | 0.621 |
|  | rs1749317 | -0.224(-0.485-0.038) | 0.097 | 0.506 |
|  | rs1925241 | -0.034(-0.261-0.193) | 0.768 | 0.768 |
|  | rs2569715 | -0.128(-0.370-0.114) | 0.303 | 0.506 |
|  | rs2569716 | 0.164(-0.091-0.420) | 0.211 | 0.506 |
|  | rs3745871 | 0.116(-0.113-0.346) | 0.322 | 0.506 |
|  | rs11540761 | -0.215(-0.535-0.105) | 0.191 | 0.506 |
|  | rs11574576 | -0.143(-0.382-0.097) | 0.247 | 0.506 |
|  | rs28366008 | 0.148(-0.116-0.413) | 0.274 | 0.506 |

CI, confidence internal; FDR, false discovery rate; 3-MT, 3-Methoxytyramine; Aβ, beta amyloid; Aβ1-42, beta amyloid 1-42; DA, dopamine; DOPA, dihydroxyphenylalanine; DOPAC, dihydroxyphenylacetic acid; FDR, false discovery rate; GFAP, glial fibrillary acid protein; HVA, homovanillic acid; IL-1b, Interleukin 1b; IL-6, Interleukin 6; NFL, neurofilament light; S100B, S-100 calcium binding protein B; sTREM2, soluble triggering receptor expressed on myeloid cells 2; VMA, Vanillymandelic Acid; YKL40, chitinase-3-like protein 1
