# Supplementary material for: Uncovering the Associations of LILRB4 Genotypes With Parkinson's Disease: From Clinical Traits to Potential Pathologies
Source: CNS Neurosci Ther. 2025 Jul 23;31(7):e70522. doi: 10.1111/cns.70522 (PMC12287542; doi:10.1111/cns.70522)
Supplement: Supplementary file 4 — Table S1. [file CNS-31-e70522-s002.zip › cns70522-sup-0013-TableS26-S28@Supplementary Table 26-28 Model 2_The correlation between LILRB4 loci and CSF biomarkers.docx]

**Supplementary Table 26**. Model 2: The correlation between *LILRB4* loci and CSF biomarkers.

| Items | SNP | β(95%CI) | P value | FDR-corrected. P |
| --- | --- | --- | --- | --- |
| 3-MT | rs731170 | -0.063(-0.135-0.009) | 0.087 | 0.606 |
|  | rs1048801 | -0.008(-0.080-0.063) | 0.819 | 0.819 |
|  | rs1749316 | 0.047(-0.030-0.125) | 0.233 | 0.606 |
|  | rs1749317 | -0.027(-0.099-0.045) | 0.466 | 0.606 |
|  | rs1925241 | 0.031(-0.040-0.103) | 0.396 | 0.606 |
|  | rs2569715 | 0.034(-0.037-0.104) | 0.351 | 0.606 |
|  | rs2569716 | -0.025(-0.097-0.047) | 0.496 | 0.606 |
|  | rs3745871 | 0.033(-0.038-0.104) | 0.36 | 0.606 |
|  | rs11540761 | 0.063(-0.033-0.159) | 0.2 | 0.606 |
|  | rs11574576 | -0.023(-0.099-0.054) | 0.563 | 0.619 |
|  | rs28366008 | -0.066(-0.154-0.022) | 0.142 | 0.606 |
| Abeta | rs731170 | -0.115(-0.273-0.042) | 0.156 | 0.693 |
|  | rs1048801 | 0.074(-0.038-0.186) | 0.197 | 0.693 |
|  | rs1749316 | 0.012(-0.110-0.134) | 0.849 | 0.889 |
|  | rs1749317 | -0.038(-0.154-0.077) | 0.519 | 0.889 |
|  | rs1925241 | 0.010(-0.108-0.129) | 0.866 | 0.889 |
|  | rs2569715 | -0.009(-0.125-0.106) | 0.874 | 0.889 |
|  | rs2569716 | 0.034(-0.099-0.167) | 0.62 | 0.889 |
|  | rs3745871 | 0.071(-0.049-0.191) | 0.252 | 0.693 |
|  | rs11540761 | 0.112(-0.026-0.251) | 0.116 | 0.693 |
|  | rs11574576 | -0.008(-0.122-0.106) | 0.889 | 0.889 |
|  | rs28366008 | -0.042(-0.186-0.102) | 0.565 | 0.889 |
| Abeta_1-42 | rs731170 | -0.017(-0.068-0.033) | 0.5 | 0.862 |
|  | rs1048801 | -0.006(-0.055-0.042) | 0.794 | 0.862 |
|  | rs1749316 | 0.026(-0.028-0.081) | 0.344 | 0.862 |
|  | rs1749317 | 0.050(-0.001-0.102) | 0.057 | 0.628 |
|  | rs1925241 | 0.008(-0.039-0.055) | 0.73 | 0.862 |
|  | rs2569715 | -0.014(-0.064-0.036) | 0.578 | 0.862 |
|  | rs2569716 | -0.036(-0.084-0.012) | 0.143 | 0.786 |
|  | rs3745871 | -0.007(-0.055-0.042) | 0.784 | 0.862 |
|  | rs11540761 | 0.005(-0.053-0.063) | 0.862 | 0.862 |
|  | rs11574576 | 0.021(-0.030-0.072) | 0.428 | 0.862 |
|  | rs28366008 | -0.024(-0.080-0.031) | 0.391 | 0.862 |
| a-Synuclein | rs731170 | -0.041(-0.129-0.047) | 0.36 | 0.997 |
|  | rs1048801 | 0.028(-0.059-0.114) | 0.531 | 0.997 |
|  | rs1749316 | 0.020(-0.073-0.113) | 0.67 | 0.997 |
|  | rs1749317 | 0.016(-0.071-0.104) | 0.719 | 0.997 |
|  | rs1925241 | 0.000(-0.081-0.081) | 0.997 | 0.997 |
|  | rs2569715 | -0.041(-0.128-0.045) | 0.351 | 0.997 |
|  | rs2569716 | -0.019(-0.105-0.067) | 0.661 | 0.997 |
|  | rs3745871 | -0.010(-0.093-0.073) | 0.815 | 0.997 |
|  | rs11540761 | -0.005(-0.107-0.096) | 0.918 | 0.997 |
|  | rs11574576 | 0.083(0.000-0.166) | 0.052 | 0.571 |
|  | rs28366008 | -0.008(-0.104-0.088) | 0.866 | 0.997 |
| DOPA | rs731170 | -0.018(-0.091-0.055) | 0.633 | 0.955 |
|  | rs1048801 | -0.007(-0.079-0.065) | 0.855 | 0.955 |
|  | rs1749316 | 0.023(-0.055-0.101) | 0.571 | 0.955 |
|  | rs1749317 | 0.058(-0.013-0.130) | 0.113 | 0.622 |
|  | rs1925241 | 0.005(-0.067-0.077) | 0.886 | 0.955 |
|  | rs2569715 | 0.030(-0.040-0.101) | 0.403 | 0.955 |
|  | rs2569716 | -0.070(-0.141-0.002) | 0.058 | 0.622 |
|  | rs3745871 | 0.002(-0.069-0.073) | 0.955 | 0.955 |
|  | rs11540761 | 0.047(-0.049-0.144) | 0.34 | 0.955 |
|  | rs11574576 | -0.025(-0.102-0.053) | 0.532 | 0.955 |
|  | rs28366008 | 0.010(-0.079-0.099) | 0.829 | 0.955 |
| DOPAC | rs731170 | -0.006(-0.109-0.098) | 0.913 | 0.986 |
|  | rs1048801 | -0.001(-0.103-0.101) | 0.986 | 0.986 |
|  | rs1749316 | -0.089(-0.198-0.021) | 0.115 | 0.385 |
|  | rs1749317 | 0.045(-0.058-0.147) | 0.394 | 0.722 |
|  | rs1925241 | 0.077(-0.025-0.178) | 0.14 | 0.385 |
|  | rs2569715 | -0.006(-0.106-0.094) | 0.906 | 0.986 |
|  | rs2569716 | -0.027(-0.129-0.076) | 0.612 | 0.842 |
|  | rs3745871 | 0.078(-0.022-0.178) | 0.129 | 0.385 |
|  | rs11540761 | 0.152(0.017-0.287) | **0.029** | 0.318 |
|  | rs11574576 | -0.041(-0.150-0.068) | 0.465 | 0.731 |
|  | rs28366008 | 0.087(-0.038-0.212) | 0.176 | 0.387 |
| Dopamine | rs731170 | -0.083(-0.268-0.103) | 0.385 | 0.637 |
|  | rs1048801 | 0.066(-0.118-0.249) | 0.485 | 0.666 |
|  | rs1749316 | -0.005(-0.203-0.194) | 0.963 | 0.999 |
|  | rs1749317 | 0.218(0.038-0.399) | **0.019** | 0.209 |
|  | rs1925241 | 0.086(-0.097-0.269) | 0.356 | 0.637 |
|  | rs2569715 | 0.151(-0.028-0.330) | 0.1 | 0.367 |
|  | rs2569716 | 0.104(-0.079-0.287) | 0.269 | 0.637 |
|  | rs3745871 | 0.077(-0.104-0.258) | 0.406 | 0.637 |
|  | rs11540761 | 0.232(-0.012-0.476) | 0.064 | 0.354 |
|  | rs11574576 | -0.02(-0.217-0.176) | 0.84 | 0.999 |
|  | rs28366008 | 0.000(-0.226-0.226) | 0.999 | 0.999 |
| GFAP | rs731170 | -0.082(-0.155--0.009) | **0.029** | 0.131 |
|  | rs1048801 | -0.008(-0.080-0.064) | 0.829 | 0.959 |
|  | rs1749316 | 0.083(0.006-0.161) | **0.036** | 0.131 |
|  | rs1749317 | 0.027(-0.046-0.100) | 0.466 | 0.959 |
|  | rs1925241 | 0.002(-0.066-0.069) | 0.959 | 0.959 |
|  | rs2569715 | -0.007(-0.080-0.065) | 0.842 | 0.959 |
|  | rs2569716 | -0.081(-0.152--0.010) | **0.027** | 0.131 |
|  | rs3745871 | -0.004(-0.073-0.066) | 0.921 | 0.959 |
|  | rs11540761 | -0.016(-0.101-0.068) | 0.704 | 0.959 |
|  | rs11574576 | 0.016(-0.054-0.086) | 0.659 | 0.959 |
|  | rs28366008 | -0.062(-0.142-0.018) | 0.129 | 0.354 |
| HVA | rs731170 | -0.017(-0.130-0.095) | 0.763 | 0.839 |
|  | rs1048801 | 0.048(-0.063-0.159) | 0.398 | 0.644 |
|  | rs1749316 | -0.075(-0.195-0.045) | 0.222 | 0.64 |
|  | rs1749317 | 0.047(-0.064-0.158) | 0.41 | 0.644 |
|  | rs1925241 | 0.060(-0.051-0.171) | 0.291 | 0.64 |
|  | rs2569715 | -0.020(-0.129-0.089) | 0.721 | 0.839 |
|  | rs2569716 | -0.064(-0.175-0.047) | 0.26 | 0.64 |
|  | rs3745871 | 0.065(-0.045-0.174) | 0.25 | 0.64 |
|  | rs11540761 | 0.139(-0.009-0.287) | 0.067 | 0.64 |
|  | rs11574576 | 0.021(-0.098-0.140) | 0.726 | 0.839 |
|  | rs28366008 | -0.002(-0.139-0.135) | 0.973 | 0.973 |
| IL-1b | rs731170 | -0.034(-0.142-0.075) | 0.545 | 0.808 |
|  | rs1048801 | 0.066(-0.040-0.171) | 0.225 | 0.808 |
|  | rs1749316 | 0.032(-0.088-0.151) | 0.604 | 0.808 |
|  | rs1749317 | 0.052(-0.059-0.162) | 0.361 | 0.808 |
|  | rs1925241 | -0.013(-0.122-0.095) | 0.808 | 0.808 |
|  | rs2569715 | -0.013(-0.119-0.092) | 0.804 | 0.808 |
|  | rs2569716 | 0.030(-0.078-0.138) | 0.591 | 0.808 |
|  | rs3745871 | 0.014(-0.095-0.122) | 0.806 | 0.808 |
|  | rs11540761 | -0.093(-0.241-0.055) | 0.222 | 0.808 |
|  | rs11574576 | 0.043(-0.069-0.156) | 0.452 | 0.808 |
|  | rs28366008 | -0.030(-0.156-0.095) | 0.639 | 0.808 |
| IL-6 | rs731170 | -0.029(-0.112-0.055) | 0.498 | 0.914 |
|  | rs1048801 | -0.018(-0.100-0.064) | 0.664 | 0.914 |
|  | rs1749316 | 0.000(-0.089-0.088) | 0.994 | 0.994 |
|  | rs1749317 | -0.029(-0.112-0.054) | 0.499 | 0.914 |
|  | rs1925241 | 0.050(-0.027-0.126) | 0.205 | 0.914 |
|  | rs2569715 | -0.023(-0.105-0.060) | 0.591 | 0.914 |
|  | rs2569716 | -0.040(-0.121-0.041) | 0.337 | 0.914 |
|  | rs3745871 | 0.035(-0.044-0.114) | 0.384 | 0.914 |
|  | rs11540761 | -0.010(-0.106-0.086) | 0.838 | 0.994 |
|  | rs11574576 | 0.003(-0.076-0.083) | 0.933 | 0.994 |
|  | rs28366008 | -0.079(-0.170-0.011) | 0.087 | 0.914 |
| NFL | rs731170 | -0.004(-0.070-0.061) | 0.894 | 0.99 |
|  | rs1048801 | 0.000(-0.064-0.065) | 0.99 | 0.99 |
|  | rs1749316 | 0.043(-0.026-0.113) | 0.223 | 0.515 |
|  | rs1749317 | 0.050(-0.015-0.115) | 0.134 | 0.491 |
|  | rs1925241 | -0.052(-0.112-0.008) | 0.091 | 0.491 |
|  | rs2569715 | -0.040(-0.104-0.025) | 0.234 | 0.515 |
|  | rs2569716 | -0.001(-0.065-0.063) | 0.974 | 0.99 |
|  | rs3745871 | -0.048(-0.111-0.014) | 0.126 | 0.491 |
|  | rs11540761 | 0.004(-0.072-0.080) | 0.91 | 0.99 |
|  | rs11574576 | 0.021(-0.042-0.083) | 0.518 | 0.95 |
|  | rs28366008 | -0.013(-0.084-0.059) | 0.728 | 0.99 |
| pTau | rs731170 | -0.004(-0.043-0.035) | 0.831 | 0.831 |
|  | rs1048801 | 0.023(-0.013-0.060) | 0.214 | 0.831 |
|  | rs1749316 | -0.011(-0.052-0.030) | 0.593 | 0.831 |
|  | rs1749317 | 0.011(-0.029-0.050) | 0.599 | 0.831 |
|  | rs1925241 | 0.016(-0.020-0.052) | 0.374 | 0.831 |
|  | rs2569715 | -0.014(-0.052-0.024) | 0.47 | 0.831 |
|  | rs2569716 | 0.006(-0.031-0.044) | 0.749 | 0.831 |
|  | rs3745871 | 0.015(-0.022-0.052) | 0.437 | 0.831 |
|  | rs11540761 | 0.011(-0.034-0.055) | 0.633 | 0.831 |
|  | rs11574576 | 0.035(-0.004-0.073) | 0.078 | 0.831 |
|  | rs28366008 | -0.006(-0.049-0.037) | 0.779 | 0.831 |
| S100 | rs731170 | -0.012(-0.058-0.035) | 0.618 | 0.737 |
|  | rs1048801 | -0.003(-0.049-0.043) | 0.898 | 0.898 |
|  | rs1749316 | 0.035(-0.014-0.084) | 0.161 | 0.713 |
|  | rs1749317 | 0.027(-0.019-0.073) | 0.252 | 0.713 |
|  | rs1925241 | -0.018(-0.061-0.024) | 0.401 | 0.713 |
|  | rs2569715 | 0.024(-0.022-0.069) | 0.315 | 0.713 |
|  | rs2569716 | 0.010(-0.035-0.055) | 0.667 | 0.737 |
|  | rs3745871 | -0.021(-0.064-0.023) | 0.36 | 0.713 |
|  | rs11540761 | -0.020(-0.074-0.033) | 0.454 | 0.713 |
|  | rs11574576 | 0.054(0.011-0.098) | **0.015** | 0.169 |
|  | rs28366008 | 0.011(-0.040-0.062) | 0.67 | 0.737 |
| sTREM2 | rs731170 | -0.020(-0.073-0.033) | 0.464 | 0.985 |
|  | rs1048801 | 0.021(-0.031-0.073) | 0.432 | 0.985 |
|  | rs1749316 | -0.001(-0.057-0.056) | 0.985 | 0.985 |
|  | rs1749317 | 0.045(-0.008-0.098) | 0.094 | 0.638 |
|  | rs1925241 | 0.020(-0.029-0.068) | 0.429 | 0.985 |
|  | rs2569715 | -0.008(-0.060-0.045) | 0.779 | 0.985 |
|  | rs2569716 | 0.006(-0.045-0.058) | 0.808 | 0.985 |
|  | rs3745871 | -0.002(-0.052-0.048) | 0.933 | 0.985 |
|  | rs11540761 | 0.009(-0.052-0.070) | 0.78 | 0.985 |
|  | rs11574576 | 0.040(-0.010-0.091) | 0.116 | 0.638 |
|  | rs28366008 | 0.001(-0.057-0.059) | 0.964 | 0.985 |
| tTau | rs731170 | 0.000(-0.037-0.038) | 0.993 | 0.993 |
|  | rs1048801 | 0.012(-0.024-0.047) | 0.518 | 0.855 |
|  | rs1749316 | -0.006(-0.046-0.033) | 0.759 | 0.855 |
|  | rs1749317 | 0.011(-0.026-0.049) | 0.551 | 0.855 |
|  | rs1925241 | 0.012(-0.022-0.047) | 0.487 | 0.855 |
|  | rs2569715 | -0.012(-0.048-0.025) | 0.532 | 0.855 |
|  | rs2569716 | 0.005(-0.03-0.041) | 0.768 | 0.855 |
|  | rs3745871 | 0.008(-0.027-0.043) | 0.662 | 0.855 |
|  | rs11540761 | 0.006(-0.037-0.049) | 0.777 | 0.855 |
|  | rs11574576 | 0.030(-0.007-0.067) | 0.111 | 0.855 |
|  | rs28366008 | -0.007(-0.049-0.034) | 0.729 | 0.855 |
| VMA | rs731170 | 0.005(-0.115-0.125) | 0.94 | 0.981 |
|  | rs1048801 | -0.058(-0.176-0.060) | 0.337 | 0.781 |
|  | rs1749316 | -0.064(-0.191-0.064) | 0.331 | 0.781 |
|  | rs1749317 | -0.001(-0.120-0.117) | 0.981 | 0.981 |
|  | rs1925241 | 0.024(-0.094-0.143) | 0.686 | 0.943 |
|  | rs2569715 | 0.084(-0.032-0.199) | 0.157 | 0.781 |
|  | rs2569716 | 0.056(-0.062-0.175) | 0.352 | 0.781 |
|  | rs3745871 | 0.016(-0.101-0.133) | 0.793 | 0.969 |
|  | rs11540761 | 0.053(-0.106-0.212) | 0.512 | 0.939 |
|  | rs11574576 | 0.060(-0.067-0.186) | 0.355 | 0.781 |
|  | rs28366008 | -0.037(-0.183-0.109) | 0.621 | 0.943 |
| YKL40 | rs731170 | 0.020(-0.077-0.117) | 0.687 | 0.756 |
|  | rs1048801 | -0.038(-0.133-0.057) | 0.434 | 0.756 |
|  | rs1749316 | -0.005(-0.107-0.098) | 0.93 | 0.93 |
|  | rs1749317 | -0.059(-0.155-0.037) | 0.232 | 0.511 |
|  | rs1925241 | -0.024(-0.113-0.065) | 0.599 | 0.756 |
|  | rs2569715 | -0.058(-0.154-0.037) | 0.231 | 0.511 |
|  | rs2569716 | 0.073(-0.021-0.167) | 0.13 | 0.511 |
|  | rs3745871 | 0.021(-0.070-0.113) | 0.652 | 0.756 |
|  | rs11540761 | -0.068(-0.180-0.043) | 0.23 | 0.511 |
|  | rs11574576 | -0.031(-0.123-0.061) | 0.511 | 0.756 |
|  | rs28366008 | 0.081(-0.024-0.186) | 0.132 | 0.511 |

CI, confidence internal; FDR, false discovery rate; 3-MT, 3-Methoxytyramine; Aβ, beta amyloid; Aβ1-42, beta amyloid 1-42; DA, dopamine; DOPA, dihydroxyphenylalanine; DOPAC, dihydroxyphenylacetic acid; FDR, false discovery rate; GFAP, glial fibrillary acid protein; HVA, homovanillic acid; IL-1b, Interleukin 1b; IL-6, Interleukin 6; NFL, neurofilament light; S100B, S-100 calcium binding protein B; sTREM2, soluble triggering receptor expressed on myeloid cells 2; VMA, Vanillymandelic Acid; YKL40, chitinase-3-like protein 1

**Supplementary Table 27**. Model 2: The correlation between *LILRB4* loci and CSF biomarkers in male.

| Items | SNP | β(95%CI) | P value | FDR-corrected. P |
| --- | --- | --- | --- | --- |
| 3-MT | rs731170 | -0.030(-0.116-0.056) | 0.496 | 0.957 |
|  | rs1048801 | -0.001(-0.085-0.083) | 0.984 | 0.984 |
|  | rs1749316 | 0.054(-0.037-0.144) | 0.249 | 0.957 |
|  | rs1749317 | 0.002(-0.080-0.083) | 0.966 | 0.984 |
|  | rs1925241 | 0.014(-0.070-0.098) | 0.744 | 0.957 |
|  | rs2569715 | -0.015(-0.100-0.070) | 0.731 | 0.957 |
|  | rs2569716 | -0.027(-0.108-0.053) | 0.508 | 0.957 |
|  | rs3745871 | 0.012(-0.073-0.097) | 0.783 | 0.957 |
|  | rs11540761 | 0.034(-0.078-0.147) | 0.549 | 0.957 |
|  | rs11574576 | -0.033(-0.123-0.057) | 0.472 | 0.957 |
|  | rs28366008 | -0.052(-0.156-0.051) | 0.321 | 0.957 |
| Abeta | rs731170 | -0.106(-0.311-0.100) | 0.319 | 0.934 |
|  | rs1048801 | -0.014(-0.159-0.130) | 0.848 | 0.934 |
|  | rs1749316 | 0.077(-0.069-0.224) | 0.307 | 0.934 |
|  | rs1749317 | -0.057(-0.204-0.090) | 0.449 | 0.934 |
|  | rs1925241 | -0.059(-0.215-0.097) | 0.464 | 0.934 |
|  | rs2569715 | 0.103(-0.047-0.254) | 0.185 | 0.934 |
|  | rs2569716 | 0.019(-0.173-0.210) | 0.85 | 0.934 |
|  | rs3745871 | 0.007(-0.157-0.170) | 0.934 | 0.934 |
|  | rs11540761 | 0.055(-0.131-0.241) | 0.562 | 0.934 |
|  | rs11574576 | -0.023(-0.183-0.137) | 0.778 | 0.934 |
|  | rs28366008 | 0.028(-0.183-0.239) | 0.797 | 0.934 |
| Abeta_1-42 | rs731170 | -0.011(-0.076-0.053) | 0.733 | 0.827 |
|  | rs1048801 | -0.017(-0.079-0.044) | 0.58 | 0.827 |
|  | rs1749316 | 0.013(-0.056-0.083) | 0.707 | 0.827 |
|  | rs1749317 | 0.052(-0.015-0.119) | 0.131 | 0.48 |
|  | rs1925241 | 0.023(-0.037-0.082) | 0.451 | 0.827 |
|  | rs2569715 | -0.034(-0.099-0.03) | 0.297 | 0.817 |
|  | rs2569716 | -0.058(-0.119-0.004) | 0.066 | 0.48 |
|  | rs3745871 | -0.003(-0.063-0.058) | 0.932 | 0.932 |
|  | rs11540761 | 0.012(-0.060-0.084) | 0.752 | 0.827 |
|  | rs11574576 | 0.052(-0.013-0.116) | 0.115 | 0.48 |
|  | rs28366008 | -0.032(-0.103-0.039) | 0.378 | 0.827 |
| a-Synuclein | rs731170 | -0.036(-0.153-0.080) | 0.541 | 0.975 |
|  | rs1048801 | -0.057(-0.173-0.059) | 0.338 | 0.975 |
|  | rs1749316 | 0.003(-0.124-0.129) | 0.967 | 0.975 |
|  | rs1749317 | 0.007(-0.108-0.122) | 0.905 | 0.975 |
|  | rs1925241 | 0.019(-0.089-0.128) | 0.727 | 0.975 |
|  | rs2569715 | -0.002(-0.119-0.115) | 0.975 | 0.975 |
|  | rs2569716 | 0.020(-0.092-0.132) | 0.724 | 0.975 |
|  | rs3745871 | -0.005(-0.117-0.108) | 0.937 | 0.975 |
|  | rs11540761 | 0.013(-0.116-0.141) | 0.845 | 0.975 |
|  | rs11574576 | 0.072(-0.039-0.184) | 0.204 | 0.975 |
|  | rs28366008 | -0.030(-0.160-0.099) | 0.647 | 0.975 |
| DOPA | rs731170 | -0.012(-0.105-0.081) | 0.8 | 0.961 |
|  | rs1048801 | -0.002(-0.093-0.088) | 0.961 | 0.961 |
|  | rs1749316 | 0.015(-0.083-0.113) | 0.769 | 0.961 |
|  | rs1749317 | 0.072(-0.015-0.158) | 0.107 | 0.937 |
|  | rs1925241 | 0.005(-0.085-0.096) | 0.911 | 0.961 |
|  | rs2569715 | 0.026(-0.065-0.117) | 0.584 | 0.961 |
|  | rs2569716 | -0.061(-0.147-0.025) | 0.17 | 0.937 |
|  | rs3745871 | 0.009(-0.082-0.101) | 0.84 | 0.961 |
|  | rs11540761 | 0.052(-0.068-0.173) | 0.399 | 0.961 |
|  | rs11574576 | -0.032(-0.128-0.065) | 0.524 | 0.961 |
|  | rs28366008 | 0.024(-0.087-0.135) | 0.672 | 0.961 |
| DOPAC | rs731170 | -0.020(-0.146-0.107) | 0.763 | 0.839 |
|  | rs1048801 | 0.027(-0.096-0.150) | 0.67 | 0.839 |
|  | rs1749316 | -0.125(-0.256-0.006) | 0.065 | 0.218 |
|  | rs1749317 | 0.056(-0.063-0.175) | 0.357 | 0.561 |
|  | rs1925241 | 0.110(-0.011-0.231) | 0.079 | 0.218 |
|  | rs2569715 | 0.025(-0.099-0.149) | 0.696 | 0.839 |
|  | rs2569716 | 0.011(-0.107-0.129) | 0.852 | 0.852 |
|  | rs3745871 | 0.129(0.007-0.250) | **0.04** | 0.218 |
|  | rs11540761 | 0.201(0.041-0.361) | **0.015** | 0.168 |
|  | rs11574576 | -0.068(-0.200-0.063) | 0.308 | 0.561 |
|  | rs28366008 | 0.093(-0.057-0.244) | 0.227 | 0.499 |
| Dopamine | rs731170 | -0.074(-0.334-0.186) | 0.58 | 0.978 |
|  | rs1048801 | 0.064(-0.189-0.318) | 0.62 | 0.978 |
|  | rs1749316 | 0.013(-0.261-0.287) | 0.929 | 0.996 |
|  | rs1749317 | 0.277(0.038-0.516) | **0.025** | 0.276 |
|  | rs1925241 | 0.059(-0.194-0.312) | 0.648 | 0.978 |
|  | rs2569715 | 0.222(-0.029-0.473) | 0.086 | 0.475 |
|  | rs2569716 | 0.150(-0.091-0.391) | 0.225 | 0.617 |
|  | rs3745871 | 0.048(-0.207-0.303) | 0.711 | 0.978 |
|  | rs11540761 | 0.252(-0.082-0.587) | 0.143 | 0.523 |
|  | rs11574576 | 0.001(-0.270-0.272) | 0.996 | 0.996 |
|  | rs28366008 | -0.018(-0.33-0.294) | 0.909 | 0.996 |
| GFAP | rs731170 | -0.103(-0.198--0.009) | **0.033** | 0.181 |
|  | rs1048801 | -0.081(-0.175-0.014) | 0.096 | 0.264 |
|  | rs1749316 | 0.131(0.030-0.233) | **0.012** | 0.131 |
|  | rs1749317 | 0.020(-0.074-0.114) | 0.675 | 0.743 |
|  | rs1925241 | -0.003(-0.092-0.085) | 0.94 | 0.94 |
|  | rs2569715 | 0.059(-0.036-0.155) | 0.223 | 0.409 |
|  | rs2569716 | -0.073(-0.163-0.018) | 0.117 | 0.264 |
|  | rs3745871 | -0.022(-0.114-0.070) | 0.639 | 0.743 |
|  | rs11540761 | -0.032(-0.137-0.073) | 0.554 | 0.743 |
|  | rs11574576 | -0.023(-0.114-0.069) | 0.629 | 0.743 |
|  | rs28366008 | -0.084(-0.189-0.021) | 0.12 | 0.264 |
| HVA | rs731170 | 0.038(-0.103-0.180) | 0.596 | 0.82 |
|  | rs1048801 | 0.036(-0.102-0.174) | 0.61 | 0.82 |
|  | rs1749316 | -0.157(-0.303--0.011) | **0.038** | 0.391 |
|  | rs1749317 | 0.035(-0.098-0.168) | 0.607 | 0.82 |
|  | rs1925241 | 0.064(-0.074-0.202) | 0.364 | 0.82 |
|  | rs2569715 | 0.035(-0.103-0.174) | 0.618 | 0.82 |
|  | rs2569716 | 0.000(-0.133-0.132) | 0.997 | 0.997 |
|  | rs3745871 | 0.087(-0.051-0.225) | 0.219 | 0.803 |
|  | rs11540761 | 0.169(-0.012-0.35) | 0.071 | 0.391 |
|  | rs11574576 | 0.015(-0.132-0.163) | 0.839 | 0.923 |
|  | rs28366008 | 0.037(-0.133-0.207) | 0.671 | 0.82 |
| IL-1b | rs731170 | 0.026(-0.132-0.183) | 0.75 | 0.875 |
|  | rs1048801 | 0.078(-0.074-0.230) | 0.32 | 0.875 |
|  | rs1749316 | 0.023(-0.15-0.196) | 0.795 | 0.875 |
|  | rs1749317 | 0.082(-0.070-0.234) | 0.292 | 0.875 |
|  | rs1925241 | -0.030(-0.185-0.125) | 0.707 | 0.875 |
|  | rs2569715 | -0.026(-0.177-0.124) | 0.733 | 0.875 |
|  | rs2569716 | 0.055(-0.091-0.201) | 0.463 | 0.875 |
|  | rs3745871 | -0.011(-0.171-0.148) | 0.89 | 0.89 |
|  | rs11540761 | -0.144(-0.359-0.071) | 0.195 | 0.875 |
|  | rs11574576 | 0.089(-0.070-0.249) | 0.278 | 0.875 |
|  | rs28366008 | -0.024(-0.202-0.153) | 0.788 | 0.875 |
| IL-6 | rs731170 | -0.048(-0.146-0.051) | 0.344 | 0.998 |
|  | rs1048801 | 0.000(-0.098-0.098) | 0.998 | 0.998 |
|  | rs1749316 | 0.058(-0.048-0.164) | 0.284 | 0.998 |
|  | rs1749317 | -0.023(-0.120-0.074) | 0.639 | 0.998 |
|  | rs1925241 | 0.005(-0.087-0.097) | 0.918 | 0.998 |
|  | rs2569715 | -0.038(-0.137-0.061) | 0.453 | 0.998 |
|  | rs2569716 | -0.014(-0.108-0.081) | 0.775 | 0.998 |
|  | rs3745871 | 0.000(-0.096-0.095) | 0.992 | 0.998 |
|  | rs11540761 | -0.001(-0.11-0.107) | 0.983 | 0.998 |
|  | rs11574576 | 0.014(-0.081-0.108) | 0.778 | 0.998 |
|  | rs28366008 | -0.087(-0.196-0.021) | 0.116 | 0.998 |
| NFL | rs731170 | -0.005(-0.081-0.072) | 0.908 | 0.999 |
|  | rs1048801 | -0.016(-0.092-0.060) | 0.675 | 0.999 |
|  | rs1749316 | 0.047(-0.035-0.130) | 0.261 | 0.935 |
|  | rs1749317 | 0.013(-0.062-0.088) | 0.734 | 0.999 |
|  | rs1925241 | -0.038(-0.109-0.033) | 0.298 | 0.935 |
|  | rs2569715 | -0.056(-0.133-0.020) | 0.15 | 0.935 |
|  | rs2569716 | -0.005(-0.078-0.068) | 0.89 | 0.999 |
|  | rs3745871 | -0.036(-0.11-0.038) | 0.34 | 0.935 |
|  | rs11540761 | 0.000(-0.084-0.084) | 0.999 | 0.999 |
|  | rs11574576 | 0.018(-0.055-0.091) | 0.628 | 0.999 |
|  | rs28366008 | -0.017(-0.102-0.067) | 0.688 | 0.999 |
| pTau | rs731170 | 0.004(-0.045-0.053) | 0.87 | 0.87 |
|  | rs1048801 | -0.019(-0.065-0.027) | 0.42 | 0.578 |
|  | rs1749316 | -0.026(-0.079-0.026) | 0.32 | 0.578 |
|  | rs1749317 | -0.024(-0.074-0.026) | 0.354 | 0.578 |
|  | rs1925241 | 0.029(-0.017-0.074) | 0.217 | 0.578 |
|  | rs2569715 | 0.026(-0.022-0.075) | 0.286 | 0.578 |
|  | rs2569716 | 0.022(-0.025-0.069) | 0.364 | 0.578 |
|  | rs3745871 | 0.012(-0.034-0.059) | 0.598 | 0.657 |
|  | rs11540761 | 0.024(-0.031-0.079) | 0.392 | 0.578 |
|  | rs11574576 | 0.043(-0.006-0.092) | 0.088 | 0.578 |
|  | rs28366008 | -0.017(-0.072-0.038) | 0.552 | 0.657 |
| S100 | rs731170 | -0.029(-0.087-0.029) | 0.332 | 0.458 |
|  | rs1048801 | -0.047(-0.105-0.011) | 0.113 | 0.257 |
|  | rs1749316 | 0.055(-0.008-0.118) | 0.087 | 0.257 |
|  | rs1749317 | -0.001(-0.058-0.057) | 0.98 | 0.98 |
|  | rs1925241 | -0.027(-0.081-0.027) | 0.333 | 0.458 |
|  | rs2569715 | 0.081(0.023-0.139) | **0.006** | 0.069 |
|  | rs2569716 | 0.045(-0.011-0.100) | 0.117 | 0.257 |
|  | rs3745871 | -0.003(-0.087-0.026) | 0.292 | 0.458 |
|  | rs11540761 | -0.029(-0.093-0.036) | 0.383 | 0.468 |
|  | rs11574576 | 0.045(-0.011-0.100) | 0.115 | 0.257 |
|  | rs28366008 | -0.009(-0.074-0.056) | 0.79 | 0.869 |
| sTREM2 | rs731170 | -0.039(-0.103-0.025) | 0.23 | 0.849 |
|  | rs1048801 | -0.020(-0.084-0.044) | 0.54 | 0.849 |
|  | rs1749316 | 0.025(-0.044-0.094) | 0.481 | 0.849 |
|  | rs1749317 | 0.020(-0.043-0.084) | 0.529 | 0.849 |
|  | rs1925241 | 0.031(-0.029-0.090) | 0.313 | 0.849 |
|  | rs2569715 | 0.008(-0.057-0.072) | 0.816 | 0.935 |
|  | rs2569716 | 0.030(-0.031-0.092) | 0.335 | 0.849 |
|  | rs3745871 | -0.007(-0.069-0.055) | 0.833 | 0.935 |
|  | rs11540761 | 0.007(-0.064-0.078) | 0.85 | 0.935 |
|  | rs11574576 | 0.037(-0.025-0.098) | 0.245 | 0.849 |
|  | rs28366008 | 0.000(-0.072-0.071) | 0.993 | 0.993 |
| tTau | rs731170 | 0.010(-0.037-0.058) | 0.672 | 0.739 |
|  | rs1048801 | -0.028(-0.073-0.016) | 0.214 | 0.687 |
|  | rs1749316 | -0.022(-0.073-0.028) | 0.389 | 0.687 |
|  | rs1749317 | -0.026(-0.075-0.023) | 0.297 | 0.687 |
|  | rs1925241 | 0.022(-0.022-0.066) | 0.328 | 0.687 |
|  | rs2569715 | 0.023(-0.025-0.070) | 0.346 | 0.687 |
|  | rs2569716 | 0.016(-0.030-0.062) | 0.499 | 0.687 |
|  | rs3745871 | 0.007(-0.038-0.052) | 0.759 | 0.759 |
|  | rs11540761 | 0.012(-0.042-0.066) | 0.66 | 0.739 |
|  | rs11574576 | 0.038(-0.009-0.086) | 0.116 | 0.687 |
|  | rs28366008 | -0.019(-0.072-0.034) | 0.486 | 0.687 |
| VMA | rs731170 | -0.057(-0.216-0.103) | 0.487 | 0.595 |
|  | rs1048801 | -0.139(-0.292-0.015) | 0.079 | 0.435 |
|  | rs1749316 | -0.083(-0.250-0.085) | 0.335 | 0.553 |
|  | rs1749317 | -0.045(-0.195-0.105) | 0.559 | 0.615 |
|  | rs1925241 | 0.068(-0.087-0.223) | 0.394 | 0.553 |
|  | rs2569715 | 0.146(-0.008-0.299) | 0.067 | 0.435 |
|  | rs2569716 | 0.075(-0.074-0.223) | 0.325 | 0.553 |
|  | rs3745871 | 0.082(-0.074-0.237) | 0.308 | 0.553 |
|  | rs11540761 | 0.130(-0.076-0.336) | 0.221 | 0.553 |
|  | rs11574576 | 0.071(-0.095-0.237) | 0.402 | 0.553 |
|  | rs28366008 | -0.024(-0.215-0.168) | 0.809 | 0.809 |
| YKL40 | rs731170 | -0.023(-0.092-0.046) | 0.509 | 0.906 |
|  | rs1048801 | -0.031(-0.099-0.038) | 0.383 | 0.906 |
|  | rs1749316 | 0.042(-0.033-0.116) | 0.274 | 0.906 |
|  | rs1749317 | -0.002(-0.070-0.066) | 0.946 | 0.946 |
|  | rs1925241 | -0.018(-0.082-0.046) | 0.59 | 0.906 |
|  | rs2569715 | -0.008(-0.077-0.061) | 0.824 | 0.906 |
|  | rs2569716 | 0.041(-0.025-0.106) | 0.227 | 0.906 |
|  | rs3745871 | -0.028(-0.094-0.039) | 0.415 | 0.906 |
|  | rs11540761 | -0.010(-0.086-0.066) | 0.803 | 0.906 |
|  | rs11574576 | 0.014(-0.052-0.080) | 0.682 | 0.906 |
|  | rs28366008 | 0.039(-0.037-0.115) | 0.316 | 0.906 |

CI, confidence internal; FDR, false discovery rate; 3-MT, 3-Methoxytyramine; Aβ, beta amyloid; Aβ1-42, beta amyloid 1-42; DA, dopamine; DOPA, dihydroxyphenylalanine; DOPAC, dihydroxyphenylacetic acid; FDR, false discovery rate; GFAP, glial fibrillary acid protein; HVA, homovanillic acid; IL-1b, Interleukin 1b; IL-6, Interleukin 6; NFL, neurofilament light; S100B, S-100 calcium binding protein B; sTREM2, soluble triggering receptor expressed on myeloid cells 2; VMA, Vanillymandelic Acid; YKL40, chitinase-3-like protein 1

**Supplementary Table 28**. Model 2: The correlation between *LILRB4* loci and CSF biomarkers in female.

| Items | SNP | β(95%CI) | P value | FDR-corrected. P |
| --- | --- | --- | --- | --- |
| 3-MT | rs731170 | -0.138(-0.268--0.008) | **0.044** | 0.241 |
|  | rs1048801 | -0.024(-0.162-0.114) | 0.735 | 0.898 |
|  | rs1749316 | 0.034(-0.115-0.183) | 0.658 | 0.898 |
|  | rs1749317 | -0.133(-0.283-0.018) | 0.092 | 0.336 |
|  | rs1925241 | 0.069(-0.068-0.205) | 0.331 | 0.520 |
|  | rs2569715 | 0.133(0.010-0.256) | **0.039** | 0.241 |
|  | rs2569716 | -0.007(-0.165-0.151) | 0.932 | 0.932 |
|  | rs3745871 | 0.080(-0.049-0.210) | 0.230 | 0.467 |
|  | rs11540761 | 0.145(-0.040-0.330) | 0.132 | 0.363 |
|  | rs11574576 | -0.010(-0.161-0.140) | 0.893 | 0.932 |
|  | rs28366008 | -0.099(-0.267-0.069) | 0.255 | 0.467 |
| Abeta | rs731170 | -0.127(-0.375-0.121) | 0.323 | 0.534 |
|  | rs1048801 | 0.244(0.079-0.408) | **0.007** | 0.078 |
|  | rs1749316 | -0.055(-0.291-0.180) | 0.648 | 0.792 |
|  | rs1749317 | -0.008(-0.195-0.178) | 0.931 | 0.931 |
|  | rs1925241 | 0.064(-0.121-0.248) | 0.505 | 0.694 |
|  | rs2569715 | -0.157(-0.328-0.013) | 0.081 | 0.448 |
|  | rs2569716 | 0.097(-0.099-0.293) | 0.340 | 0.534 |
|  | rs3745871 | 0.114(-0.067-0.296) | 0.228 | 0.534 |
|  | rs11540761 | 0.154(-0.060-0.368) | 0.170 | 0.534 |
|  | rs11574576 | 0.024(-0.141-0.189) | 0.777 | 0.855 |
|  | rs28366008 | -0.123(-0.328-0.082) | 0.250 | 0.534 |
| Abeta_1-42 | rs731170 | -0.027(-0.11-0.055) | 0.517 | 0.943 |
|  | rs1048801 | 0.008(-0.073-0.089) | 0.846 | 0.943 |
|  | rs1749316 | 0.044(-0.043-0.131) | 0.324 | 0.943 |
|  | rs1749317 | 0.054(-0.029-0.137) | 0.206 | 0.943 |
|  | rs1925241 | -0.017(-0.094-0.061) | 0.671 | 0.943 |
|  | rs2569715 | 0.016(-0.062-0.094) | 0.685 | 0.943 |
|  | rs2569716 | -0.003(-0.082-0.076) | 0.943 | 0.943 |
|  | rs3745871 | -0.013(-0.093-0.067) | 0.746 | 0.943 |
|  | rs11540761 | -0.005(-0.104-0.094) | 0.927 | 0.943 |
|  | rs11574576 | -0.033(-0.118-0.052) | 0.444 | 0.943 |
|  | rs28366008 | -0.009(-0.100-0.081) | 0.840 | 0.943 |
| a-Synuclein | rs731170 | -0.047(-0.174-0.080) | 0.471 | 0.724 |
|  | rs1048801 | 0.186(0.071-0.301) | **0.002** | **0.022** |
|  | rs1749316 | 0.040(-0.090-0.171) | 0.544 | 0.724 |
|  | rs1749317 | 0.029(-0.104-0.161) | 0.673 | 0.740 |
|  | rs1925241 | -0.031(-0.144-0.082) | 0.592 | 0.724 |
|  | rs2569715 | -0.117(-0.237-0.004) | 0.060 | 0.298 |
|  | rs2569716 | -0.105(-0.233-0.022) | 0.108 | 0.298 |
|  | rs3745871 | -0.013(-0.129-0.102) | 0.819 | 0.819 |
|  | rs11540761 | -0.060(-0.221-0.101) | 0.468 | 0.724 |
|  | rs11574576 | 0.107(-0.012-0.226) | 0.082 | 0.298 |
|  | rs28366008 | 0.051(-0.084-0.186) | 0.462 | 0.724 |
| DOPA | rs731170 | -0.035(-0.146-0.076) | 0.543 | 0.975 |
|  | rs1048801 | -0.013(-0.127-0.100) | 0.821 | 0.975 |
|  | rs1749316 | 0.044(-0.077-0.166) | 0.478 | 0.975 |
|  | rs1749317 | -0.009(-0.137-0.118) | 0.887 | 0.975 |
|  | rs1925241 | 0.001(-0.113-0.114) | 0.991 | 0.991 |
|  | rs2569715 | 0.043(-0.062-0.148) | 0.425 | 0.975 |
|  | rs2569716 | -0.079(-0.207-0.048) | 0.230 | 0.975 |
|  | rs3745871 | -0.008(-0.116-0.100) | 0.882 | 0.975 |
|  | rs11540761 | 0.055(-0.100-0.210) | 0.487 | 0.975 |
|  | rs11574576 | -0.033(-0.156-0.090) | 0.604 | 0.975 |
|  | rs28366008 | -0.025(-0.164-0.114) | 0.726 | 0.975 |
| DOPAC | rs731170 | 0.019(-0.162-0.201) | 0.834 | 0.998 |
|  | rs1048801 | -0.062(-0.245-0.121) | 0.509 | 0.998 |
|  | rs1749316 | 0.000(-0.199-0.199) | 0.998 | 0.998 |
|  | rs1749317 | -0.018(-0.225-0.189) | 0.865 | 0.998 |
|  | rs1925241 | -0.005(-0.189-0.178) | 0.954 | 0.998 |
|  | rs2569715 | -0.065(-0.235-0.105) | 0.459 | 0.998 |
|  | rs2569716 | -0.131(-0.337-0.076) | 0.221 | 0.998 |
|  | rs3745871 | -0.022(-0.197-0.152) | 0.804 | 0.998 |
|  | rs11540761 | 0.052(-0.200-0.305) | 0.685 | 0.998 |
|  | rs11574576 | 0.001(-0.200-0.201) | 0.994 | 0.998 |
|  | rs28366008 | 0.070(-0.156-0.295) | 0.549 | 0.998 |
| Dopamine | rs731170 | -0.096(-0.265-0.073) | 0.271 | 0.745 |
|  | rs1048801 | 0.064(-0.109-0.236) | 0.475 | 0.746 |
|  | rs1749316 | -0.050(-0.237-0.138) | 0.607 | 0.752 |
|  | rs1749317 | 0.075(-0.119-0.270) | 0.451 | 0.746 |
|  | rs1925241 | 0.156(-0.011-0.323) | 0.074 | 0.641 |
|  | rs2569715 | 0.004(-0.158-0.166) | 0.966 | 0.966 |
|  | rs2569716 | -0.077(-0.275-0.120) | 0.447 | 0.746 |
|  | rs3745871 | 0.131(-0.029-0.292) | 0.117 | 0.641 |
|  | rs11540761 | 0.160(-0.074-0.394) | 0.188 | 0.689 |
|  | rs11574576 | -0.042(-0.231-0.148) | 0.669 | 0.752 |
|  | rs28366008 | 0.045(-0.169-0.258) | 0.683 | 0.752 |
| GFAP | rs731170 | -0.042(-0.158-0.073) | 0.475 | 0.870 |
|  | rs1048801 | 0.129(0.022-0.236) | **0.020** | 0.108 |
|  | rs1749316 | 0.007(-0.111-0.125) | 0.908 | 0.908 |
|  | rs1749317 | 0.062(-0.057-0.182) | 0.309 | 0.680 |
|  | rs1925241 | 0.011(-0.092-0.113) | 0.838 | 0.908 |
|  | rs2569715 | -0.143(-0.251--0.036) | **0.010** | 0.108 |
|  | rs2569716 | -0.108(-0.223-0.007) | 0.070 | 0.192 |
|  | rs3745871 | 0.025(-0.079-0.129) | 0.640 | 0.879 |
|  | rs11540761 | 0.021(-0.125-0.168) | 0.777 | 0.908 |
|  | rs11574576 | 0.106(-0.002-0.213) | 0.057 | 0.192 |
|  | rs28366008 | -0.030(-0.153-0.093) | 0.632 | 0.879 |
| HVA | rs731170 | -0.141(-0.322-0.04) | 0.134 | 0.532 |
|  | rs1048801 | 0.079(-0.108-0.266) | 0.413 | 0.754 |
|  | rs1749316 | 0.120(-0.081-0.321) | 0.247 | 0.679 |
|  | rs1749317 | 0.069(-0.142-0.281) | 0.522 | 0.754 |
|  | rs1925241 | 0.047(-0.140-0.235) | 0.622 | 0.760 |
|  | rs2569715 | -0.130(-0.301-0.042) | 0.145 | 0.532 |
|  | rs2569716 | -0.265(-0.465--0.064) | **0.013** | 0.143 |
|  | rs3745871 | 0.021(-0.158-0.200) | 0.823 | 0.839 |
|  | rs11540761 | 0.080(-0.178-0.337) | 0.548 | 0.754 |
|  | rs11574576 | 0.021(-0.184-0.227) | 0.839 | 0.839 |
|  | rs28366008 | -0.096(-0.326-0.135) | 0.420 | 0.754 |
| IL-1b | rs731170 | -0.139(-0.255--0.022) | **0.026** | 0.286 |
|  | rs1048801 | 0.045(-0.078-0.168) | 0.476 | 0.927 |
|  | rs1749316 | 0.047(-0.090-0.184) | 0.504 | 0.927 |
|  | rs1749317 | -0.018(-0.161-0.124) | 0.803 | 0.927 |
|  | rs1925241 | 0.017(-0.110-0.144) | 0.794 | 0.927 |
|  | rs2569715 | 0.011(-0.117-0.139) | 0.870 | 0.927 |
|  | rs2569716 | -0.040(-0.184-0.105) | 0.595 | 0.927 |
|  | rs3745871 | 0.053(-0.067-0.173) | 0.393 | 0.927 |
|  | rs11540761 | -0.008(-0.177-0.161) | 0.927 | 0.927 |
|  | rs11574576 | -0.045(-0.182-0.093) | 0.530 | 0.927 |
|  | rs28366008 | -0.041(-0.193-0.110) | 0.597 | 0.927 |
| IL-6 | rs731170 | -0.009(-0.166-0.149) | 0.914 | 0.975 |
|  | rs1048801 | -0.036(-0.185-0.113) | 0.635 | 0.975 |
|  | rs1749316 | -0.106(-0.265-0.054) | 0.197 | 0.664 |
|  | rs1749317 | -0.058(-0.220-0.105) | 0.487 | 0.893 |
|  | rs1925241 | 0.142(0.006-0.279) | **0.044** | 0.482 |
|  | rs2569715 | 0.002(-0.149-0.154) | 0.975 | 0.975 |
|  | rs2569716 | -0.095(-0.253-0.063) | 0.242 | 0.664 |
|  | rs3745871 | 0.113(-0.028-0.253) | 0.119 | 0.652 |
|  | rs11540761 | -0.026(-0.225-0.173) | 0.796 | 0.975 |
|  | rs11574576 | -0.016(-0.165-0.133) | 0.832 | 0.975 |
|  | rs28366008 | -0.072(-0.239-0.095) | 0.399 | 0.877 |
| NFL | rs731170 | -0.007(-0.134-0.120) | 0.911 | 0.952 |
|  | rs1048801 | 0.030(-0.090-0.150) | 0.623 | 0.952 |
|  | rs1749316 | 0.028(-0.102-0.158) | 0.675 | 0.952 |
|  | rs1749317 | 0.108(-0.022-0.238) | 0.107 | 0.952 |
|  | rs1925241 | -0.078(-0.190-0.034) | 0.174 | 0.952 |
|  | rs2569715 | 0.008(-0.114-0.130) | 0.899 | 0.952 |
|  | rs2569716 | 0.020(-0.109-0.148) | 0.762 | 0.952 |
|  | rs3745871 | -0.063(-0.177-0.051) | 0.283 | 0.952 |
|  | rs11540761 | 0.027(-0.134-0.188) | 0.742 | 0.952 |
|  | rs11574576 | 0.006(-0.115-0.126) | 0.928 | 0.952 |
|  | rs28366008 | -0.004(-0.139-0.131) | 0.952 | 0.952 |
| pTau | rs731170 | -0.017(-0.082-0.048) | 0.600 | 0.843 |
|  | rs1048801 | 0.097(0.035-0.158) | **0.002** | **0.025** |
|  | rs1749316 | 0.014(-0.053-0.082) | 0.679 | 0.843 |
|  | rs1749317 | 0.062(-0.002-0.126) | 0.060 | 0.221 |
|  | rs1925241 | -0.006(-0.066-0.054) | 0.843 | 0.843 |
|  | rs2569715 | -0.071(-0.131--0.012) | **0.020** | 0.108 |
|  | rs2569716 | -0.020(-0.081-0.042) | 0.527 | 0.843 |
|  | rs3745871 | 0.017(-0.045-0.079) | 0.590 | 0.843 |
|  | rs11540761 | -0.014(-0.090-0.062) | 0.714 | 0.843 |
|  | rs11574576 | 0.020(-0.042-0.082) | 0.530 | 0.843 |
|  | rs28366008 | 0.010(-0.061-0.081) | 0.780 | 0.843 |
| S100 | rs731170 | 0.020(-0.057-0.097) | 0.614 | 0.931 |
|  | rs1048801 | 0.080(0.008-0.152) | **0.031** | 0.084 |
|  | rs1749316 | 0.013(-0.066-0.092) | 0.751 | 0.931 |
|  | rs1749317 | 0.113(0.036-0.190) | **0.005** | 0.054 |
|  | rs1925241 | -0.006(-0.074-0.063) | 0.873 | 0.960 |
|  | rs2569715 | -0.094(-0.166--0.022) | **0.012** | 0.057 |
|  | rs2569716 | -0.078(-0.155--0.001) | **0.049** | 0.109 |
|  | rs3745871 | -0.011(-0.081-0.059) | 0.762 | 0.931 |
|  | rs11540761 | -0.001(-0.099-0.097) | 0.987 | 0.987 |
|  | rs11574576 | 0.089(0.018-0.161) | **0.016** | 0.057 |
|  | rs28366008 | 0.038(-0.044-0.120) | 0.368 | 0.675 |
| sTREM2 | rs731170 | 0.018(-0.078-0.114) | 0.718 | 0.942 |
|  | rs1048801 | 0.099(0.009-0.188) | **0.033** | 0.358 |
|  | rs1749316 | -0.055(-0.153-0.043) | 0.274 | 0.898 |
|  | rs1749317 | 0.087(-0.011-0.186) | 0.086 | 0.474 |
|  | rs1925241 | 0.002(-0.083-0.088) | 0.957 | 0.957 |
|  | rs2569715 | -0.029(-0.121-0.063) | 0.540 | 0.942 |
|  | rs2569716 | -0.041(-0.138-0.056) | 0.408 | 0.898 |
|  | rs3745871 | 0.0130(-0.074-0.100) | 0.768 | 0.942 |
|  | rs11540761 | 0.018(-0.104-0.140) | 0.774 | 0.942 |
|  | rs11574576 | 0.041(-0.050-0.132) | 0.380 | 0.898 |
|  | rs28366008 | 0.010(-0.093-0.112) | 0.856 | 0.942 |
| tTau | rs731170 | -0.016(-0.077-0.045) | 0.606 | 0.882 |
|  | rs1048801 | 0.081(0.023-0.138) | **0.007** | 0.072 |
|  | rs1749316 | 0.020(-0.043-0.083) | 0.525 | 0.882 |
|  | rs1749317 | 0.068(0.008-0.128) | **0.026** | 0.126 |
|  | rs1925241 | -0.006(-0.062-0.050) | 0.837 | 0.882 |
|  | rs2569715 | -0.061(-0.116--0.005) | **0.034** | 0.126 |
|  | rs2569716 | -0.012(-0.070-0.045) | 0.676 | 0.882 |
|  | rs3745871 | 0.008(-0.050-0.066) | 0.782 | 0.882 |
|  | rs11540761 | -0.005(-0.076-0.066) | 0.882 | 0.882 |
|  | rs11574576 | 0.016(-0.043-0.074) | 0.601 | 0.882 |
|  | rs28366008 | 0.011(-0.055-0.078) | 0.738 | 0.882 |
| VMA | rs731170 | 0.134(-0.020-0.288) | 0.096 | 0.473 |
|  | rs1048801 | 0.134(-0.022-0.291) | 0.100 | 0.473 |
|  | rs1749316 | -0.016(-0.190-0.159) | 0.861 | 0.891 |
|  | rs1749317 | 0.116(-0.063-0.294) | 0.210 | 0.577 |
|  | rs1925241 | -0.081(-0.240-0.079) | 0.327 | 0.600 |
|  | rs2569715 | -0.038(-0.188-0.112) | 0.621 | 0.853 |
|  | rs2569716 | 0.013(-0.172-0.198) | 0.891 | 0.891 |
|  | rs3745871 | -0.118(-0.267-0.031) | 0.129 | 0.473 |
|  | rs11540761 | -0.121(-0.340-0.098) | 0.286 | 0.600 |
|  | rs11574576 | 0.015(-0.161-0.192) | 0.864 | 0.891 |
|  | rs28366008 | -0.069(-0.267-0.129) | 0.498 | 0.783 |
| YKL40 | rs731170 | 0.095(-0.163-0.354) | 0.470 | 0.595 |
|  | rs1048801 | -0.045(-0.290-0.200) | 0.720 | 0.792 |
|  | rs1749316 | -0.094(-0.358-0.170) | 0.487 | 0.595 |
|  | rs1749317 | -0.224(-0.489-0.041) | 0.100 | 0.474 |
|  | rs1925241 | -0.031(-0.260-0.199) | 0.794 | 0.794 |
|  | rs2569715 | -0.140(-0.388-0.107) | 0.268 | 0.474 |
|  | rs2569716 | 0.161(-0.099-0.421) | 0.228 | 0.474 |
|  | rs3745871 | 0.123(-0.110-0.356) | 0.302 | 0.474 |
|  | rs11540761 | -0.212(-0.538-0.113) | 0.204 | 0.474 |
|  | rs11574576 | -0.141(-0.385-0.102) | 0.259 | 0.474 |
|  | rs28366008 | 0.157(-0.116-0.431) | 0.263 | 0.474 |

CI, confidence internal; FDR, false discovery rate; 3-MT, 3-Methoxytyramine; Aβ, beta amyloid; Aβ1-42, beta amyloid 1-42; DA, dopamine; DOPA, dihydroxyphenylalanine; DOPAC, dihydroxyphenylacetic acid; FDR, false discovery rate; GFAP, glial fibrillary acid protein; HVA, homovanillic acid; IL-1b, Interleukin 1b; IL-6, Interleukin 6; NFL, neurofilament light; S100B, S-100 calcium binding protein B; sTREM2, soluble triggering receptor expressed on myeloid cells 2; VMA, Vanillymandelic Acid; YKL40, chitinase-3-like protein 1
