# Supplementary material for: Uncovering the Associations of LILRB4 Genotypes With Parkinson's Disease: From Clinical Traits to Potential Pathologies
Source: CNS Neurosci Ther. 2025 Jul 23;31(7):e70522. doi: 10.1111/cns.70522 (PMC12287542; doi:10.1111/cns.70522)
Supplement: Supplementary file 4 — Table S1. [file CNS-31-e70522-s002.zip › cns70522-sup-0014-TableS29-S31@Supplementary Table 29-31 Model 1_The correlation between LILRB4 loci and scales.docx]

**Supplementary Table 29**. Model 1: The correlation between *LILRB4* loci and scales.

| Items | SNP | β(95%CI) | P | FDR-corrected.P |
| --- | --- | --- | --- | --- |
| MDS-UPDRS Part I (P) | rs731170 | 0.411(0.039-0.783) | **0.031** | 0.169 |
|  | rs1048801 | 0.142(-0.205-0.489) | 0.423 | 0.927 |
|  | rs1749316 | -0.048(-0.437-0.34) | 0.808 | 1.000 |
|  | rs1749317 | 0.273(-0.098-0.645) | 0.150 | 0.435 |
|  | rs1925241 | 0.246(-0.095-0.588) | 0.158 | 0.435 |
|  | rs2569715 | -0.118(-0.476-0.240) | 0.519 | 0.927 |
|  | rs2569716 | 0.021(-0.336-0.378) | 0.907 | 1.000 |
|  | rs3745871 | 0.453(0.104-0.802) | **0.011** | 0.123 |
|  | rs11540761 | 0.001(-0.422-0.425) | 0.995 | 1.000 |
|  | rs11574576 | -0.100(-0.465-0.264) | 0.590 | 0.927 |
|  | rs28366008 | 0.000(-0.413-0.413) | 1.000 | 1.000 |
| MDS-UPDRS Part I (R) | rs731170 | -0.252(-0.445--0.058) | **0.011** | 0.123 |
|  | rs1048801 | -0.002(-0.183-0.179) | 0.982 | 0.982 |
|  | rs1749316 | 0.151(-0.051-0.354) | 0.143 | 0.464 |
|  | rs1749317 | -0.027(-0.221-0.167) | 0.786 | 0.926 |
|  | rs1925241 | 0.035(-0.144-0.213) | 0.702 | 0.926 |
|  | rs2569715 | -0.019(-0.206-0.168) | 0.842 | 0.926 |
|  | rs2569716 | -0.134(-0.320-0.052) | 0.160 | 0.464 |
|  | rs3745871 | 0.128(-0.054-0.311) | 0.169 | 0.464 |
|  | rs11540761 | -0.112(-0.332-0.109) | 0.322 | 0.708 |
|  | rs11574576 | 0.036(-0.154-0.226) | 0.711 | 0.926 |
|  | rs28366008 | 0.073(-0.142-0.288) | 0.505 | 0.925 |
| MDS-UPDRS Part II | rs731170 | -0.326(-0.851-0.199) | 0.224 | 0.845 |
|  | rs1048801 | 0.074(-0.416-0.563) | 0.768 | 0.845 |
|  | rs1749316 | 0.105(-0.443-0.652) | 0.708 | 0.845 |
|  | rs1749317 | -0.107(-0.633-0.418) | 0.690 | 0.845 |
|  | rs1925241 | 0.136(-0.346-0.618) | 0.580 | 0.845 |
|  | rs2569715 | -0.115(-0.620-0.390) | 0.656 | 0.845 |
|  | rs2569716 | -0.162(-0.666-0.342) | 0.529 | 0.845 |
|  | rs3745871 | 0.320(-0.174-0.814) | 0.205 | 0.845 |
|  | rs11540761 | 0.166(-0.431-0.763) | 0.586 | 0.845 |
|  | rs11574576 | -0.050(-0.565-0.465) | 0.850 | 0.850 |
|  | rs28366008 | -0.117(-0.699-0.464) | 0.693 | 0.845 |
| MDS-UPDRS Part III | rs731170 | -1.023(-2.280-0.233) | 0.111 | 0.610 |
|  | rs1048801 | -0.205(-1.380-0.971) | 0.733 | 0.873 |
|  | rs1749316 | 0.187(-1.124-1.498) | 0.780 | 0.873 |
|  | rs1749317 | 0.217(-1.038-1.472) | 0.735 | 0.873 |
|  | rs1925241 | 0.319(-0.835-1.472) | 0.589 | 0.873 |
|  | rs2569715 | 0.162(-1.048-1.372) | 0.793 | 0.873 |
|  | rs2569716 | 0.175(-1.031-1.38) | 0.776 | 0.873 |
|  | rs3745871 | 1.064(-0.117-2.244) | 0.078 | 0.610 |
|  | rs11540761 | 0.766(-0.662-2.193) | 0.294 | 0.873 |
|  | rs11574576 | 0.067(-1.166-1.299) | 0.916 | 0.916 |
|  | rs28366008 | -0.583(-1.974-0.809) | 0.412 | 0.873 |
| MDS-UPDRS Part IV | rs731170 | -0.495(-0.876--0.114) | **0.011** | **0.030** |
|  | rs1048801 | 0.467(0.108-0.826) | **0.011** | **0.030** |
|  | rs1749316 | 0.188(-0.208-0.585) | 0.352 | 0.484 |
|  | rs1749317 | 0.550(0.165-0.935) | **0.005** | **0.030** |
|  | rs1925241 | 0.222(-0.119-0.563) | 0.203 | 0.319 |
|  | rs2569715 | -0.051(-0.413-0.311) | 0.783 | 0.858 |
|  | rs2569716 | 0.089(-0.281-0.460) | 0.637 | 0.779 |
|  | rs3745871 | 0.287(-0.276-0.849) | 0.109 | 0.240 |
|  | rs11540761 | 0.286(-0.139-0.711) | 0.188 | 0.319 |
|  | rs11574576 | 0.033(-0.330-0.397) | 0.858 | 0.858 |
|  | rs28366008 | -0.564(-0.986--0.141) | **0.009** | **0.030** |
| ADL | rs731170 | 0.347(-0.534-1.227) | 0.440 | 0.708 |
|  | rs1048801 | -0.233(-1.054-0.589) | 0.579 | 0.708 |
|  | rs1749316 | 0.420(-0.492-1.332) | 0.367 | 0.708 |
|  | rs1749317 | -0.171(-1.066-0.724) | 0.708 | 0.779 |
|  | rs1925241 | -0.410(-1.206-0.385) | 0.312 | 0.708 |
|  | rs2569715 | -0.106(-0.949-0.737) | 0.805 | 0.805 |
|  | rs2569716 | 0.432(-0.413-1.276) | 0.317 | 0.708 |
|  | rs3745871 | -0.737(-1.548-0.075) | 0.076 | 0.708 |
|  | rs11540761 | -0.364(-1.359-0.631) | 0.474 | 0.708 |
|  | rs11574576 | -0.282(-1.132-0.568) | 0.516 | 0.708 |
|  | rs28366008 | 0.553(-0.418-1.523) | 0.265 | 0.708 |
| BJLOT | rs731170 | 0.169(-0.313-0.652) | 0.492 | 0.676 |
|  | rs1048801 | 0.222(-0.228-0.672) | 0.334 | 0.676 |
|  | rs1749316 | 0.003(-0.498-0.505) | 0.989 | 0.989 |
|  | rs1749317 | -0.123(-0.605-0.358) | 0.616 | 0.753 |
|  | rs1925241 | -0.281(-0.723-0.161) | 0.214 | 0.676 |
|  | rs2569715 | -0.265(-0.728-0.198) | 0.262 | 0.676 |
|  | rs2569716 | 0.206(-0.256-0.668) | 0.382 | 0.676 |
|  | rs3745871 | -0.344(-0.798-0.109) | 0.137 | 0.676 |
|  | rs11540761 | -0.079(-0.625-0.467) | 0.777 | 0.855 |
|  | rs11574576 | -0.170(-0.642-0.303) | 0.482 | 0.676 |
|  | rs28366008 | 0.495(-0.036-1.027) | 0.074 | 0.676 |
| BNT | rs731170 | 1.092(-0.140-2.324) | 0.083 | 0.228 |
|  | rs1048801 | 0.503(-0.661-1.668) | 0.397 | 0.575 |
|  | rs1749316 | 0.463(-1.216-2.141) | 0.476 | 0.575 |
|  | rs1749317 | -0.421(-1.693-0.850) | 0.516 | 0.575 |
|  | rs1925241 | -1.227(-2.339--0.115) | **0.026** | 0.094 |
|  | rs2569715 | -0.451(-1.654-0.751) | 0.462 | 0.575 |
|  | rs2569716 | 0.387(-0.800-1.575) | 0.523 | 0.575 |
|  | rs3745871 | -1.377(-2.510--0.243) | **0.018** | 0.094 |
|  | rs11540761 | -1.752(-3.178--0.326) | **0.016** | 0.094 |
|  | rs11574576 | -0.727(-1.958-0.504) | 0.248 | 0.545 |
|  | rs28366008 | 0.149(-1.196-1.494) | 0.828 | 0.828 |
| HVLT | rs731170 | 0.915(-0.240-2.071) | 0.121 | 0.443 |
|  | rs1048801 | 0.341(-0.737-1.419) | 0.535 | 0.630 |
|  | rs1749316 | -0.476(-1.678-0.726) | 0.438 | 0.630 |
|  | rs1749317 | -0.579(-1.733-0.575) | 0.326 | 0.630 |
|  | rs1925241 | -0.211(-1.270-0.848) | 0.696 | 0.696 |
|  | rs2569715 | 0.319(-0.790-1.429) | 0.573 | 0.630 |
|  | rs2569716 | 1.098(-0.007-2.203) | 0.052 | 0.285 |
|  | rs3745871 | -0.450(-1.536-0.636) | 0.417 | 0.630 |
|  | rs11540761 | -0.476(-1.786-0.835) | 0.477 | 0.630 |
|  | rs11574576 | -1.170(-2.297--0.042) | **0.042** | 0.285 |
|  | rs28366008 | 0.511(-0.766-1.787) | 0.433 | 0.630 |
| LNS | rs731170 | -0.046(-0.355-0.264) | 0.773 | 0.850 |
|  | rs1048801 | 0.194(-0.094-0.483) | 0.187 | 0.646 |
|  | rs1749316 | -0.155(-0.477-0.167) | 0.346 | 0.656 |
|  | rs1749317 | 0.102(-0.208-0.412) | 0.518 | 0.712 |
|  | rs1925241 | 0.241(-0.042-0.524) | 0.095 | 0.523 |
|  | rs2569715 | -0.139(-0.437-0.158) | 0.358 | 0.656 |
|  | rs2569716 | 0.179(-0.117-0.476) | 0.235 | 0.646 |
|  | rs3745871 | 0.109(-0.181-0.400) | 0.462 | 0.712 |
|  | rs11540761 | 0.338(-0.013-0.688) | 0.059 | 0.523 |
|  | rs11574576 | -0.011(-0.313-0.291) | 0.944 | 0.944 |
|  | rs28366008 | 0.064(-0.278-0.406) | 0.713 | 0.850 |
| LXFLUEA | rs731170 | -0.371(-1.020-0.278) | 0.263 | 0.909 |
|  | rs1048801 | -0.036(-0.656-0.583) | 0.909 | 0.909 |
|  | rs1749316 | 0.457(-0.216-1.129) | 0.184 | 0.909 |
|  | rs1749317 | 0.068(-0.607-0.743) | 0.843 | 0.909 |
|  | rs1925241 | -0.108(-0.699-0.482) | 0.719 | 0.909 |
|  | rs2569715 | 0.133(-0.501-0.766) | 0.682 | 0.909 |
|  | rs2569716 | -0.272(-0.904-0.359) | 0.398 | 0.909 |
|  | rs3745871 | -0.115(-0.721-0.490) | 0.709 | 0.909 |
|  | rs11540761 | -0.114(-0.871-0.644) | 0.769 | 0.909 |
|  | rs11574576 | -0.712(-1.357--0.067) | **0.031** | 0.341 |
|  | rs28366008 | -0.061(-0.777-0.656) | 0.868 | 0.909 |
| LXFLUEF | rs731170 | 0.169(-0.537-0.874) | 0.639 | 0.946 |
|  | rs1048801 | -0.226(-0.899-0.446) | 0.327 | 0.946 |
|  | rs1749316 | -0.058(-0.789-0.673) | 0.876 | 0.946 |
|  | rs1749317 | 0.125(-0.605-0.855) | 0.737 | 0.946 |
|  | rs1925241 | -0.086(-0.727-0.554) | 0.792 | 0.946 |
|  | rs2569715 | 0.161(-0.526-0.848) | 0.646 | 0.946 |
|  | rs2569716 | 0.126(-0.56-0.812) | 0.719 | 0.946 |
|  | rs3745871 | -0.239(-0.895-0.416) | 0.474 | 0.946 |
|  | rs11540761 | -0.037(-0.860-0.785) | 0.929 | 0.946 |
|  | rs11574576 | -0.926(-1.622--0.229) | **0.009** | 0.104 |
|  | rs28366008 | 0.027(-0.751-0.805) | 0.946 | 0.946 |
| LXFLUES | rs731170 | -0.229(-0.957-0.499) | 0.538 | 0.784 |
|  | rs1048801 | -0.242(-0.937-0.452) | 0.494 | 0.784 |
|  | rs1749316 | 0.490(-0.267-1.247) | 0.205 | 0.751 |
|  | rs1749317 | -0.142(-0.899-0.614) | 0.713 | 0.784 |
|  | rs1925241 | -0.204(-0.866-0.457) | 0.545 | 0.784 |
|  | rs2569715 | -0.155(-0.865-0.554) | 0.668 | 0.784 |
|  | rs2569716 | -0.645(-1.350-0.060) | 0.074 | 0.405 |
|  | rs3745871 | -0.331(-1.009-0.348) | 0.340 | 0.784 |
|  | rs11540761 | 0.105(-0.744-0.954) | 0.809 | 0.809 |
|  | rs11574576 | -0.742(-1.466--0.019) | **0.045** | 0.405 |
|  | rs28366008 | -0.201(-1.004-0.602) | 0.624 | 0.784 |
| MoCA | rs731170 | 0.250(-0.032-0.532) | 0.083 | 0.275 |
|  | rs1048801 | 0.220(-0.042-0.483) | 0.100 | 0.275 |
|  | rs1749316 | -0.009(-0.303-0.285) | 0.951 | 0.951 |
|  | rs1749317 | -0.076(-0.358-0.205) | 0.596 | 0.771 |
|  | rs1925241 | -0.176(-0.435-0.083) | 0.183 | 0.288 |
|  | rs2569715 | 0.066(-0.205-0.338) | 0.631 | 0.771 |
|  | rs2569716 | 0.253(-0.018-0.523) | 0.067 | 0.275 |
|  | rs3745871 | -0.207(-0.472-0.058) | 0.125 | 0.275 |
|  | rs11540761 | -0.062(-0.383-0.258) | 0.703 | 0.773 |
|  | rs11574576 | -0.193(-0.469-0.082) | 0.170 | 0.288 |
|  | rs28366008 | 0.411(0.100-0.722) | **0.010** | 0.107 |
| SFT | rs731170 | 0.155(-0.969-1.280) | 0.787 | 0.865 |
|  | rs1048801 | 0.505(-0.543-1.552) | 0.345 | 0.633 |
|  | rs1749316 | 1.002(-0.167-2.171) | 0.094 | 0.311 |
|  | rs1749317 | 0.468(-0.656-1.592) | 0.415 | 0.652 |
|  | rs1925241 | -0.541(-1.571-0.490) | 0.251 | 0.553 |
|  | rs2569715 | 0.182(-0.898-1.262) | 0.742 | 0.865 |
|  | rs2569716 | 0.001(-1.076-1.078) | 0.999 | 0.999 |
|  | rs3745871 | -0.855(-1.911-0.201) | 0.113 | 0.311 |
|  | rs11540761 | -0.398(-1.169-0.374) | 0.536 | 0.736 |
|  | rs11574576 | -2.135(-3.225--1.046) | **0.000** | **0.001** |
|  | rs28366008 | 1.177(-0.064-2.418) | 0.063 | 0.311 |

CI, confidence internal; FDR, false discovery rate; ADL, Modified Schwab & England Activities of Daily Living Test; BJLOT, Benton Judgement of Line Orientation; BNT, Modified Boston Naming Test; FDR, false discovery rate; HVLT, Hopkins Verbal Learning Test; LNS, Letter-Number Sequencing Test; LXFLUEA, Lexical Fluency-A; LXFLUEF, Lexical Fluency-F; LXFLUES, Lexical Fluency-S; MoCA, Montreal Cognitive Assessment; SFT, semantic fluency test.

**Supplementary Table 30**. Model 1: The correlation between *LILRB4* loci and scales in male.

| Items | SNP | β(95%CI) | P | FDR-corrected. P |
| --- | --- | --- | --- | --- |
| MDS-UPDRS Part I (P) | rs731170 | -0.369(-0.809-0.071) | 0.101 | 0.818 |
|  | rs1048801 | 0.001(-0.408-0.410) | 0.996 | 0.996 |
|  | rs1749316 | 0.111(-0.359-0.580) | 0.644 | 0.885 |
|  | rs1749317 | 0.278(-0.168-0.724) | 0.223 | 0.818 |
|  | rs1925241 | 0.166(-0.241-0.574) | 0.424 | 0.855 |
|  | rs2569715 | -0.061(-0.498-0.377) | 0.786 | 0.961 |
|  | rs2569716 | -0.124(-0.548-0.300) | 0.567 | 0.885 |
|  | rs3745871 | 0.266(-0.149-0.682) | 0.209 | 0.818 |
|  | rs11540761 | -0.023(-0.517-0.471) | 0.926 | 0.996 |
|  | rs11574576 | 0.207(-0.230-0.644) | 0.354 | 0.855 |
|  | rs28366008 | -0.183(-0.675-0.309) | 0.466 | 0.855 |
| MDS-UPDRS Part I (R) | rs731170 | -0.227(-0.448--0.005) | **0.045** | 0.249 |
|  | rs1048801 | -0.123(-0.329-0.083) | 0.241 | 0.435 |
|  | rs1749316 | 0.131(-0.105-0.367) | 0.277 | 0.435 |
|  | rs1749317 | 0.095(-0.130-0.319) | 0.410 | 0.501 |
|  | rs1925241 | 0.122(-0.083-0.327) | 0.243 | 0.435 |
|  | rs2569715 | -0.067(-0.287-0.153) | 0.552 | 0.607 |
|  | rs2569716 | -0.342(-0.553--0.130) | **0.002** | **0.018** |
|  | rs3745871 | 0.131(-0.078-0.340) | 0.221 | 0.435 |
|  | rs11540761 | -0.164(-0.412-0.084) | 0.197 | 0.435 |
|  | rs11574576 | 0.105(-0.115-0.325) | 0.351 | 0.482 |
|  | rs28366008 | 0.034(-0.213-0.282) | 0.787 | 0.787 |
| MDS-UPDRS Part II | rs731170 | -0.118(-0.770-0.534) | 0.723 | 0.884 |
|  | rs1048801 | 0.035(-0.571-0.641) | 0.910 | 0.910 |
|  | rs1749316 | 0.139(-0.555-0.833) | 0.694 | 0.884 |
|  | rs1749317 | 0.084(-0.579-0.748) | 0.803 | 0.884 |
|  | rs1925241 | 0.083(-0.520-0.686) | 0.786 | 0.884 |
|  | rs2569715 | -0.368(-1.015-0.278) | 0.265 | 0.884 |
|  | rs2569716 | -0.471(-1.098-0.155) | 0.141 | 0.884 |
|  | rs3745871 | 0.113(-0.502-0.729) | 0.718 | 0.884 |
|  | rs11540761 | -0.103(-0.834-0.627) | 0.782 | 0.884 |
|  | rs11574576 | 0.238(-0.409-0.884) | 0.472 | 0.884 |
|  | rs28366008 | -0.182(-0.910-0.545) | 0.624 | 0.884 |
| MDS-UPDRS Part III | rs731170 | -0.883(-2.524-0.758) | 0.292 | 0.777 |
|  | rs1048801 | -0.162(-1.691-1.368) | 0.836 | 0.836 |
|  | rs1749316 | 0.628(-1.119-2.375) | 0.482 | 0.777 |
|  | rs1749317 | 0.644(-1.018-2.307) | 0.448 | 0.777 |
|  | rs1925241 | -0.318(-1.837-1.201) | 0.682 | 0.777 |
|  | rs2569715 | -0.750(-2.379-0.879) | 0.367 | 0.777 |
|  | rs2569716 | -0.689(-2.268-0.890) | 0.393 | 0.777 |
|  | rs3745871 | 0.505(-1.044-2.053) | 0.523 | 0.777 |
|  | rs11540761 | -0.354(-2.193-1.485) | 0.706 | 0.777 |
|  | rs11574576 | 0.450(-1.182-2.083) | 0.589 | 0.777 |
|  | rs28366008 | -0.852(-2.682-0.979) | 0.362 | 0.777 |
| MDS-UPDRS Part IV | rs731170 | -0.296(-0.794-0.202) | 0.245 | 0.538 |
|  | rs1048801 | 0.578(0.107-1.049) | **0.017** | 0.183 |
|  | rs1749316 | 0.123(-0.408-0.654) | 0.651 | 0.716 |
|  | rs1749317 | 0.517(0.017-1.016) | **0.043** | 0.239 |
|  | rs1925241 | 0.126(-0.329-0.581) | 0.588 | 0.716 |
|  | rs2569715 | 0.045(-0.451-0.542) | 0.858 | 0.858 |
|  | rs2569716 | 0.253(-0.240-0.745) | 0.315 | 0.564 |
|  | rs3745871 | 0.217(-0.247-0.681) | 0.359 | 0.564 |
|  | rs11540761 | 0.46(-0.093-1.014) | 0.104 | 0.286 |
|  | rs11574576 | 0.116(-0.368-0.601) | 0.638 | 0.716 |
|  | rs28366008 | -0.516(-1.075-0.043) | 0.071 | 0.262 |
| ADL | rs731170 | -0.465(-1.536-0.607) | 0.396 | 0.946 |
|  | rs1048801 | -0.145(-1.136-0.846) | 0.774 | 0.946 |
|  | rs1749316 | 0.354(-0.776-1.484) | 0.540 | 0.946 |
|  | rs1749317 | -0.340(-1.432-0.753) | 0.543 | 0.946 |
|  | rs1925241 | 0.243(-0.736-1.221) | 0.627 | 0.946 |
|  | rs2569715 | 0.025(-1.035-1.085) | 0.963 | 0.982 |
|  | rs2569716 | 0.745(-0.278-1.769) | 0.154 | 0.847 |
|  | rs3745871 | 0.175(-0.816-1.166) | 0.729 | 0.946 |
|  | rs11540761 | 0.977(-0.217-2.170) | 0.109 | 0.847 |
|  | rs11574576 | 0.012(-1.026-1.049) | 0.982 | 0.982 |
|  | rs28366008 | 0.456(-0.725-1.636) | 0.450 | 0.946 |
| BJLOT | rs731170 | -0.407(-0.955-0.141) | 0.146 | 0.612 |
|  | rs1048801 | 0.454(-0.054-0.962) | 0.080 | 0.612 |
|  | rs1749316 | 0.113(-0.471-0.697) | 0.705 | 0.854 |
|  | rs1749317 | -0.093(-0.651-0.466) | 0.744 | 0.854 |
|  | rs1925241 | 0.221(-0.287-0.730) | 0.394 | 0.619 |
|  | rs2569715 | -0.262(-0.805-0.282) | 0.346 | 0.619 |
|  | rs2569716 | 0.050(-0.479-0.578) | 0.854 | 0.854 |
|  | rs3745871 | -0.050(-0.570-0.470) | 0.850 | 0.854 |
|  | rs11540761 | 0.386(-0.227-1.000) | 0.218 | 0.612 |
|  | rs11574576 | 0.303(-0.243-0.849) | 0.278 | 0.612 |
|  | rs28366008 | 0.359(-0.253-0.970) | 0.251 | 0.612 |
| BNT | rs731170 | 0.697(-0.614-2.008) | 0.298 | 0.561 |
|  | rs1048801 | 0.792(-0.445-2.030) | 0.211 | 0.561 |
|  | rs1749316 | 0.331(-1.069-1.730) | 0.643 | 0.727 |
|  | rs1749317 | -0.488(-1.849-0.873) | 0.483 | 0.694 |
|  | rs1925241 | -0.68(-1.873-0.513) | 0.265 | 0.561 |
|  | rs2569715 | -0.687(-2.001-0.627) | 0.306 | 0.561 |
|  | rs2569716 | 0.282(-0.979-1.544) | 0.661 | 0.727 |
|  | rs3745871 | -1.122(-2.344-0.100) | 0.073 | 0.402 |
|  | rs11540761 | -1.607(-3.096--0.119) | **0.035** | 0.387 |
|  | rs11574576 | -0.226(-1.565-1.113) | 0.741 | 0.741 |
|  | rs28366008 | 0.490(-0.948-1.928) | 0.505 | 0.694 |
| HVLT | rs731170 | 0.296(-1.149-1.741) | 0.688 | 0.972 |
|  | rs1048801 | 0.586(-0.755-1.927) | 0.392 | 0.863 |
|  | rs1749316 | -0.214(-1.750-1.323) | 0.785 | 0.972 |
|  | rs1749317 | -0.959(-2.426-0.508) | 0.201 | 0.737 |
|  | rs1925241 | 0.238(-1.098-1.574) | 0.727 | 0.972 |
|  | rs2569715 | 0.679(-0.752-2.110) | 0.353 | 0.863 |
|  | rs2569716 | 2.076(0.698-3.453) | **0.003** | **0.036** |
|  | rs3745871 | -0.005(-1.369-1.360) | 0.995 | 0.995 |
|  | rs11540761 | -0.214(-1.831-1.403) | 0.795 | 0.972 |
|  | rs11574576 | -0.934(-2.364-0.496) | 0.201 | 0.737 |
|  | rs28366008 | 0.064(-1.547-1.676) | 0.938 | 0.995 |
| LNS | rs731170 | -0.183(-0.566-0.200) | 0.349 | 0.640 |
|  | rs1048801 | 0.224(-0.131-0.580) | 0.216 | 0.590 |
|  | rs1749316 | -0.058(-0.465-0.350) | 0.781 | 0.955 |
|  | rs1749317 | 0.057(-0.333-0.447) | 0.774 | 0.955 |
|  | rs1925241 | 0.363(0.010-0.716) | **0.045** | 0.164 |
|  | rs2569715 | -0.001(-0.381-0.379) | 0.996 | 0.996 |
|  | rs2569716 | 0.441(0.074-0.807) | **0.019** | 0.164 |
|  | rs3745871 | 0.205(-0.157-0.566) | 0.268 | 0.590 |
|  | rs11540761 | 0.458(0.031-0.885) | **0.036** | 0.164 |
|  | rs11574576 | 0.120(-0.259-0.500) | 0.535 | 0.841 |
|  | rs28366008 | 0.024(-0.404-0.451) | 0.914 | 0.996 |
| LXFLUEA | rs731170 | -0.495(-1.341-0.351) | 0.383 | 0.842 |
|  | rs1048801 | 0.199(-0.606-1.005) | 0.628 | 0.842 |
|  | rs1749316 | 0.214(-0.692-1.121) | 0.644 | 0.842 |
|  | rs1749317 | 0.134(-0.748-1.017) | 0.766 | 0.842 |
|  | rs1925241 | 0.332(-0.441-1.105) | 0.400 | 0.842 |
|  | rs2569715 | -0.033(-0.879-0.814) | 0.939 | 0.939 |
|  | rs2569716 | 0.216(-0.610-1.041) | 0.609 | 0.842 |
|  | rs3745871 | 0.363(-0.436-1.163) | 0.374 | 0.842 |
|  | rs11540761 | -0.245(-1.068-0.578) | 0.619 | 0.842 |
|  | rs11574576 | 0.781(-0.074-1.635) | 0.074 | 0.817 |
|  | rs28366008 | -0.177(-1.115-0.761) | 0.712 | 0.842 |
| LXFLUEF | rs731170 | 0.410(-0.51-1.329) | 0.252 | 0.867 |
|  | rs1048801 | -0.127(-1.002-0.749) | 0.777 | 0.889 |
|  | rs1749316 | -0.505(-1.488-0.478) | 0.315 | 0.867 |
|  | rs1749317 | 0.133(-0.826-1.092) | 0.785 | 0.889 |
|  | rs1925241 | 0.218(-0.622-1.058) | 0.611 | 0.889 |
|  | rs2569715 | -0.019(-0.939-0.900) | 0.967 | 0.967 |
|  | rs2569716 | 0.461(-0.435-1.356) | 0.314 | 0.867 |
|  | rs3745871 | -0.108(-0.977-0.761) | 0.808 | 0.889 |
|  | rs11540761 | -0.172(-1.219-0.876) | 0.748 | 0.889 |
|  | rs11574576 | -0.958(-1.885--0.031) | **0.044** | 0.481 |
|  | rs28366008 | -0.201(-1.221-0.818) | 0.699 | 0.889 |
| LXFLUES | rs731170 | -0.488(-1.404-0.429) | 0.298 | 0.841 |
|  | rs1048801 | 0.047(-0.827-0.921) | 0.916 | 0.943 |
|  | rs1749316 | 0.203(-0.787-1.193) | 0.688 | 0.841 |
|  | rs1749317 | -0.21(-1.166-0.747) | 0.668 | 0.841 |
|  | rs1925241 | 0.435(-0.404-1.273) | 0.310 | 0.841 |
|  | rs2569715 | -0.425(-1.340-0.491) | 0.364 | 0.841 |
|  | rs2569716 | -0.353(-1.247-0.540) | 0.439 | 0.841 |
|  | rs3745871 | 0.316(-0.552-1.183) | 0.476 | 0.841 |
|  | rs11540761 | 0.038(-1.007-1.083) | 0.943 | 0.943 |
|  | rs11574576 | -0.716(-1.644-0.212) | 0.131 | 0.841 |
|  | rs28366008 | -0.217(-1.234-0.800) | 0.676 | 0.841 |
| MoCA | rs731170 | 0.039(-0.297-0.376) | 0.819 | 0.923 |
|  | rs1048801 | 0.198(-0.114-0.510) | 0.213 | 0.662 |
|  | rs1749316 | 0.018(-0.341-0.376) | 0.923 | 0.923 |
|  | rs1749317 | -0.120(-0.461-0.220) | 0.489 | 0.896 |
|  | rs1925241 | 0.071(-0.240-0.383) | 0.654 | 0.923 |
|  | rs2569715 | 0.196(-0.138-0.529) | 0.250 | 0.662 |
|  | rs2569716 | 0.323(0.001-0.645) | 0.050 | 0.276 |
|  | rs3745871 | -0.027(-0.345-0.291) | 0.868 | 0.923 |
|  | rs11540761 | 0.199(-0.178-0.576) | 0.301 | 0.662 |
|  | rs11574576 | -0.053(-0.387-0.281) | 0.755 | 0.923 |
|  | rs28366008 | 0.390(0.016-0.764) | **0.042** | 0.276 |
| SFT | rs731170 | -0.577(-1.998-0.844) | 0.426 | 0.873 |
|  | rs1048801 | 0.995(-0.322-2.312) | 0.139 | 0.511 |
|  | rs1749316 | 0.659(-0.852-2.170) | 0.393 | 0.873 |
|  | rs1749317 | 0.435(-1.010-1.880) | 0.556 | 0.873 |
|  | rs1925241 | 0.040(-1.275-1.354) | 0.953 | 0.970 |
|  | rs2569715 | -0.027(-1.437-1.382) | 0.970 | 0.970 |
|  | rs2569716 | 0.411(-0.955-1.778) | 0.556 | 0.873 |
|  | rs3745871 | 0.109(-1.233-1.451) | 0.873 | 0.970 |
|  | rs11540761 | 0.095(-1.497-1.686) | 0.907 | 0.970 |
|  | rs11574576 | -1.960(-3.359--0.561) | **0.006** | 0.069 |
|  | rs28366008 | 1.432(-0.148-3.013) | 0.076 | 0.419 |

CI, confidence internal; FDR, false discovery rate; ADL, Modified Schwab & England Activities of Daily Living Test; BJLOT, Benton Judgement of Line Orientation; BNT, Modified Boston Naming Test; FDR, false discovery rate; HVLT, Hopkins Verbal Learning Test; LNS, Letter-Number Sequencing Test; LXFLUEA, Lexical Fluency-A; LXFLUEF, Lexical Fluency-F; LXFLUES, Lexical Fluency-S; MoCA, Montreal Cognitive Assessment; SFT, semantic fluency test.

**Supplementary Table 31**. Model 1: The correlation between *LILRB4* loci and scales in female.

| Items | SNP | β(95%CI) | P | FDR-corrected. P |
| --- | --- | --- | --- | --- |
| MDS-UPDRS Part I (P) | rs731170 | -0.476(-1.140-0.188) | 0.161 | 0.534 |
|  | rs1048801 | 0.370(-0.254-0.995) | 0.243 | 0.534 |
|  | rs1749316 | -0.279(-0.950-0.392) | 0.416 | 0.547 |
|  | rs1749317 | 0.253(-0.399-0.905) | 0.448 | 0.547 |
|  | rs1925241 | 0.366(-0.236-0.968) | 0.234 | 0.534 |
|  | rs2569715 | -0.189(-0.800-0.423) | 0.546 | 0.600 |
|  | rs2569716 | 0.255(-0.378-0.888) | 0.431 | 0.547 |
|  | rs3745871 | 0.747(0.129-1.364) | **0.018** | 0.202 |
|  | rs11540761 | 0.045(-0.730-0.819) | 0.910 | 0.910 |
|  | rs11574576 | -0.587(-1.224-0.049) | 0.072 | 0.394 |
|  | rs28366008 | 0.287(-0.439-1.014) | 0.439 | 0.547 |
| MDS-UPDRS Part I (R) | rs731170 | -0.288(-0.646-0.071) | 0.117 | 0.709 |
|  | rs1048801 | 0.190(-0.148-0.527) | 0.272 | 0.709 |
|  | rs1749316 | 0.183(-0.180-0.545) | 0.325 | 0.709 |
|  | rs1749317 | -0.239(-0.591-0.113) | 0.185 | 0.709 |
|  | rs1925241 | -0.108(-0.434-0.218) | 0.515 | 0.709 |
|  | rs2569715 | 0.062(-0.269-0.393) | 0.715 | 0.787 |
|  | rs2569716 | 0.201(-0.141-0.543) | 0.251 | 0.709 |
|  | rs3745871 | 0.119(-0.218-0.456) | 0.489 | 0.709 |
|  | rs11540761 | -0.021(-0.439-0.398) | 0.923 | 0.923 |
|  | rs11574576 | -0.093(-0.440-0.253) | 0.597 | 0.730 |
|  | rs28366008 | 0.138(-0.255-0.531) | 0.491 | 0.709 |
| MDS-UPDRS Part II | rs731170 | -0.665(-1.548-0.217) | 0.141 | 0.699 |
|  | rs1048801 | 0.138(-0.692-0.968) | 0.745 | 0.911 |
|  | rs1749316 | 0.054(-0.838-0.947) | 0.905 | 0.974 |
|  | rs1749317 | -0.399(-1.264-0.467) | 0.368 | 0.809 |
|  | rs1925241 | 0.219(-0.583-1.02) | 0.594 | 0.817 |
|  | rs2569715 | 0.238(-0.574-1.051) | 0.566 | 0.817 |
|  | rs2569716 | 0.330(-0.512-1.172) | 0.443 | 0.811 |
|  | rs3745871 | 0.650(-0.175-1.475) | 0.124 | 0.699 |
|  | rs11540761 | 0.64(-0.388-1.667) | 0.223 | 0.699 |
|  | rs11574576 | -0.497(-1.35-0.356) | 0.254 | 0.699 |
|  | rs28366008 | -0.016(-0.983-0.951) | 0.974 | 0.974 |
| MDS-UPDRS Part III | rs731170 | -1.226(-3.174-0.722) | 0.218 | 0.400 |
|  | rs1048801 | -0.318(-2.152-1.515) | 0.734 | 0.807 |
|  | rs1749316 | -0.452(-2.421-1.516) | 0.653 | 0.798 |
|  | rs1749317 | -0.556(-2.469-1.357) | 0.569 | 0.783 |
|  | rs1925241 | 1.277(-0.485-3.039) | 0.156 | 0.344 |
|  | rs2569715 | 1.494(-0.291-3.279) | 0.102 | 0.281 |
|  | rs2569716 | 1.559(-0.291-3.409) | 0.100 | 0.281 |
|  | rs3745871 | 1.931(0.118-3.744) | **0.038** | 0.207 |
|  | rs11540761 | 2.722(0.473-4.970) | **0.018** | 0.201 |
|  | rs11574576 | -0.629(-2.504-1.246) | 0.511 | 0.783 |
|  | rs28366008 | 0.149(-1.983-2.281) | 0.891 | 0.891 |
| MDS-UPDRS Part IV | rs731170 | -0.825(-1.416--0.233) | **0.007** | 0.075 |
|  | rs1048801 | 0.316(-0.242-0.875) | 0.268 | 0.491 |
|  | rs1749316 | 0.284(-0.313-0.882) | 0.351 | 0.552 |
|  | rs1749317 | 0.623(0.014-1.232) | **0.046** | 0.190 |
|  | rs1925241 | 0.362(-0.154-0.878) | 0.170 | 0.375 |
|  | rs2569715 | -0.174(-0.703-0.355) | 0.521 | 0.716 |
|  | rs2569716 | -0.137(-0.699-0.426) | 0.634 | 0.775 |
|  | rs3745871 | 0.398(-0.139-0.934) | 0.148 | 0.375 |
|  | rs11540761 | 0.015(-0.648-0.678) | 0.965 | 0.965 |
|  | rs11574576 | -0.076(-0.628-0.476) | 0.788 | 0.867 |
|  | rs28366008 | -0.645(-1.293-0.002) | 0.052 | 0.190 |
| ADL | rs731170 | 1.526(0.023-3.029) | **0.048** | 0.155 |
|  | rs1048801 | -0.335(-1.762-1.091) | 0.645 | 0.805 |
|  | rs1749316 | 0.523(-1.002-2.047) | 0.502 | 0.789 |
|  | rs1749317 | 0.168(-1.365-1.700) | 0.830 | 0.913 |
|  | rs1925241 | -1.310(-2.651-0.030) | 0.056 | 0.155 |
|  | rs2569715 | -0.311(-1.692-1.070) | 0.659 | 0.805 |
|  | rs2569716 | -0.032(-1.487-1.424) | 0.966 | 0.966 |
|  | rs3745871 | -2.073(-3.446--0.699) | **0.003** | **0.025** |
|  | rs11540761 | -2.508(-4.223--0.793) | **0.004** | **0.025** |
|  | rs11574576 | -0.634(-2.094-0.826) | 0.395 | 0.789 |
|  | rs28366008 | 0.652(-1.014-2.318) | 0.444 | 0.789 |
| BJLOT | rs731170 | 1.107(0.219-1.995) | **0.015** | 0.083 |
|  | rs1048801 | -0.132(-0.975-0.711) | 0.759 | 0.835 |
|  | rs1749316 | -0.162(-1.060-0.735) | 0.723 | 0.835 |
|  | rs1749317 | -0.075(-0.948-0.798) | 0.866 | 0.866 |
|  | rs1925241 | -1.042(-1.841--0.243) | **0.011** | 0.083 |
|  | rs2569715 | -0.326(-1.174-0.523) | 0.438 | 0.603 |
|  | rs2569716 | 0.448(-0.374-1.270) | 0.301 | 0.474 |
|  | rs3745871 | -0.796(-1.625-0.033) | 0.061 | 0.177 |
|  | rs11540761 | -0.895(-1.924-0.135) | 0.090 | 0.197 |
|  | rs11574576 | -0.81(-1.666-0.045) | 0.064 | 0.177 |
|  | rs28366008 | 0.669(-0.301-1.639) | 0.177 | 0.325 |
| BNT | rs731170 | 1.737(-0.714-4.189) | 0.167 | 0.473 |
|  | rs1048801 | 0.074(-2.248-2.397) | 0.950 | 0.950 |
|  | rs1749316 | 0.586(-1.830-3.002) | 0.635 | 0.950 |
|  | rs1749317 | -0.251(-2.766-2.263) | 0.845 | 0.950 |
|  | rs1925241 | -2.189(-4.381-0.002) | 0.052 | 0.473 |
|  | rs2569715 | -0.180(-2.482-2.122) | 0.878 | 0.950 |
|  | rs2569716 | 0.603(-1.770-2.976) | 0.619 | 0.950 |
|  | rs3745871 | -1.716(-3.947-0.515) | 0.133 | 0.473 |
|  | rs11540761 | -2.052(-4.984-0.880) | 0.172 | 0.473 |
|  | rs11574576 | -1.437(-3.809-0.935) | 0.237 | 0.521 |
|  | rs28366008 | -0.387(-3.039-2.265) | 0.775 | 0.950 |
| HVLT | rs731170 | 1.905(-0.008-3.818) | 0.052 | 0.571 |
|  | rs1048801 | 0.009(-1.795-1.813) | 0.992 | 0.992 |
|  | rs1749316 | -0.872(-2.799-1.054) | 0.376 | 0.646 |
|  | rs1749317 | 0.176(-1.699-2.050) | 0.854 | 0.939 |
|  | rs1925241 | -0.863(-2.594-0.868) | 0.329 | 0.646 |
|  | rs2569715 | -0.286(-2.113-1.541) | 0.750 | 0.917 |
|  | rs2569716 | -0.494(-2.251-1.263) | 0.597 | 0.821 |
|  | rs3745871 | -1.122(-2.908-0.663) | 0.219 | 0.646 |
|  | rs11540761 | -0.932(-3.151-1.288) | 0.411 | 0.646 |
|  | rs11574576 | -1.375(-3.217-0.467) | 0.144 | 0.646 |
|  | rs28366008 | 1.180(-0.904-3.264) | 0.268 | 0.646 |
| LNS | rs731170 | 0.177(-0.345-0.699) | 0.507 | 0.835 |
|  | rs1048801 | 0.147(-0.345-0.640) | 0.558 | 0.835 |
|  | rs1749316 | -0.297(-0.822-0.228) | 0.268 | 0.835 |
|  | rs1749317 | 0.181(-0.334-0.696) | 0.491 | 0.835 |
|  | rs1925241 | 0.054(-0.418-0.527) | 0.821 | 0.869 |
|  | rs2569715 | -0.338(-0.817-0.140) | 0.167 | 0.835 |
|  | rs2569716 | -0.239(-0.736-0.258) | 0.346 | 0.835 |
|  | rs3745871 | -0.041(-0.529-0.447) | 0.869 | 0.869 |
|  | rs11540761 | 0.126(-0.481-0.733) | 0.683 | 0.835 |
|  | rs11574576 | -0.206(-0.707-0.295) | 0.421 | 0.835 |
|  | rs28366008 | 0.127(-0.443-0.696) | 0.663 | 0.835 |
| LXFLUEA | rs731170 | -0.154(-1.163-0.855) | 0.766 | 0.917 |
|  | rs1048801 | -0.434(-1.399-0.531) | 0.380 | 0.696 |
|  | rs1749316 | 0.795(-0.196-1.786) | 0.118 | 0.324 |
|  | rs1749317 | -0.022(-1.069-1.026) | 0.968 | 0.968 |
|  | rs1925241 | -0.830(-1.734-0.073) | 0.073 | 0.269 |
|  | rs2569715 | 0.364(-0.584-1.312) | 0.453 | 0.712 |
|  | rs2569716 | -1.093(-2.057--0.129) | **0.028** | 0.269 |
|  | rs3745871 | -0.868(-1.782-0.047) | 0.065 | 0.269 |
|  | rs11540761 | 0.132(-1.099-1.363) | 0.834 | 0.917 |
|  | rs11574576 | -0.588(-1.568-0.393) | 0.242 | 0.532 |
|  | rs28366008 | 0.145(-0.960-1.25) | 0.797 | 0.917 |
| LXFLUEF | rs731170 | -0.231(-1.325-0.862) | 0.679 | 0.779 |
|  | rs1048801 | -0.678(-1.725-0.369) | 0.206 | 0.665 |
|  | rs1749316 | 0.552(-0.527-1.630) | 0.318 | 0.665 |
|  | rs1749317 | 0.162(-0.964-1.287) | 0.779 | 0.779 |
|  | rs1925241 | -0.562(-1.545-0.421) | 0.264 | 0.665 |
|  | rs2569715 | 0.391(-0.637-1.420) | 0.457 | 0.665 |
|  | rs2569716 | -0.426(-1.485-0.633) | 0.431 | 0.665 |
|  | rs3745871 | -0.413(-1.404-0.579) | 0.416 | 0.665 |
|  | rs11540761 | 0.199(-1.133-1.531) | 0.770 | 0.779 |
|  | rs11574576 | -0.837(-1.889-0.215) | 0.121 | 0.665 |
|  | rs28366008 | 0.428(-0.767-1.624) | 0.483 | 0.665 |
| LXFLUES | rs731170 | 0.212(-0.99-1.414) | 0.730 | 0.873 |
|  | rs1048801 | -0.745(-1.892-0.402) | 0.204 | 0.374 |
|  | rs1749316 | 0.908(-0.273-2.09) | 0.134 | 0.369 |
|  | rs1749317 | -0.031(-1.279-1.217) | 0.961 | 0.961 |
|  | rs1925241 | -1.273(-2.343--0.203) | **0.021** | 0.114 |
|  | rs2569715 | 0.253(-0.877-1.384) | 0.661 | 0.873 |
|  | rs2569716 | -1.138(-2.290-0.014) | 0.054 | 0.199 |
|  | rs3745871 | -1.369(-2.451--0.287) | **0.014** | 0.114 |
|  | rs11540761 | 0.236(-1.231-1.703) | 0.753 | 0.873 |
|  | rs11574576 | -0.792(-1.959-0.375) | 0.185 | 0.374 |
|  | rs28366008 | -0.176(-1.492-1.140) | 0.794 | 0.873 |
| MoCA | rs731170 | 0.590(0.094-1.086) | **0.020** | 0.112 |
|  | rs1048801 | 0.264(-0.205-0.732) | 0.270 | 0.425 |
|  | rs1749316 | -0.049(-0.553-0.455) | 0.848 | 0.933 |
|  | rs1749317 | 0.005(-0.484-0.495) | 0.983 | 0.983 |
|  | rs1925241 | -0.557(-1.005--0.109) | **0.015** | 0.112 |
|  | rs2569715 | -0.122(-0.581-0.337) | 0.602 | 0.736 |
|  | rs2569716 | 0.137(-0.340-0.615) | 0.573 | 0.736 |
|  | rs3745871 | -0.493(-0.957--0.029) | **0.038** | 0.140 |
|  | rs11540761 | -0.521(-1.099-0.057) | 0.078 | 0.205 |
|  | rs11574576 | -0.396(-0.875-0.083) | 0.106 | 0.205 |
|  | rs28366008 | 0.442(-0.102-0.986) | 0.112 | 0.205 |
| SFT | rs731170 | 1.361(-0.473-3.196) | 0.147 | 0.323 |
|  | rs1048801 | -0.343(-2.072-1.385) | 0.697 | 0.697 |
|  | rs1749316 | 1.507(-0.341-3.356) | 0.111 | 0.305 |
|  | rs1749317 | 0.437(-1.366-2.241) | 0.635 | 0.697 |
|  | rs1925241 | -1.630(-3.287-0.027) | 0.055 | 0.201 |
|  | rs2569715 | 0.521(-1.169-2.210) | 0.546 | 0.667 |
|  | rs2569716 | -0.642(-2.392-1.108) | 0.473 | 0.650 |
|  | rs3745871 | -2.401(-4.110--0.710) | **0.006** | **0.032** |
|  | rs11540761 | -1.261(-3.396-0.873) | 0.248 | 0.454 |
|  | rs11574576 | -2.508(-4.255--0.761) | **0.005** | **0.032** |
|  | rs28366008 | 0.792(-1.216-2.800) | 0.440 | 0.650 |

CI, confidence internal; FDR, false discovery rate; ADL, Modified Schwab & England Activities of Daily Living Test; BJLOT, Benton Judgement of Line Orientation; BNT, Modified Boston Naming Test; FDR, false discovery rate; HVLT, Hopkins Verbal Learning Test; LNS, Letter-Number Sequencing Test; LXFLUEA, Lexical Fluency-A; LXFLUEF, Lexical Fluency-F; LXFLUES, Lexical Fluency-S; MoCA, Montreal Cognitive Assessment; SFT, semantic fluency test.
