# Supplementary material for: Uncovering the Associations of LILRB4 Genotypes With Parkinson's Disease: From Clinical Traits to Potential Pathologies
Source: CNS Neurosci Ther. 2025 Jul 23;31(7):e70522. doi: 10.1111/cns.70522 (PMC12287542; doi:10.1111/cns.70522)
Supplement: Supplementary file 4 — Table S1. [file CNS-31-e70522-s002.zip › cns70522-sup-0015-TableS32-S34@Supplementary Table 32-34 Model 2_The correlation between LILRB4 loci and scales.docx]

**Supplementary Table 32**. Model 2: The correlation between *LILRB4* loci and scales.

| Items | SNP | β(95%CI) | P value | FDR-corrected. P |
| --- | --- | --- | --- | --- |
| MDS-UPDRS Part I (P) | rs731170 | -0.320(-0.683-0.043) | 0.084 | 0.464 |
|  | rs1048801 | 0.093(-0.244-0.431) | 0.587 | 0.883 |
|  | rs1749316 | -0.100(-0.478-0.278) | 0.604 | 0.883 |
|  | rs1749317 | 0.170(-0.193-0.532) | 0.359 | 0.883 |
|  | rs1925241 | 0.177(-0.155-0.510) | 0.296 | 0.883 |
|  | rs2569715 | -0.122(-0.470-0.226) | 0.493 | 0.883 |
|  | rs2569716 | 0.082(-0.265-0.430) | 0.643 | 0.883 |
|  | rs3745871 | 0.395(0.056-0.735) | **0.023** | 0.251 |
|  | rs11540761 | 0.031(-0.380-0.442) | 0.883 | 0.883 |
|  | rs11574576 | -0.048(-0.402-0.306) | 0.791 | 0.883 |
|  | rs28366008 | 0.031(-0.372-0.434) | 0.881 | 0.883 |
| MDS-UPDRS Part I (R) | rs731170 | -0.225(-0.416--0.034) | **0.021** | 0.231 |
|  | rs1048801 | -0.022(-0.200-0.156) | 0.809 | 0.89 |
|  | rs1749316 | 0.135(-0.064-0.334) | 0.184 | 0.656 |
|  | rs1749317 | -0.072(-0.263-0.119) | 0.462 | 0.761 |
|  | rs1925241 | 0.011(-0.164-0.187) | 0.899 | 0.899 |
|  | rs2569715 | -0.028(-0.212-0.155) | 0.763 | 0.89 |
|  | rs2569716 | -0.117(-0.299-0.066) | 0.211 | 0.656 |
|  | rs3745871 | 0.108(-0.071-0.287) | 0.239 | 0.656 |
|  | rs11540761 | -0.094(-0.311-0.122) | 0.395 | 0.761 |
|  | rs11574576 | 0.056(-0.130-0.243) | 0.553 | 0.761 |
|  | rs28366008 | 0.071(-0.141-0.284) | 0.509 | 0.761 |
| MDS-UPDRS Part II | rs731170 | -0.189(-0.701-0.324) | 0.471 | 0.992 |
|  | rs1048801 | 0.003(-0.473-0.478) | 0.992 | 0.992 |
|  | rs1749316 | 0.029(-0.504-0.562) | 0.915 | 0.992 |
|  | rs1749317 | -0.233(-0.745-0.280) | 0.374 | 0.992 |
|  | rs1925241 | 0.038(-0.431-0.507) | 0.875 | 0.992 |
|  | rs2569715 | -0.118(-0.609-0.372) | 0.636 | 0.992 |
|  | rs2569716 | -0.078(-0.568-0.412) | 0.755 | 0.992 |
|  | rs3745871 | 0.232(-0.248-0.713) | 0.344 | 0.992 |
|  | rs11540761 | 0.198(-0.381-0.778) | 0.503 | 0.992 |
|  | rs11574576 | 0.012(-0.489-0.512) | 0.963 | 0.992 |
|  | rs28366008 | -0.058(-0.626-0.511) | 0.842 | 0.992 |
| MDS-UPDRS Part III | rs731170 | -0.772(-2.014-0.47) | 0.224 | 0.9 |
|  | rs1048801 | -0.335(-1.491-0.822) | 0.571 | 0.931 |
|  | rs1749316 | 0.057(-1.235-1.348) | 0.931 | 0.931 |
|  | rs1749317 | -0.079(-1.319-1.162) | 0.901 | 0.931 |
|  | rs1925241 | 0.126(-1.011-1.264) | 0.828 | 0.931 |
|  | rs2569715 | 0.168(-1.023-1.358) | 0.783 | 0.931 |
|  | rs2569716 | 0.344(-0.844-1.532) | 0.571 | 0.931 |
|  | rs3745871 | 0.905(-0.258-2.068) | 0.128 | 0.9 |
|  | rs11540761 | 0.833(-0.571-2.238) | 0.245 | 0.9 |
|  | rs11574576 | 0.214(-0.999-1.427) | 0.73 | 0.931 |
|  | rs28366008 | -0.504(-1.882-0.873) | 0.473 | 0.931 |
| MDS-UPDRS Part IV | rs731170 | -0.514(-0.897--0.132) | **0.009** | **0.024** |
|  | rs1048801 | 0.483(0.124-0.843) | **0.009** | **0.024** |
|  | rs1749316 | 0.202(-0.197-0.600) | 0.321 | 0.442 |
|  | rs1749317 | 0.581(0.193-0.969) | **0.003** | **0.024** |
|  | rs1925241 | 0.230(-0.112-0.572) | 0.187 | 0.338 |
|  | rs2569715 | -0.054(-0.418-0.309) | 0.769 | 0.846 |
|  | rs2569716 | 0.075(-0.297-0.447) | 0.693 | 0.846 |
|  | rs3745871 | 0.293(-0.058-0.644) | 0.102 | 0.224 |
|  | rs11540761 | 0.270(-0.156-0.696) | 0.215 | 0.338 |
|  | rs11574576 | 0.023(-0.341-0.388) | 0.901 | 0.901 |
|  | rs28366008 | -0.574(-1.000--0.147) | **0.009** | **0.024** |
| ADL | rs731170 | 0.274(-0.606-1.153) | 0.542 | 0.746 |
|  | rs1048801 | -0.163(-0.98-0.654) | 0.695 | 0.85 |
|  | rs1749316 | 0.473(-0.436-1.381) | 0.308 | 0.7 |
|  | rs1749317 | -0.016(-0.911-0.88) | 0.972 | 0.972 |
|  | rs1925241 | -0.353(-1.146-0.439) | 0.382 | 0.7 |
|  | rs2569715 | -0.077(-0.915-0.762) | 0.858 | 0.944 |
|  | rs2569716 | 0.385(-0.456-1.226) | 0.369 | 0.7 |
|  | rs3745871 | -0.695(-1.502-0.112) | 0.092 | 0.7 |
|  | rs11540761 | -0.420(-1.409-0.569) | 0.406 | 0.7 |
|  | rs11574576 | -0.329(-1.174-0.516) | 0.446 | 0.7 |
|  | rs28366008 | 0.573(-0.397-1.544) | 0.247 | 0.7 |
| BJLOT | rs731170 | 0.138(-0.344-0.620) | 0.575 | 0.791 |
|  | rs1048801 | 0.250(-0.198-0.697) | 0.274 | 0.627 |
|  | rs1749316 | 0.018(-0.481-0.518) | 0.942 | 0.942 |
|  | rs1749317 | -0.065(-0.546-0.416) | 0.791 | 0.87 |
|  | rs1925241 | -0.251(-0.691-0.190) | 0.265 | 0.627 |
|  | rs2569715 | -0.252(-0.713-0.209) | 0.285 | 0.627 |
|  | rs2569716 | 0.188(-0.272-0.648) | 0.423 | 0.664 |
|  | rs3745871 | -0.314(-0.766-0.137) | 0.173 | 0.627 |
|  | rs11540761 | -0.104(-0.647-0.440) | 0.709 | 0.867 |
|  | rs11574576 | -0.194(-0.664-0.277) | 0.42 | 0.664 |
|  | rs28366008 | 0.512(-0.019-1.044) | 0.059 | 0.627 |
| BNT | rs731170 | 0.983(-0.252-2.218) | 0.119 | 0.328 |
|  | rs1048801 | 0.550(-0.610-1.711) | 0.353 | 0.615 |
|  | rs1749316 | 0.555(-0.713-1.823) | 0.391 | 0.615 |
|  | rs1749317 | -0.245(-1.525-1.035) | 0.708 | 0.779 |
|  | rs1925241 | -1.226(-2.334--0.119) | **0.031** | 0.112 |
|  | rs2569715 | -0.466(-1.665-0.734) | 0.447 | 0.615 |
|  | rs2569716 | 0.267(-0.920-1.454) | 0.659 | 0.779 |
|  | rs3745871 | -1.324(-2.453--0.194) | **0.022** | 0.112 |
|  | rs11540761 | -1.803(-3.222--0.383) | **0.013** | 0.112 |
|  | rs11574576 | -0.755(-1.980-0.471) | 0.228 | 0.502 |
|  | rs28366008 | 0.079(-1.264-1.423) | 0.908 | 0.908 |
| HVLT | rs731170 | 0.906(-0.256-2.068) | 0.127 | 0.465 |
|  | rs1048801 | 0.368(-0.711-1.448) | 0.504 | 0.606 |
|  | rs1749316 | -0.471(-1.676-0.734) | 0.444 | 0.606 |
|  | rs1749317 | -0.526(-1.686-0.635) | 0.375 | 0.606 |
|  | rs1925241 | -0.190(-1.253-0.872) | 0.726 | 0.726 |
|  | rs2569715 | 0.338(-0.773-1.449) | 0.551 | 0.606 |
|  | rs2569716 | 1.095(-0.013-2.203) | 0.053 | 0.292 |
|  | rs3745871 | -0.428(-1.516-0.660) | 0.441 | 0.606 |
|  | rs11540761 | -0.505(-1.817-0.806) | 0.45 | 0.606 |
|  | rs11574576 | -1.197(-2.326--0.067) | **0.038** | 0.292 |
|  | rs28366008 | 0.557(-0.729-1.842) | 0.396 | 0.606 |
| LNS | rs731170 | -0.049(-0.359-0.262) | 0.759 | 0.835 |
|  | rs1048801 | 0.204(-0.084-0.493) | 0.165 | 0.605 |
|  | rs1749316 | -0.153(-0.475-0.169) | 0.353 | 0.617 |
|  | rs1749317 | 0.120(-0.191-0.431) | 0.449 | 0.617 |
|  | rs1925241 | 0.247(-0.036-0.53) | 0.088 | 0.483 |
|  | rs2569715 | -0.129(-0.426-0.168) | 0.395 | 0.617 |
|  | rs2569716 | 0.180(-0.116-0.477) | 0.233 | 0.617 |
|  | rs3745871 | 0.116(-0.175-0.407) | 0.434 | 0.617 |
|  | rs11540761 | 0.326(-0.025-0.676) | 0.069 | 0.483 |
|  | rs11574576 | -0.017(-0.319-0.285) | 0.914 | 0.914 |
|  | rs28366008 | 0.081(-0.263-0.425) | 0.643 | 0.786 |
| LXFLUEA | rs731170 | -0.355(-1.007-0.296) | 0.285 | 0.923 |
|  | rs1048801 | -0.041(-0.659-0.578) | 0.898 | 0.923 |
|  | rs1749316 | 0.434(-0.237-1.105) | 0.206 | 0.923 |
|  | rs1749317 | 0.033(-0.647-0.714) | 0.923 | 0.923 |
|  | rs1925241 | -0.122(-0.711-0.467) | 0.685 | 0.923 |
|  | rs2569715 | 0.113(-0.520-0.745) | 0.727 | 0.923 |
|  | rs2569716 | -0.232(-0.864-0.400) | 0.473 | 0.923 |
|  | rs3745871 | -0.14(-0.744-0.464) | 0.65 | 0.923 |
|  | rs11540761 | -0.087(-0.842-0.669) | 0.822 | 0.923 |
|  | rs11574576 | -0.717(-1.36--0.074) | **0.029** | 0.322 |
|  | rs28366008 | -0.065(-0.782-0.651) | 0.858 | 0.923 |
| LXFLUEF | rs731170 | 0.203(-0.508-0.914) | 0.575 | 0.939 |
|  | rs1048801 | -0.354(-1.029-0.321) | 0.305 | 0.939 |
|  | rs1749316 | -0.078(-0.811-0.655) | 0.835 | 0.939 |
|  | rs1749317 | 0.083(-0.657-0.822) | 0.826 | 0.939 |
|  | rs1925241 | -0.100(-0.742-0.542) | 0.761 | 0.939 |
|  | rs2569715 | 0.171(-0.519-0.861) | 0.628 | 0.939 |
|  | rs2569716 | 0.158(-0.532-0.848) | 0.654 | 0.939 |
|  | rs3745871 | -0.254(-0.911-0.403) | 0.449 | 0.939 |
|  | rs11540761 | -0.032(-0.856-0.791) | 0.939 | 0.939 |
|  | rs11574576 | -0.919(-1.617--0.222) | **0.01** | 0.111 |
|  | rs28366008 | 0.047(-0.734-0.829) | 0.905 | 0.939 |
| LXFLUES | rs731170 | -0.216(-0.950-0.519) | 0.565 | 0.754 |
|  | rs1048801 | -0.252(-0.949-0.445) | 0.478 | 0.754 |
|  | rs1749316 | 0.484(-0.276-1.243) | 0.213 | 0.754 |
|  | rs1749317 | -0.168(-0.935-0.599) | 0.667 | 0.754 |
|  | rs1925241 | -0.210(-0.874-0.454) | 0.535 | 0.754 |
|  | rs2569715 | -0.147(-0.86-0.565) | 0.686 | 0.754 |
|  | rs2569716 | -0.640(-1.350-0.070) | 0.078 | 0.428 |
|  | rs3745871 | -0.336(-1.017-0.344) | 0.333 | 0.754 |
|  | rs11540761 | 0.103(-0.748-0.954) | 0.812 | 0.812 |
|  | rs11574576 | -0.739(-1.464--0.013) | **0.046** | 0.428 |
|  | rs28366008 | -0.189(-0.997-0.618) | 0.646 | 0.754 |
| MoCA | rs731170 | 0.198(-0.080-0.476) | 0.163 | 0.321 |
|  | rs1048801 | 0.246(-0.011-0.504) | 0.061 | 0.321 |
|  | rs1749316 | 0.032(-0.257-0.321) | 0.83 | 0.83 |
|  | rs1749317 | -0.043(-0.321-0.234) | 0.76 | 0.83 |
|  | rs1925241 | -0.142(-0.397-0.112) | 0.273 | 0.429 |
|  | rs2569715 | 0.072(-0.194-0.338) | 0.596 | 0.782 |
|  | rs2569716 | 0.215(-0.051-0.481) | 0.113 | 0.321 |
|  | rs3745871 | -0.180(-0.441-0.080) | 0.175 | 0.321 |
|  | rs11540761 | -0.075(-0.390-0.239) | 0.64 | 0.782 |
|  | rs11574576 | -0.211(-0.481-0.06) | 0.128 | 0.321 |
|  | rs28366008 | 0.371(0.064-0.679) | **0.018** | 0.199 |
| Semantic Fluency | rs731170 | 0.172(-0.958-1.301) | 0.766 | 0.842 |
|  | rs1048801 | 0.505(-0.543-1.553) | 0.346 | 0.634 |
|  | rs1749316 | 1.026(-0.145-2.196) | 0.086 | 0.291 |
|  | rs1749317 | 0.405(-0.724-1.534) | 0.482 | 0.735 |
|  | rs1925241 | -0.629(-1.661-0.403) | 0.232 | 0.511 |
|  | rs2569715 | 0.206(-0.874-1.286) | 0.709 | 0.842 |
|  | rs2569716 | 0.015(-1.064-1.094) | 0.978 | 0.978 |
|  | rs3745871 | -0.873(-1.93-0.184) | 0.106 | 0.291 |
|  | rs11540761 | -0.404(-1.68-0.872) | 0.535 | 0.735 |
|  | rs11574576 | -2.107(-3.198--1.017) | **＜0.001** | **0.002** |
|  | rs28366008 | 1.156(-0.092-2.405) | 0.07 | 0.291 |

CI, confidence internal; FDR, false discovery rate; ADL, Modified Schwab & England Activities of Daily Living Test; BJLOT, Benton Judgement of Line Orientation; BNT, Modified Boston Naming Test; FDR, false discovery rate; HVLT, Hopkins Verbal Learning Test; LNS, Letter-Number Sequencing Test; LXFLUEA, Lexical Fluency-A; LXFLUEF, Lexical Fluency-F; LXFLUES, Lexical Fluency-S; MoCA, Montreal Cognitive Assessment; SFT, semantic fluency test.

**Supplementary Table 33**. Model 2: The correlation between *LILRB4* loci and scales in male.

| Items | SNP | β(95%CI) | P value | FDR-corrected. P |
| --- | --- | --- | --- | --- |
| MDS-UPDRS Part I (P) | rs731170 | -0.288(-0.72-0.143) | 0.191 | 0.924 |
|  | rs1048801 | -0.063(-0.463-0.337) | 0.758 | 0.924 |
|  | rs1749316 | 0.036(-0.424-0.496) | 0.878 | 0.924 |
|  | rs1749317 | 0.152(-0.287-0.591) | 0.498 | 0.924 |
|  | rs1925241 | 0.122(-0.277-0.52) | 0.55 | 0.924 |
|  | rs2569715 | -0.068(-0.495-0.359) | 0.754 | 0.924 |
|  | rs2569716 | -0.105(-0.519-0.309) | 0.618 | 0.924 |
|  | rs3745871 | 0.240(-0.166-0.645) | 0.247 | 0.924 |
|  | rs11540761 | 0.023(-0.458-0.505) | 0.924 | 0.924 |
|  | rs11574576 | 0.230(-0.196-0.657) | 0.29 | 0.924 |
|  | rs28366008 | -0.145(-0.631-0.341) | 0.559 | 0.924 |
| MDS-UPDRS Part I (R) | rs731170 | -0.206(-0.424-0.011) | 0.063 | 0.348 |
|  | rs1048801 | -0.146(-0.347-0.056) | 0.157 | 0.434 |
|  | rs1749316 | 0.108(-0.124-0.339) | 0.362 | 0.498 |
|  | rs1749317 | 0.039(-0.182-0.261) | 0.727 | 0.8 |
|  | rs1925241 | 0.112(-0.089-0.312) | 0.276 | 0.434 |
|  | rs2569715 | -0.078(-0.293-0.137) | 0.476 | 0.582 |
|  | rs2569716 | -0.335(-0.541--0.128) | **0.002** | **0.017** |
|  | rs3745871 | 0.123(-0.081-0.328) | 0.238 | 0.434 |
|  | rs11540761 | -0.137(-0.379-0.106) | 0.271 | 0.434 |
|  | rs11574576 | 0.121(-0.094-0.336) | 0.271 | 0.434 |
|  | rs28366008 | 0.024(-0.222-0.269) | 0.85 | 0.85 |
| MDS-UPDRS Part II | rs731170 | 0.015(-0.624-0.655) | 0.962 | 0.968 |
|  | rs1048801 | -0.061(-0.653-0.531) | 0.841 | 0.968 |
|  | rs1749316 | 0.014(-0.667-0.694) | 0.968 | 0.968 |
|  | rs1749317 | -0.066(-0.718-0.587) | 0.844 | 0.968 |
|  | rs1925241 | 0.026(-0.563-0.616) | 0.93 | 0.968 |
|  | rs2569715 | -0.378(-1.008-0.253) | 0.241 | 0.968 |
|  | rs2569716 | -0.440(-1.052-0.172) | 0.159 | 0.968 |
|  | rs3745871 | 0.075(-0.526-0.675) | 0.808 | 0.968 |
|  | rs11540761 | -0.039(-0.753-0.674) | 0.914 | 0.968 |
|  | rs11574576 | 0.259(-0.372-0.890) | 0.422 | 0.968 |
|  | rs28366008 | -0.096(-0.816-0.623) | 0.793 | 0.968 |
| MDS-UPDRS Part III | rs731170 | -0.616(-2.238-1.007) | 0.457 | 0.819 |
|  | rs1048801 | -0.370(-1.876-1.136) | 0.63 | 0.819 |
|  | rs1749316 | 0.375(-1.350-2.101) | 0.67 | 0.819 |
|  | rs1749317 | 0.254(-1.393-1.901) | 0.763 | 0.823 |
|  | rs1925241 | -0.459(-1.953-1.035) | 0.548 | 0.819 |
|  | rs2569715 | -0.774(-2.375-0.827) | 0.344 | 0.819 |
|  | rs2569716 | -0.622(-2.175-0.931) | 0.433 | 0.819 |
|  | rs3745871 | 0.423(-1.099-1.946) | 0.586 | 0.819 |
|  | rs11540761 | -0.207(-2.015-1.602) | 0.823 | 0.823 |
|  | rs11574576 | 0.515(-1.090-2.121) | 0.53 | 0.819 |
|  | rs28366008 | -0.702(-2.526-1.121) | 0.451 | 0.819 |
| MDS-UPDRS Part IV | rs731170 | -0.306(-0.807-0.195) | 0.233 | 0.512 |
|  | rs1048801 | 0.597(0.125-1.069) | **0.014** | 0.15 |
|  | rs1749316 | 0.135(-0.399-0.67) | 0.62 | 0.734 |
|  | rs1749317 | 0.540(0.036-1.045) | **0.037** | 0.201 |
|  | rs1925241 | 0.124(-0.332-0.580) | 0.594 | 0.734 |
|  | rs2569715 | 0.055(-0.441-0.552) | 0.827 | 0.827 |
|  | rs2569716 | 0.250(-0.243-0.742) | 0.321 | 0.56 |
|  | rs3745871 | 0.219(-0.245-0.683) | 0.356 | 0.56 |
|  | rs11540761 | 0.440(-0.115-0.995) | 0.121 | 0.334 |
|  | rs11574576 | 0.107(-0.378-0.591) | 0.667 | 0.734 |
|  | rs28366008 | -0.513(-1.082-0.055) | 0.078 | 0.285 |
| ADL | rs731170 | -0.528(-1.599-0.543) | 0.335 | 0.949 |
|  | rs1048801 | -0.067(-1.056-0.922) | 0.895 | 0.949 |
|  | rs1749316 | 0.417(-0.712-1.547) | 0.469 | 0.949 |
|  | rs1749317 | -0.145(-1.243-0.952) | 0.795 | 0.949 |
|  | rs1925241 | 0.277(-0.696-1.251) | 0.577 | 0.949 |
|  | rs2569715 | 0.065(-0.990-1.119) | 0.905 | 0.949 |
|  | rs2569716 | 0.734(-0.284-1.752) | 0.158 | 0.871 |
|  | rs3745871 | 0.192(-0.793-1.178) | 0.702 | 0.949 |
|  | rs11540761 | 0.912(-0.276-2.100) | 0.133 | 0.871 |
|  | rs11574576 | -0.034(-1.066-0.999) | 0.949 | 0.949 |
|  | rs28366008 | 0.505(-0.686-1.696) | 0.406 | 0.949 |
| BJLOT | rs731170 | -0.401(-0.951-0.149) | 0.154 | 0.579 |
|  | rs1048801 | 0.467(-0.041-0.975) | 0.072 | 0.579 |
|  | rs1749316 | 0.099(-0.487-0.685) | 0.741 | 0.892 |
|  | rs1749317 | -0.039(-0.601-0.523) | 0.892 | 0.892 |
|  | rs1925241 | 0.224(-0.284-0.732) | 0.388 | 0.61 |
|  | rs2569715 | -0.246(-0.789-0.296) | 0.374 | 0.61 |
|  | rs2569716 | 0.056(-0.472-0.584) | 0.835 | 0.892 |
|  | rs3745871 | -0.043(-0.562-0.476) | 0.872 | 0.892 |
|  | rs11540761 | 0.361(-0.253-0.974) | 0.25 | 0.61 |
|  | rs11574576 | 0.281(-0.264-0.827) | 0.313 | 0.61 |
|  | rs28366008 | 0.446(-0.172-1.064) | 0.158 | 0.579 |
| BNT | rs731170 | 0.743(-0.583-2.069) | 0.273 | 0.57 |
|  | rs1048801 | 0.782(-0.461-2.025) | 0.219 | 0.57 |
|  | rs1749316 | 0.309(-1.101-1.720) | 0.668 | 0.735 |
|  | rs1749317 | -0.539(-1.915-0.838) | 0.444 | 0.634 |
|  | rs1925241 | -0.695(-1.893-0.503) | 0.257 | 0.57 |
|  | rs2569715 | -0.683(-2.002-0.635) | 0.311 | 0.57 |
|  | rs2569716 | 0.300(-0.968-1.568) | 0.643 | 0.735 |
|  | rs3745871 | -1.127(-2.354-0.100) | 0.073 | 0.401 |
|  | rs11540761 | -1.616(-3.11--0.123) | **0.035** | 0.383 |
|  | rs11574576 | -0.227(-1.571-1.117) | 0.741 | 0.741 |
|  | rs28366008 | 0.549(-0.908-2.006) | 0.461 | 0.634 |
| HVLT | rs731170 | 0.381(-1.072-1.834) | 0.608 | 0.88 |
|  | rs1048801 | 0.562(-0.782-1.906) | 0.413 | 0.88 |
|  | rs1749316 | -0.321(-1.866-1.223) | 0.684 | 0.88 |
|  | rs1749317 | -0.931(-2.410-0.548) | 0.218 | 0.799 |
|  | rs1925241 | 0.235(-1.104-1.573) | 0.731 | 0.88 |
|  | rs2569715 | 0.685(-0.747-2.117) | 0.349 | 0.88 |
|  | rs2569716 | 2.113(0.735-3.490) | **0.003** | **0.031** |
|  | rs3745871 | -0.003(-1.369-1.362) | 0.996 | 0.996 |
|  | rs11540761 | -0.209(-1.828-1.410) | 0.8 | 0.88 |
|  | rs11574576 | -0.961(-2.392-0.470) | 0.189 | 0.799 |
|  | rs28366008 | 0.227(-1.406-1.861) | 0.785 | 0.88 |
| LNS | rs731170 | -0.172(-0.557-0.214) | 0.383 | 0.702 |
|  | rs1048801 | 0.225(-0.132-0.581) | 0.218 | 0.585 |
|  | rs1749316 | -0.073(-0.483-0.337) | 0.727 | 0.838 |
|  | rs1749317 | 0.075(-0.318-0.468) | 0.709 | 0.838 |
|  | rs1925241 | 0.361(0.007-0.715) | **0.046** | 0.17 |
|  | rs2569715 | 0.006(-0.374-0.387) | 0.975 | 0.975 |
|  | rs2569716 | 0.446(0.079-0.813) | **0.018** | 0.17 |
|  | rs3745871 | 0.206(-0.156-0.568) | 0.266 | 0.585 |
|  | rs11540761 | 0.450(0.022-0.878) | **0.04** | 0.17 |
|  | rs11574576 | 0.111(-0.269-0.491) | 0.568 | 0.838 |
|  | rs28366008 | 0.067(-0.367-0.501) | 0.762 | 0.838 |
| LXFLUEA | rs731170 | -0.475(-1.320-0.370) | 0.272 | 0.811 |
|  | rs1048801 | 0.184(-0.617-0.984) | 0.654 | 0.811 |
|  | rs1749316 | 0.173(-0.730-1.076) | 0.708 | 0.811 |
|  | rs1749317 | 0.151(-0.731-1.034) | 0.737 | 0.811 |
|  | rs1925241 | 0.331(-0.436-1.099) | 0.398 | 0.811 |
|  | rs2569715 | -0.038(-0.878-0.801) | 0.929 | 0.929 |
|  | rs2569716 | 0.231(-0.590-1.051) | 0.582 | 0.811 |
|  | rs3745871 | 0.329(-0.464-1.122) | 0.417 | 0.811 |
|  | rs11540761 | -0.216(-1.173-0.740) | 0.658 | 0.811 |
|  | rs11574576 | -0.805(-1.652-0.042) | 0.064 | 0.699 |
|  | rs28366008 | -0.210(-1.150-0.729) | 0.661 | 0.811 |
| LXFLUEF | rs731170 | 0.473(-0.452-1.398) | 0.317 | 0.872 |
|  | rs1048801 | -0.157(-1.033-0.719) | 0.726 | 0.917 |
|  | rs1749316 | -0.566(-1.552-0.419) | 0.261 | 0.872 |
|  | rs1749317 | 0.103(-0.862-1.069) | 0.834 | 0.917 |
|  | rs1925241 | 0.204(-0.636-1.044) | 0.635 | 0.917 |
|  | rs2569715 | -0.014(-0.933-0.904) | 0.976 | 0.976 |
|  | rs2569716 | 0.493(-0.403-1.389) | 0.282 | 0.872 |
|  | rs3745871 | -0.138(-1.007-0.731) | 0.756 | 0.917 |
|  | rs11540761 | -0.155(-1.201-0.891) | 0.772 | 0.917 |
|  | rs11574576 | -0.978(-1.903--0.053) | **0.039** | 0.431 |
|  | rs28366008 | -0.178(-1.206-0.850) | 0.735 | 0.917 |
| LXFLUES | rs731170 | -0.474(-1.395-0.448) | 0.315 | 0.872 |
|  | rs1048801 | 0.022(-0.851-0.896) | 0.96 | 0.96 |
|  | rs1749316 | 0.186(-0.806-1.177) | 0.714 | 0.872 |
|  | rs1749317 | -0.217(-1.179-0.745) | 0.659 | 0.872 |
|  | rs1925241 | 0.423(-0.414-1.26) | 0.323 | 0.872 |
|  | rs2569715 | -0.424(-1.337-0.489) | 0.363 | 0.872 |
|  | rs2569716 | -0.338(-1.231-0.554) | 0.458 | 0.872 |
|  | rs3745871 | 0.278(-0.588-1.144) | 0.53 | 0.872 |
|  | rs11540761 | 0.054(-0.988-1.096) | 0.919 | 0.96 |
|  | rs11574576 | -0.745(-1.671-0.180) | 0.116 | 0.872 |
|  | rs28366008 | -0.244(-1.268-0.780) | 0.641 | 0.872 |
| MoCA | rs731170 | -0.002(-0.338-0.334) | 0.991 | 0.991 |
|  | rs1048801 | 0.220(-0.090-0.530) | 0.166 | 0.607 |
|  | rs1749316 | 0.070(-0.287-0.427) | 0.701 | 0.916 |
|  | rs1749317 | -0.101(-0.442-0.24) | 0.561 | 0.916 |
|  | rs1925241 | 0.081(-0.228-0.391) | 0.607 | 0.916 |
|  | rs2569715 | 0.199(-0.131-0.53) | 0.238 | 0.654 |
|  | rs2569716 | 0.306(-0.014-0.626) | 0.062 | 0.401 |
|  | rs3745871 | -0.024(-0.339-0.291) | 0.881 | 0.969 |
|  | rs11540761 | 0.183(-0.191-0.556) | 0.339 | 0.745 |
|  | rs11574576 | -0.054(-0.385-0.277) | 0.749 | 0.916 |
|  | rs28366008 | 0.345(-0.031-0.721) | 0.073 | 0.401 |
| Semantic Fluency | rs731170 | -0.481(-1.904-0.941) | 0.508 | 0.995 |
|  | rs1048801 | 0.919(-0.395-2.234) | 0.171 | 0.627 |
|  | rs1749316 | 0.618(-0.893-2.130) | 0.423 | 0.995 |
|  | rs1749317 | 0.252(-1.199-1.702) | 0.734 | 0.995 |
|  | rs1925241 | -0.052(-1.362-1.259) | 0.938 | 0.995 |
|  | rs2569715 | -0.005(-1.408-1.399) | 0.995 | 0.995 |
|  | rs2569716 | 0.408(-0.953-1.769) | 0.557 | 0.995 |
|  | rs3745871 | 0.059(-1.278-1.396) | 0.931 | 0.995 |
|  | rs11540761 | 0.106(-1.480-1.691) | 0.896 | 0.995 |
|  | rs11574576 | -1.942(-3.336--0.548) | **0.007** | 0.072 |
|  | rs28366008 | 1.546(-0.048-3.139) | 0.058 | 0.318 |

CI, confidence internal; FDR, false discovery rate; ADL, Modified Schwab & England Activities of Daily Living Test; BJLOT, Benton Judgement of Line Orientation; BNT, Modified Boston Naming Test; FDR, false discovery rate; HVLT, Hopkins Verbal Learning Test; LNS, Letter-Number Sequencing Test; LXFLUEA, Lexical Fluency-A; LXFLUEF, Lexical Fluency-F; LXFLUES, Lexical Fluency-S; MoCA, Montreal Cognitive Assessment; SFT, semantic fluency test.

**Supplementary Table 34**. Model 2: The correlation between *LILRB4* loci and scales in female.

| Items | SNP | β(95%CI) | P value | FDR-corrected. P |
| --- | --- | --- | --- | --- |
| MDS-UPDRS Part I (P) | rs731170 | -0.371(-1.016-0.274) | 0.260 | 0.552 |
|  | rs1048801 | 0.34(-0.264-0.944) | 0.270 | 0.552 |
|  | rs1749316 | -0.291(-0.939-0.358) | 0.380 | 0.552 |
|  | rs1749317 | 0.191(-0.44-0.823) | 0.554 | 0.609 |
|  | rs1925241 | 0.251(-0.334-0.835) | 0.401 | 0.552 |
|  | rs2569715 | -0.202(-0.794-0.39) | 0.505 | 0.609 |
|  | rs2569716 | 0.369(-0.247-0.985) | 0.241 | 0.552 |
|  | rs3745871 | 0.636(0.036-1.237) | **0.039** | 0.424 |
|  | rs11540761 | 0.038(-0.710-0.787) | 0.920 | 0.920 |
|  | rs11574576 | -0.490(-1.109-0.129) | 0.122 | 0.552 |
|  | rs28366008 | 0.307(-0.396-1.009) | 0.393 | 0.552 |
| MDS-UPDRS Part I (R) | rs731170 | -0.253(-0.608-0.103) | 0.165 | 0.617 |
|  | rs1048801 | 0.177(-0.157-0.510) | 0.300 | 0.659 |
|  | rs1749316 | 0.177(-0.181-0.535) | 0.332 | 0.659 |
|  | rs1749317 | -0.261(-0.608-0.087) | 0.143 | 0.617 |
|  | rs1925241 | -0.151(-0.474-0.172) | 0.359 | 0.659 |
|  | rs2569715 | 0.054(-0.273-0.381) | 0.746 | 0.821 |
|  | rs2569716 | 0.239(-0.100-0.579) | 0.168 | 0.617 |
|  | rs3745871 | 0.079(-0.254-0.413) | 0.642 | 0.821 |
|  | rs11540761 | -0.021(-0.434-0.392) | 0.921 | 0.921 |
|  | rs11574576 | -0.060(-0.403-0.283) | 0.732 | 0.821 |
|  | rs28366008 | 0.146(-0.241-0.534) | 0.460 | 0.723 |
| MDS-UPDRS Part II | rs731170 | -0.519(-1.376-0.338) | 0.236 | 0.587 |
|  | rs1048801 | 0.097(-0.706-0.900) | 0.813 | 0.957 |
|  | rs1749316 | 0.040(-0.823-0.902) | 0.928 | 0.957 |
|  | rs1749317 | -0.475(-1.312-0.362) | 0.267 | 0.587 |
|  | rs1925241 | 0.053(-0.726-0.832) | 0.894 | 0.957 |
|  | rs2569715 | 0.225(-0.562-1.011) | 0.576 | 0.905 |
|  | rs2569716 | 0.483(-0.336-1.301) | 0.249 | 0.587 |
|  | rs3745871 | 0.491(-0.311-1.293) | 0.231 | 0.587 |
|  | rs11540761 | 0.619(-0.374-1.613) | 0.223 | 0.587 |
|  | rs11574576 | -0.376(-1.204-0.453) | 0.375 | 0.687 |
|  | rs28366008 | 0.026(-0.909-0.961) | 0.957 | 0.957 |
| MDS-UPDRS Part III | rs731170 | -0.987(-2.920-0.946) | 0.318 | 0.582 |
|  | rs1048801 | -0.346(-2.158-1.467) | 0.709 | 0.780 |
|  | rs1749316 | -0.458(-2.402-1.487) | 0.645 | 0.780 |
|  | rs1749317 | -0.706(-2.597-1.185) | 0.465 | 0.731 |
|  | rs1925241 | 1.037(-0.712-2.787) | 0.246 | 0.541 |
|  | rs2569715 | 1.535(-0.231-3.301) | 0.090 | 0.246 |
|  | rs2569716 | 1.879(0.042-3.717) | **0.046** | 0.241 |
|  | rs3745871 | 1.696(-0.104-3.497) | 0.066 | 0.241 |
|  | rs11540761 | 2.678(0.456-4.900) | **0.019** | 0.207 |
|  | rs11574576 | -0.386(-2.247-1.475) | 0.684 | 0.780 |
|  | rs28366008 | -0.128(-2.234-1.978) | 0.905 | 0.905 |
| MDS-UPDRS Part IV | rs731170 | -0.861(-1.456--0.266) | **0.005** | 0.054 |
|  | rs1048801 | 0.313(-0.247-0.873) | 0.275 | 0.504 |
|  | rs1749316 | 0.289(-0.309-0.887) | 0.345 | 0.542 |
|  | rs1749317 | 0.654(0.042-1.265) | **0.037** | 0.193 |
|  | rs1925241 | 0.382(-0.136-0.900) | 0.150 | 0.329 |
|  | rs2569715 | -0.202(-0.734-0.330) | 0.458 | 0.616 |
|  | rs2569716 | -0.196(-0.769-0.377) | 0.504 | 0.616 |
|  | rs3745871 | 0.416(-0.122-0.954) | 0.131 | 0.329 |
|  | rs11540761 | 0.013(-0.652-0.678) | 0.969 | 0.969 |
|  | rs11574576 | -0.106(-0.662-0.45) | 0.708 | 0.779 |
|  | rs28366008 | -0.645(-1.294-0.004) | 0.053 | 0.193 |
| ADL | rs731170 | 1.442(-0.064-2.947) | 0.062 | 0.201 |
|  | rs1048801 | -0.28(-1.703-1.143) | 0.700 | 0.823 |
|  | rs1749316 | 0.561(-0.958-2.08) | 0.470 | 0.738 |
|  | rs1749317 | 0.251(-1.28-1.783) | 0.748 | 0.823 |
|  | rs1925241 | -1.231(-2.572-0.11) | 0.073 | 0.201 |
|  | rs2569715 | -0.291(-1.67-1.088) | 0.679 | 0.823 |
|  | rs2569716 | -0.132(-1.596-1.331) | 0.860 | 0.860 |
|  | rs3745871 | -2.007(-3.38--0.635) | **0.004** | **0.025** |
|  | rs11540761 | -2.550(-4.258--0.842) | **0.004** | **0.025** |
|  | rs11574576 | -0.698(-2.158-0.762) | 0.350 | 0.738 |
|  | rs28366008 | 0.632(-1.028-2.293) | 0.456 | 0.738 |
| BJLOT | rs731170 | 1.034(0.152-1.916) | **0.022** | 0.122 |
|  | rs1048801 | -0.113(-0.947-0.721) | 0.791 | 0.871 |
|  | rs1749316 | -0.162(-1.05-0.725) | 0.720 | 0.871 |
|  | rs1749317 | -0.025(-0.889-0.84c) | 0.956 | 0.956 |
|  | rs1925241 | -0.95c(-1.745--0.155) | **0.020** | 0.122 |
|  | rs2569715 | -0.32c(-1.134-0.495) | 0.443 | 0.609 |
|  | rs2569716 | 0.368(-0.478-1.214) | 0.395 | 0.609 |
|  | rs3745871 | -0.705(-1.529-0.12c) | 0.095 | 0.209 |
|  | rs11540761 | -0.884(-1.903-0.135) | 0.090 | 0.209 |
|  | rs11574576 | -0.892(-1.741--0.043) | **0.040** | 0.148 |
|  | rs28366008 | 0.666(-0.293-1.625) | 0.174 | 0.320 |
| BNT | rs731170 | 1.48c(-0.925-3.886) | 0.230 | 0.505 |
|  | rs1048801 | 0.228(-2.043-2.5cc) | 0.844 | 0.929 |
|  | rs1749316 | 0.655(-1.701-3.011) | 0.587 | 0.929 |
|  | rs1749317 | 0.575(-1.928-3.078) | 0.653 | 0.929 |
|  | rs1925241 | -2.057(-4.197-0.083) | 0.061 | 0.444 |
|  | rs2569715 | -0.416(-2.689-1.857) | 0.720 | 0.929 |
|  | rs2569716 | 0.072(-2.265-2.408) | 0.952 | 0.952 |
|  | rs3745871 | -1.565(-3.746-0.616) | 0.161 | 0.444 |
|  | rs11540761 | -2.269(-5.131-0.592) | 0.122 | 0.444 |
|  | rs11574576 | -1.832(-4.151-0.487) | 0.123 | 0.444 |
|  | rs28366008 | -0.404(-2.989-2.181) | 0.760 | 0.929 |
| HVLT | rs731170 | 1.798(-0.11c-3.706) | 0.066 | 0.615 |
|  | rs1048801 | 0.079(-1.714-1.872) | 0.931 | 0.931 |
|  | rs1749316 | -0.85(-2.764-1.064) | 0.385 | 0.650 |
|  | rs1749317 | 0.244(-1.62c-2.109) | 0.798 | 0.877 |
|  | rs1925241 | -0.721(-2.448-1.006) | 0.414 | 0.650 |
|  | rs2569715 | -0.241(-1.99-1.509) | 0.788 | 0.877 |
|  | rs2569716 | -0.602(-2.43-1.226) | 0.519 | 0.714 |
|  | rs3745871 | -0.987(-2.768-0.795) | 0.279 | 0.650 |
|  | rs11540761 | -0.947(-3.153-1.258) | 0.401 | 0.650 |
|  | rs11574576 | -1.494(-3.33c-0.342) | 0.112 | 0.615 |
|  | rs28366008 | 1.148(-0.922-3.219) | 0.278 | 0.650 |
| LNS | rs731170 | 0.157(-0.364-0.678) | 0.555 | 0.799 |
|  | rs1048801 | 0.169(-0.321-0.658) | 0.501 | 0.799 |
|  | rs1749316 | -0.288(-0.81c-0.234) | 0.281 | 0.799 |
|  | rs1749317 | 0.194(-0.319-0.707) | 0.459 | 0.799 |
|  | rs1925241 | 0.084(-0.387-0.556) | 0.727 | 0.799 |
|  | rs2569715 | -0.319(-0.795-0.158) | 0.192 | 0.799 |
|  | rs2569716 | -0.252(-0.749-0.246) | 0.323 | 0.799 |
|  | rs3745871 | -0.014(-0.501-0.473) | 0.956 | 0.956 |
|  | rs11540761 | 0.119(-0.485-0.723) | 0.700 | 0.799 |
|  | rs11574576 | -0.223(-0.723-0.278) | 0.384 | 0.799 |
|  | rs28366008 | 0.117(-0.45c-0.683) | 0.687 | 0.799 |
| LXFLUEA | rs731170 | -0.14(-1.158-0.879) | 0.789 | 0.889 |
|  | rs1048801 | -0.436(-1.408-0.535) | 0.380 | 0.697 |
|  | rs1749316 | 0.787(-0.209-1.782) | 0.123 | 0.339 |
|  | rs1749317 | -0.077(-1.152-0.999) | 0.889 | 0.889 |
|  | rs1925241 | -0.845(-1.753-0.064) | 0.070 | 0.257 |
|  | rs2569715 | 0.36(-0.603-1.322) | 0.465 | 0.731 |
|  | rs2569716 | -1.069(-2.048--0.091) | **0.034** | 0.257 |
|  | rs3745871 | -0.884(-1.804-0.036) | 0.061 | 0.257 |
|  | rs11540761 | 0.147(-1.09-1.385) | 0.816 | 0.889 |
|  | rs11574576 | -0.579(-1.568-0.411) | 0.253 | 0.557 |
|  | rs28366008 | 0.149(-0.96-1.259) | 0.793 | 0.889 |
| LXFLUEF | rs731170 | -0.229(-1.332-0.874) | 0.685 | 0.795 |
|  | rs1048801 | -0.697(-1.751-0.357) | 0.196 | 0.659 |
|  | rs1749316 | 0.563(-0.521-1.646) | 0.310 | 0.659 |
|  | rs1749317 | 0.18(-0.973-1.333) | 0.760 | 0.795 |
|  | rs1925241 | -0.554(-1.543-0.434) | 0.273 | 0.659 |
|  | rs2569715 | 0.442(-0.602-1.486) | 0.408 | 0.659 |
|  | rs2569716 | -0.469(-1.543-0.606) | 0.394 | 0.659 |
|  | rs3745871 | -0.412(-1.409-0.585) | 0.419 | 0.659 |
|  | rs11540761 | 0.178(-1.161-1.516) | 0.795 | 0.795 |
|  | rs11574576 | -0.846(-1.908-0.216) | 0.120 | 0.659 |
|  | rs28366008 | 0.423(-0.778-1.624) | 0.491 | 0.675 |
| LXFLUES | rs731170 | 0.221(-0.982-1.424) | 0.719 | 0.892 |
|  | rs1048801 | -0.788(-1.933-0.356) | 0.179 | 0.340 |
|  | rs1749316 | 0.939(-0.237-2.115) | 0.119 | 0.328 |
|  | rs1749317 | 0.021(-1.25-1.292) | 0.974 | 0.974 |
|  | rs1925241 | -1.256(-2.323--0.189) | **0.022** | 0.122 |
|  | rs2569715 | 0.369(-0.769-1.507) | 0.526 | 0.827 |
|  | rs2569716 | -1.253(-2.41c--0.097) | **0.035** | 0.129 |
|  | rs3745871 | -1.365(-2.444--0.286) | **0.014** | 0.122 |
|  | rs11540761 | 0.178(-1.284-1.64c) | 0.811 | 0.892 |
|  | rs11574576 | -0.793(-1.96c-0.375) | 0.185 | 0.340 |
|  | rs28366008 | -0.192(-1.503-1.119) | 0.775 | 0.892 |
| MoCA | rs731170 | 0.528(0.045-1.011) | **0.033** | 0.159 |
|  | rs1048801 | 0.288(-0.167-0.742) | 0.216 | 0.339 |
|  | rs1749316 | -0.043(-0.532-0.446) | 0.863 | 0.863 |
|  | rs1749317 | 0.043(-0.432-0.519) | 0.858 | 0.863 |
|  | rs1925241 | -0.482(-0.919--0.045) | **0.032** | 0.159 |
|  | rs2569715 | -0.11c(-0.555-0.336) | 0.630 | 0.863 |
|  | rs2569716 | 0.06c(-0.408-0.527) | 0.803 | 0.863 |
|  | rs3745871 | -0.419(-0.872-0.033) | 0.070 | 0.159 |
|  | rs11540761 | -0.516(-1.077-0.045) | 0.072 | 0.159 |
|  | rs11574576 | -0.461(-0.926-0.005) | 0.053 | 0.159 |
|  | rs28366008 | 0.432(-0.095-0.96) | 0.109 | 0.200 |
| Semantic Fluency | rs731170 | 1.284(-0.561-3.128) | 0.173 | 0.382 |
|  | rs1048801 | -0.337(-2.069-1.395) | 0.703 | 0.703 |
|  | rs1749316 | 1.508(-0.343-3.36c) | 0.111 | 0.306 |
|  | rs1749317 | 0.491(-1.317-2.299) | 0.595 | 0.654 |
|  | rs1925241 | -1.556(-3.223-0.11c) | 0.068 | 0.250 |
|  | rs2569715 | 0.508(-1.188-2.203) | 0.558 | 0.654 |
|  | rs2569716 | -0.758(-2.524-1.007) | 0.401 | 0.610 |
|  | rs3745871 | -2.342(-4.052--0.632) | **0.008** | **0.042** |
|  | rs11540761 | -1.245(-3.383-0.894) | 0.255 | 0.467 |
|  | rs11574576 | -2.619(-4.373--0.864) | **0.004** | **0.041** |
|  | rs28366008 | 0.786(-1.224-2.797) | 0.444 | 0.610 |

CI, confidence internal; FDR, false discovery rate; ADL, Modified Schwab & England Activities of Daily Living Test; BJLOT, Benton Judgement of Line Orientation; BNT, Modified Boston Naming Test; FDR, false discovery rate; HVLT, Hopkins Verbal Learning Test; LNS, Letter-Number Sequencing Test; LXFLUEA, Lexical Fluency-A; LXFLUEF, Lexical Fluency-F; LXFLUES, Lexical Fluency-S; MoCA, Montreal Cognitive Assessment; SFT, semantic fluency test.
