# Supplementary material for: Uncovering the Associations of LILRB4 Genotypes With Parkinson's Disease: From Clinical Traits to Potential Pathologies
Source: CNS Neurosci Ther. 2025 Jul 23;31(7):e70522. doi: 10.1111/cns.70522 (PMC12287542; doi:10.1111/cns.70522)
Supplement: Supplementary file 4 — Table S1. [file CNS-31-e70522-s002.zip › cns70522-sup-0016-TableS35-S37@Supplementary Table 35-37 Model 3_The correlation between LILRB4 loci and scales.docx]

**Supplementary Table 35**. Model 3: The correlation between *LILRB4* loci and scales of cognitive function.

| Items | SNP | β(95%CI) | P value | FDR-corrected. P |
| --- | --- | --- | --- | --- |
| ADL | rs731170 | 1.406(-1.235-4.047) | 0.303 | 0.824 |
|  | rs1048801 | -0.532(-2.752-1.687) | 0.641 | 0.824 |
|  | rs1749316 | 0.371(-2.301-3.042) | 0.787 | 0.828 |
|  | rs1749317 | -0.636(-3.460-2.188) | 0.661 | 0.824 |
|  | rs1925241 | -1.142(-3.297-1.012) | 0.305 | 0.824 |
|  | rs2569715 | 0.510(-1.776-2.796) | 0.664 | 0.824 |
|  | rs2569716 | 0.256(-2.037-2.548) | 0.828 | 0.828 |
|  | rs3745871 | -0.941(-3.187-1.306) | 0.417 | 0.824 |
|  | rs11540761 | -3.427(-6.374--0.481) | **0.028** | 0.31 |
|  | rs11574576 | -0.482(-2.713-1.748) | 0.674 | 0.824 |
|  | rs28366008 | 1.388(-1.050-3.825) | 0.271 | 0.824 |
| BJLOT | rs731170 | 1.822(-0.086-3.730) | 0.069 | 0.252 |
|  | rs1048801 | 0.979(-0.648-2.607) | 0.245 | 0.337 |
|  | rs1749316 | 1.279(-0.670-3.227) | 0.206 | 0.324 |
|  | rs1749317 | 0.821(-1.270-2.911) | 0.446 | 0.545 |
|  | rs1925241 | -2.618(-4.02--1.216) | **0.001** | **0.008** |
|  | rs2569715 | -0.121(-1.826-1.583) | 0.89 | 0.89 |
|  | rs2569716 | 1.120(-0.550-2.790) | 0.196 | 0.324 |
|  | rs3745871 | -1.428(-3.053-0.197) | 0.093 | 0.255 |
|  | rs11540761 | -3.442(-5.51--1.373) | **0.002** | **0.013** |
|  | rs11574576 | -0.351(-2.011-1.309) | 0.681 | 0.749 |
|  | rs28366008 | 1.307(-0.489-3.103) | 0.162 | 0.324 |
| BNT | rs731170 | 2.284(-3.668-8.237) | 0.457 | 0.728 |
|  | rs1048801 | 5.290(0.520-10.059) | **0.037** | 0.357 |
|  | rs1749316 | 1.822(-4.653-8.298) | 0.585 | 0.728 |
|  | rs1749317 | 3.250(-3.027-9.527) | 0.317 | 0.728 |
|  | rs1925241 | -4.734(-9.601-0.133) | 0.065 | 0.357 |
|  | rs2569715 | 0.218(-5.126-5.561) | 0.937 | 0.937 |
|  | rs2569716 | 2.471(-2.874-7.817) | 0.371 | 0.728 |
|  | rs3745871 | -1.541(-6.757-3.675) | 0.566 | 0.728 |
|  | rs11540761 | -5.641(-12.501-1.219) | 0.116 | 0.425 |
|  | rs11574576 | -0.832(-5.979-4.315) | 0.753 | 0.829 |
|  | rs28366008 | 1.631(-4.343-7.605) | 0.596 | 0.728 |
| HVLT | rs731170 | 2.858(-1.169-6.886) | 0.172 | 0.596 |
|  | rs1048801 | 0.143(-3.288-3.573) | 0.935 | 0.962 |
|  | rs1749316 | -0.100(-4.221-4.021) | 0.962 | 0.962 |
|  | rs1749317 | 0.879(-3.475-5.234) | 0.694 | 0.849 |
|  | rs1925241 | -1.985(-5.293-1.323) | 0.247 | 0.596 |
|  | rs2569715 | 1.214(-2.297-4.726) | 0.502 | 0.69 |
|  | rs2569716 | 3.692(0.352-7.032) | **0.036** | 0.401 |
|  | rs3745871 | -1.558(-5.016-1.900) | 0.383 | 0.69 |
|  | rs11540761 | -2.712(-7.471-2.046) | 0.271 | 0.596 |
|  | rs11574576 | -2.142(-5.521-1.238) | 0.222 | 0.596 |
|  | rs28366008 | 1.354(-2.438-5.147) | 0.488 | 0.69 |
| LNS | rs731170 | -0.734(-2.020-0.552) | 0.27 | 0.476 |
|  | rs1048801 | 0.295(-0.787-1.377) | 0.596 | 0.688 |
|  | rs1749316 | -0.794(-2.075-0.487) | 0.232 | 0.476 |
|  | rs1749317 | 0.725(-0.637-2.088) | 0.303 | 0.476 |
|  | rs1925241 | 1.172(0.171-2.172) | **0.027** | 0.202 |
|  | rs2569715 | 0.637(-0.464-1.737) | 0.264 | 0.476 |
|  | rs2569716 | 0.118(-1.001-1.237) | 0.837 | 0.837 |
|  | rs3745871 | 0.937(-0.129-2.003) | 0.093 | 0.34 |
|  | rs11540761 | 0.383(-1.143-1.909) | 0.625 | 0.688 |
|  | rs11574576 | -0.499(-1.579-0.581) | 0.371 | 0.509 |
|  | rs28366008 | 1.261(0.119-2.402) | **0.037** | 0.202 |
| LXFLUEA | rs731170 | -1.128(-3.839-1.583) | 0.42 | 0.767 |
|  | rs1048801 | 1.723(-0.521-3.966) | 0.141 | 0.596 |
|  | rs1749316 | -0.408(-3.340-2.525) | 0.787 | 0.787 |
|  | rs1749317 | 1.557(-1.301-4.415) | 0.293 | 0.767 |
|  | rs1925241 | 1.029(-1.260-3.318) | 0.384 | 0.767 |
|  | rs2569715 | 1.707(-0.640-4.053) | 0.163 | 0.596 |
|  | rs2569716 | -0.609(-3.057-1.839) | 0.629 | 0.767 |
|  | rs3745871 | 0.675(-1.677-3.026) | 0.577 | 0.767 |
|  | rs11540761 | 3.156(0.122-6.190) | **0.049** | 0.536 |
|  | rs11574576 | -0.455(-2.734-1.824) | 0.698 | 0.767 |
|  | rs28366008 | -0.628(-3.350-2.094) | 0.654 | 0.767 |
| LXFLUEF | rs731170 | 0.844(-1.329-3.017) | 0.452 | 0.778 |
|  | rs1048801 | 1.005(-0.817-2.827) | 0.287 | 0.778 |
|  | rs1749316 | -0.570(-2.913-1.774) | 0.637 | 0.778 |
|  | rs1749317 | 1.884(-0.359-4.127) | 0.108 | 0.595 |
|  | rs1925241 | 0.073(-1.779-1.926) | 0.939 | 0.993 |
|  | rs2569715 | 1.708(-0.142-3.558) | 0.079 | 0.595 |
|  | rs2569716 | -0.567(-2.525-1.391) | 0.574 | 0.778 |
|  | rs3745871 | -0.587(-2.468-1.295) | 0.545 | 0.778 |
|  | rs11540761 | 0.846(-1.702-3.394) | 0.519 | 0.778 |
|  | rs11574576 | -0.008(-1.836-1.820) | 0.993 | 0.993 |
|  | rs28366008 | 1.316(-0.828-3.461) | 0.237 | 0.778 |
| LXFLUES | rs731170 | 0.328(-2.445-3.101) | 0.818 | 0.9 |
|  | rs1048801 | 1.050(-1.270-3.370) | 0.381 | 0.73 |
|  | rs1749316 | -2.057(-4.960-0.847) | 0.173 | 0.73 |
|  | rs1749317 | 0.945(-1.983-3.873) | 0.531 | 0.73 |
|  | rs1925241 | 1.115(-1.204-3.434) | 0.352 | 0.73 |
|  | rs2569715 | 0.973(-1.452-3.398) | 0.437 | 0.73 |
|  | rs2569716 | -1.344(-3.798-1.109) | 0.29 | 0.73 |
|  | rs3745871 | 0.636(-1.751-3.023) | 0.605 | 0.739 |
|  | rs11540761 | 1.170(-2.055-4.394) | 0.482 | 0.73 |
|  | rs11574576 | -0.046(-2.362-2.270) | 0.969 | 0.969 |
|  | rs28366008 | 1.489(-1.238-4.217) | 0.291 | 0.73 |
| MoCA | rs731170 | -0.146(-1.105-0.813) | 0.767 | 0.872 |
|  | rs1048801 | -0.309(-1.101-0.483) | 0.449 | 0.848 |
|  | rs1749316 | -0.615(-1.554-0.325) | 0.207 | 0.6 |
|  | rs1749317 | -0.137(-1.151-0.877) | 0.793 | 0.872 |
|  | rs1925241 | 0.490(-0.278-1.259) | 0.218 | 0.6 |
|  | rs2569715 | 0.111(-0.710-0.932) | 0.793 | 0.872 |
|  | rs2569716 | -0.291(-1.109-0.526) | 0.489 | 0.848 |
|  | rs3745871 | 0.528(-0.267-1.324) | 0.201 | 0.6 |
|  | rs11540761 | -0.353(-1.473-0.766) | 0.54 | 0.848 |
|  | rs11574576 | -0.020(-0.822-0.782) | 0.962 | 0.962 |
|  | rs28366008 | 0.686(-0.176-1.547) | 0.127 | 0.6 |
| SFT | rs731170 | -1.523(-7.376-4.330) | 0.613 | 0.834 |
|  | rs1048801 | 0.040(-4.843-4.922) | 0.987 | 0.987 |
|  | rs1749316 | 2.051(-3.778-7.881) | 0.494 | 0.834 |
|  | rs1749317 | 4.150(-1.921-10.222) | 0.188 | 0.689 |
|  | rs1925241 | -0.757(-5.542-4.028) | 0.758 | 0.834 |
|  | rs2569715 | 4.160(-0.694-9.014) | 0.101 | 0.556 |
|  | rs2569716 | -1.289(-6.304-3.726) | 0.617 | 0.834 |
|  | rs3745871 | -1.023(-5.983-3.937) | 0.688 | 0.834 |
|  | rs11540761 | -3.009(-9.823-3.806) | 0.392 | 0.834 |
|  | rs11574576 | -4.451(-9.152-0.250) | 0.071 | 0.556 |
|  | rs28366008 | 1.014(-4.408-6.436) | 0.716 | 0.834 |

CI, confidence internal; FDR, false discovery rate; ADL, Modified Schwab & England Activities of Daily Living Test; BJLOT, Benton Judgement of Line Orientation; BNT, Modified Boston Naming Test; FDR, false discovery rate; HVLT, Hopkins Verbal Learning Test; LNS, Letter-Number Sequencing Test; LXFLUEA, Lexical Fluency-A; LXFLUEF, Lexical Fluency-F; LXFLUES, Lexical Fluency-S; MoCA, Montreal Cognitive Assessment; SFT, semantic fluency test.

**Supplementary Table 36** Model 3: The correlation between *LILRB4* loci and scales of cognitive function in male

| Items | SNP | β(95%CI) | P value | FDR-corrected. P |
| --- | --- | --- | --- | --- |
| ADL | rs731170 | 0.371(-2.718-3.460) | 0.817 | 0.817 |
|  | rs1048801 | -2.648(-4.703--0.593) | **0.021** | 0.214 |
|  | rs1749316 | -3.051(-6.316-0.213) | 0.084 | 0.23 |
|  | rs1749317 | -2.935(-5.677--0.194) | 0.05 | 0.214 |
|  | rs1925241 | 2.763(0.083-5.443) | 0.058 | 0.214 |
|  | rs2569715 | 1.182(-1.684-4.047) | 0.43 | 0.594 |
|  | rs2569716 | 1.349(-1.226-3.923) | 0.318 | 0.583 |
|  | rs3745871 | 2.197(-0.936-5.331) | 0.186 | 0.41 |
|  | rs11540761 | -1.952(-6.708-2.805) | 0.432 | 0.594 |
|  | rs11574576 | 0.314(-2.274-2.902) | 0.815 | 0.817 |
|  | rs28366008 | 0.712(-2.642-4.065) | 0.682 | 0.817 |
| BJLOT | rs731170 | 1.160(-0.776-3.096) | 0.256 | 0.849 |
|  | rs1048801 | 0.300(-1.246-1.846) | 0.708 | 0.854 |
|  | rs1749316 | -0.339(-2.642-1.964) | 0.776 | 0.854 |
|  | rs1749317 | 0.432(-1.544-2.408) | 0.674 | 0.854 |
|  | rs1925241 | -0.855(-2.742-1.031) | 0.386 | 0.849 |
|  | rs2569715 | 0.658(-1.211-2.528) | 0.499 | 0.854 |
|  | rs2569716 | 1.509(-0.063-3.082) | 0.076 | 0.419 |
|  | rs3745871 | -1.024(-3.109-1.061) | 0.349 | 0.849 |
|  | rs11540761 | -0.758(-3.881-2.366) | 0.64 | 0.854 |
|  | rs11574576 | -0.127(-1.809-1.555) | 0.884 | 0.884 |
|  | rs28366008 | 1.979(-0.009-3.966) | 0.067 | 0.419 |
| BNT | rs731170 | 0.939(-0.672-2.550) | 0.268 | 0.763 |
|  | rs1048801 | 0.694(-0.555-1.943) | 0.29 | 0.763 |
|  | rs1749316 | -0.612(-2.508-1.285) | 0.535 | 0.763 |
|  | rs1749317 | 0.688(-0.931-2.306) | 0.416 | 0.763 |
|  | rs1925241 | -0.260(-1.856-1.336) | 0.753 | 0.828 |
|  | rs2569715 | 1.002(-0.502-2.505) | 0.208 | 0.763 |
|  | rs2569716 | 0.588(-0.814-1.991) | 0.422 | 0.763 |
|  | rs3745871 | -0.539(-2.298-1.219) | 0.555 | 0.763 |
|  | rs11540761 | 1.559(-0.950-4.068) | 0.239 | 0.763 |
|  | rs11574576 | 0.290(-1.101-1.682) | 0.687 | 0.828 |
|  | rs28366008 | 0.028(-1.789-1.844) | 0.976 | 0.976 |
| HVLT | rs731170 | 3.542(-2.509-9.593) | 0.266 | 0.569 |
|  | rs1048801 | -0.334(-5.176-4.507) | 0.894 | 0.994 |
|  | rs1749316 | 0.026(-7.178-7.230) | 0.994 | 0.994 |
|  | rs1749317 | -3.204(-9.222-2.813) | 0.311 | 0.569 |
|  | rs1925241 | -3.292(-9.110-2.526) | 0.282 | 0.569 |
|  | rs2569715 | 6.309(1.167-11.450) | **0.027** | 0.149 |
|  | rs2569716 | 6.851(2.517-11.185) | **0.006** | 0.068 |
|  | rs3745871 | -4.036(-10.442-2.371) | 0.233 | 0.569 |
|  | rs11540761 | 0.093(-9.715-9.901) | 0.985 | 0.994 |
|  | rs11574576 | -1.023(-6.253-4.207) | 0.706 | 0.971 |
|  | rs28366008 | -2.772(-9.477-3.932) | 0.428 | 0.673 |
| LNS | rs731170 | -0.519(-2.300-1.262) | 0.575 | 0.596 |
|  | rs1048801 | 0.38(-0.998-1.757) | 0.596 | 0.596 |
|  | rs1749316 | -0.638(-2.682-1.406) | 0.548 | 0.596 |
|  | rs1749317 | 1.818(0.252-3.383) | **0.035** | 0.194 |
|  | rs1925241 | 1.388(-0.213-2.988) | 0.106 | 0.373 |
|  | rs2569715 | 1.881(0.426-3.335) | **0.021** | 0.194 |
|  | rs2569716 | 0.789(-0.706-2.283) | 0.315 | 0.577 |
|  | rs3745871 | 1.135(-0.704-2.975) | 0.242 | 0.533 |
|  | rs11540761 | 2.103(-0.536-4.741) | 0.136 | 0.373 |
|  | rs11574576 | -0.447(-1.938-1.044) | 0.564 | 0.596 |
|  | rs28366008 | 0.822(-1.097-2.742) | 0.412 | 0.596 |
| LXFLUEA | rs731170 | -0.695(-4.754-3.365) | 0.741 | 0.909 |
|  | rs1048801 | 2.773(-0.101-5.648) | 0.075 | 0.274 |
|  | rs1749316 | 0.064(-4.616-4.743) | 0.979 | 0.979 |
|  | rs1749317 | 4.017(0.444-7.589) | **0.041** | 0.224 |
|  | rs1925241 | 0.696(-3.197-4.590) | 0.73 | 0.909 |
|  | rs2569715 | 2.641(-1.000-6.281) | 0.172 | 0.474 |
|  | rs2569716 | 1.164(-2.281-4.608) | 0.516 | 0.909 |
|  | rs3745871 | 0.732(-3.589-5.054) | 0.744 | 0.909 |
|  | rs11540761 | 8.555(3.557-13.553) | **0.004** | **0.039** |
|  | rs11574576 | -1.227(-4.591-2.137) | 0.484 | 0.909 |
|  | rs28366008 | -0.298(-4.730-4.134) | 0.897 | 0.979 |
| LXFLUEF | rs731170 | 1.205(-2.453-4.862) | 0.527 | 0.828 |
|  | rs1048801 | 2.286(-0.371-4.943) | 0.109 | 0.4 |
|  | rs1749316 | -1.443(-5.642-2.756) | 0.509 | 0.828 |
|  | rs1749317 | 3.920(0.741-7.098) | **0.027** | 0.292 |
|  | rs1925241 | 0.491(-3.051-4.034) | 0.789 | 0.95 |
|  | rs2569715 | 3.087(-0.097-6.270) | 0.074 | 0.4 |
|  | rs2569716 | 0.811(-2.335-3.957) | 0.619 | 0.852 |
|  | rs3745871 | -0.127(-4.065-3.811) | 0.95 | 0.95 |
|  | rs11540761 | 4.232(-1.218-9.681) | 0.145 | 0.4 |
|  | rs11574576 | -0.246(-3.343-2.852) | 0.878 | 0.95 |
|  | rs28366008 | 2.554(-1.298-6.406) | 0.21 | 0.462 |
| LXFLUES | rs731170 | -0.154(-3.986-3.677) | 0.938 | 0.938 |
|  | rs1048801 | 0.675(-2.270-3.620) | 0.659 | 0.805 |
|  | rs1749316 | -1.490(-5.840-2.860) | 0.51 | 0.758 |
|  | rs1749317 | 2.112(-1.549-5.773) | 0.273 | 0.758 |
|  | rs1925241 | 1.550(-2.055-5.156) | 0.41 | 0.758 |
|  | rs2569715 | 2.520(-0.900-5.940) | 0.166 | 0.758 |
|  | rs2569716 | -0.378(-3.655-2.898) | 0.823 | 0.905 |
|  | rs3745871 | 1.453(-2.570-5.477) | 0.488 | 0.758 |
|  | rs11540761 | 4.180(-1.497-9.856) | 0.166 | 0.758 |
|  | rs11574576 | 1.058(-2.115-4.231) | 0.522 | 0.758 |
|  | rs28366008 | 1.282(-2.848-5.413) | 0.551 | 0.758 |
| MoCA | rs731170 | -0.125(-1.235-0.985) | 0.827 | 0.866 |
|  | rs1048801 | -0.976(-1.707--0.245) | **0.018** | 0.106 |
|  | rs1749316 | -1.183(-2.337--0.028) | 0.06 | 0.155 |
|  | rs1749317 | -0.473(-1.550-0.604) | 0.401 | 0.735 |
|  | rs1925241 | 1.196(0.284-2.108) | **0.019** | 0.106 |
|  | rs2569715 | 0.936(-0.018-1.891) | 0.07 | 0.155 |
|  | rs2569716 | 0.083(-0.868-1.034) | 0.866 | 0.866 |
|  | rs3745871 | 1.157(0.101-2.212) | **0.046** | 0.155 |
|  | rs11540761 | 0.549(-1.172-2.269) | 0.54 | 0.743 |
|  | rs11574576 | -0.183(-1.111-0.744) | 0.703 | 0.859 |
|  | rs28366008 | 0.424(-0.770-1.618) | 0.495 | 0.743 |
| SFT | rs731170 | 1.513(-7.628-10.653) | 0.749 | 0.982 |
|  | rs1048801 | -2.300(-9.305-4.705) | 0.528 | 0.982 |
|  | rs1749316 | -2.128(-12.618-8.362) | 0.696 | 0.982 |
|  | rs1749317 | 0.583(-8.476-9.642) | 0.901 | 0.982 |
|  | rs1925241 | 0.820(-7.967-9.607) | 0.857 | 0.982 |
|  | rs2569715 | 9.624(2.212-17.036) | **0.02** | 0.223 |
|  | rs2569716 | 1.717(-6.092-9.526) | 0.672 | 0.982 |
|  | rs3745871 | 0.111(-9.647-9.870) | 0.982 | 0.982 |
|  | rs11540761 | -1.606(-15.931-12.72) | 0.829 | 0.982 |
|  | rs11574576 | -6.257(-13.373-0.858) | 0.102 | 0.561 |
|  | rs28366008 | 1.736(-8.214-11.687) | 0.736 | 0.982 |

CI, confidence internal; FDR, false discovery rate; ADL, Modified Schwab & England Activities of Daily Living Test; BJLOT, Benton Judgement of Line Orientation; BNT, Modified Boston Naming Test; FDR, false discovery rate; HVLT, Hopkins Verbal Learning Test; LNS, Letter-Number Sequencing Test; LXFLUEA, Lexical Fluency-A; LXFLUEF, Lexical Fluency-F; LXFLUES, Lexical Fluency-S; MoCA, Montreal Cognitive Assessment; SFT, semantic fluency test.

**Supplementary Table 37** Model 3: The correlation between *LILRB4* loci and scales of cognitive function in female

| Items | SNP | β(95%CI) | P value | FDR-corrected. P |
| --- | --- | --- | --- | --- |
| ADL | rs731170 | 2.166(-2.539-6.87) | 0.380 | 0.523 |
|  | rs1048801 | 2.695(-1.933-7.322) | 0.270 | 0.495 |
|  | rs1749316 | 2.978(-1.074-7.031) | 0.169 | 0.401 |
|  | rs1749317 | 3.820(-1.550-9.190) | 0.182 | 0.401 |
|  | rs1925241 | -3.462(-6.513--0.411) | **0.041** | 0.327 |
|  | rs2569715 | -0.600(-4.501-3.301) | 0.767 | 0.818 |
|  | rs2569716 | -2.193(-6.815-2.430) | 0.366 | 0.523 |
|  | rs3745871 | -2.657(-5.898-0.585) | 0.128 | 0.401 |
|  | rs11540761 | -4.694(-9.228--0.160) | 0.059 | 0.327 |
|  | rs11574576 | -0.581(-5.443-4.280) | 0.818 | 0.818 |
|  | rs28366008 | 1.193(-2.964-5.349) | 0.582 | 0.711 |
| BJLOT | rs731170 | 1.846(-1.717-5.408) | 0.325 | 0.585 |
|  | rs1048801 | 2.267(-1.228-5.761) | 0.222 | 0.550 |
|  | rs1749316 | 2.631(-0.387-5.650) | 0.107 | 0.392 |
|  | rs1749317 | 1.978(-2.246-6.202) | 0.372 | 0.585 |
|  | rs1925241 | -3.593(-5.588--1.599) | **0.003** | **0.030** |
|  | rs2569715 | -0.871(-3.821-2.080) | 0.571 | 0.785 |
|  | rs2569716 | 0.694(-2.907-4.294) | 0.711 | 0.803 |
|  | rs3745871 | -1.553(-4.104-0.998) | 0.250 | 0.550 |
|  | rs11540761 | -4.543(-7.714--1.372) | **0.013** | 0.069 |
|  | rs11574576 | 0.361(-3.346-4.069) | 0.851 | 0.851 |
|  | rs28366008 | 0.571(-2.615-3.758) | 0.730 | 0.803 |
| BNT | rs731170 | 3.829(-10.642-18.301) | 0.613 | 0.768 |
|  | rs1048801 | 11.883(-0.957-24.724) | 0.095 | 0.521 |
|  | rs1749316 | 4.737(-9.84-19.315) | 0.536 | 0.768 |
|  | rs1749317 | 4.476(-12.772-21.723) | 0.620 | 0.768 |
|  | rs1925241 | -7.726(-17.801-2.350) | 0.159 | 0.582 |
|  | rs2569715 | 1.978(-10.888-14.844) | 0.768 | 0.768 |
|  | rs2569716 | 5.24(-10.201-20.681) | 0.519 | 0.768 |
|  | rs3745871 | -2.261(-13.041-8.519) | 0.688 | 0.768 |
|  | rs11540761 | -12.527(-25.661-0.607) | 0.086 | 0.521 |
|  | rs11574576 | -2.864(-17.353-11.625) | 0.705 | 0.768 |
|  | rs28366008 | 3.947(-10.294-18.187) | 0.597 | 0.768 |
| HVLT | rs731170 | 3.899(-1.803-9.601) | 0.199 | 0.421 |
|  | rs1048801 | 0.526(-5.468-6.520) | 0.866 | 0.979 |
|  | rs1749316 | -0.203(-5.572-5.167) | 0.942 | 0.979 |
|  | rs1749317 | 7.356(1.25-13.462) | **0.031** | 0.121 |
|  | rs1925241 | -1.863(-6.119-2.393) | 0.404 | 0.634 |
|  | rs2569715 | -2.968(-7.624-1.688) | 0.229 | 0.421 |
|  | rs2569716 | -0.080(-5.996-5.837) | 0.979 | 0.979 |
|  | rs3745871 | -1.140(-5.459-3.179) | 0.612 | 0.842 |
|  | rs11540761 | -7.257(-12.505--2.009) | **0.015** | 0.121 |
|  | rs11574576 | -6.246(-11.490--1.001) | **0.033** | 0.121 |
|  | rs28366008 | 4.384(-0.388-9.156) | 0.091 | 0.249 |
| LNS | rs731170 | -0.547(-2.579-1.485) | 0.605 | 0.739 |
|  | rs1048801 | 0.324(-1.715-2.363) | 0.760 | 0.836 |
|  | rs1749316 | -0.715(-2.511-1.082) | 0.447 | 0.739 |
|  | rs1749317 | -0.123(-2.539-2.293) | 0.922 | 0.922 |
|  | rs1925241 | 0.837(-0.589-2.263) | 0.267 | 0.739 |
|  | rs2569715 | -0.565(-2.205-1.074) | 0.509 | 0.739 |
|  | rs2569716 | -1.200(-3.129-0.729) | 0.240 | 0.739 |
|  | rs3745871 | 0.548(-0.912-2.008) | 0.472 | 0.739 |
|  | rs11540761 | -1.065(-3.162-1.032) | 0.334 | 0.739 |
|  | rs11574576 | -0.621(-2.668-1.426) | 0.561 | 0.739 |
|  | rs28366008 | 1.518(-0.103-3.139) | 0.085 | 0.739 |
| LXFLUEA | rs731170 | -2.334(-6.216-1.547) | 0.258 | 0.568 |
|  | rs1048801 | 0.803(-3.267-4.872) | 0.705 | 0.835 |
|  | rs1749316 | 0.109(-4.087-4.306) | 0.960 | 0.960 |
|  | rs1749317 | -0.779(-5.662-4.105) | 0.759 | 0.835 |
|  | rs1925241 | 1.083(-1.947-4.113) | 0.495 | 0.835 |
|  | rs2569715 | 0.910(-2.544-4.363) | 0.614 | 0.835 |
|  | rs2569716 | -4.069(-7.873--0.264) | 0.055 | 0.568 |
|  | rs3745871 | 0.491(-2.537-3.520) | 0.755 | 0.835 |
|  | rs11540761 | 2.493(-1.614-6.599) | 0.254 | 0.568 |
|  | rs11574576 | 2.748(-1.054-6.550) | 0.178 | 0.568 |
|  | rs28366008 | -2.284(-6.048-1.480) | 0.254 | 0.568 |
| LXFLUEF | rs731170 | -0.236(-2.781-2.310) | 0.859 | 0.973 |
|  | rs1048801 | -0.916(-3.432-1.601) | 0.487 | 0.903 |
|  | rs1749316 | 0.603(-2.006-3.212) | 0.657 | 0.903 |
|  | rs1749317 | -0.775(-3.816-2.267) | 0.625 | 0.903 |
|  | rs1925241 | -0.142(-2.071-1.787) | 0.887 | 0.973 |
|  | rs2569715 | 0.999(-1.120-3.119) | 0.371 | 0.903 |
|  | rs2569716 | -2.911(-5.176--0.646) | **0.025** | 0.271 |
|  | rs3745871 | -0.779(-2.638-1.081) | 0.426 | 0.903 |
|  | rs11540761 | 0.652(-2.024-3.329) | 0.640 | 0.903 |
|  | rs11574576 | 1.759(-0.614-4.133) | 0.168 | 0.903 |
|  | rs28366008 | -0.044(-2.517-2.430) | 0.973 | 0.973 |
| LXFLUES | rs731170 | -0.031(-4.282-4.220) | 0.989 | 0.989 |
|  | rs1048801 | 2.325(-1.772-6.421) | 0.285 | 0.989 |
|  | rs1749316 | -1.412(-5.733-2.910) | 0.532 | 0.989 |
|  | rs1749317 | 0.638(-4.471-5.746) | 0.810 | 0.989 |
|  | rs1925241 | 0.653(-2.549-3.855) | 0.695 | 0.989 |
|  | rs2569715 | -0.217(-3.857-3.424) | 0.909 | 0.989 |
|  | rs2569716 | -3.992(-8.039-0.056) | 0.074 | 0.811 |
|  | rs3745871 | -0.048(-3.223-3.128) | 0.977 | 0.989 |
|  | rs11540761 | 1.459(-2.977-5.895) | 0.530 | 0.989 |
|  | rs11574576 | 1.479(-2.697-5.655) | 0.499 | 0.989 |
|  | rs28366008 | 0.345(-3.777-4.467) | 0.872 | 0.989 |
| MoCA | rs731170 | 0.145(-1.516-1.807) | 0.866 | 0.972 |
|  | rs1048801 | 1.069(-0.505-2.644) | 0.202 | 0.717 |
|  | rs1749316 | -0.064(-1.549-1.421) | 0.933 | 0.972 |
|  | rs1749317 | 0.861(-1.054-2.777) | 0.391 | 0.717 |
|  | rs1925241 | -0.198(-1.398-1.002) | 0.751 | 0.972 |
|  | rs2569715 | -0.664(-1.973-0.645) | 0.335 | 0.717 |
|  | rs2569716 | -0.968(-2.534-0.599) | 0.244 | 0.717 |
|  | rs3745871 | -0.022(-1.226-1.183) | 0.972 | 0.972 |
|  | rs11540761 | -1.188(-2.842-0.465) | 0.178 | 0.717 |
|  | rs11574576 | 0.607(-1.046-2.259) | 0.482 | 0.758 |
|  | rs28366008 | 0.806(-0.586-2.199) | 0.273 | 0.717 |
| SFT | rs731170 | -5.382(-13.671-2.906) | 0.221 | 0.608 |
|  | rs1048801 | 2.493(-6.100-11.085) | 0.578 | 0.868 |
|  | rs1749316 | 5.641(-1.619-12.900) | 0.147 | 0.539 |
|  | rs1749317 | 11.448(2.861-20.034) | **0.019** | 0.207 |
|  | rs1925241 | -1.541(-7.792-4.710) | 0.635 | 0.868 |
|  | rs2569715 | 0.224(-6.831-7.279) | 0.951 | 0.951 |
|  | rs2569716 | -8.387(-15.894--0.879) | **0.044** | 0.240 |
|  | rs3745871 | -1.991(-8.215-4.233) | 0.539 | 0.868 |
|  | rs11540761 | -4.547(-13.442-4.347) | 0.331 | 0.728 |
|  | rs11574576 | -1.471(-10.225-7.282) | 0.746 | 0.868 |
|  | rs28366008 | -1.050(-8.602-6.502) | 0.789 | 0.868 |

CI, confidence internal; FDR, false discovery rate; ADL, Modified Schwab & England Activities of Daily Living Test; BJLOT, Benton Judgement of Line Orientation; BNT, Modified Boston Naming Test; FDR, false discovery rate; HVLT, Hopkins Verbal Learning Test; LNS, Letter-Number Sequencing Test; LXFLUEA, Lexical Fluency-A; LXFLUEF, Lexical Fluency-F; LXFLUES, Lexical Fluency-S; MoCA, Montreal Cognitive Assessment; SFT, semantic fluency test.
