# Supplementary material for: Uncovering the Associations of LILRB4 Genotypes With Parkinson's Disease: From Clinical Traits to Potential Pathologies
Source: CNS Neurosci Ther. 2025 Jul 23;31(7):e70522. doi: 10.1111/cns.70522 (PMC12287542; doi:10.1111/cns.70522)
Supplement: Supplementary file 4 — Table S1. [file CNS-31-e70522-s002.zip › cns70522-sup-0017-TableS38@Supplementary Table 38. The correlations between LILRB4 genotypes and sex..docx]

**Supplementary Table 38.** The correlations between LILRB4 genotypes and sex.

| SNPs | Heterozygous mutation* | | Homozygous mutation* | |
| --- | --- | --- | --- | --- |
|  | **β** | **P-value** | **β** | **P-value** |
| rs731170 | 0.028(-0.272-0.328) | 0.855 | 0.109(-0.393-0.611) | 0.670 |
| rs1048801 | -0.203(-0.513-0.107) | 0.199 | -0.081(-0.508-0.346) | 0.710 |
| rs1749316 | -0.012(-0.310-0.286) | 0.937 | -0.243(-0.794-0.308) | 0.387 |
| rs1749317 | -0.094(-0.394-0.206) | 0.539 | -0.231(-0.727-0.265) | 0.361 |
| rs1925241 | -0.054(-0.375-0.267) | 0.742 | -0.117(-0.519-0.285) | 0.568 |
| rs2569715 | 0.194(-0.112-0.500) | 0.213 | -0.149(-0.588-0.290) | 0.506 |
| rs2569716 | -0.034(-0.340-0.272) | 0.827 | 0.009(-0.436-0.454) | 0.968 |
| rs3745871 | -0.031(-0.341-0.279) | 0.844 | -0.001(-0.428-0.426) | 0.996 |
| rs11540761 | -0.222(-0.524-0.080) | 0.149 | -0.233(-0.711-0.245) | 0.340 |
| rs11574576 | -0.142(-0.452-0.168) | 0.369 | 0.284(-0.422-0.990) | 0.430 |
| rs28366008 | -0.264(-0.562-0.034) | 0.082 | -0.116(-0.771-0.539) | 0.728 |

* Used the unmutated genotype as a reference.
