# Supplementary material for: Uncovering the Associations of LILRB4 Genotypes With Parkinson's Disease: From Clinical Traits to Potential Pathologies
Source: CNS Neurosci Ther. 2025 Jul 23;31(7):e70522. doi: 10.1111/cns.70522 (PMC12287542; doi:10.1111/cns.70522)
Supplement: Supplementary file 4 — Table S1. [file CNS-31-e70522-s002.zip › Response Letter.docx]

Dear Editor,

Thank you for your valuable questions regarding our manuscript. We have carefully addressed all the necessary corrections, which are documented in detail in the attached file. We hope our prompt response has not caused any inconvenience to your workflow and sincerely appreciate your time and effort in reviewing our work. Please let us know if any further clarifications are needed.

Best regards,

Anmu Xie

[xieanmu@163.com](mailto:xieanmu@163.com)

**Figures 1-5 & Figures S1-S3 caption.**

**Figure 1. Schematic of SNPs in the *LILRB4* gene.** UTR, untranslated region.

**Figure 2. The correlation between *LILRB4* loci and PD risk.** A) The correlation between *LILRB4* loci and PD. B) Rs731170 genotype frequency. C) Rs 731170 allele frequency. D) Rs3745871 genotype frequency. E) Rs3745871 allele frequency. FDR, false discovery rate;

HC, health control; PD, Parkinson’s disease.

**Figure 3. Brain images with *LILRB4* genotypes.** A) Rs731170 was associated with the striatal binding ratios in left caudate. B) Rs731170 was associated with the striatal binding ratios in right caudate. C) Rs731170 was associated with the striatal binding ratios in right putamen. D) Rs731170 was associated with the striatal binding ratios in right anterior putamen. E) The correlation between *LILRB4* loci and DAT-SPECT striatal binding ratios. F) Rs1925241 was associated with FA value in right rostral of SN. G) Rs1925241 was associated with FA value in right middle of SN. H) Rs3745871 was associated with FA value in right rostral of SN. I) Rs3745871 was associated with FA value in right middle of SN. FDR, false discovery rate. J) The correlation between *LILRB4* loci and DTI FA values.

**Figure 4. CSF biomarkers with *LILRB4* genotypes.** A)Rs1048801 was associated with the level of CSF α-synuclein in female. B) The correlation between *LILRB4* loci and CSF dopaminergic neuron-related biomarkers in female. C) Rs1048801 was associated with the level of CSF pTau in female. D) The correlation between *LILRB4* loci and CSF Aβ and Tau in female. 3-MT, 3-Methoxytyramine; Aβ, beta amyloid; Aβ1-42, beta amyloid 1-42; DA, dopamine; DOPA, dihydroxyphenylalanine; DOPAC, dihydroxyphenylacetic acid; FDR, false discovery rate; HVA, homovanillic acid; VMA, Vanillymandelic Acid.

**Figure 5. MDS-UPDRS and Non-motor function scales with LILRB4 genotypes.** A) Rs11574576 was associated with the score of Modifed Semantic Fluncy test. B) The correlations between *LILRB4* loci and non-motor function scales. C) Rs 3745871 was associated with the score of SFT. D) Rs 11540761 was associated with the score of SFT. E) The correlations between *LILRB4* loci and non-motor function scales in female. ADL, Modified Schwab & England Activities of Daily Living Test; BJLOT, Benton Judgement of Line Orientation; BNT, Modified Boston Naming Test; FDR, false discovery rate; HVLT, Hopkins Verbal Learning Test; LNS, Letter-Number Sequencing Test; LXFLUEA, Lexical Fluency-A; LXFLUEF, Lexical Fluency-F; LXFLUES, Lexical Fluency-S; MoCA, Montreal Cognitive Assessment; SFT, semantic fluency test.

**Supplementary Figure 1. Test for normality of continuous variables.** A) DAT-SPECT striatal binding ratios; B) DTI FA value; C) CSF biomarkers. 3-MT, 3-Methoxytyrosine; DOPA, 3,4-Dihydroxymandelic acid; DOPAC, 3,4-Dihydroxyphenylacetic acid; GFAP, glial fibrillary

acid protein; HVA, homovanillic acid; IL-1b, Interleukin 1b; IL-6, Interleukin 6; NFL, neurofilament light; S100, S-100 calcium binding protein B; sTREM2, soluble triggering receptor expressed on myeloid cells 2; VMA, Vanillymandelic Acid; YKL40, chitinase-3-like protein 1.

**Supplementary Figure 2. The significances of correlation between *LILRB4* loci and CSF biomarkers.** 3-MT, 3-Methoxytyrosine; DA, Dopamine; DOPA, 3,4-Dihydroxymandelic acid; DOPAC, 3,4-Dihydroxyphenylacetic acid; GFAP, glial fibrillary acid protein; HVA, homovanillic acid; IL-1b, Interleukin 1b; IL-6, Interleukin 6; NFL, neurofilament light; S100B, S-100 calcium binding protein B; sTREM2, soluble triggering receptor expressed on myeloid cells 2; VMA, Vanillymandelic Acid; YKL40, chitinase-3-like protein 1.

**Supplementary Figure 3. The significances of correlations between LILRB4 loci and MDS-UPDRS.**

MDS-UPDRS, Movement Disorder Society-sponsored revision of the Unified Parkinson's Disease Rating Scale; P, Patient Questionnaire; R, Rater Completed.

**The correct version of Supplementary Table 9-10, 12-13.**

**Supplementary Table 9.** Model 2: The correlation between *LILRB4* loci and PD in male.

| SNP | OR (95%CI) | P value | FDR-corrected. P |
| --- | --- | --- | --- |
| rs731170 | 0.961(0.907-1.018) | 0.174 | 0.949 |
| rs1048801 | 1.01(0.957-1.065) | 0.726 | 0.949 |
| rs1749316 | 1.029(0.968-1.094) | 0.365 | 0.949 |
| rs1749317 | 1.022(0.964-1.083) | 0.472 | 0.949 |
| rs1925241 | 0.993(0.941-1.047) | 0.791 | 0.949 |
| rs2569715 | 0.981(0.926-1.038) | 0.501 | 0.949 |
| rs2569716 | 0.99(0.937-1.046) | 0.713 | 0.871 |
| rs3745871 | 1.026(0.972-1.083) | 0.353 | 0.949 |
| rs11540761 | 1.026(0.962-1.094) | 0.432 | 0.871 |
| rs11574576 | 1.008(0.952-1.066) | 0.796 | 0.949 |
| rs28366008 | 0.981(0.919-1.046) | 0.557 | 0.949 |

CI, Confidence internal; FDR, false discovery rate; OR, odds ratio.

**Supplementary Table 10.** Model 2: The correlation between *LILRB4* loci and PD in female.

| SNP | OR (95%CI) | P value | FDR-corrected. P |
| --- | --- | --- | --- |
| rs731170 | 0.945(0.883-1.011) | 0.103 | 0.228 |
| rs1048801 | 0.973(0.913-1.037) | 0.403 | 0.555 |
| rs1749316 | 0.988(0.922-1.058) | 0.721 | 0.732 |
| rs1749317 | 1.012(0.946-1.082) | 0.732 | 0.732 |
| rs1925241 | 1.043(0.981-1.11) | 0.179 | 0.329 |
| rs2569715 | 1.071(1.007-1.14) | **0.031** | 0.101 |
| rs2569716 | 1.076(1.009-1.148) | **0.027** | 0.101 |
| rs3745871 | 1.07(1.004-1.141) | **0.037** | 0.101 |
| rs11540761 | 1.103(1.02-1.193) | **0.015** | 0.101 |
| rs11574576 | 0.97(0.908-1.036) | 0.361 | 0.555 |
| rs28366008 | 0.983(0.913-1.059) | 0.658 | 0.732 |

CI, Confidence internal; FDR, false discovery rate; OR, odds ratio.

**Supplementary Table 12**. Model 1: The correlation between *LILRB4* loci and DAT-SPECT striatal binding ratios in male.

| Items | SNP | β(95%CI) | P | FDR-corrected. P |
| --- | --- | --- | --- | --- |
| Left Caudate | rs731170 | 0.118(0.019-0.216) | **0.020** | 0.221 |
|  | rs1048801 | -0.001(-0.095-0.093) | 0.984 | 0.984 |
|  | rs1749316 | -0.061(-0.168-0.046) | 0.267 | 0.545 |
|  | rs1749317 | -0.062(-0.162-0.039) | 0.230 | 0.545 |
|  | rs1925241 | -0.042(-0.133-0.049) | 0.367 | 0.545 |
|  | rs2569715 | -0.043(-0.142-0.056) | 0.396 | 0.545 |
|  | rs2569716 | 0.031(-0.066-0.128) | 0.533 | 0.586 |
|  | rs3745871 | -0.060(-0.153-0.033) | 0.206 | 0.545 |
|  | rs11540761 | -0.057(-0.167-0.054) | 0.317 | 0.545 |
|  | rs11574576 | -0.050(-0.149-0.049) | 0.324 | 0.545 |
|  | rs28366008 | 0.042(-0.071-0.154) | 0.467 | 0.571 |
| Right Caudate | rs731170 | 0.108(0.007-0.209) | **0.036** | 0.396 |
|  | rs1048801 | 0.024(-0.071-0.120) | 0.618 | 0.755 |
|  | rs1749316 | -0.086(-0.195-0.023) | 0.123 | 0.594 |
|  | rs1749317 | -0.063(-0.165-0.040) | 0.232 | 0.594 |
|  | rs1925241 | -0.019(-0.112-0.074) | 0.692 | 0.761 |
|  | rs2569715 | -0.044(-0.145-0.057) | 0.392 | 0.682 |
|  | rs2569716 | -0.003(-0.102-0.095) | 0.947 | 0.947 |
|  | rs3745871 | -0.027(-0.122-0.068) | 0.578 | 0.755 |
|  | rs11540761 | -0.045(-0.158-0.068) | 0.434 | 0.682 |
|  | rs11574576 | -0.063(-0.164-0.038) | 0.224 | 0.594 |
|  | rs28366008 | 0.064(-0.050-0.179) | 0.270 | 0.594 |
| Left Putamen | rs731170 | 0.090(-0.005-0.184) | 0.063 | 0.334 |
|  | rs1048801 | -0.033(-0.122-0.057) | 0.471 | 0.715 |
|  | rs1749316 | -0.079(-0.181-0.022) | 0.128 | 0.334 |
|  | rs1749317 | -0.089(-0.184-0.007) | 0.070 | 0.334 |
|  | rs1925241 | -0.014(-0.100-0.073) | 0.755 | 0.755 |
|  | rs2569715 | -0.017(-0.111-0.077) | 0.721 | 0.755 |
|  | rs2569716 | 0.022(-0.070-0.114) | 0.636 | 0.755 |
|  | rs3745871 | -0.029(-0.118-0.060) | 0.520 | 0.715 |
|  | rs11540761 | -0.083(-0.188-0.022) | 0.122 | 0.334 |
|  | rs11574576 | -0.062(-0.157-0.032) | 0.196 | 0.359 |
|  | rs28366008 | 0.008(-0.099-0.114) | 0.152 | 0.334 |
| Left Anterior of Putamen | rs731170 | 0.100(0.001-0.200) | **0.049** | 0.475 |
|  | rs1048801 | -0.019(-0.113-0.075) | 0.694 | 0.763 |
|  | rs1749316 | -0.066(-0.173-0.042) | 0.233 | 0.475 |
|  | rs1749317 | -0.070(-0.171-0.031) | 0.177 | 0.475 |
|  | rs1925241 | -0.026(-0.117-0.066) | 0.580 | 0.709 |
|  | rs2569715 | -0.045(-0.144-0.054) | 0.376 | 0.517 |
|  | rs2569716 | 0.015(-0.082-0.112) | 0.765 | 0.765 |
|  | rs3745871 | -0.046(-0.140-0.047) | 0.333 | 0.517 |
|  | rs11540761 | -0.073(-0.184-0.038) | 0.199 | 0.475 |
|  | rs11574576 | -0.058(-0.157-0.042) | 0.259 | 0.475 |
|  | rs28366008 | 0.071(-0.042-0.184) | 0.217 | 0.475 |
| Right Putamen | rs731170 | 0.120(0.024-0.216) | **0.015** | 0.161 |
|  | rs1048801 | 0.016(-0.076-0.107) | 0.737 | 0.811 |
|  | rs1749316 | -0.114(-0.218--0.010) | **0.031** | 0.173 |
|  | rs1749317 | -0.058(-0.156-0.040) | 0.243 | 0.446 |
|  | rs1925241 | -0.005(-0.093-0.084) | 0.913 | 0.913 |
|  | rs2569715 | -0.022(-0.118-0.074) | 0.656 | 0.811 |
|  | rs2569716 | 0.021(-0.073-0.115) | 0.664 | 0.811 |
|  | rs3745871 | -0.021(-0.112-0.069) | 0.644 | 0.811 |
|  | rs11540761 | -0.069(-0.176-0.038) | 0.221 | 0.446 |
|  | rs11574576 | -0.072(-0.168-0.024) | 0.143 | 0.393 |
|  | rs28366008 | 0.086(-0.023-0.195) | 0.122 | 0.393 |
| Right Anterior of Putamen | rs731170 | 0.124(0.022-0.226) | **0.017** | 0.190 |
|  | rs1048801 | 0.017(-0.080-0.114) | 0.727 | 0.889 |
|  | rs1749316 | -0.110(-0.220-0.000) | **0.050** | 0.274 |
|  | rs1749317 | -0.065(-0.168-0.039) | 0.222 | 0.513 |
|  | rs1925241 | -0.010(-0.104-0.084) | 0.836 | 0.916 |
|  | rs2569715 | -0.005(-0.107-0.096) | 0.916 | 0.916 |
|  | rs2569716 | 0.025(-0.075-0.124) | 0.628 | 0.884 |
|  | rs3745871 | -0.023(-0.119-0.073) | 0.643 | 0.884 |
|  | rs11540761 | -0.061(-0.175-0.053) | 0.295 | 0.541 |
|  | rs11574576 | -0.093(-0.195-0.010) | 0.077 | 0.282 |
|  | rs28366008 | 0.070(-0.045-0.186) | 0.233 | 0.513 |

CI, Confidence internal; DAT, dopamine transporter; FDR, false discovery rate

**Supplementary Table 13**. Model 1: The correlation between *LILRB4* loci and DAT-SPECT striatal binding ratios in female.

| Items | SNP | β(95%CI) | P | FDR-corrected. P |
| --- | --- | --- | --- | --- |
| Left Caudate | rs731170 | 0.123(-0.026-0.272) | 0.108 | 0.592 |
|  | rs1048801 | 0.017(-0.125-0.158) | 0.820 | 0.889 |
|  | rs1749316 | -0.068(-0.223-0.087) | 0.394 | 0.722 |
|  | rs1749317 | -0.074(-0.225-0.077) | 0.338 | 0.722 |
|  | rs1925241 | -0.026(-0.162-0.111) | 0.713 | 0.889 |
|  | rs2569715 | -0.087(-0.226-0.052) | 0.222 | 0.722 |
|  | rs2569716 | -0.024(-0.166-0.119) | 0.745 | 0.889 |
|  | rs3745871 | -0.067(-0.207-0.073) | 0.352 | 0.722 |
|  | rs11540761 | -0.147(-0.321-0.027) | 0.098 | 0.592 |
|  | rs11574576 | 0.051(-0.092-0.195) | 0.483 | 0.759 |
|  | rs28366008 | 0.012(-0.153-0.177) | 0.889 | 0.889 |
| Right Caudate | rs731170 | 0.246(0.099-0.392) | **0.001** | **0.013** |
|  | rs1048801 | -0.001(-0.143-0.140) | 0.984 | 0.984 |
|  | rs1749316 | -0.078(-0.233-0.076) | 0.321 | 0.589 |
|  | rs1749317 | -0.151(-0.301--0.001) | **0.049** | 0.117 |
|  | rs1925241 | -0.134(-0.269-0.001) | 0.053 | 0.117 |
|  | rs2569715 | -0.044(-0.183-0.095) | 0.535 | 0.654 |
|  | rs2569716 | -0.022(-0.164-0.120) | 0.764 | 0.840 |
|  | rs3745871 | -0.169(-0.308--0.031) | **0.017** | 0.067 |
|  | rs11540761 | -0.209(-0.381--0.037) | **0.018** | 0.067 |
|  | rs11574576 | 0.059(-0.084-0.202) | 0.422 | 0.592 |
|  | rs28366008 | 0.066(-0.098-0.230) | 0.431 | 0.592 |
| Left Putamen | rs731170 | 0.116(-0.021-0.253) | 0.099 | 0.359 |
|  | rs1048801 | 0.070(-0.060-0.200) | 0.290 | 0.399 |
|  | rs1749316 | -0.029(-0.172-0.113) | 0.687 | 0.687 |
|  | rs1749317 | -0.057(-0.196-0.081) | 0.418 | 0.460 |
|  | rs1925241 | -0.061(-0.186-0.065) | 0.343 | 0.419 |
|  | rs2569715 | -0.116(-0.243-0.011) | 0.075 | 0.359 |
|  | rs2569716 | -0.077(-0.208-0.054) | 0.250 | 0.399 |
|  | rs3745871 | -0.099(-0.228-0.029) | 0.131 | 0.359 |
|  | rs11540761 | -0.154(-0.313-0.006) | 0.060 | 0.359 |
|  | rs11574576 | 0.084(-0.048-0.216) | 0.214 | 0.399 |
|  | rs28366008 | 0.083(-0.069-0.234) | 0.285 | 0.399 |
| Left Anterior of Putamen | rs731170 | 0.131(-0.016-0.277) | 0.082 | 0.453 |
|  | rs1048801 | 0.049(-0.090-0.188) | 0.490 | 0.587 |
|  | rs1749316 | -0.040(-0.192-0.113) | 0.610 | 0.610 |
|  | rs1749317 | -0.062(-0.210-0.087) | 0.415 | 0.587 |
|  | rs1925241 | -0.071(-0.205-0.063) | 0.300 | 0.550 |
|  | rs2569715 | -0.095(-0.232-0.041) | 0.172 | 0.457 |
|  | rs2569716 | -0.049(-0.189-0.092) | 0.498 | 0.587 |
|  | rs3745871 | -0.104(-0.242-0.033) | 0.137 | 0.457 |
|  | rs11540761 | -0.013(-0.030-0.004) | 0.069 | 0.453 |
|  | rs11574576 | 0.091(-0.050-0.232) | 0.208 | 0.457 |
|  | rs28366008 | 0.052(-0.111-0.214) | 0.534 | 0.587 |
| Right Putamen | rs731170 | 0.159(0.022-0.296) | **0.024** | 0.129 |
|  | rs1048801 | 0.077(-0.053-0.207) | 0.248 | 0.425 |
|  | rs1749316 | -0.059(-0.202-0.084) | 0.421 | 0.463 |
|  | rs1749317 | -0.046(-0.186-0.093) | 0.516 | 0.516 |
|  | rs1925241 | -0.071(-0.196-0.055) | 0.271 | 0.425 |
|  | rs2569715 | -0.107(-0.234-0.021) | 0.104 | 0.285 |
|  | rs2569716 | -0.064(-0.195-0.068) | 0.343 | 0.463 |
|  | rs3745871 | -0.110(-0.239-0.019) | 0.095 | 0.285 |
|  | rs11540761 | -0.189(-0.349--0.030) | **0.021** | 0.129 |
|  | rs11574576 | 0.059(-0.073-0.191) | 0.384 | 0.463 |
|  | rs28366008 | 0.113(-0.038-0.265) | 0.145 | 0.319 |
| Right Anterior of Putamen | rs731170 | 0.208(0.063-0.353) | **0.005** | 0.057 |
|  | rs1048801 | 0.039(-0.100-0.178) | 0.585 | 0.585 |
|  | rs1749316 | -0.078(-0.230-0.074) | 0.315 | 0.433 |
|  | rs1749317 | -0.098(-0.246-0.049) | 0.193 | 0.304 |
|  | rs1925241 | -0.106(-0.239-0.028) | 0.122 | 0.284 |
|  | rs2569715 | -0.099(-0.235-0.037) | 0.155 | 0.284 |
|  | rs2569716 | -0.057(-0.197-0.082) | 0.423 | 0.465 |
|  | rs3745871 | -0.136(-0.273-0.000) | 0.051 | 0.188 |
|  | rs11540761 | -0.222(-0.391--0.053) | **0.011** | 0.058 |
|  | rs11574576 | 0.059(-0.082-0.200) | 0.411 | 0.465 |
|  | rs28366008 | 0.117(-0.044-0.279) | 0.155 | 0.284 |

CI, Confidence internal; DAT, dopamine transporter; FDR, false discovery rate
